# Tachyporinae Revisited: Phylogeny, Evolution, and Higher Classification Based on Morphology, with Recognition of a New Rove Beetle Subfamily (Coleoptera: Staphylinidae)

**DOI:** 10.3390/biology10040323

**Published:** 2021-04-13

**Authors:** Shûhei Yamamoto

**Affiliations:** 1 Integrative Research Center, Field Museum of Natural History, 1400 S Lake Shore Drive, Chicago, IL 60605, USA; syamamoto@fieldmuseum.org; 2 The Hokkaido University Museum, Hokkaido University, Kita 8, Nishi 5, Kita-ku, Sapporo 060-0808, Japan

**Keywords:** Tachyporinae, Mycetoporinae, Tachyporini, Vatesini, Deropini, Tachinusini, revised classification, phylogeny, systematics, identification keys

## Abstract

**Simple Summary:**

The rove beetle subfamily Tachyporinae has been suggested to be polyphyletic for the last half century but there are no previous studies conducting phylogenetic analysis on this group specifically. Here, the most comprehensive tachyporine phylogeny is shown, which again rejects the monophyly of Tachyporinae and its largest tribe Tachyporini. A revised classification of Tachyporinae is proposed here based on observation of morphological characters and their phylogenetic analyses. This backbone phylogeny will be a framework for further evolutionary and ecological studies.

**Abstract:**

Tachyporinae are one of the most phylogenetically problematic subfamilies in the mega-diverse rove beetle family Staphylinidae. Despite its high diversity and abundance in forest micro-environments, with over 1600 species worldwide, several previous studies had refuted the monophyly of this subfamily and its largest tribe, Tachyporini. Based on the results of morphology-based phylogenetic analyses and direct examination of specimens encompassing two extinct and all forty extant genera, a new suprageneric classification of Tachyporinae is proposed here, with the removal of the tribe Mycetoporini into a newly recognized subfamily Mycetoporinae stat. nov. Four tribes with two subtribes are arranged within Tachyporinae sensu nov.: Tachyporini sensu nov. (Tachyporina stat. nov., sensu nov. and Euconosomatina stat. rev., sensu nov.), Vatesini sensu nov., Deropini, and Tachinusini stat. rev., sensu nov. (= Megarthropsini syn. nov.). *Urolitus* syn. nov. is placed as a junior synonym of *Sepedophilus*. Additionally, *Palporus* stat. nov. is raised to a distinct genus from a subgenus of *Tachyporus* sensu. nov., and †*Mesotachyporus* syn. nov. is synonymized with the latter. Mycetoporine *Bobitobus* stat. rev. is resurrected from synonymy with *Lordithon* sensu nov., and considered as a valid genus. My revised classification provides a novel framework for taxonomic inventories and ecological studies of these groups.

## 1. Introduction

Staphylinidae, whose members are commonly called rove beetles, is the largest family of the animal kingdom, with an incredible diversity of 65,561 living and 450 extinct species as of 4 March 2021 (A.F. Newton, pers. comm.). Among one extinct and 32 extant subfamilies [1], Tachyporinae is a medium-sized group at the subfamily level and currently comprises 1638 species (28 extinct) of 52 genera (12 extinct) in five tribes as of 9 April 2021 (A.F. Newton, pers. comm.; Appendix A). The family Staphylinidae is so diverse and species-rich, it has been further subdivided into four or five groups of subfamilies [2,3]. The tachyporines have been placed in the Tachyporine Group of subfamilies [4,5]. However, none of these groupings are supported as monophyletic (e.g., [6,7]), and they are all in need of further investigation and proper grouping.

Tachyporinae are distributed throughout all zoogeographical regions except for Antarctica (Table A1 and Table A2). At the genus level, many have relatively wide distributional ranges, as listed in Table A1 and Table A2. Interestingly, *Leucotachinus* Coiffait & Sáiz is distributed disjunctly in South America and Australia, showing a Gondwanan relictual distribution similar to that of the *Glypholoma* rove beetles [8], Lampriminae stag beetles [9], and moss bugs (Hemiptera: Coleorrhyncha) [10]. In contrast, the members of the current tachyporine tribe Mycetoporini indicate more or less Palearctic or Holarctic distributions, although there are a few cosmopolitan genera (Table A2). Similarly, several genera are limited to one biogeographic region as seen in *Vatesus* Sharp and *Olophrinus* Fauvel (Table A1). Many genera are rarely collected, sometimes represented only by a handful of occurrences from the original descriptions (e.g., [11,12]). There are still many undescribed species globally, especially within the genera *Sepedophilus* Gistel, *Tachinus* Gravenhorst, or *Coproporus* Kraatz. Therefore, more new species will continuously be described in the future.

**Figure 1:**
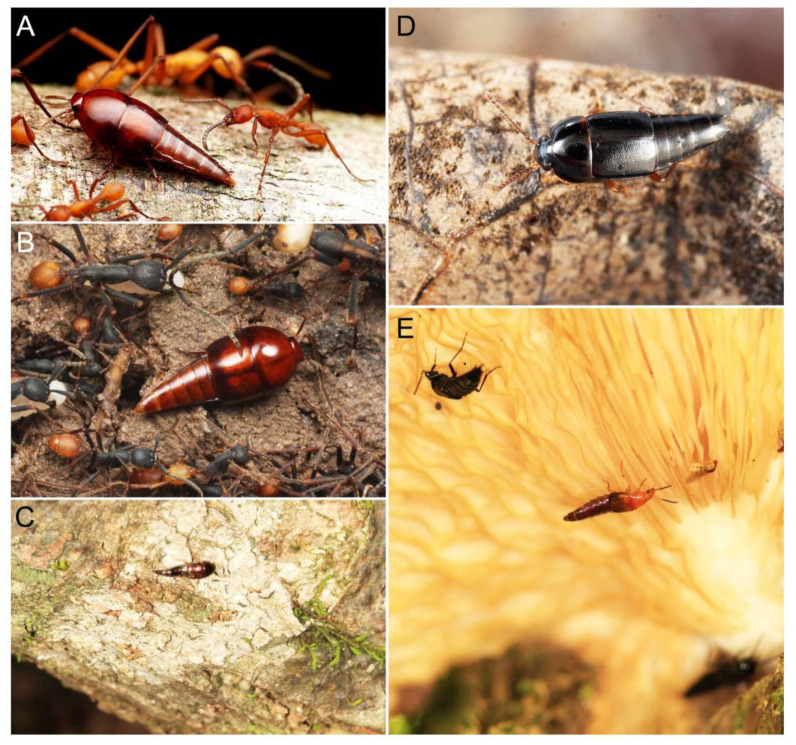
Photos of Tachyporinae adults in their corresponding microhabitats. (**A**) *Vatesus* sp. 1 (Vatesini) associated with the army ant species *Eciton hamatum* (Fabricius) in French Guiana. (**B**) *Vatesus* sp. 2 (Vatesini) associated with the army ant species *Eciton burchellii* (Westwood) in Peru. (**C**) *Sepedophilus* sp. belonging to *S*. *crassus* species group (Tachyporini) in Kumamoto Pref., Japan. (**D**) *Tachinus* (*Tachinoderus*) sp. (Tachyporini, now Tachinusini) in Shimane Pref., Japan. (**E**) *Lordithon* (*Lordithon*) *bicolor* (Gravenhorst) (Mycetoporini, now Mycetoporinae) in Kumamoto Pref., Japan. Photo credits: Taku Shimada (**A**,**B**) both used with permission) and Masakazu Hayashi (**D**), used with permission).

Tachyporines are abundant and frequently encountered in forest-associated environments. A characteristic morphological feature is their tapered abdomen as compared to other rove beetles, so they are rather easily recognized to subfamily in insect inventories, faunal investigations, and ecological surveys. Nevertheless, little has been known regarding their biology and ecological habits, though some are thought to be mycophagous or active predators on other arthropods [3]. Most tachyporine species are small to medium in size, commonly found in micro-environments, such as leaf litter, fungi, bark, or dead wood (Figure 1). The genus *Vatesus* (Figure 1A,B) is famous for its myrmecophilous lifestyle with army ants in Neotropical rain forests [13,14]. Such remarkable ecology resulted in modifications of the overall body structures, including an exaggerated limuloid morphology with a hugely expanded pronotum that has protective functions against worker ants [15]. A few members, e.g., *Derops* Sharp or *Nitidotachinus* Campbell, are found in very wet conditions, often along rivers and streams [16]. *Sepedophilus* has been targeted by evolutionary studies as its feeding habit switches between predatory and mycophagy within the genus for both larvae and adults [17,18]. A recent study suggests that some members of *Sepedophilus* may be effective pollinators of two Japanese plant species of *Arisaema* (Araceae) because of their extremely high visitation frequency [19].

Five tribes are currently included in Tachyporinae [20]: Deropini Smetana, Megarthropsini Cameron, Mycetoporini Thomson, Tachyporini MacLeay, and Vatesini Seevers. Although Herman [21] recognized two more tribes, i.e., Symmixini Bernhauer and Cordobanini Bernhauer, within Tachyporinae, the former is now synonymized under Tachyporini [22], and the latter has been transferred to the subfamily Aleocharinae [20]. Three tribes, namely Deropini, Megarthropsini, and Vatesini, are each monogeneric or containing a small group of distinctive tachyporines, resulting focused taxonomic investigations [13,23,24,25]. A number of systematic studies on the remaining tribes of Tachyporinae have been published, but they are mostly restricted geographically and/or taxonomically. One of these tribes, Tachyporini, is by far the largest tribe with more than 1000 species, comprising the vast majority of Tachyporinae in terms of the number of species.

In spite of their high species richness, Tachyporinae are known as one of the most problematic subfamilies in Staphylinidae, because they are likely a non-monophyletic, and probably polyphyletic, lineage ([3]: Figure 14.30). In fact, several phylogenetic analyses have refuted the monophyly of Tachyporini, Tachyporinae, and even the Tachyporine Group. Non-monophyly of Tachyporinae has been suggested since mid-1970s [26], but Ashe & Newton [27] first indicated the polyphyly of Tachyporinae and Tachyporini on the basis of 27 larval characters with limited taxon sampling. Later, Herman [25] used a phylogenetic approach to examine the internal relationships of the genera of Megarthropsini and assessed the tribe’s systematic position among the other tachyporine tribes. In his tree, the monophyly of Tachyporini was unsupported based on 19 adult characters with very limited taxon sampling [25]. Notably, the Tachyporini genus *Coproporus* formed a sister group with the Vatesini genus *Vatesus* in both Ashe & Newton [27] and Herman [25]. Recent molecular studies with extensive taxon sampling of Staphylinoidea have significantly contributed in clarifying internal phylogenies within Staphylinidae and also Tachyporinae. McKenna et al. [6] proposed one of the largest phylogenetic trees of Staphylinidae including three tachyporine tribes, i.e., Deropini, Tachyporini, and Mycetoporini. As a result, Deropini and Tachyporini were recovered together, sister unexpectedly to the carrion beetle family Silphidae, but quite distant from the monophyletic Mycetoporini [6]. Similar results were also obtained in the largest phylogenies of Staphylinoidea [7], in which *Vatesus* (Vatesini) was clustered with Deropini and Tachyporini. In spite of the dense taxon sampling in these two studies, no representative of Megarthropsini was included [6,7].

One of the major reasons for a lack of comprehensive tachyporine phylogeny is the difficulty of taxon sampling. Members of Tachyporini are frequently encountered, but belong mostly to the four larger genera, i.e., *Sepedophilus*, *Tachinus*, *Coproporus*, or *Tachyporus* Gravenhorst. Many other genera are rarely collected, some with only handful of records including the original descriptions. This led to insufficient taxon sampling in phylogenetic studies of the subfamily. Additionally, many of the tachyporine genera have not been adequately explored morphologically, resulting in a significant lack of information useful to constructing phylogenetic trees or generic identification aids. Since there is no previous study targeted at inferring the phylogeny of the entire Tachyporinae subfamily, a large-scale phylogeny of this subfamily including all five tribes is not available at the present, but it is strongly desired in order to determine whether Mycetoporini is a true member of Tachyporinae. To address these issues, I tried to test the monophyly, assess sister group relationships with and within Tachyporinae, and provide a new framework for the subfamily. Herein, the first comprehensive phylogeny and a fully updated higher classification of Tachyporinae are provided based on results of phylogenetic analyses of adult morphological characters and direct examination of specimens encompassing two extinct and all forty extant genera. Finally, several fossil genera with uncertain systematic placements are incorporated into proper taxonomic categories. By reducing phylogenetic uncertainty, my study will contribute to the basic foundation for taxonomic, ecological, and evolutionary studies in the mega-diverse Staphylinidae tree of life.

## 2. Materials and Methods

### 2.1. General Outline of Study

The main focus of the present study was to provide a backbone phylogeny of Tachyporinae and to assess sister group relationships of or within the subfamily. Despite the importance of genomic data, I decided to use only adult morphological characters here. One of the reasons is the difficulty of collecting samples from various tachyporine genera. Because the tachyporines include so many rare taxa, it would be quite challenging to obtain molecular samples or even conventional dried, pinned specimens for these taxa. Larval characters generally contain vital information for morphology-based phylogeny in rove beetles (e.g., [2,28,29]). However, the larvae of Tachyporinae are far from adequately understood, resulting in a significant lack of larval information for most genera of Tachyporinae. Even within large genera such as *Sepedophilus*, *Coproporus*, or *Tachinus*, accurate identifications of species considering the larva-adult associations are generally difficult, with a high degree of uncertainty in identification. Taking these conditions into account, my morphological dataset is derived from four different sources: (i) pinned specimens, (ii) wet specimens preserved in 70–80% EtOH (ethyl alcohol) solution, (iii) permanent slide specimens, and (iv) literature (Supplementary Files S1 and S2). Therefore, it was possible to include as many extant tachyporine genera as possible in this study. As Tachyporinae may be a polyphyletic lineage, this approach is currently the best strategy to infer the higher-level phylogeny by reducing the number of potentially phylogenetic “distinct” taxa, i.e., each could represent as own higher-taxonomic category (e.g., tribe). The morphological definition for each tribe has not been well established, thus a morphology-based approach is also desired to solve this issue, rather than relying on a smaller taxon sampling based on molecular data, but such a DNA-oriented study is desired in future.

### 2.2. Taxon Sampling and Deposition of Material

All forty extant genera in five tribes of Tachyporinae were used in this study. Although two very rare genera, *Tachinoporus* Cameron and *Tachinoproporus* Cameron, were not included in the formal phylogenetic analyses, the type specimens of these taxa were examined and are redescribed below. This is because I could only observe a subset of the characters from dorsal and lateral views, with limited dissected body parts. The dissected parts were generally covered with glue or distorted, and it may have caused erroneous scoring of characters. Therefore, I decided to remove these two taxa from the analyses, but the outline of Tachyporinae still can be drawn without the inclusion of these two taxa. For most terminal taxa in phylogenetic analyses, morphological data were obtained at least partially from direct observation, with the exception of *Urolitus* Silvestri (from literature only). In addition, two fossil tachyporine genera, namely †*Mesotachyporus* Gusarov and †*Procileoporus* Yamamoto, were directly examined based on the holotypes of each type species, whereas the remaining eight fossil genera were studied from references to evaluate their systematic assignments.

A total of 70 operational taxonomic units (OTUs) were scored for the cladistic analyses, comprised of 57 species in 38 genera of Tachyporinae and 13 representatives of ten other selected subfamilies of Staphylinidae, together with one Silphidae (subfamily Silphinae), as outgroups (Supplementary Files S1 and S2). At least one representative for each subfamily of the Tachyporine Group was included: Aleocharinae, Habrocerinae, Olisthaerinae, Phloeocharinae, and Trichophyinae. More outgroups were carefully chosen based on the results of McKenna et al. [6] and Lü et al. [7], namely Neophoninae, Omaliinae, Osoriinae, Pseudopsinae, Staphylininae, and Silphidae.

Material from the following collections was examined: American Museum of Natural History, New York, USA (AMNH: D. Grimaldi); Field Museum of Natural History, Chicago, IL, USA (FMNH: C. Maier, J. Snyder, or A. F. Newton for wet and slide collections); Kyushu University Museum, Fukuoka, Japan (KUM: M. Maruyama); Muséum d’histoire naturelle de Genève, Geneva, Switzerland (MHNG: G. Cuccodoro); National Museum of Nature and Science, Tsukuba, Japan (NSMT: S. Nomura, private collection); Natural History Museum, London, London, UK (BMNH: M. Geiser); Natural History Museum Vienna, Vienna, Austria (NHMW: H. Schillhammer).

The original higher classification follows Bouchard et al. [20]. A complete list of the taxa used with their collecting data is shown in Supplementary Files S1 and S2. To enhance the understanding the morphological details of Tachyporinae, a series of photographs is shown in the paper.

### 2.3. Microscopy, Imaging, Measurements, and Terminology

Observations of dry and wet specimens and some permanent slides were made under a Leica MZ16 stereomicroscope (Leica Microsystems, Wetzlar, Germany). Amber fossils were examined using either a Leica MZ16 or a Nikon SMZ1500 stereomicroscope (Nikon, Tokyo, Japan) in air, without immersion in oil or similar liquid. Photographs of dry specimens were taken using an EOS 80D digital camera (Canon, Tokyo, Japan) mounted on an extreme macro lens (Canon MP-E 65 mm, F2.8, 1–5×; Canon) with a flash (Canon Macro Twin Lite MT-24EX Flash; Canon) as light source. The dissected body parts of *Tachinoproporus* were photographed with the Dun Ink BK PLUS Lab System (Dun, Palmyra, VA, USA) attached to a 6D digital camera (Canon) and a 10× lens. A single amber fossil of †*Mesotachyporus* was photographed by David Grimaldi (AMNH) using a Nikon Digital Sight DS-R1i camera (Nikon, Tokyo, Japan) as an attachment of a Nikon SMZ1500 stereomicroscope. Slide specimens were imaged using an EOS 80D camera and an AmScope CA-CAN-SLR Canon SLR/DSLR Camera Adapter for Microscopes (AmScope Microsystem, USA), mounted on an Olympus BX50 stereomicroscope (Olympus Corp., Tokyo, Japan). Montage images were later produced using Helicon Focus 7.5.4 (Helicon Soft, Kharkiv, Ukraine), but †*Mesotachyporus* images were optimized with NIS-Elements D (Nikon). All images were edited and assembled in plates in Photoshop Elements 15 (Adobe Systems, San Jose, CA, USA). The measurements are given in millimetres. The total body length was measured from the apex of the clypeus to the posterior end of the abdomen. The maximum lengths were measured in the head, pronotum, and each elytron (elytral lengths were measured from each elytron with the maximum lengths and widths). The ratios of elytral length and width were calculated basically based on dissected specimens. Some diagnostic characters are indicated with arrows and/or character numbers on the figures. For descriptions and character list, I generally followed the morphological terminology of Blackwelder [30], Herman [25], and Ashe [28]. The age of Baltic amber is controversial from middle to late Eocene, but I followed the mid-Eocene (Lutetian: 44.1 ± 1.1 Ma) age in this study based on the most recent estimations obtained by the absolute dating analyses of glauconites from the “Blaue Erde” (blue earth) layer in Sambia Peninsula [31]. The higher-resolution figure plates used in this study have been deposited in the figshare (https://doi.org/10.6084/m9.figshare.14179529; accessed on 9 April 2021) and Zenodo repositories (https://doi.org/10.5281/zenodo.4660863; accessed on 9 April 2021). This published work has been registered in ZooBank, with the following LSIDs (Life Science Identifiers): urn:lsid:zoobank.org:pub:8F45A3EA-4193-49D3-BF57-A65332D3262C.

### 2.4. Selection of Characters

One of the most challenging points in constructing the tachyporine phylogeny based on morphology is the selection of characters. Since there is no study specifically focused on the entire Tachyporinae with dense sampling of taxa and characters, I needed to extract some characters from the literature treating mainly non-Tachyporinae such as Ashe [28] or Grebennikov & Newton [2]. Consequently, most characters used here are new and original for Tachyporinae. Some characters used in generic identification were not adopted in this study such as the conditions of spines at the apices of the meso- and metatibiae, pronotal chaetotaxy, or the basomedial carina of the scutellum in Mycetoporini (e.g., [32,33]) because of the significant variations found in a broad set of the outgroups and difficulty to accurately assess these characters in Tachyporinae s. str. Accordingly, this study intended to reveal the relationship between Mycetoporini and the rest of Tachyporinae, and the internal tribal relationship in Tachyporinae s. str., rather than the generic relationships within Mycetoporini.

As a result of my extensive morphological investigations on Tachyporinae and the other staphylinoid outgroups, the final character list is comprised of a total of 156 binary and multi-state characters, taken from various parts of the adult body: 18 from head, 4 from antennae, 26 from mouthparts, 56 from thorax and legs, 36 from abdomen, and 16 from genitalia (10 from male, 6 from female). To evaluate generic level characters, possible autapomorphies for some genera were also included. In the present paper, I also aim to provide morphological resources for diagnostic key characters in Tachyporinae. Thus, habitus photographs representing all extant genera are shown here, together with diverse characters across all body parts of the selected taxa and their associated type-specimen labels for some taxa. The complete list of characters with explanations and post-evaluations is given in the Appendix B.

### 2.5. Phylogenetic Analyses

The character matrix was created in Mesquite version 3.61 [34], with missing character states coded as ‘?’. My final data matrix included 156 characters scored for 70 terminal taxa (57 ingroup Tachyporinae, 13 non-tachyporine outgroups). The nexus formatted file containing the character matrix with the resulting phylogenetic trees is provided in Supplementary File S3, with the addition of TNT format matrix as Supplementary File S4. In all analyses, *Lispinus quadripunctulus* Fauvel, 1864 [35] (Osoriinae: Thoracophorini) was chosen to root the trees, considering the results of McKenna et al. [6]. This decision seems to be reasonable after testing different outgroups to root the trees as *L. quadripunctulus* resulted in smallest number of trees. The software TNT version 1.5 [36] was used for maximum parsimony (MP) analysis using the ‘New Technology search’ option to find the most parsimonious trees (MPTs) with all characters treated as unordered and equally weighted. In order to estimate branch support on a cladogram, I calculated Bootstrap support values generated via TNT version 1.5 under the following parameters: memory set to hold 99,999 trees; slack for sectors set to 80 by entering a command line, “sect:slack 80”; ‘absolute frequencies’ under ‘new technology search’ option with 2000 replications, collapsing groups below 1. Character distributions were mapped using WinClada version 1.00.08 [37]. Nodes with bootstrap values were considered with the following criteria: bootstrap values (BV) > 90 were judged to be strongly supported; with BV = 90–70 moderately supported; with BV = 70–50 weakly supported, and; with BV < 50 unsupported.

## 3. Results

### 3.1. Phylogenetic Analyses

#### 3.1.1. Maximum Parsimony Analysis

**Figure 2:**
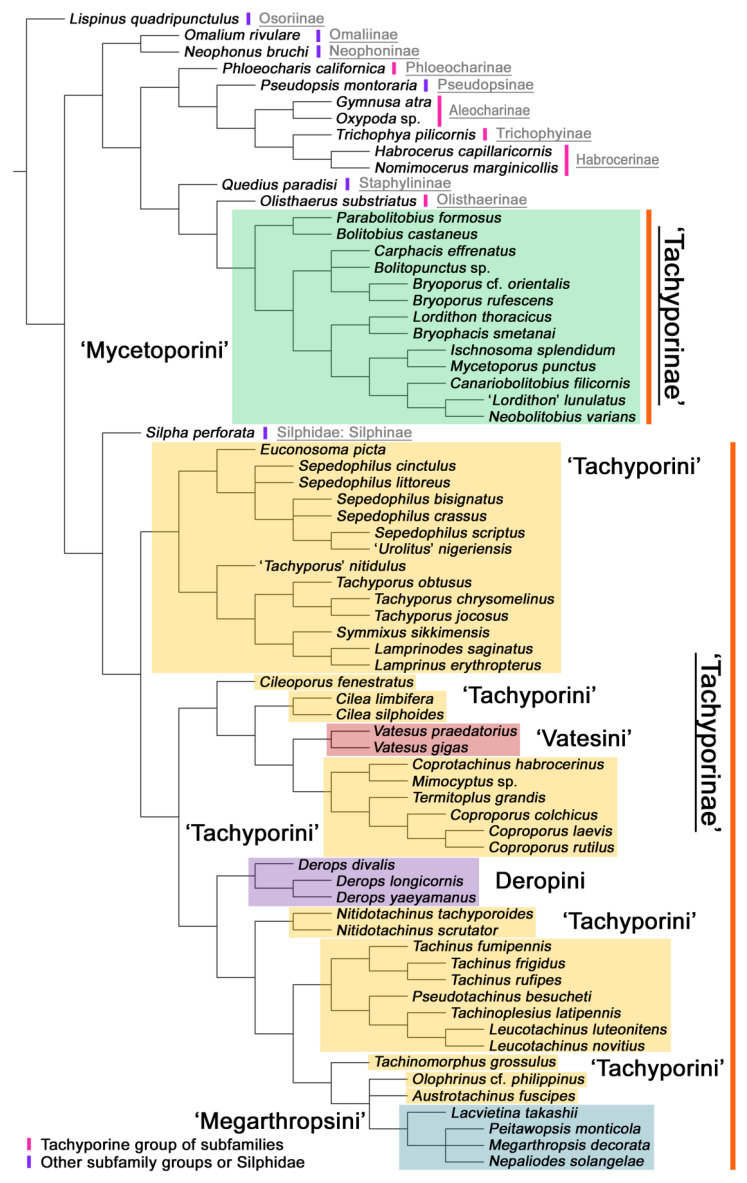
Results of the parsimony analysis of the phylogenetic relationships within Tachyporinae (strict consensus tree of the three obtained trees), showing non-monophyly of both Tachyporinae and Tachyporini. The family-group taxon names shown here reflect current usage.

**Figure 3:**
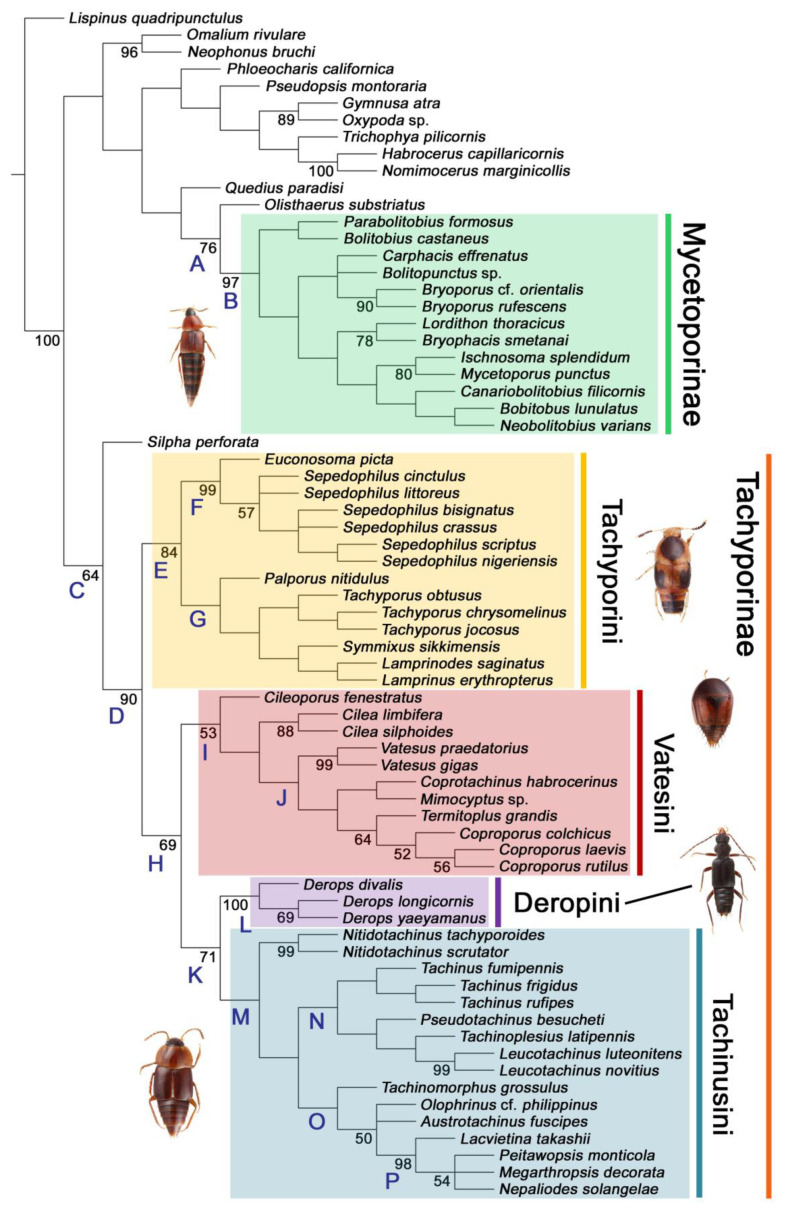
Results of the parsimony analysis of the phylogenetic relationships within Tachyporinae (strict consensus tree of the three obtained trees), refleccting my revised classification of Tachyporinae and its tribes. Each taxon name shown here reflects my taxonomic changes. A series of clades are named and used in the main text. Bootstrap support values > 50 are shown below branches.

**Figure 4:**
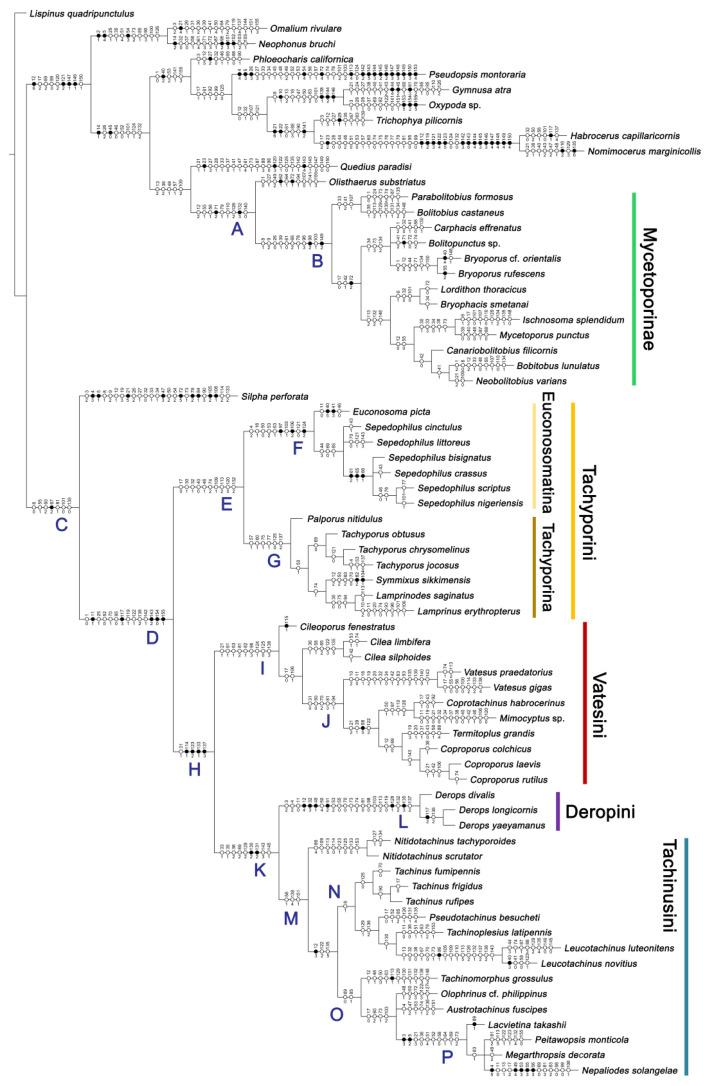
Results of the parsimony analysis of the phylogenetic relationships within Tachyporinae (strict consensus tree of the three obtained trees), with unambiguously optimized character changes plotted along the internodes. Black circles indicate unique character changes (unique synapomorphies), whereas white circles indicate parallelisms or reversals (homoplasious synapomorphies). Character numbers are given above circles, while character states are shown below circles. Each taxon name shown here reflects my taxonomic changes. A series of clades are named and used in the main text. Higher-resolution figure can be viewed at the figshare repository (https://doi.org/10.6084/m9.figshare.14179529; accessed on 9 April 2021).

Maximum parsimony analysis yielded only three most parsimonious trees, each with the following characteristics: tree length (TL) = 977, consistency index (CI) = 0.38, and retention index (RI) = 0.78. The resulting trees were largely congruent with previous studies, although many nodes were weakly supported or not supported. A strict consensus tree is shown here as the representative of the result (TL = 981, CI = 0.37, RI = 0.77; Figure 2, Figure 3 and Figure 4).

As in earlier studies [6,7], Tachyporinae were found to be polyphyletic and divided into two distant clades: one consisting of Mycetoporini (Figures 2–4, clade B in Figures 3 and 4) and the other containing the rest of the remaining tachyporines (Figures 2–4, clade D in Figures 3 and 4). Mycetoporini formed a sister group to *Olisthaerus* Dejean (Olisthaerinae; Figures 2–4, clade A in Figures 3 and 4) with moderate support, and these two combined was found to be sister to *Quedius* Stephens (Staphylininae). The Mycetoporini clade was strongly supported as a monophyletic group (Figure 3). Within Mycetoporini, two genera combined, i.e., *Parabolitobius* L.-Z. Li, M.-J. Zhao & Sakai + *Bolitobius* Leach, were revealed as sister to the rest of the mycetoporines. However, this relationship was not statistically supported (Figure 3). *Lordithon* Thomson was found to be polyphyletic (Figure 2), whereas *Bryoporus* Kraatz formed a monophyletic group (Figures 2–4).

**Figure 5:**
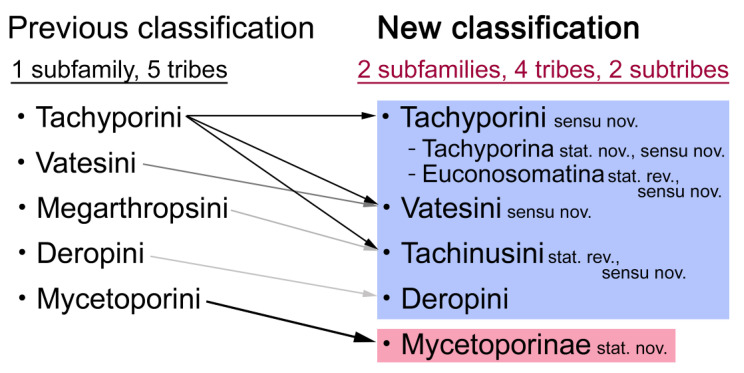
Summary of the previous and current taxonomic frameworks of Tachyporinae and its tribes. Each arrow with different coloration indicates the degree of taxonomic changes (darker arrow with more significant changes).

Tachyporinae, in the narrow sense, was also resolved as a monophylum with moderate support value (Figures 2–4, clade D in Figures 3 and 4), forming a sister group to *Silpha* Linnaeus (Silphidae) with weak support (Figures 2–4, clade C in Figures 3 and 4). The genera with more than two species included in the analysis were recovered as monophyletic, except that the enigmatic *Urolitus nigeriensis* and *Tachyporus nitidulus* (Fabricius) came out within *Sepedophilus* and outside *Tachyporus*, respectively, as in Figure 2. The tribe Tachyporini was shown here to be paraphyletic, divided into a total of nine clades (Figure 2). Two tribes, Vatesini and Megarthropsini, were each found to be monophyletic, but were resolved within parts of the paraphyletic Tachyporini (Figure 2). Tachyporini sensu str. (Figures 2–4, clade E in Figures 3 and 4), which contains the type genus *Tachyporus*, formed a sister group to the other Tachyporinae lineage comprised of Vatesini, Deropini, and Megarthropsini, and the rest of Tachyporini. Tachyporini sensu str. was moderately supported and divided into two clades, i.e., the unsupported *Tachyporus*-related genera (Figures 2–4, clade G in Figures 3 and 4) and the strongly supported *Sepedophilus*-related genera (Figures 2–4, clade F in Figures 3 and 4). This clade (clade E in Figures 3 and 4) is the moderately supported sister group of the weakly supported clade H (Figure 3), which includes Deropini and Megarthropsini in addition to the some Tachyporini genera related to *Coproporus* (Figures 2–4, clade I in Figures 3 and 4). *Vatesus*, the sole member of Vatesini, was strongly supported as monophyletic, belonging in the weakly supported clade I. In clade I, *Cileoporus* Campbell first diverged within this clade, followed by *Cilea* Jacquelin du Val, and *Vatesus* (Figures 2–4). The monophyletic *Coporoporus* formed a sister group relationship with *Termitoplus* (Figures 2–4). Subsequently, clade I was recovered as a sister group to clade K (Figures 3 and 4), wherein Deropini (clade L in Figures 3 and 4), Megarthropsini (clade P in Figures 3 and 4), and the remaining Tachyporini with the *Tachinus*-related genera were nested within it (Figures 2–4). Within clade M (Figures 3 and 4), *Nitidotachinus* Campbell was a sister taxon to all other members of this clade, and the strongly supported Megarthropsini (clade P in Figures 3 and 4) was resolved within it. Clade M (Figures 3 and 4) was further divided into two subclades: typical members of *Tachinus* and its allies (clade N in Figures 3 and 4) and atypical ones (clade O in Figures 3 and 4). Deropini was strongly supported as monophyletic, but the clade K (Figures 3 and 4) resulting from the combination of Deropini and clade M (Figures 3 and 4) has moderate support, and clade M alone was unsupported as were all but two of its included suprageneric subclades (Figures 2–4). Contrary to Herman [25], I found that Deropini is distantly related to Megarthropsini, rather than forming sister groups (Figure 2), although a direct comparison between the studies is difficult because of the different study design and taxonomic sampling. Based on observation of morphological characters and their phylogenetic analysis, a new classification is proposed, as shown in Figure 5.

#### 3.1.2. Character Optimization

The unambiguously optimized characters were mapped in Figure 4. Clade A (Figures 3 and 4), the sister group relationship between *Olisthaerus* (Olisthaerinae) and Mycetoporini was supported by two unique synapomorphies (71-2, elytra with three rows of setigerous punctures; 132-3, sternite VIII in posterior half covered with short to long setae in V-shaped punctures) and seven homoplasious synapomorphies. The distant tachyporine tribe Mycetoporini (clade B) was supported by two exclusive synapomorphies (98-2, metacoxae markedly large in each lateral half; 149-2, parameres each with a row of parameral setae at least partially aligned) and nine non-exclusive synapomorphies. Clade C (Figures 3 and 4), *Silpha* (Silphidae), and its inferred sister taxon to the remaining tachyporine genera was supported by one exclusive synapomorphy (67-2, mesospiracular peritremes (sensu Blackwelder [30]: Figure 3A,E) fully sclerotized, conspicuous and six non-exclusive synapomorphies. The true tachyporines, i.e., clade D (Figures 3 and 4), were supported by five unique synapomorphies (11-1, midcranial suture present; 117-3, female tergite VIII with lobes; 143-2, parameres with apical inner margins only narrowly separated longitudinally from each other; 154-0, gonocoxite II small in comparison with gonostylus; 155-1, gonocoxite II covered with curved setae) and nine homoplasious synapomorphies. Within clade (D) Tachyporini sensu str. (clade E in Figures 3 and 4) showed a sister group relationship to the remaining tachyporine members except Mycetoporini, and was supported by ten non-exclusive synapomorphies, such as strikingly densely setose maxillary palpomere 3 (32-1), pubescent ground microsetae on elytra (74-2), and tergites IV–V with macrosetae on posterolateral edges (109-1). Two subclades of clade E were the *Sepedophilus*-related genera (clade F in Figures 3 and 4) and *Tachyporus* and its relatives (clade G in Figures 3 and 4): the clade F was supported by three unique synapomorphies (e.g., 97-1, protibia with longitudinal row of closely spaced spines along outer margin; 124-2, abdominal segments V–VI without paratergites) and seven homoplasious synapomorphies (clade F), whereas the clade G (Figures 3 and 4) was supported by six homoplasious synapomorphies only (e.g., 57-1, pronotum with arranged stout setae; 75-1, elytron with macrosetae on median area; 125-0, sternite III without longitudinal median basal carina). Clade H was a relatively large monophylum which includes the members of Vatesini, Deropini, Megarthropsini, and the rest of Tachyporini, and it was supported by four unique synapomorphies (114-1, tergite VIII with only a few to several distinct macrosetae; 123-2, male tergite IX with only single to several prominent macrosetae, restricted to apex; 133-1, sternite VIII with only a few to several distinct macrosetae; 137-3, female sternite VIII with lobes, including small internal lobes) and one homoplasious synapomorphy. Clade I was comprised of Vatesini and the *Coproporus*-related genera of Tachyporini, supported by nine non-exclusive synapomorphies, including only weakly developed postcoxal process with rounded apex behind procoxae (61-1), thin, blade-like lateral and posterior margins of elytra (82-1), and distinctly large procoxae (96-3). Clade K (Figures 3 and 4), a sister group relationship between Deropini (clade L) and clade M, was supported by two exclusive synapomorphies (130-2, male sternite VII with moderately to strongly concave posteromedial margin; 131-2, male sternite VII with more than several to dozens of peg-like setae along posteromedial margin) and seven non-exclusive synapomorphies. Within clade K, Deropini was well supported by six unique synapomorphies (12-4, middle of postocular area distinctly narrowed, all around; 32-3, maxillary palpomere 3 completely glabrous; 49-1, pronotum moderately constricted in posterior half; 51-2, pronotum with uniform punctation dense, but shallow in apical half; 128-1, sternite VII along basomedial margin with a broad, semicircular protrusion; 135-3, male sternite VIII with posterior margin deeply incised or strongly emarginate medially) and additionally by 16 homoplasious synapomorphies. Compared to Deropini, its sister clade M (Figures 3 and 4), comprised of Megarthropsini and the *Tachinus*-related genera of Tachyporini, was weakly supported by only three non-exclusive synapomorphies, namely: 58-1, pronotum with transverse cluster of blackish to blackish brown, conspicuous small pores near posterior margin (Figure 54G); 138-4, female sternite VIII having both prominent lobes and inner lobes with rows of more than three, minute sensory setae in fan-like arrangement, located in apical area of each inner lobe; 151-1, spermatheca well sclerotized, comparatively complex or distinctively complicated structure, usually associated with coils. Megarthropsini (clade P in Figures 3 and 4) formed a sister clade to the genus *Austrotachinus* Steel. Clade P was supported by two unique synapomorphies (3-3, head with large punctation uniformly on vertex; 5-2, head with moderately rough or tuberculate surface) and eight homoplasious synapomorphies.

### 3.2. Systematic Part

Order Coleoptera Linnaeus, 1758 [38]

Superfamily Staphylinoidea Latreille, 1802 [39]

Family Staphylinidae Latreille, 1802 [39]

#### 3.2.1. Subfamily Tachyporinae MacLeay sensu nov.

Tachyporinae MacLeay, 1825: 49 [40] (Figures 1A–D, 2–61 and Figures S1 and S2B; Table A1)

Type genus: *Tachyporus* Gravenhorst, 1802 [41]: 124.

Differential diagnosis: Tachyporinae sensu. nov. differ from all other subfamilies of Staphylinidae based on the following combination of characters: body usually sub-limuloid, head small usually with midcranial suture, mandibles without inner teeth, elytron with epipleural keel folded inward, procoxae large, tarsal formula 5-5-5, abdomen with six visible sternites lacking ‘brick-wall’ pattern on intersegmental membranes, tergite VIII and sternite VIII frequently modified to form lobes.

**Figure 6:**
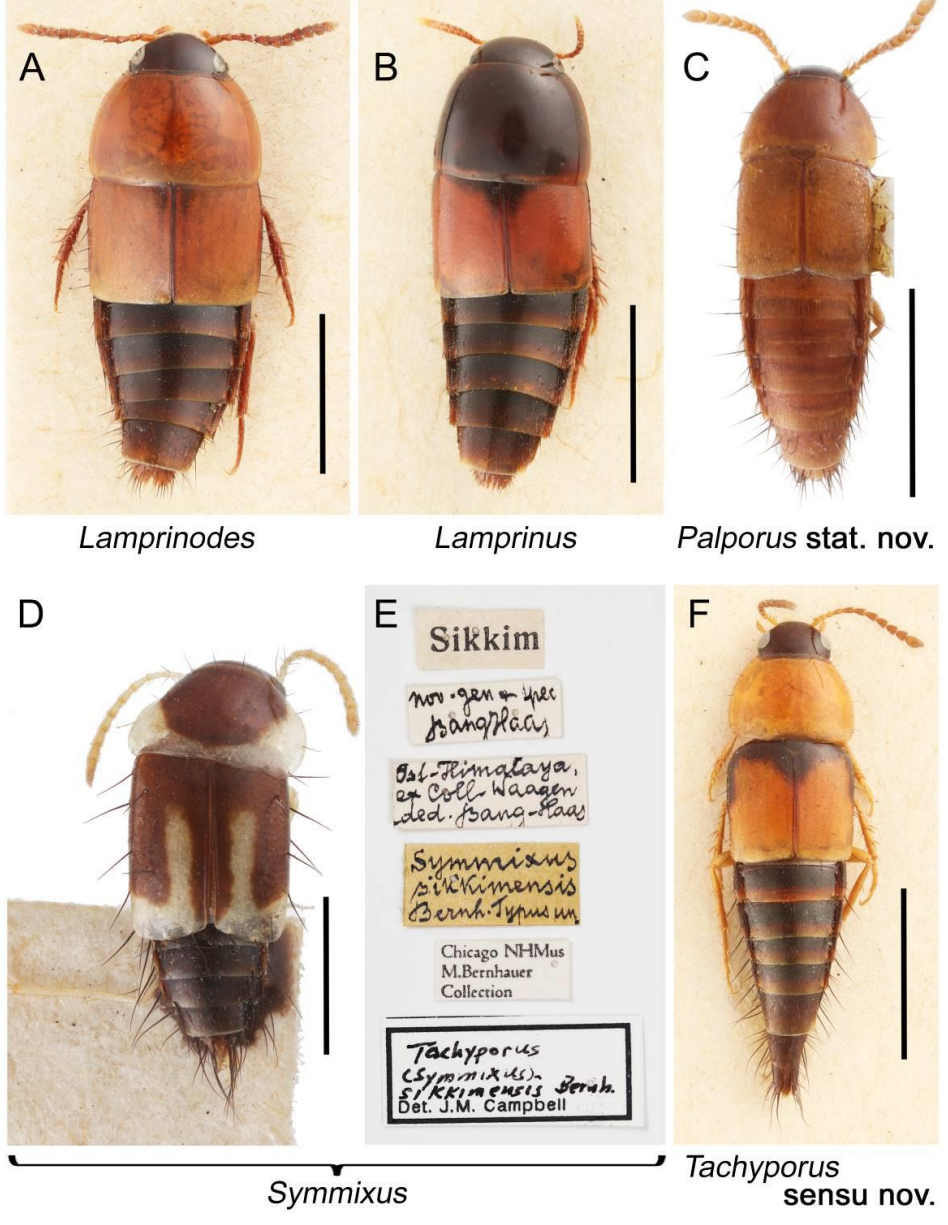
Habitus photographs of Tachyporini: Tachyporina, dorsal view. (**A**) *Lamprinodes saginatus* (Gravenhorst). (**B**) *Lamprinus erythropterus* (Panzer). (**C**) *Palporus nitidulus*. (**D**) *Symmixus sikkimensis*, holotype. (**E**) holotype labels of *Symmixus sikkimensis*. (**F**) *Tachyporus chrysomelinus*. Scale bars: 1.5 mm (**A**,**B**,**F**); 1.0 mm (**C**,**D**).

**Figure 7:**
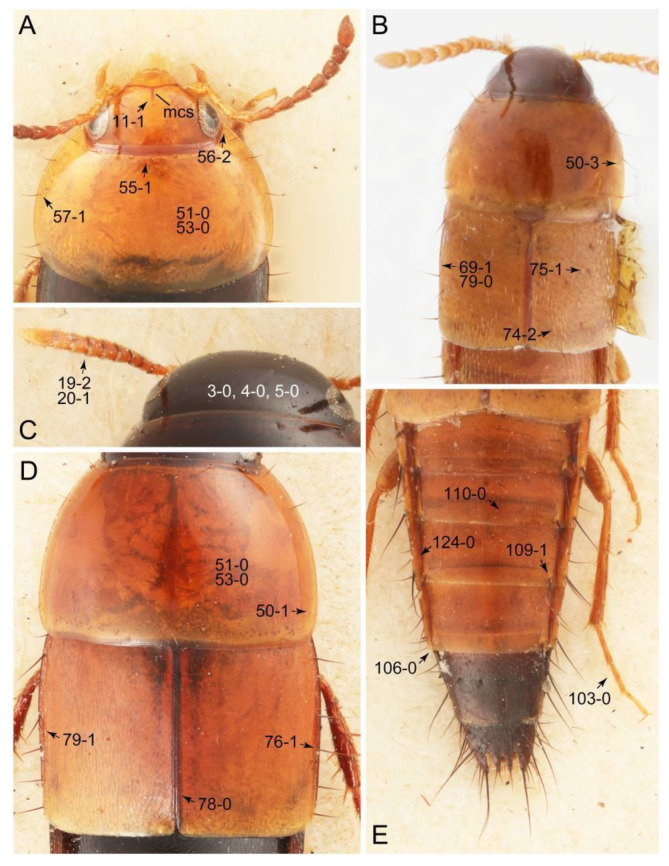
Photographs of body parts of Tachyporini: Tachyporina, enlarged. (**A**) head and pronotum of *Tachyporus obtusus* (Linnaeus), dorsal view. (**B**) forebody of *Palporus nitidulus*, dorsal view. (**C**) head and antenna of *Lamprinus erythropterus*, dorsal view. (**D**) pronotum and elytra of *Lamprinodes saginatus*, dorsal view. (**E**) female abdomen of *Tachyporus obtusus*, dorsal view. Abbreviation: mcs, midcranial suture. Characters and character states (format X-X) are indicated on each figure.

Emended diagnosis: Body usually more or less sub-limuloid (e.g., Figure 6A–D,F, Figures 14, 21, 22, 48, 49 and 57A–C,F), except Deropini (Figure 43) and *Nepaliodes* (Figure 57D,E); head small, not elongate, with midcranial suture in most taxa (e.g., Figure 7A, *mcs*: 11-1), lacking distinct neck constriction; antennal insertion more or less visible from above, located anterior to eye (e.g., Figure 50B: 9-0, 10-0); antennae not extremely slender and verticillate; mandibles without inner teeth (e.g., Figure 9B: 26-0, 27-0); maxillary palpus 4-segmented; labial palpus 3-segmented; ligula large, as long as wide to transverse (e.g., Figures 45B and 53D,E,G: 44-0, 45-1); mesospiracular peritremes well sclerotized (e.g., Figures 18A and 27E, *msp*: 67-2); elytron with epipleural keel, lacking longitudinally raised sutural edge (e.g., Figures 51A,C and 52C: 78-0, 80-0); procoxa large, prominent (e.g., Figures 12A, 24C and 29A: 96-3); metacoxae with developed ventral lamellae both lateral and posterior margins in mesial inner areas (e.g., Figures 12B, 60E and Supplementary Figure S2B, *vlmtc*: 100-3); tarsi 5-5-5; abdomen with six visible sternites; intersegmental membranes without ‘brick-wall’ pattern (Figure 12C: 107-0); tergite VIII and sternite VIII usually with sexual dimorphisms, frequently modified into lobes (e.g., Figures 12E, 19C, 31, 32, 47B and 61B,C: 116-1, 117-3, 135-3, 135-5, 137-3); tergite IX in male without ventral struts, basally ventrally fused (e.g., Figure 61E: 119-1), except Deropini; male aedeagus with simple and glabrous parameres very closely appressed to median lobe, parameres not widely separated from each other (e.g., Figures 12F, 20B,C, 33E–H and 56A–D: 142-1, 143-1, 143-2, 143-3); gonocoxites with small gonocoxite II and large gonostylus (e.g., Figures 20D and 56E: 154-0, 156-2), bearing curved setae (e.g., Figures 47D and 56E: 155-1). Modified after Newton et al. [16].

**Figure 8:**
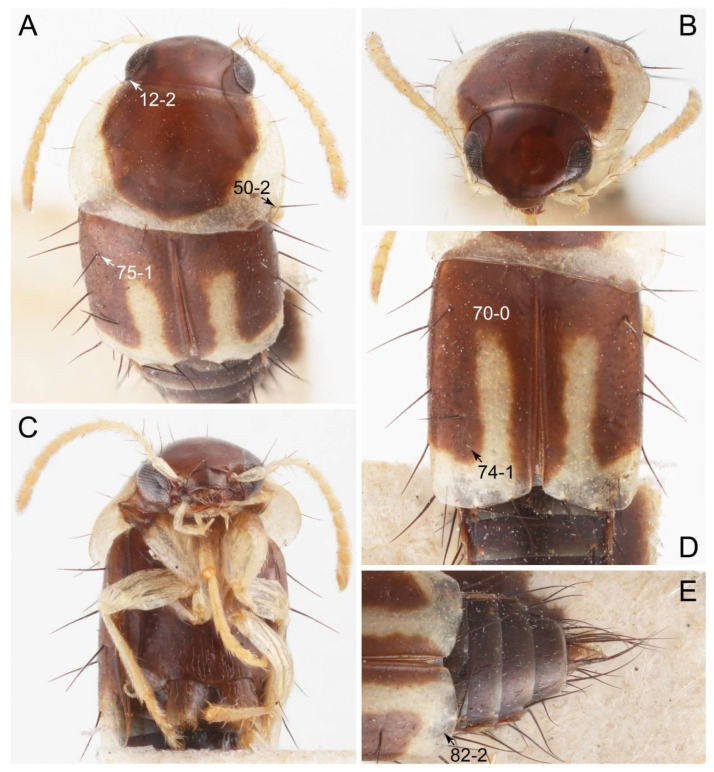
Photographs of body parts of Tachyporini: Tachyporina, enlarged (*Symmixus sikkimensis*, holotype). (**A**) forebody, dorsal view. (**B**) head and pronotum, frontal view. (**C**) forebody, ventral view. (**D**) elytra, dorsal view. (**E**) apical half of elytra and abdomen, dorsal view. Characters and character states (format X-X) are indicated on the selected figures.

**Figure 9:**
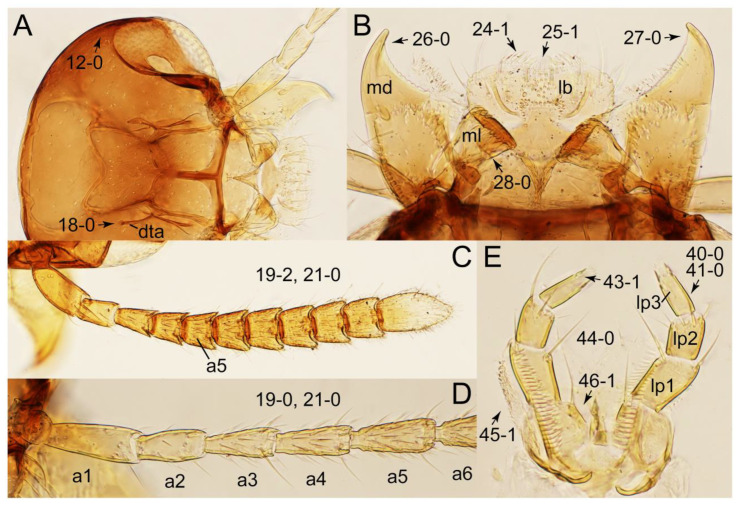
Photographs of body parts of Tachyporini: Tachyporina, enlarged. (**A**) head of *Tachyporus jocosus* Say, dorsal view. (**B**) mandibles and labrum (epipharynx) of *Tachyporus jocosus*, ventral view. (**C**) left antenna of *Lamprinus erythropterus*, dorsal view. (**D**) basal half of left antenna of *Tachyporus jocosus*, dorsal view. (**E**) labium of *Lamprinodes saginatus*, ventral view. Abbreviations: a1–6, antennomere 1–6; dta, dorsal tentorial arm; lb, labrum; lp1–3, labial palpomere 1–3; md, mandible; ml, molar lobe. Characters and character states (format X-X) are indicated on each figure.

**Figure 10:**
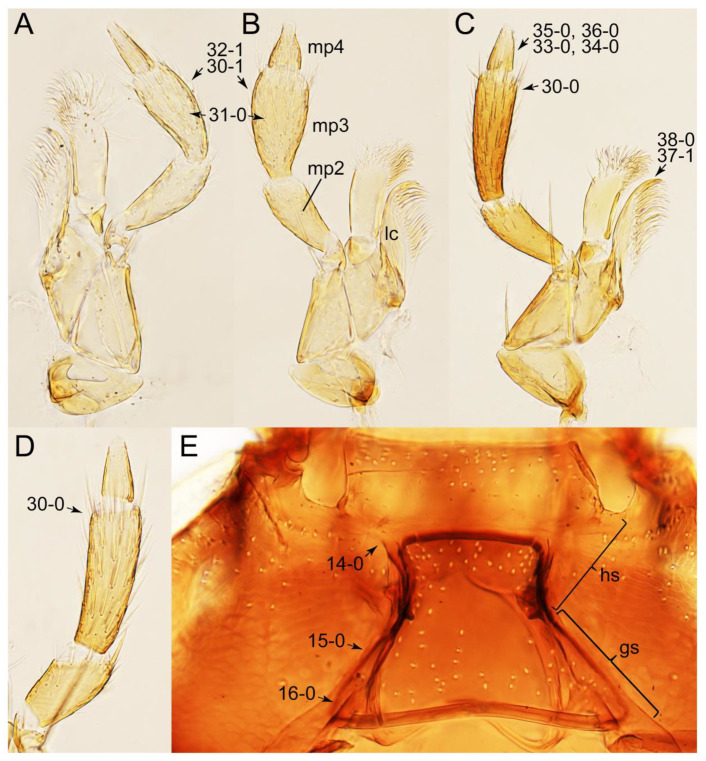
Photographs of body parts of Tachyporini: Tachyporina, enlarged. (**A**) left maxilla of *Tachyporus jocosus*, ventral view. (**B**) right maxilla of *Palporus nitidulus*, ventral view. (**C**) right maxilla of *Lamprinodes saginatus*, ventral view. (**D**) left maxilla of *Lamprinus erythropterus*, ventral view. (**E**) hypostomal and gular sutures of *Tachyporus jocosus*, ventral view. Abbreviations: gs, gular suture; hs, hypostomal suture; lc, lacinia; mp2–4, maxillary palpomere 2–4. Characters and character states (format X-X) are indicated on each figure.

**Figure 11:**
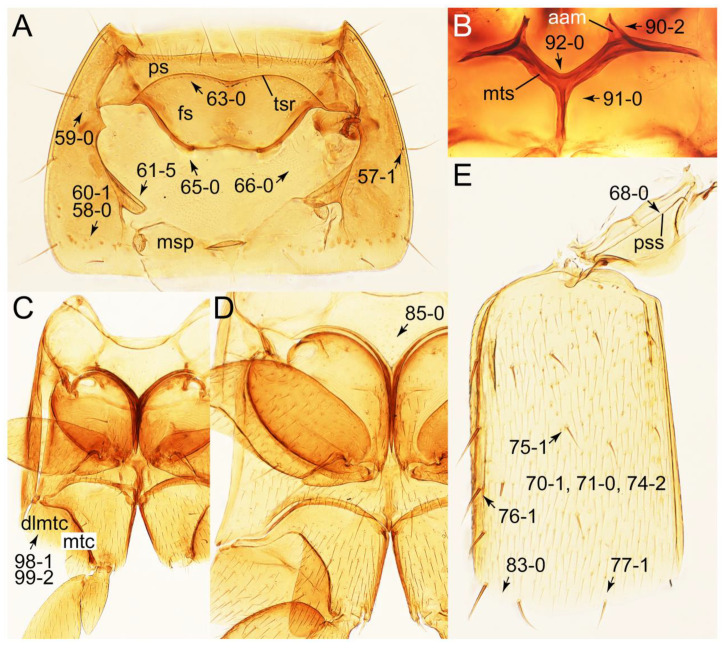
Photographs of body parts of Tachyporini: Tachyporina, enlarged. (**A**) prosternum and pronotum of *Palporus nitidulus*, ventral view. (**B**) metendosternite of *Tachyporus jocosus*, dorsal view. (**C**) mesothorax and metathorax of *Palporus nitidulus*, ventral view. (**D**) mesothorax and metathorax of *Lamprinus erythropterus*, ventral view. (**E**) scutellum and left elytron of *Palporus nitidulus*, dorsal view. Abbreviations: aam, anterior arm of metendosternite; dlmtc, dorsal lamella of metacoxa; fs, furcasternum; msp, mesospiracular peritremes; mtc, metacoxa; mts, metendosternite; ps, prosternum; pss, prescutoscutellar suture; tsr, transverse sternacoxal ridge of prosternum. Characters and character states (format X-X) are indicated on each figure.

**Figure 12:**
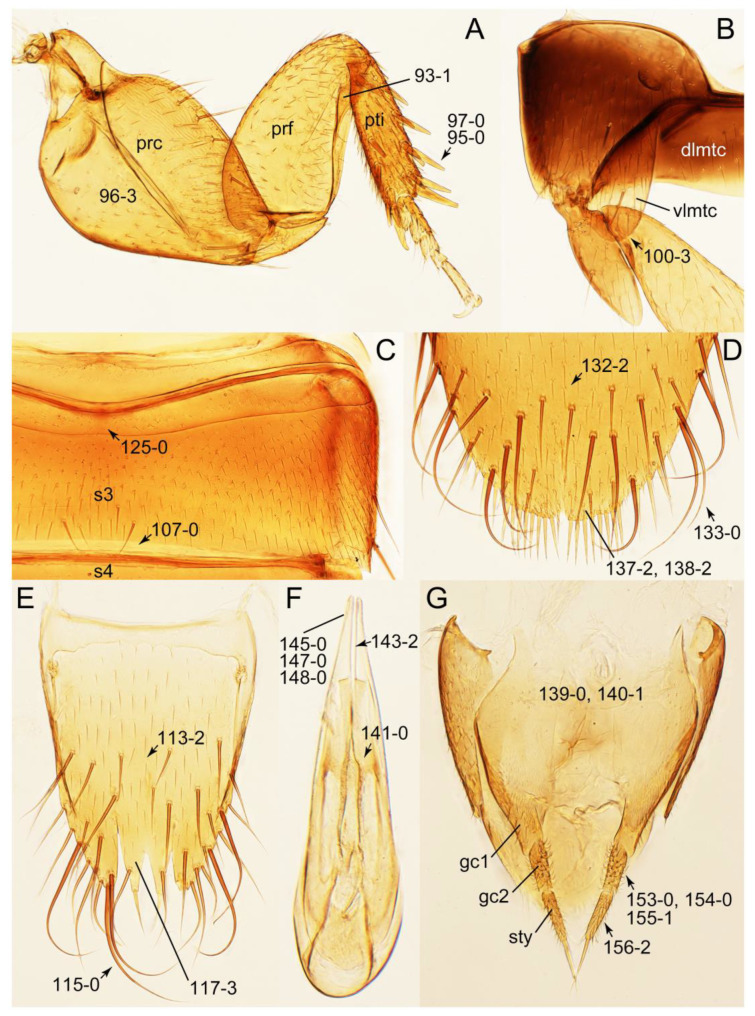
Photographs of body parts of Tachyporini: Tachyporina, enlarged. (**A**) left foreleg of *Lamprinus erythropterus*, ventral view. (**B**) left metacoxa of *Tachyporus chrysomelinus*, ventral view. (**C**) sternite III of *Lamprinodes saginatus*, ventral view. (**D**) female sternite VIII of *Lamprinodes saginatus*, ventral view. (**E**) female tergite VIII of *Lamprinodes saginatus*, dorsal view. (**F**) male aedeagus of *Palporus nitidulus*, dorsal view. (**G**) female genital segments of *Lamprinodes saginatus*, ventral view. Abbreviations: dlmtc, dorsal lamella of metacoxa; gc1–2, gonocoxite 1–2; prc, procoxa; prf, profemur; pti, protibia; s3–4, sternite III–IV; sty, gonostylus; vlmtc, ventral lamella of metacoxa. Characters and character states (format X-X) are indicated on each figure.

**Figure 13:**
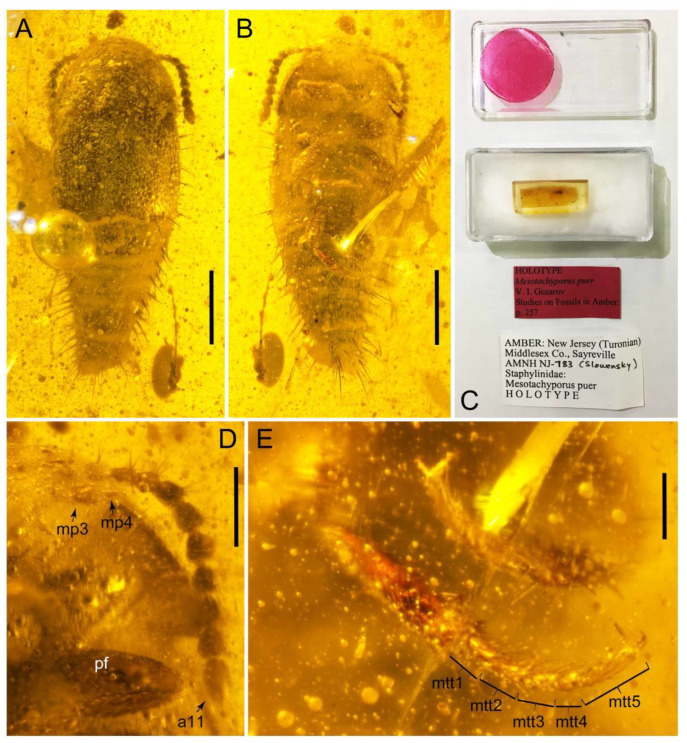
Photographs of Tachyporini: Tachyporina [†*Tachyporus puer* (Gusarov) (= †*Mesotachyporus puer* Gusarov) in Upper Cretaceous (Turonian) New Jersey amber, holotype, AMNH NJ-783]. (**A**) habitus, dorsal view. (**B**) habitus, ventral view. (**C**) specimen with its data labels. (**D**) antenna, maxillary palpus, and foreleg, ventral view. (**E**) metatarsus. Scale bars: 0.5 mm (**A**,**B**); 0.2 mm (**D**); 0.1 mm (**E**). Abbreviations: a11, antennomere 11; mp3–4, maxillary palpomere 3–4; mtt1–5, metatarsomere 1–5; pf, profemur. Photo credits: David Grimaldi (**A**,**B**,**D**,**E**, all used with permission).

**Figure 14:**
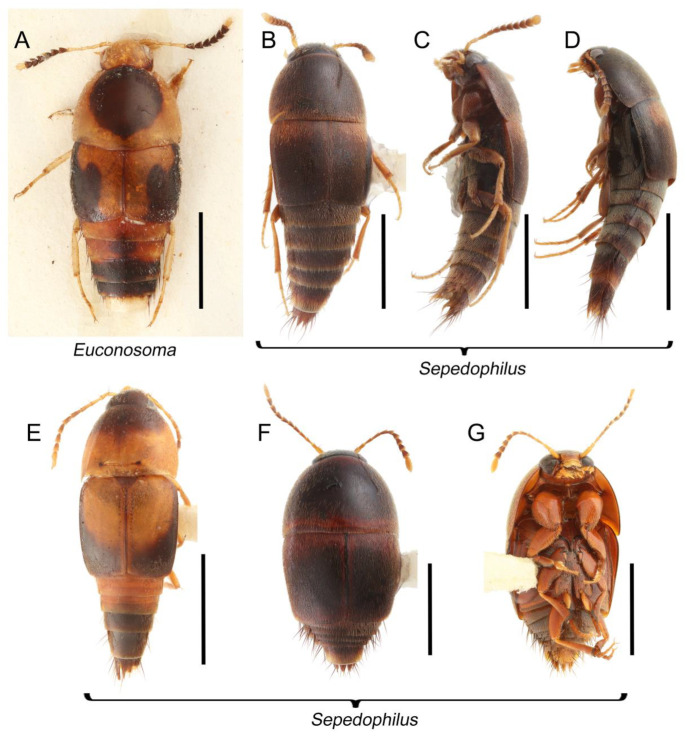
Habitus photographs of Tachyporini: Euconosomatina. (**A**) *Euconosoma picta* (Bernhauer), dorsal view. (**B**) *Sepedophilus cinctulus* (Erichson), dorsal view. (**C**) *Sepedophilus cinctulus* (Erichson), ventrolateral view. (**D**) *Sepedophilus bisignatus* (Horn), dorsolateral view. (**E**) *Sepedophilus littoreus* (Linnaeus), dorsal view. (**F**) *Sepedophilus crassus* (Gravenhorst), dorsal view. (**G**) *Sepedophilus crassus*, ventral view. Scale bars: 2.0 mm (**A**); 1.0 mm (**B**,**C**); 1.5 mm (**D**–**G**).

**Figure 15:**
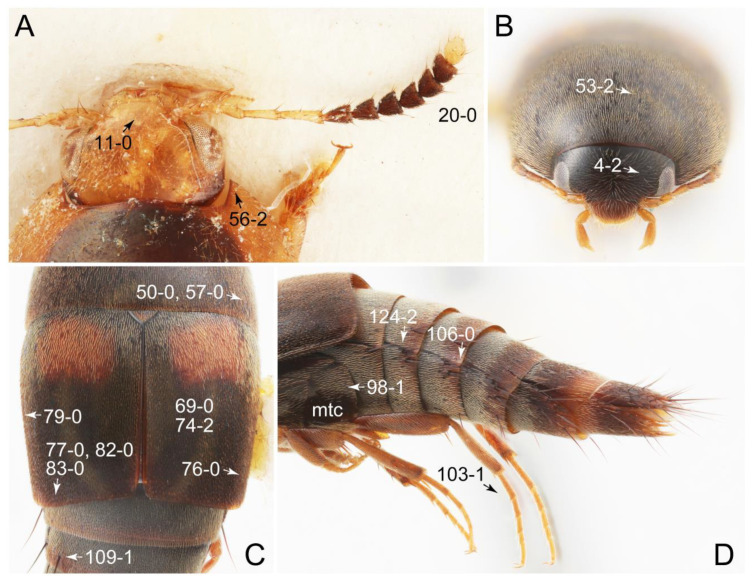
Photographs of body parts of Tachyporini: Euconosomatina, enlarged. (**A**) antenna, head, and pronotum of *Euconosoma picta*, dorsal view. (**B**) head and pronotum of *Sepedophilus bisignatus*, frontal view. (**C**) elytra with pronotal and abdominal bases of *Sepedophilus bisignatus*, dorsal view. (**D**) legs and abdomen of *Sepedophilus bisignatus*, lateral view. Abbreviation: mtc, metacoxa. Characters and character states (format X-X) are indicated on each figure.

**Figure 16:**
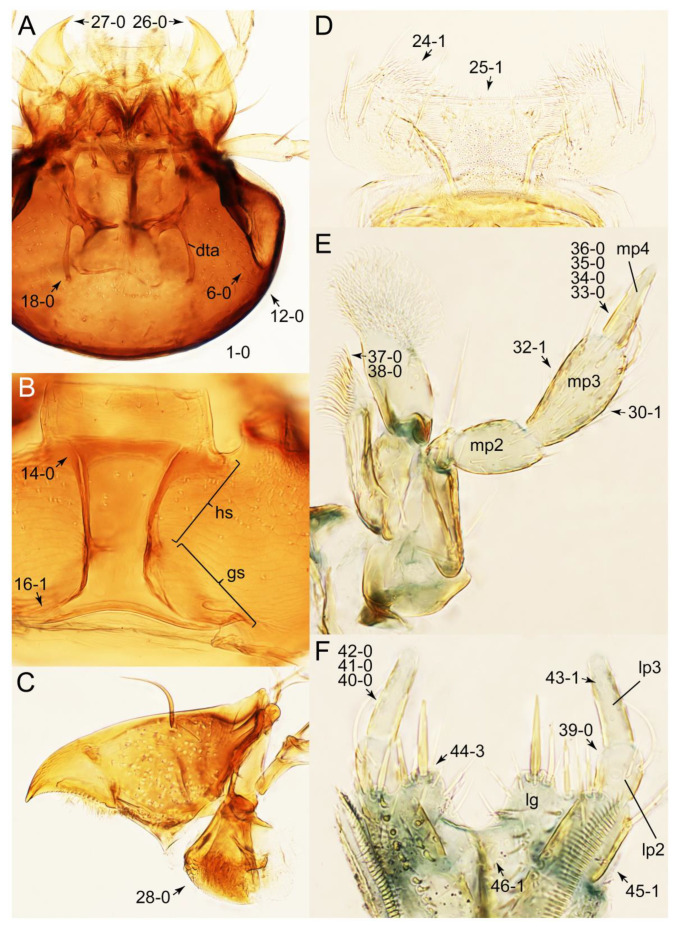
Photographs of body parts of Tachyporini: Euconosomatina, enlarged. (**A**) head of *Sepedophilus littoreus*, dorsal view. (**B**) hypostomal and gular sutures of *Sepedophilus bisignatus*, ventral view. (**C**) right mandible of *Sepedophilus littoreus*, dorsal view. (**D**) labrum of *Sepedophilus crassus*, dorsal view. (**E**) left maxilla of *Sepedophilus scriptus* (Horn), ventral view. (**F**) labium of *Sepedophilus cinctulus*, dorsal view. Abbreviations: dta, dorsal tentorial arm; gs, gular suture; hs, hypostomal suture; lg, ligula; lp2–3, labial palpomere 2–3; mp2–4, maxillary palpomere 2–4. Characters and character states (format X-X) are indicated on each figure.

Description: Body (Figures 6A–D,F, 13A,B, 14, 21, 22, 35C,D, 36C–E, 40C–E, 43, 48, 49 and 57) minute to medium-sized, sub-limuloid, or rarely of a different unique form (*Derops*; Figure 43). Head (e.g., Figure 7A, Figure 8B, Figures 23A, 44A and 50) clearly smaller than pronotum, as long as wide to transverse, usually lacking ocular seta (sensu Campbell [42]) near base of eye on dorsal surface (Figure 16A: 6-0), except some Tachinusini (Figure 50: 6-1); frontal suture and midcranial suture present in most taxa (e.g., Figures 7A and 50, *mcs*: 11-1); tempora or postocular areas without neck constriction (e.g., Figure 9A and Figure 16A: 12-0), but occasionally with neck-like narrowing (e.g., Figures 26A, 45A and 51B,D: 12-1, 12-3); ventral side lacking longitudinal basolateral ridges along eyes (e.g., Figure 26A: 13-0), except in a few examples (e.g., *Leucotachinus, Nepaliodes*; Figures 53A and 58D: 13-1). Dorsal tentorial arms (e.g., Figures 9A and 16A: 18-0) developed, reaching to inner surface of head capsule and forming tentorial pits in most taxa. Hypostomal sutures (e.g., Figure 10E, Figures 16B and 53A: 14-0) fully separated, each curved or angulate. Gular sutures (e.g., Figures 10E, 16B and 53A: 15-0, 16-0, 16-1) widely separated, each rather short, usually more or less curved. Antennal insertion (e.g., Figures 7A, 15A, 26G, 44A and 50) located at, or anterior to, anterior margin of eyes in most taxa (e.g., Figures 44A and 50B: 10-0), usually partially concealed by frontal shelf (e.g., Figure 50B: 8-0, 9-0). Antenna (e.g., Figures 7A–C, 9C,D, 23, 37A and 41B) usually filiform to moniliform (e.g., Figure 15A: 19-0) to clavate or clubbed apically (Figure 23B: 19-3), rarely modified (e.g., Figure 26G: 19-2), not verticillate, frequently with clear pattern of dense and fine recumbent pubescence (e.g., Figures 26D and 59C: 21-1, 21-3). Labrum (e.g., Figures 9B and 53H: 24-1, 25-1) with setose or spinose processes in middle and lateral areas along anterior margin in most cases. Mandible (Figures 9B and 16C) triangular to falciform, lacking subapical inner tooth (Figure 9B: 26-0, 27-0), with developed molar lobe (Figure 16C: 28-0). Maxillary palpus (e.g., Figures 10A–D, 16E, 27A–D, 45D, 53C,F and 59A) 4-segmented, moderately long; palpomere 1 shortest. Labial palpus (e.g., Figures 9E, 16F, 26B,E,F, 45B, 53B,D,E,G and 59B) 3-segmented, inconspicuous, except *Euconosoma*. Ligula (glossae) (e.g., Figures 9E, 45B and 53D,E,G) large, as long as wide to transverse, clearly extending beyond each labial palpus laterally (Figures 9E, 45B and 53E: 45-1). Pronotum (e.g., Figures 7A,B,D, 8A, 11A, 18A, 27E–G, 37B, 41D, 51 and 58A) usually broad, bell-shaped to transverse oval, widened toward base in most cases, but with few exceptions (e.g., *Derops*, *Nepaliodes*; Figures 44A and 58B,C: 49-1, 49-4). Pronotal hypomeron (Figure 11A, Figures 18B, 27F, 54G and 59D: 59-0) without transverse ridge at apical 1/3 to 1/4 (such ridge shown in Figure 69A,B: 59-1). Mesospiracular peritremes (e.g., Figures 18A, 27E–G and 52B, *msp*: 67-2) well sclerotized, except *Leucotachinus*. Scutellum (Figure 11E: 68-0) with prescutoscutellar suture (*pss*) (sensu Blackwelder [30]: Figure 4A,C) lying near base of scutellum. Elytron (e.g., Figures 7B,D, 8D, 11E, 15C, 30, 38A–C, 41E,F, 44C, 51C and 58A,C) short to relatively long, exposing most of abdomen; sutural edge not longitudinally raised (Figures 7D, 24D and 51A, C: 78-0); lateral side with epipleural ridge or keel (e.g., Figure 52C: 80-0), sometimes very strongly folded inward (Figure 30A,B: 81-3). Metendosternite (e.g., Figures 11B, 18D, 28G,I and 54H) Y-shaped, without median process (most, see Figure 28G: 92-0 vs. Figure 28I: 92-1) and posterolateral arms. Legs (e.g., Figures 7E, 12A, 14C,D,G, 15D, 21F, 22C, 28E, 29, 36C–E, 38E–G, 43, 44C, 48E and 52C) with 5-5-5 tarsal formula; protrochantin (e.g., Figure 45E, *prtcn*) well exposed; procoxae (e.g., Figures 12A, 24A–C, 29A, 45E and 52A) prominent, usually enlarged (e.g., Figures 12A, 24C and 29A: 96-3); mesocoxae (e.g., Figures 11C,D, 24A–C, 28A–D, 46A, 52B and 54C–F) narrowly separated in most taxa (Figures 24B, 28C, 46A and 54C,D,F: 87-0), rarely contiguous (some Vatesini and *Leucotachinus*; Figures 52A and 54E: 87-1); metacoxae contiguous (e.g., Figures 11C,D, 52B and 58D), with ventral lamellae in both lateral and posterior margins in mesial inner areas (e.g., Figure 12B, Figures 18E, 28E, 46B, 60E and Supplementary Figure S2B, *vlmtc*: 100-3), except *Vatesus* (Figure 28H, *vlmtc*: 100-0). Abdomen (e.g., Figures 7E, 15D, 25A,C and 44C) gradually to strongly tapering posteriorly, with six visible sterna; paratergites present (most), or absent (Euconosomatina stat. rev., sensu nov.; Figure 15D: 124-2), usually with only a single pair per segment on segments III–VII (Figures 7E and 44C: 124-0), but occasionally with two pairs (some Vatesini sensu nov.; Figure 39F, *pts*); intersegmental membranes without ‘brick-wall’ pattern (Figure 12C: 107-0); tergite VIII and sternite VIII sexually dimorphic, frequently with distinct apical lobes (e.g., Figures 12E, 19C, 31, 32, 39A,B,D,E, 42C,D, 44C, 47B, 55B–D and 61B,C: 116-1, 117-2, 117-3, 135-2, 135-3, 135-4, 135-5); female sternite VIII with row of sensory setae along lobes or in alternative positions on posterior margin (Figures 12D, 19D, 32A,B, 47E, 55D and 61C: 138-2, 138-3, 138-4). Male genital segments (abdominal segments IX and X): tergite IX (e.g., Figures 20A, 33A–D, 39C, 42E, 47F, 55E–G and 61E) dorsally contiguous or separated in basal half, ventrally basally fused except in *Derops* (see [23,43]), without ventral struts (see Figures 33D and 61E: 119-1). Male genitalia: aedeagus (Figures 12F, 20B,C, 33E–H, 47F, 56A–D and 61F) with parameres very closely appressed to median lobe (e.g., Figures 33E,G, 56C and 61F: 142-1); each paramere simple, the two not widely separated from each other (e.g., Figures 12F, 20B,C, 33F,H, 47F, 56A,B,D and 61F: 143-1, 143-2, 143-3), without parameral setae (e.g., Figure 12F: 147-0); internal sac usually without prominent spines (e.g., Figure 56A: 141-0), except *Tachinoproporus* (Figure 42B, *dsp*). Female genital segments (Figures 12G, 20E, 34, 39H, 47C and 61D: 139-0) usually transverse to only weakly elongate; basal area not usually well sclerotized (Figures 20E and 61D: 140-1). Female genitalia: gonocoxites (e.g., Figure 12G, 20E, 34, 39H, 47D, 56E and 61D) present, well-developed; gonocoxite II shorter than gonostylus in most taxa (e.g., Figures 20D, 47C,D: 154-0), usually covered with curved setae (e.g., Figures 20D, 34A,C, 47C,D and 56E: 155-1, 155-2); gonostylus (e.g., Figure 12G, 34, 47C,D and 56E: 156-2) large, conspicuous.

Composition: Four tribes of 36 genera (7 extinct), with 1194 species (18 extinct). See Table A1 for overview and distributions. See also Appendix A.

- Tribe Tachyporini MacLeay, 1825 [40] sensu nov.
  - Subtribe Tachyporina MacLeay, 1825 [40] stat. nov., sensu nov.
  - Subtribe Euconosomatina Cameron, 1918 [44] stat. rev., sensu nov.
- Tribe Vatesini Seevers, 1958 [13] sensu nov.
- Tribe Deropini Smetana, 1983 [23]
- Tribe Tachinusini Fleming, 1821 [45] stat. rev., sensu nov.

Fossils: Fossil records of Tachyporinae sensu nov. from both the Cenozoic and Mesozoic are relatively prevalent and even abundant, found mainly from the various Holarctic deposits ([46,47]; see details in each of the taxonomic category below). †*Leehermania prorova* Chatzimanolis et al., 2012, the Upper Triassic fossil species from the Cow Branch Formation of southern Virginia, USA, was described as the oldest Staphylinidae and the earliest representative of Polyphaga [48]. In the original description, it was suggested that there is a possible affinity with Tachyporinae, or its close relatives [48]. Contrary to doubts of the systematic position of the fossil outside of Staphylinidae [2], Chatzimanolis [47] again recognized †*Leehermania* as the oldest described staphylinoid beetle. Nevertheless, †*Leehermania* was recently moved to an extinct lineage within the small beetle suborder Myxophaga, closest to the modern family Hydroscaphidae, based on re-examination of the type material and rigorous phylogenetic analyses [49]. As there are no staphylinid fossils found from the Triassic deposits, the most reliable oldest fossil records of Tachyporinae sensu nov. date back to the Middle to Upper Jurassic based on compression fossils found in China, Kazakhstan, and Australia [49,50,51,52]. Most described tachyporine fossils are compressions, whereas only a handful of amber inclusions have been described so far (Table A1 and Table A2).

Notes on family-group names: The present study on Tachyporinae (in the current sense) resurrects a little-used family-group name based on *Tachinus*. The taxonomic treatment (Opinion 1743) is made by The International Commission on Zoological Nomenclature (ICZN) [53]. This opinion established the family-group name priority for names based on *Tachyporus* over those based on *Tachinus* and emended the stem of the family-group names based on *Tachinus* to *Tachinus*- instead of *Tachin*- because of the (senior) homonymy of the latter with the extremely widely used family name Tachinidae in Diptera (based on *Tachina* Meigen) [53]. It also added names to the Official Lists of names in zoology and designated type species that are relevant in some cases [53].

Remarks: A series of significant changes are made for the new definition of Tachyporinae sensu nov. and its tribal divisions (Figure 5). After my phylogenetic analyses and observations, the revised classification contains the four tribes as listed there. This means that the originally included tribe Mycetoporini is now excluded from Tachyporinae, and is raised to subfamily rank (see below and Discussion). Therefore, the former classification comprised of five tribes is rejected here. Although I could not examine most of the tachyporine fossils, they are putatively assigned to the newly defined tribes based on available information. Two extant tachyporine genera, namely *Tachinoporus* and *Tachinoproporus*, were unambiguously placed in Tachyporinae sensu nov. on the basis of my direct examination of the type material.

The revised classification also sheds light on their ecology and microhabitats. Although there is significant lack of ecological information for the tachyporine taxa used here, I speculate a possible ecological trend for Tachyporinae sensu nov. First, semi-aquatic taxa are found only in Deropini and Tachinusini stat. rev., sensu nov. (clade E in Figures 3 and 4), and at least some members seem to be adapted such environments. Second, social parasitism is only found in Tachyporini sensu nov. (myrmecophilous only: *Lamprinodes* Luze and *Lamprinus* Heer) and Vatesini sensu nov. (myrmecophilous: *Vatesus*; termitophilous: *Termitoplus* Silvestri). A possible termitophile, *Sepedophilus nigeriensis* (Silvestri, 1947), comb. nov. (= ‘*Urolitus*’ *nigeriensis* Silvestri, 1947 [54] (see Euconosomatina stat. rev., sensu nov. section below)), was probably incidentally collected from a termite nest, lacking any adaptive morphological features on the body based on the original description [54]. All other tachyporine genera have putatively free-living lifestyles.

#### 3.2.2. Tribe Tachyporini MacLeay, 1825 sensu nov.

Tachyporini MacLeay, 1825: 49 [40] (Figures 1C, 2–20 and Figure S1; Table A1)

Type genus: *Tachyporus* Gravenhorst, 1802 [41]: 124.

Differential diagnosis: Tachyporini sensu nov. differs from all other tribes of Tachyporinae sensu nov. based on the following combination of characters: body pubescent with long and thin setae (at least elytra and abdomen), head without neck-like narrowing, antenna basally lacking clear borderline of dense and fine recumbent setae, maxillary palpomere 3 setose, longer and narrower than penultimate palpomere, and abdomen dorsally with blackish macrosetae.

**Figure 17:**
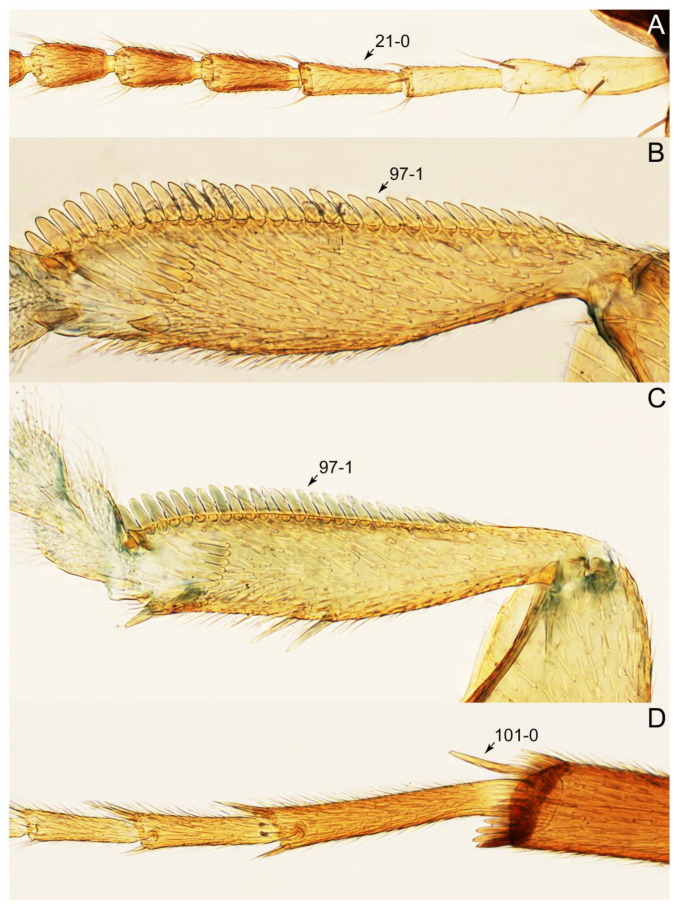
Photographs of body parts of Tachyporini: Euconosomatina, enlarged, dorsal view. (**A**) antenna of *Sepedophilus littoreus*. (**B**) protibia of *Sepedophilus bisignatus*. (**C**) protibia of *Sepedophilus cinctulus*. (**D**) metatarsus and metatibial spines of *Sepedophilus crassus*. Characters and character states (format X-X) are indicated on each figure.

**Figure 18:**
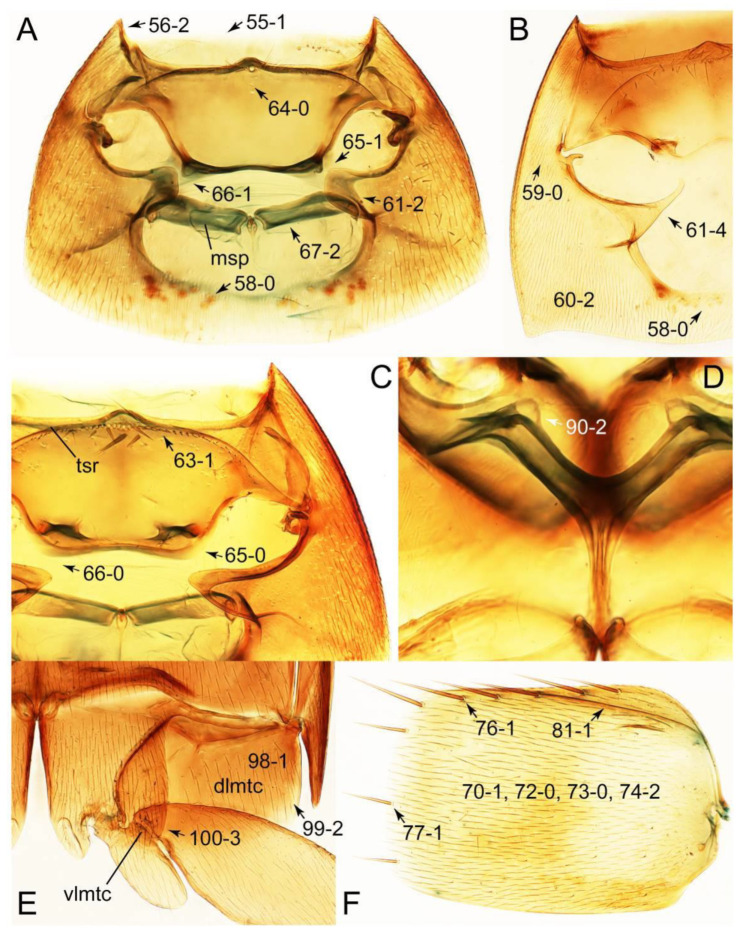
Photographs of body parts of Tachyporini: Euconosomatina, enlarged. (**A**) prosternum and pronotum of *Sepedophilus scriptus*, ventral view. (**B**), prosternum and pronotum of *Sepedophilus littoreus*, ventral view. (**C**) prosternum and pronotum of *Sepedophilus cinctulus*, ventral view. (D) metendosternite of *Sepedophilus scriptus*, dorsal view. (**E**) left metacoxa of *Sepedophilus bisignatus*, ventral view. (**F**) left elytron of *Sepedophilus scriptus*, dorsal view. Abbreviations: dlmtc, dorsal lamella of metacoxa; msp, mesospiracular peritremes; tsr, transverse sternacoxal ridge of prosternum; vlmtc, ventral lamella of metacoxa. Characters and character states (format X-X) are indicated on each figure.

**Figure 19:**
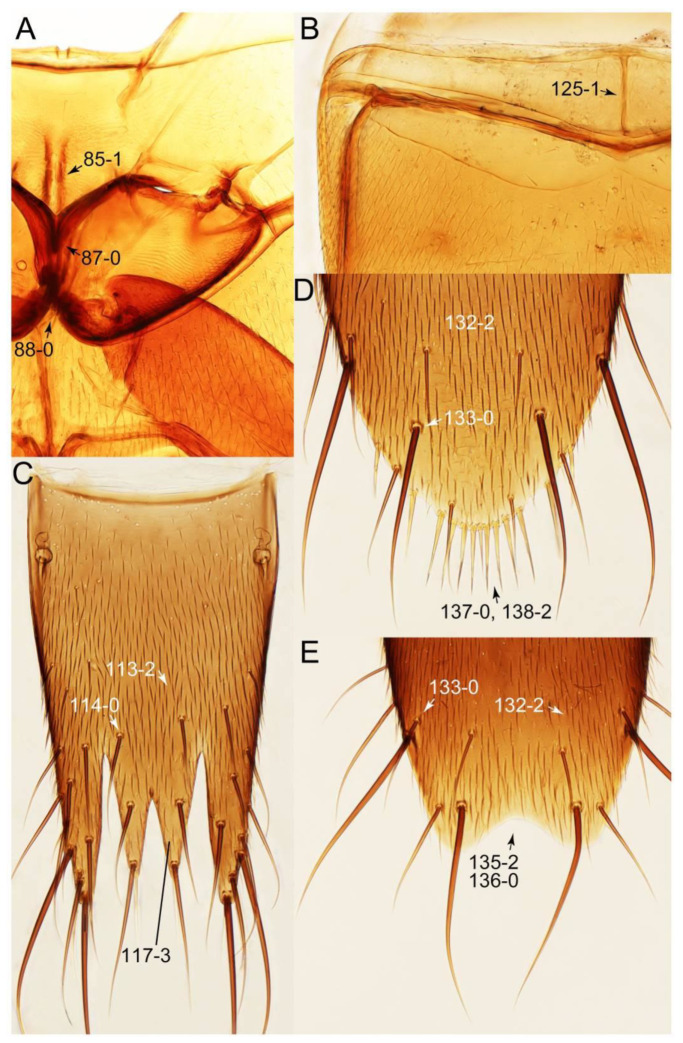
Photographs of body parts of Tachyporini: Euconosomatina, enlarged. (**A**) mesoventrite and metaventrite of *Sepedophilus crassus*, ventral view. (**B**) sternite III of *Sepedophilus littoreus*, ventral view. (**C**) female tergite VIII of *Sepedophilus littoreus*, dorsal view. (**D**) female sternite VIII of *Sepedophilus littoreus*, ventral view. (**E**) male sternite VIII of *Sepedophilus littoreus*, ventral view. Characters and character states (format X-X) are indicated on each figure.

**Figure 20:**
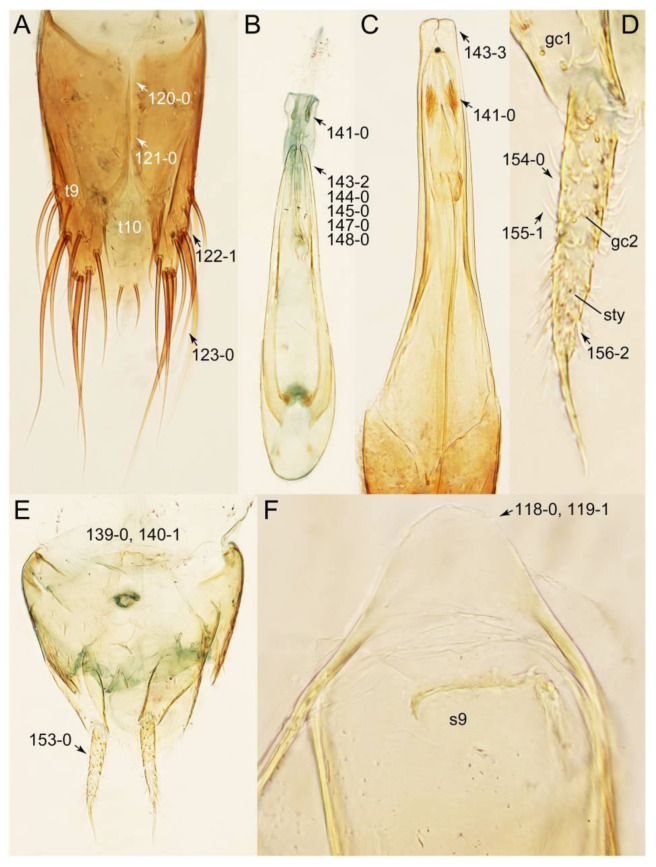
Photographs of body parts of Tachyporini: Euconosomatina, enlarged. (**A**) male tergites IX–X of *Sepedophilus cinctulus*, dorsal view. (**B**) male aedeagus of *Sepedophilus cinctulus*, ventral view. (**C**) male aedeagus of *Sepedophilus littoreus*, ventral view. (**D**) female left gonocoxites and gonostylus of *Sepedophilus cinctulus*, dorsal view. (**E**) female genital segments of *Sepedophilus cinctulus*, ventral view. (**F**) male tergite IX and sternite X of *Sepedophilus crassus*, dorsal view. Abbreviations: gc1–2, gonocoxite 1–2; s9, sternite IX; sty, gonostylus; t9–10, tergite IX–X. Characters and character states (format X-X) are indicated on each figure.

Emended diagnosis. Body surface pubescent with long and thin setae (e.g., Figure 15B–D: 53-2, 74-2) (Tachyporina stat. nov., sensu nov. usually lacking setae on head and pronotum, but pubescent on elytra and abdomen; Figures 7B and 8D: 74-1, 74-2); head without neck-like narrowing (Figures 9A and 16A: 12-0); antenna without clear pattern (borderline) of dense and fine recumbent pubescence (Figures 9C,D and 17A: 21-0); maxillary palpomere 3 widest around middle (Figures 10A,B and 16E: 30-1) (widest at each apex in *Lamprinus* and *Lamprinodes*; Figure 10C,D: 30-0), not distinctly shorter than palpomere 2 (Figures 10A,B and 16E: 31-0, 32-1), with dense ground setae; maxillary palpomere 4 moderately to strongly pointed, widest at or near base, much shorter and narrower than penultimate palpomere (Figures 10C and 16E: 33-0, 34-0, 35-0); labial palpomere 3 more or less pointed, narrower than penultimate palpomere (Figures 9E and 16F: 40-0, 41-0), except *Euconosoma* Cameron; pronotum and elytra with (e.g., Figures 7A,B,D, 8A and 18F: 57-1, 75-1, 76-1, 77-1) or without (e.g., Figure 15C: 76-0, 77-0) arranged stout setae; abdomen strongly to very strongly tapering posteriorly; tergites III–VI with blackish macrosetae (e.g., Figures 7E, 15C: 109-1), each tergite not strongly transverse, width less than 2.5 times its length; tergite VIII and sternite VIII with more than several macrosetae (Figure 19C–E): 114-0, 133-0); male tergite VIII without modification, that of female with modification (Figures 12E and 19C: 117-3); male tergite IX contiguous dorsally in basal 1/3 (Figure 20A: 120-0), each apex elongate (Figure 20A: 122-1), with numerous macrosetae (Figure 20A: 123-0); male sternite VIII with posterior margin rather widely deeply emarginate medially (Figure 19E: 135-2); female sternite VIII without modification (Figure 19D: 137-0) or with only feebly developed projections (Figure 12D: 137-2).

Description: Body (Figure 6A–D,F, Figure 13A,B and Figure 14) small to relatively large for tachyporines, sub-limuloid to limuloid, with tapered abdomen; surface generally pubescent with long and thin setae, at least on elytra and abdomen (e.g., Figure 7B and Figure 15B,C: 4-2, 53-2, 74-2), but sometimes generally glabrous in head and pronotum (e.g., Figure 7A,C,D: 4-0, 53-0); punctation usually absent or inconspicuous (e.g., Figures 7A,C,D: 3-0, 51-0); color frequently bright, occasionally having markings or patterns on dorsum (e.g., Figures 6D,F and 14A,E). Head (Figures 7A–C, 8A,B, 9A and 15A,B and Figure 16A: 6-0) without ocular seta; postocular areas (e.g., Figures 9A, 16A: 12-0) without neck-like narrowing, but *Symmixus* with slight modification just behind eyes (Figure 8A: 12-2); postgena without minute setae. Antenna (Figures 6A–D,F, 7A–C, 8A–C, 9A,C,D, 13A,B, 14 and 15A and Figure 17A) short to moderate, filiform to fili-moniliform (Figure 9D: 19-0) [weakly modified in *Lamprinodes* (Figure 6A) and myrmecophilous form in *Lamprinus* (Figure 9C: 19-2)], without clear pattern (borderline) of dense and fine recumbent pubescence (e.g., Figures 9C,D and 17A: 21-0). Maxillary palpus (Figures 10A–D, 13D, 16E) somewhat small, palpomeres 2 and 3 usually pubescent (Figures 10A,B and 16E: 32-1); palpomere 3 widest around middle in most taxa (Figures 10A,B and 16E: 30-1) [except *Lamprinodes* (Figure 10C: 30-0) and *Lamprinus* (Figure 10D: 30-0)], not distinctly shorter than palpomere 2 (>0.7×; Figure 10A,B: 31-0); palpomere 4 moderately to strongly pointed, widest at or near base (Figure 10C: 33-0), clearly shorter and narrower than penultimate palpomere (Figures 10C and 16E: 34-0, 35-0). Maxillary lacinia with (Tachyporina stat. nov., sensu nov.) or without (Euconosomatina stat. rev., sensu nov.) unarticulated large, long spine; other numerous teeth and spines long, well-developed (Figures 10C and 16E: 38-0). Labial palpi (Figures 9E and 16F: 46-1) usually rather widely separated at base, slightly narrower, or subequal, to longest labial palpomere; palpomere 3 (Figures 9E and 16F) usually more or less pointed (except in *Euconosoma*, see Cameron [55]: Figure 2), widest at base (Figures 9E and 16F: 40-0), distinctly to moderately narrower than penultimate palpomere (Figures 9E and 16F: 41-0). Mentum strongly transverse, with truncate (Tachyporina stat. nov., sensu nov.) or broadly rounded (Euconosomatina stat. rev., sensu nov.) anterior margin. Pronotum (Figures 7A,B,D and 8A and Figure 18A,B) normal, bell-shaped, usually widest between base and basal 1/5 (Figures 7D and 15C: 50-0, 50-1), with (Tachyporina stat. nov., sensu nov.) or without (Euconosomatina stat. rev., sensu nov.) stout setae on dorsum; surface with (Euconosomatina stat. rev., sensu nov.) or without (most Tachyporina stat. nov., sensu nov.) ground microsetae; anterior margin weakly to moderately concave (Figures 7A and 18A: 55-1), with sharply pointed anterior angles (Figures 7A and 18A: 56-2). Pronotal hypomeron (Figures 8C, 11A, 14G and 18A–C) moderate to wide in basal third (Figures 11A and 18B: 60-1, 60-2), strongly inflexed, not visible in lateral view, with diverse structural patterns of postcoxal processes (Figures 11A and 18A,B: 61-2, 61-4, 61-5). Prosternum with sternacoxal ridge (*tsr*) moderately distant from (Tachyporina stat. nov., sensu nov.), or very close to (Euconosomatina stat. rev., sensu nov.) anterior margin of prosternum in medio-lateral areas, without developed prosternal process (Figure 18A: 64-0). Procoxal cavities open (Figure 11A: 66-0) or closed (Figure 18A: 66-1) posterolaterally (limited members of Euconosomatina stat. rev., sensu nov.). Elytron (Figures 6A–D,F, 7B,D, 8A,D,E, 11E, 13A, 14A–F, 15C and 18F) elongate, usually moderate length, pubescent, with or without macrosetae on dorsum; epipleural gutter along outer margin absent (Euconosomatina stat. rev., sensu nov.) or very narrow (most Tachyporina stat. nov., sensu nov.); lateral side with epipleural ridge or keel, which is moderately folded inward (e.g., Figure 18F: 81-1). Mesoventrite with (Euconosomatina stat. rev., sensu nov., except *Euconosoma*) or without (Tachyporina stat. nov., sensu nov.) longitudinal median carina. Metendosternite (Figures 11B and 18D: 90-2) with anterior arms thick, frequently lamellate. Legs (Figures 6A,F, 7E, 8C, 11C,D, 12A,B, 13E, 14A–D,G, 15A,D, 17B–D and 18E and Figure 19A) moderately long to long (see Figure 14C,D); procoxae (e.g., Figures 8C, 12A and 14C,G) expanded, large (distinctly expanded in *Lamprinus*; Figure 12A: 96-3); tibial apices with (Euconosomatina stat. rev., sensu nov.) or without (Tachyporina stat. nov., sensu nov.) ctenidium of evenly arranged dense equal length spines with much larger apical spur; metacoxae (Figures 11C,D and 18E: 98-1) medium size in each lateral half; metatibiae with short spurs at apex, shorter than 2/5 of metatarsomere 1 (Figure 17D: 101-0), except *Lamprinus*; metatarsus long to markedly long, more than half length of metatibia (Tachyporina stat. nov., sensu nov.), or even longer (Euconosomatina stat. rev., sensu nov.). Abdomen (Figures 6A–D,F, 7E, 8E, 12C, 13A,B 14 and 15D) strongly to very strongly tapering from base to apex, with single pair of paratergites (Tachyporina stat. nov., sensu nov.), or without paratergites (Euconosomatina stat. rev., sensu nov.), on segments IV–VII. Tergites III–VI with macrosetae on posterolateral edges (e.g., Figures 7E, 15C: 109-1), each tergite moderately transverse. Tergite VIII (Figures 12E and 19C: 114-0) with several or more macrosetae; posterior margin in male without modification (116-0), that of female (Figures 12E and 19C: 117-3) with simplified lobes. Male tergite IX (Figure 20A: 119-0, 120-0, 121-0, 122-1, 123-0) fused at base of ventral side, not divided by sternite IX; dorsal side contiguous in basal 1/3 to 2/3 (Figures 20A: 120-0, 121-0), then abruptly divided apically, each with elongate apex (Figure 20A: 122-1); apical areas each with more than several macrosetae, not restricted to apices (Figure 20A: 123-0). Sternite III with (Euconosomatina stat. rev., sensu nov.) or without (Tachyporina stat. nov., sensu nov.) longitudinal median carina. Male sternite VII lacking characteristically arranged peg-like setae. Sternite VIII (Figures 12D and 19D,E: 133-0) with numerous distinct macrosetae; posterior margin in male (Figure 19E: 135-2, 136-0) rather widely deeply emarginate medially without lobes, that of female unmodified (Euconosomatina stat. rev., sensu nov.), or with a pair of feebly developed projections (most Tachyporina stat. nov., sensu nov.), each apex with row of sensory setae along these projections or alternate positions in female (Figures 12D and 19D: 138-2). Male aedeagus (Figures 12F and 20B,C) narrowly elongate, slender, not curved or arcuate in lateral view; parameres in apical part longitudinally contiguous or only weakly separated from each other (Figures 12F and 20B,C: 143-2, 143-3), not widely longitudinally flattened or plate-like (e.g., Figures 12F and 20B: 145-0). Female genitalia (Figures 12G and 20D,E) with gonocoxite II slender, narrowly elongate (Figures 20E: 153-0), bearing only curved setae (Figures 12G and 20D: 155-1).

Composition: Two subtribes, 8 genera (1 extinct), with 510 species (6 extinct). See Table A1 for overview and distributions.

- Subtribe Tachyporina MacLeay, 1825 [40] stat. nov., sensu nov.
- Subtribe Euconosomatina Cameron, 1918 [44] stat. rev., sensu nov.

Remarks: The newly defined tribe Tachyporini sensu nov. contains only the core-members of the tribe, namely the *Tachyporus*-related and *Sepedophilus*-related genera, with a newly established subtribal division as above. This led to the exclusion of many genera previously treated as ‘Tachyporini’, such as *Coproporus* and *Tachinus*. Based on the results of the phylogenetic analyses, Tachyporini sensu nov. forms a sister group to the rest of Tachyporinae sensu nov.

#### 3.2.3. Subtribe Tachyporina MacLeay, 1825 stat. nov., sensu nov.

Tachyporina MacLeay, 1825: 49 [40] (Figures 2–13; Table A1)

Type genus: *Tachyporus* Gravenhorst, 1802 [41]: 124 (= †*Mesotachyporus* Gusarov, 2000 [56]: 256 syn. nov.).

Differential diagnosis: Tachyporina stat. nov., sensu nov. differs from members of Euconosomatina stat. rev., sensu nov. based on the following combination of characters: head and pronotum glabrous (sparsely and inconspicuous, if present), pronotum with arranged macrosetae, protibia without longitudinal row of close-spaced spines along outer margin, metatarsus clearly shorter than whole length of metatibia, and abdomen with single pair of paratergites.

Emended diagnosis: Body rather less convex above in cross section (e.g., Figure 6A,D,F), with usually moderate-width pronotal hypomera (Figure 11A: 60-1); surface somewhat glossy, typically glabrous on head and pronotum (e.g., Figure 7A,C,D: 4-0, 53-0); color usually bright, at least on elytra for many taxa (e.g., Figure 6A,B,D,F); gular sutures not distinctly divergent in basal-most areas (Figure 10E: 16-0); maxillary lacinia with unarticulated large, long spine at apex (e.g., Figure 10C: 37-1); mentum with anterior margin more or less linear; pronotum with characteristically arranged macrosetae (e.g., Figures 7A and 11A: 57-1); prosternum with sternacoxal ridge (*tsr*) moderately distant from anterior margin of prosternum, without prominent modification of furcasternum (Figure 11A: 63-0); protibia without longitudinal row of close-spaced spines along outer margin (e.g., Figure 12A: 97-0); procoxal cavities always open behind (Figure 11A: 66-0); elytron usually with very narrow epipleural gutter along outer margin (Figure 7D: 79-1) (but absent in *Palporus*; Figure 7: 79-0), bearing arranged macrosetae on surface in most taxa (e.g., Figures 7B,D, 8A and 11E: 75-1, 76-1, 77-1); mesoventrite without longitudinal median carina (Figure 11D: 85-0); metatarsus long, more than half length of metatibia, but shorter than whole length of metatibia (e.g., Figure 7E: 103-0); abdomen with a single pair of paratergites on segments III–VII (e.g., Figure 7E: 124-0); sternite III without longitudinal median carina (e.g., Figure 12C: 125-0); female sternite VIII frequently with a pair of feebly developed projections along posterior margin (Figure 12D: 137-2); sclerotized spermatheca absent.

Composition: Five genera, with 144 species (4 extinct). See Table A1 for overview and distributions.

- *Lamprinodes* Luze, 1901 [57]: 181.
  Type species: *Tachyporus saginatus* Gravenhorst, 1806 [58]: 6.
- *Lamprinus* Heer, 1839 [59]: 286.
  Type species: *Lamprinus lasserrei* Heer, 1839 [59]: 286 (= *Oxyporus erythropterus* Panzer, 1796 [60]: pl. 21).
- *Palporus* Campbell, 1979 [61]: 11 stat. nov.
  Type species: *Staphylinus nitidulus* Fabricius, 1781 [62]: 337.
- *Symmixus* Bernhauer, 1915 [63]: 56.
  Type species: *Symmixus sikkimensis* Bernhauer, 1915 [63]: 57.
- *Tachyporus* Gravenhorst, 1802 [41] sensu nov. (= †*Mesotachyporus* Gusarov, 2000 [56]: 256 syn. nov.).
  Type species: *Staphylinus chrysomelinus* Linnaeus, 1758 [38]: 423.

Fossils: Similar to the abovementioned situation of *Sepedophilus*, the fossils of *Tachyporus* sensu nov. are also diverse and abundant in mid-Eocene Baltic amber ([64,65]; Yamamoto, pers. obs.), but with only a single described species, *Tachyporus bicoloratus* Paśnik, 2005 [66]. The only extinct genus †*Mesotachyporus* has been known from the Mesozoic [56], but it is here synonymized under *Tachyporus* (see discussion below). From the Cenozoic, †*Tachyporus annosus* Herman, 2001 [67] (= †*T. nigripennis* Scudder, 1900) [68] is known from the Eocene Florissant deposit in Colorado, USA [21]. Additionally, there is a *Tachyporus* fossil found from early Middle Miocene Dominican amber [69], but it has not yet been described.

Remarks. This newly defined subtribe contains the type genus of Tachyporinae sensu. nov., namely *Tachyporus* sensu nov. Compared to the subtribe Euconosomatina stat. rev., sensu nov., the members of Tachyporina stat. nov., sensu nov. share the conserved body plan including the presence of paratergites, and all species lack the comb-like spines on the protibiae.

The tachyporine monobasic tribe Symmixini Bernhauer, 1915 [61] had been recognized until rather recently [21], but this tribe was synonymized under Tachyporini by Schülke [22]. In this study, I have examined the type genus *Symmixus*, specifically the holotype of the type species (Figures 6D,E and 8), and found that it is indeed very close to *Tachyporus* sensu nov. in many aspects, although *Symmixus* has the characteristically modified metatarsomere 3 [22]. In light of this conclusion and the phylogenetic results, the synonymy of Symmixini is maintained here and I have placed it in this subtribe.

#### 3.2.4. Genus *Tachyporus* Gravenhorst, 1802 sensu nov.

*Tachyporus* Gravenhorst, 1802: 124 [41] (Figures 2–4, 6F, 7A,E, 9A,B,D, 10A,E, 11B, 13 and Figure S1B,D; Table A1)

= †*Mesotachyporus* Gusarov, 2000 [56]: 256 syn. nov.

Type species: *Staphylinus chrysomelinus* Linnaeus, 1758 [38]: 423.

Diagnosis: See Campbell [61], as subgenus *Tachyporus*.

Remarks: Previously, the genus *Tachyporus* included two subgenera, *Tachyporus* sensu str. and *Palporus* Campbell [21,61,70]. In this study, I have recognized only *Tachyporus* sensu str. as members of the newly defined genus *Tachyporus* sensu. nov. *Palporus* is raised to a distinct genus based on observation of morphological characters and their phylogenetic analyses (see below).

The extinct monotypic genus †*Mesotachyporus* from Upper Cretaceous (Turonian) New Jersey amber can undoubtedly be assigned to this subtribe on the basis of general morphological similarity with *Tachyporus* in the traditional sense, namely the overall body shape, short and slender maxillary palpomere 4, antennal pubescence, general shape of the pronotum, and chaetotaxy of the elytra (Figure 13A,B,D,E; [56]; see also a habitus photograph in Grimaldi et al. [71]: Figure 8A). During the course of my study, I re-examined the holotype of †*Mesotachyporus puer* Gusarov, 2000 [56] to unveil the generic identity and to extract more characters for the genus and species. As a result, the only notable difference between †*Mesotachyporus* and *Tachyporus* is the presence of markedly shortened first tarsomeres in †*Mesotachyporus*, compared to recent *Tachyporus* species, as already mentioned in the original description [56]. However, this character alone is not enough to justify the generic validity. Similarly, a fossil species of megalopsidiine rove beetle in mid-Cretaceous Kachin amber (the Albian–Cenomanian boundary or older) from northern Myanmar, which also has similarly reduced first tarsomeres compared with the modern congeners, was placed in the extant genus *Megalopinus* Eichelbaum, rather than creating a new genus [72]. Here, †*Mesotachyporus* syn. nov. is not maintained at generic rank, but is synonymized under *Tachyporus* sensu nov. The new combination, †*Tachyporus puer* (Gusarov, 2000) [56] comb. nov., is established here. This fossil species may represent another example of bradytely, showing long-term morphological stasis of an organismal lineage over deep evolutionary time [73]. This phenomenon has frequently been found in beetles, particularly Staphylinoidea, in Kachin amber (e.g., [74,75,76,77]), and Clarke & Chatzimanolis [74] suggested that the continuous presence of mesic habitats over geological time had contributed to such morphological stability. As Chatzimanolis [47] noted, studies assessing the background of this phenomenon based on statistical evidence will be desired. On the other hand, there are some staphylinid taxa known from Kachin amber with unusual morphological features compared to Recent relatives [78,79,80,81]. Further discovery may reveal morphological diversity and evolution in the Mesozoic.

#### 3.2.5. Genus *Palporus* Campbell, 1979 stat nov.

*Palporus* Campbell, 1979: 11 [61] (Figures 3, 4, 6C, 7B, 10B, 11A,C,E, 12F and Supplementary Figure S1A,C; Table A1)

Type species: *Staphylinus nitidulus* Fabricius, 1781 [62]: 337.

Diagnosis: See Campbell [61].

Composition: Two species, as listed below.

1. *nitidulus* (Fabricius, 1781 [62]: 337), comb. nov. (*Staphylinus*). Distribution: Palaearctic, Oriental, and Afrotropical Regions; intro. Nearctic and Australian Regions.
  * See Herman [21] and Schülke & Smetana [70] for synonymic information.
2. *neomexicanus* (Campbell, 1979 [61]: 14), comb. nov. (*Tachyporus*). Distribution: USA (New Mexico, Colorado).

Remarks: The subgenus *Palporus* Campbell was established for the two *Tachyporus* species in a study of the North American *Tachyporus* [61]. Since the original description, no additional species have been included in *Palporus*. This subgenus was characterized by the following combination of characters [61]: body small (less than 1.4 mm), slender; elytra narrower, parallel-sided, and; maxillary palpus with palpomere 4 short and thick, etc.

As a result of the phylogenetic analyses (Figures 3 and 4), *Palporus*, i.e., *Palporus nitidulus* comb. nov. (= *Tachyporus nitidulus*; Figures 6C, 7B, 10B, 11A,E,C 12F, and Supplementary Figure S1A,C), was separated from the *Tachyporus* clade, which is comprised of three species of *Tachyporus* sensu str., including the type species of *Tachyporus*, namely *Tachyporus chrysomelinus* (Figures 6F and 12B). The internal generic relationship within Tachyporina stat. nov., sensu nov. was not statistically supported by the lower bootstrap values (Figure 3), resulting in weak evidence for the generic status of *Palporus*. However, several morphological evidences probably justify this new generic validity.

As mentioned above, *Palporus* can be separated from *Tachyporus* sensu str. by the general body form in having smaller and more slender body [61], with more or less parallel-sided elytra which widest in middle (Figure 7B: 69-1). In Tachyporina stat. nov., sensu nov., the typical members have basally widened pronotum, with the maximum width situated at, or near, the base in most taxa (except *Symmixus*). In contrast, *Palporus* species have distinct pronotum in comparison with three genera (*Tachyporus*, *Lamprinus*, and *Lamprinodes*), with the maximum width located between the basal 1/4 and middle of the pronotum (Figure 7B: 50-3). These three genera have posteriorly broadened pronotum widest at or near base (Figure 7D: 50-1), whereas the widest point of the pronotum in *Symmixus* is located between basal 1/5 and basal 1/4 (Figure 8A: 50-2). Moreover, *Symmixus* can be discriminated from *Palporus* by having more elongate elytron (ratio of length/width: >1.8×; Figures 6D and 8D: 70-0), and *Palporus* has shorter and less elongate elytron (length/width: 1.3–1.8×; Figures 6C and 7B: 70-1). Another distinct feature is the structures of the maxillary palpi (Figures 10B and Supplementary Figure S1A). The maxillary palpomere 3 of *Palporus* is very thick and strongly expanded, but the remaining other Tachyporina stat. nov., sensu nov. genera do not have such structure. In *Lamprinus* and *Lamprinodes*, the maxillary palpomere 3 is gradually widened apically and widest at (or near) the apex (Figures 10C, D: 30-0). On the other hand, *Tachyporus* and *Symmixus* has weakly expanded maxillary palpomere 3, with the widest point located around middle of the palpomere (Figures 10A and Supplementary Figure S1B: 30-1). The density of ground setation on maxillary palpomere 3 is also clearly different. In *Palporus*, it is much more densely covered with setae than in the other genera. The maxillary palpomere 4 is also distinctive in *Palporus*; it is very short and thick, clearly broader than that of the rest of Tachyporina stat. nov., sensu nov. (Figure 10B and Supplementary Figure S1B). Furthermore, this palpomere is unusual in *Palporus* as it is abruptly narrower in apical third (Supplementary Figure S1B, arrow), and it is prominently densely pubescent ([61]: Figure 84) (see also Figure S1A). I found another characteristic feature in the female genitalia of *Palporus*. The gonostylus in *Palporus* is much longer than gonocoxite II (Supplementary Figure S1C), but it is only slightly to moderately longer, or even shorter, than gonocoxite II in the other members of Tachyporina stat. nov., sensu nov. (Figure 12G and Supplementary Figure S1D). Finally, *Palporus* is the unique taxon which completely lacks epipleural gutter along the outer margin of elytron in Tachyporina stat. nov., sensu nov. (Figure 7B: 79-0).

In light of morphological characters and their phylogenetic analyses, these subgenera should be considered as separate genera, i.e., *Tachyporus* sensu nov. and *Palporus* stat. nov.

#### 3.2.6. Subtribe Euconosomatina Cameron, 1918 stat. rev., sensu nov.

Euconosomatina Cameron, 1918: 216 [44] (Figures 1C, 2–5 and 14–20; Table A1)

Type genus: *Euconosoma* Cameron, 1918 [44]: 215.

Differential diagnosis: Euconosomatina stat. rev., sensu nov. differs from members of Tachyporina stat. nov., sensu nov. based on the following combination of characters: head and pronotum densely pubescent with silky fine setae, pronotum without arranged macrosetae, protibia with longitudinal row of close-spaced spines along outer margin, metatarsus very long, much longer than whole length of metatibia, and abdomen without paratergites.

Emended diagnosis: Body rather strongly convex above in cross section (e.g., Figures 14D,F and 15B), with wide pronotal hypomera (Figure 18B: 60-2); surface less glossy, uniformly densely pubescent including head and pronotum with silky fine setae (e.g., Figure 15B–D: 4-2, 53-2, 74-2); color usually black to dark reddish brown (Figure 14), sometimes with colorful spots or maculations (Figures 14A,E and 15C); gular sutures strongly divergent sides, nearly transverse, in basal 1/4 (Figure 16B: 16-1); maxillary lacinia without unarticulated large, long spine at apex (e.g., Figure 16E: 37-0); labium with ligula sometimes with medial premental lobes and large, peg-like setae (Figure 16F: 44-3); mentum with anterior margin broadly rounded (Figure 16B: 47-2); pronotum widest at base, densely pubescent, lacking arranged stout setae (e.g., Figure 15B,C: 50-0, 53-2, 57-0); prosternum with sternacoxal ridge (*tsr*) very close to anterior margin of prosternum (Figure 18C: 63-1), with frequently modified furcasternum (Figure 18A: 65-1); protibia with longitudinal row of close-spaced spines along outer margin (Figure 17B,C: 97-1), except †*Palaeosepedophilus* [82]; protarsi with basal three tarsomeres expanded (Figures 15A and 17C); meso- and metatibial apices bordered by ctenidium of evenly arranged dense equal length spines with much larger apical spur (Figures 15D, 17D); procoxal cavities occasionally closed behind (e.g., Figure 18A: 66-1); elytron without epipleural gutter along outer margin (Figure 15C: 79-0), lacking arranged stout setae in most taxa as in Figure 15C, D (except the *Sepedophilus scriptus* species group; Figure 18F: 76-1, 77-1); mesoventrite with longitudinal median carina (Figure 19A: 85-1), except *Euconosoma*; metatarsus very long, much longer than whole length of metatibia (e.g., Figure 15D: 103-1); abdomen without paratergites, with only sutures (Figure 15D: 124-2); sternite III with longitudinal median carina (Figure 19B: 125-1); female sternite VIII with posterior margin unmodified, lacking a pair of feebly developed projections (Figure 19D: 137-0); spermatheca sclerotized, simple.

Composition: Three genera (1 extinct), with 366 species (2 extinct). See Table A1 for overview and distributions.

- *Euconosoma* Cameron, 1918 [44]: 215.
  Type species: *Euconosoma elegans* Cameron, 1918 [44]: 216 (= *Conosoma pictum* Bernhauer, 1903 [83]: 25).
- *Sepedophilus* Gistel, 1856 [84]: 386 (= *Urolitus* Silvestri, 1947 [54]: 147 syn. nov.).
  Type species: *Staphylinus pubescens* Paykull, 1790 [85]: 138 (= *Staphylinus littoreus* Linnaeus, 1758 [38]: 422).
- †*Palaeosepedophilus* Paśnik & Kubisz, 2002 [82]: 357.
  Type species: †*Palaeosepedophilus succinicus* Paśnik & Kubisz, 2002 [82]: 357.

Fossils: It is noteworthy that the fossils of *Sepedophilus* in mid-Eocene Baltic amber are very diverse and abundant (Yamamoto, pers. obs.), although only one species, *Sepedophilus balticus*, has been formally described from this deposit [82]. Additionally, I have seen a single *Sepedophilus* fossil in early Middle Miocene Dominican amber deposited in the FMNH. As a newly defined subtribe Euconosomatina stat. rev., sensu nov. includes one extinct genus, †*Palaeosepedophilus* from Baltic amber [82], which can be placed in Euconosomatina stat. rev., sensu nov. by the general characters including the absence of the paratergites [82]. However, †*Palaeosepedophilus* is morphologically very distinct in lacking the rows of spines along the outer margin of protibiae and has ventro-dorsally compressed, axe-shaped terminal maxillary palpomeres [82].

Notes on family-group name: The name Euconosomini had been considered incorrect original stem formation, therefore I herein adopt the tribe-level name Euconosomatini Cameron, 1918 [44] established on the genus *Euconosoma* Cameron, 1918 [44] (stem: Euconosomat-) [20]. The family-group name Euconosomatini (= Euconosomini) originally contained a single genus *Euconosoma* only [44], but I also included *Sepedophilus* and †*Palaeosepedophilus* in Euconosomatina stat. rev., sensu nov. based on general similarities including the lack of abdominal paratergites, presence of a row of comb-like protibial spines (not in †*Palaeosepedophilus* [82]), and densely pubescent body. The name Sepedophilini Ádám, 2001 [86] is a subjective junior synonym of this name that never came into common use (Sepedophilini was synonymized under Tachyporini by Bouchard et al. [20]).

Remarks: In the present study, three genera of Tachyporini sensu nov. were grouped together to form a newly resurrected and defined subtribe Euconosomatina stat. rev., sensu nov. It can be easily recognized by the above-mentioned characters, especially the presence of the longitudinal row of close-spaced spines along the outer margin of the protibia (Figure 17B,C). Another remarkable feature in this subtribe is the absence of the abdominal paratergites (Figures 14D and 15D). Differing from most Osoriinae (except Eleusinini), the Staphylininae genus *Coomania* Cameron [87], or the Paederinae subtribe Procirrina (Pinophilini) have the same kind of fusion, the abdominal tergites and sternites are not fused together, i.e., ring-like, but are separated by a narrow suture along each lateral side.

The enigmatic monotypic genus *Urolitus* shares the same basic body plan with *Sepedophilus*, including the spines along outer margins of the protibiae and the absence of paratergites [54]. Other characters such as mouthparts and sternite VIII are nearly fully congruent with this genus. Therefore, *Urolitus* syn. nov. should be a junior synonym of *Sepedophilus* based on morphological similarity and the results of my phylogenetic analyses. Following this treatment, a new combination of the type species is provided: *Sepedophilus nigeriensis* (Silvestri, 1947) [54] comb. nov. Based on the presence of a row of macrosetae along the outer margin of the elytron [54], it may be close to the *scriptus* or similar undescribed species group (sensu Campbell [88]).

Campbell [88] divided *Sepedophilus* into 12 species groups based primarily on the North American species. Known larvae of these species groups have been the focus of interesting evolutionary research on feeding habits and strategies, as the larval types are generally associated with species groups and are also relevant to predaceous and mycophagous feeding habits [17,18]. Since the presence of so many undescribed *Sepedophilus* species and species groups have been suggested (e.g., [89,90,91]), more new species will undoubtedly be discovered in the future.

#### 3.2.7. Tribe Vatesini Seevers, 1958 sensu nov.

Vatesini Seevers, 1958: 183 [13] (Figures 1A,B, 2–5 and Figure 21, Figure 22, Figure 23, Figure 24, Figure 25, Figure 26, Figure 27, Figure 28, Figure 29, Figure 30, Figure 31, Figure 32, Figure 33, Figure 34, Figure 35, Figure 36, Figure 37, Figure 38, Figure 39, Figure 40, Figure 41 and Figure 42; Table A1)

Type genus: *Vatesus* Sharp, 1876 [92]: 201.

Differential diagnosis: Vatesini sensu nov. differs from all other tribes of Tachyporinae sensu nov. based on the following combination of characters: forebody usually glabrous and strongly glossy, antenna short in most taxa, usually shorter than pronotal width, devoid of dense and fine recumbent pubescence in basal three or four antennomeres in most taxa, maxillary palpomere 4 longer than penultimate palpomere, pronotal hypomeron with very short postcoxal process, elytra usually shorter and less elongate, with strongly to distinctly folded elytral epipleuron, and abdomen rather strongly to very strongly tapering posteriorly, lacking blackish macrosetae dorsally.

Emended diagnosis: Forebody with surface glabrous (e.g., Figures 22A,B and 24D: 53-0, 74-0), rarely with inconspicuous microsetae, very strongly glossy (matte in *Tachinoproporus*; Figure 41D,E); head with (Figure 26A: 12-1) or without (Figure 23A: 12-0) very weak neck-like narrowing behind eyes; antenna short in most taxa, usually shorter than pronotal width (e.g., Figure 23B: 20-1), devoid of dense and fine recumbent pubescence in basal two or four antennomeres (Figure 26D: 21-1); maxillary palpomere 4 more or less pointed, widest at or near base, longer and narrower than penultimate palpomere (Figure 27A,C,D: 33-0, 34-2, 35-0) (shorter or subequal in *Vatesus* and *Mimocyptus*; Figure 27C: 34-0); pronotal hypomeron with weakly developed postcoxal process (e.g., Figure 27E: 61-1); elytra short in most taxa, with blade-like lateral and posterior margins (Figure 30: 70-2, 82-1); elytral epipleuron strongly to distinctly sharply inflexed (Figure 30A,B: 81-3); procoxa markedly large, strongly expanded (e.g., Figures 24B,C and 29A: 96-3); abdomen rather strongly to very strongly tapering posteriorly, lacking blackish macrosetae dorsally (except abdominal terminalia), except *Tachinoporus* and *Vatesus berghoffae* Kistner, 2006, which have scattered erect macrosetae on the abdominal tergites; abdominal segments III–VII with one (Figure 25A: 124-0) or two pairs of paratergites (Figure 39F, *pts*); abdominal segments IV–VI frequently with somewhat produced posterolateral margins (Figures 25A,B and 28F: 106-1); tergite VIII in both sexes usually with lobe-like modifications (Figure 31: 116-1, 117-3); male tergite IX abruptly divergent and separated dorsally in basal 1/3 in most taxa (Figure 33A: 120-1), each apex usually forming single lobe (Figure 33B,C: 122-2), usually with only single to several macrosetae (Figure 33C: 123-2), except few genera; male sternite VIII usually widely emarginate with developed lobes (Figure 32E: 135-4), that of female with longer lobes, bearing two or three sensory setae at apex (Figure 32A,B: 137-3, 138-3); male aedeagus usually slender, arcuate in lateral view in most taxa (Figure 33E–G); gonocoxite II triangular or narrowly elongate, with both normal and curved setae (Figure 34A,C: 153-1, 153-2, 155-2).

**Figure 21:**
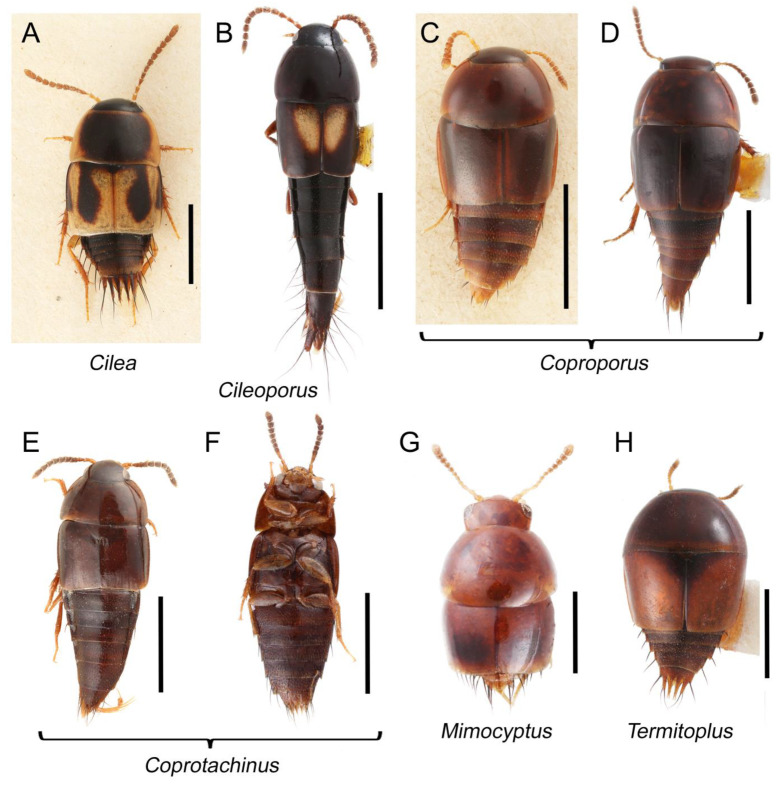
Habitus photographs of Vatesini. (**A**) *Cilea silphoides*, dorsal view. (**B**) *Cileoporus fenestratus* (Sharp), dorsal view. (**C**) *Coproporus colchicus*, dorsal view. (**D**) *Coproporus laevis* LeConte, dorsal view. (**E**) *Coprotachinus habrocerinus* (Eppelsheim), dorsal view. (**F**) *Coprotachinus habrocerinus*, ventral view. (**G**) *Mimocyptus* sp., dorsal view. (**H**) *Termitoplus grandis*, dorsal view. Scale bars: 1.5 mm (A,B); 1.0 mm (**C**–**F**,**H**); 0.5 mm (**G**).

**Figure 22:**
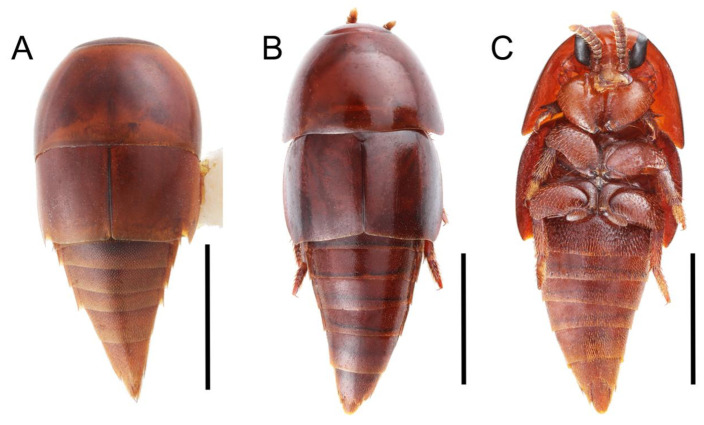
Habitus photographs of Vatesini (*Vatesus*). (**A**) *Vatesus praedatorius* Seevers, dorsal view. (**B**) *Vatesus gigas* (Wasmann), dorsal view. (**C**) *Vatesus gigas*, ventral view. Scale bars: 1.5 mm (**A**); 2.0 mm (**B**,**C**).

**Figure 23:**
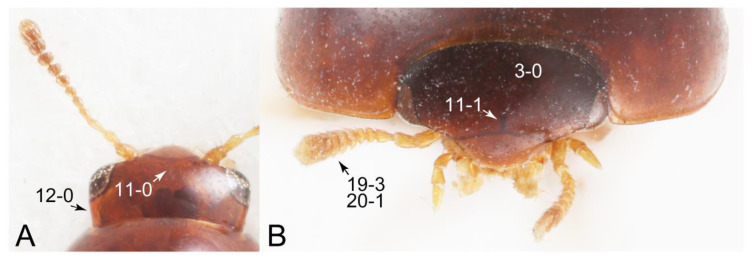
Photographs of body parts of Vatesini, head and pronotum, enlarged. (**A**) *Mimocyptus* sp., dorsal view. (**B**) *Termitoplus grandis*, frontal view. Characters and character states (format X-X) are indicated on each figure.

**Figure 24:**
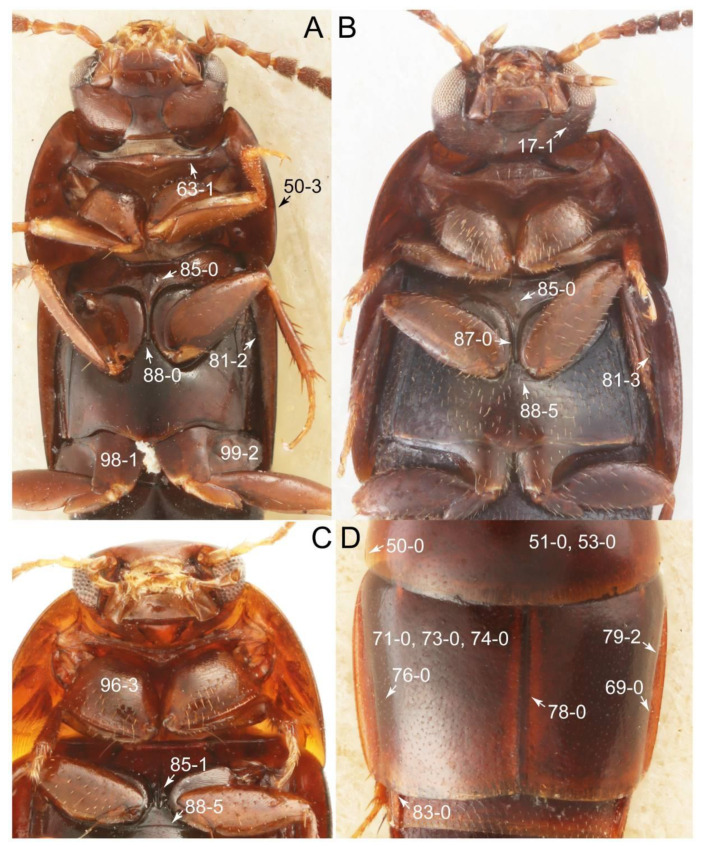
Photographs of body parts of Vatesini, enlarged. (**A**) forebody of *Cileoporus fenestratus*, ventral view. (**B**) forebody of *Coprotachinus habrocerinus*, ventral view. (**C**) pronotum and intermesocoxal processes of *Coproporus laevis*, ventral view. (**D**) elytra of *Coproporus colchicus*, dorsal view. Characters and character states (format X-X) are indicated on each figure.

**Figure 25:**
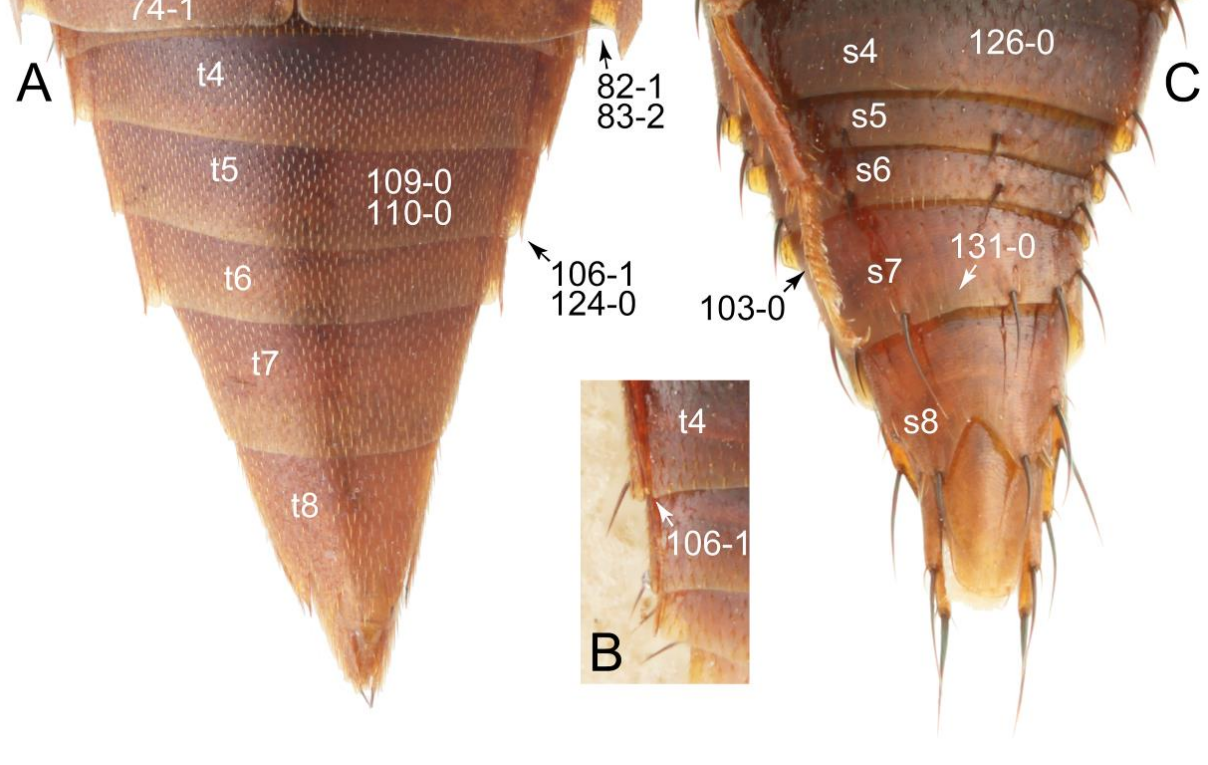
Photographs of body parts of Vatesini, abdomen, enlarged. (**A**) *Vatesus praedatorius*, dorsal view. (**B**) tergite IV and its associated paratergites of *Coproporus colchicus*, dorsal view. (**C**) *Coproporus laevis*, male, ventral view. Abbreviations: s4–8, sternite IV–VIII; t4–8, tergite IV–VIII. Characters and character states (format X-X) are indicated on each figure.

**Figure 26:**
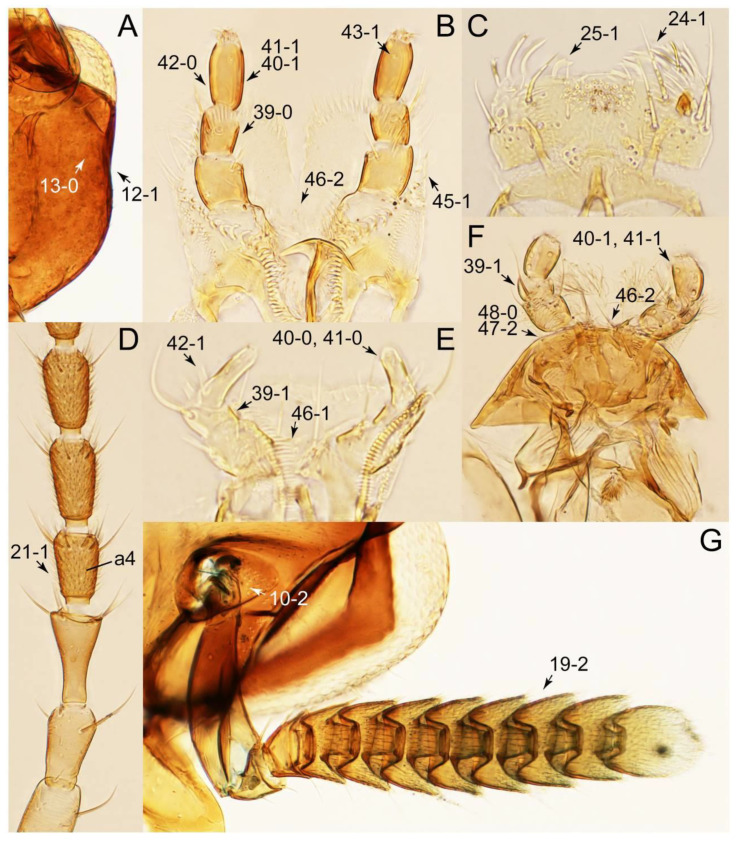
Photographs of body parts of Vatesini, enlarged. (**A**) head of *Coproporus colchicus*, ventral view. (**B**) labium of *Cilea limbifera* (Motschulsky), ventral view. (**C**) labrum of *Coproporus colchicus*, dorsal view. (**D**) basal half of left antenna of *Cilea limbifera*, ventral view. (**E**) labium of *Mimocyptus* sp., ventral view. (**F**) labium and mentum of *Coproporus laevis*, dorsal view. (**G**) antennal insertion and left antenna of *Vatesus praedatorius*, dorsal view. Abbreviation: a4, antennomere 4. Characters and character states (format X-X) are indicated on each figure.

**Figure 27:**
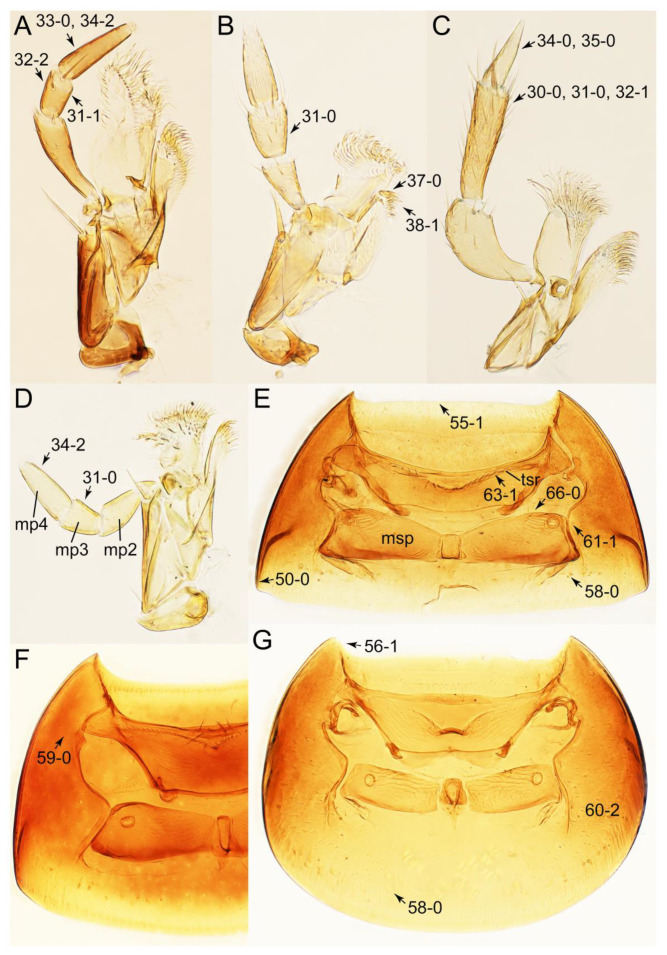
Photographs of body parts of Vatesini, enlarged. (**A**) right maxilla of *Cilea limbifera*, ventral view. (**B**) left maxilla of *Coprotachinus habrocerinus*, dorsal view. (**C**) right maxilla of *Vatesus praedatorius*, ventral view. (**D**) right maxilla of *Termitoplus grandis*, ventral view. (**E**) prosternum and pronotum of *Coproporus colchicus*, ventral view. (**F**) prosternum and pronotum of *Coproporus laevis*, ventral view. (**G**) prosternum and pronotum of *Mimocyptus* sp., ventral view. Abbreviations: mp2–4, maxillary palpomere 2–4; msp, mesospiracular peritremes; tsr, transverse sternacoxal ridge of prosternum. Characters and character states (format X-X) are indicated on each figure.

**Figure 28:**
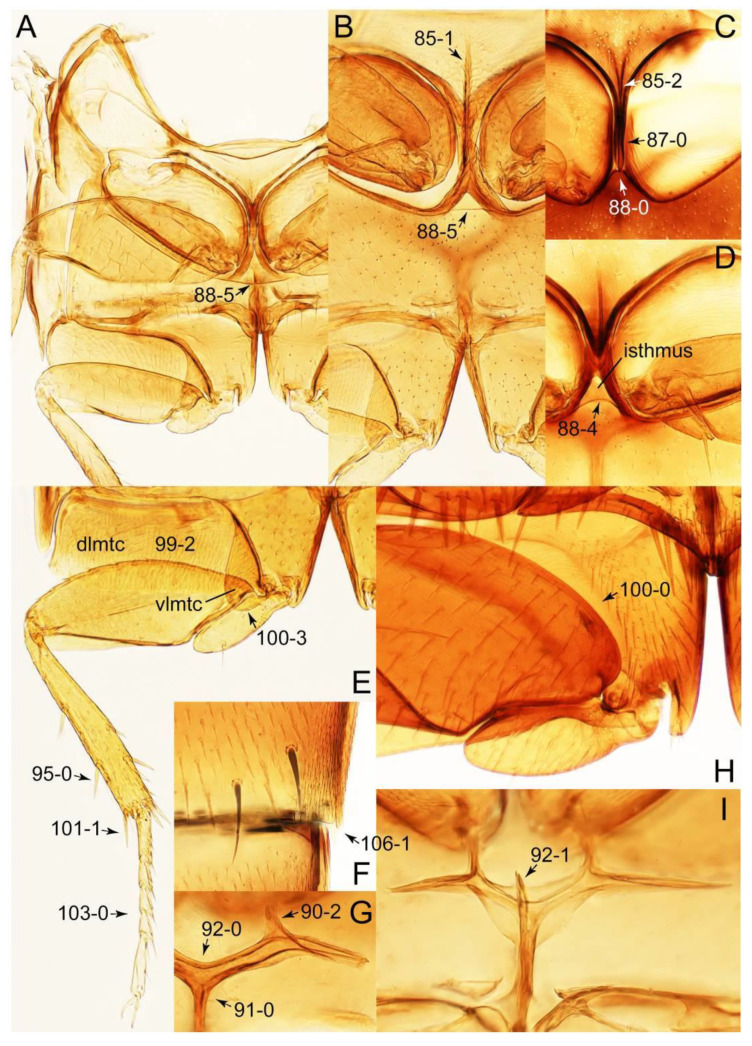
Photographs of body parts of Vatesini, enlarged. (**A**) mesothorax and metathorax of *Mimocyptus* sp., ventral view. (**B**) mesoventrite and metaventrite of *Coproporus colchicus*, ventral view. (**C**) intermesocoxal processes of *Cilea limbifera*, ventral view. (**D**) intermesocoxal processes of *Termitoplus grandis*, ventral view. (**E**) hindleg of *Coproporus colchicus*, ventral view. (**F**) posterior margin of sternite V of *Vatesus praedatorius*, ventral view. (**G**) metendosternite of *Coproporus colchicus*, dorsal view. (**H**) right metacoxa of *Vatesus gigas*, ventral view. (**I**), metendosternite of *Coprotachinus habrocerinus*, dorsal view. Abbreviations: dlmtc, dorsal lamella of metacoxa; vlmtc, ventral lamella of metacoxa. Characters and character states (format X-X) are indicated on each figure.

**Figure 29:**
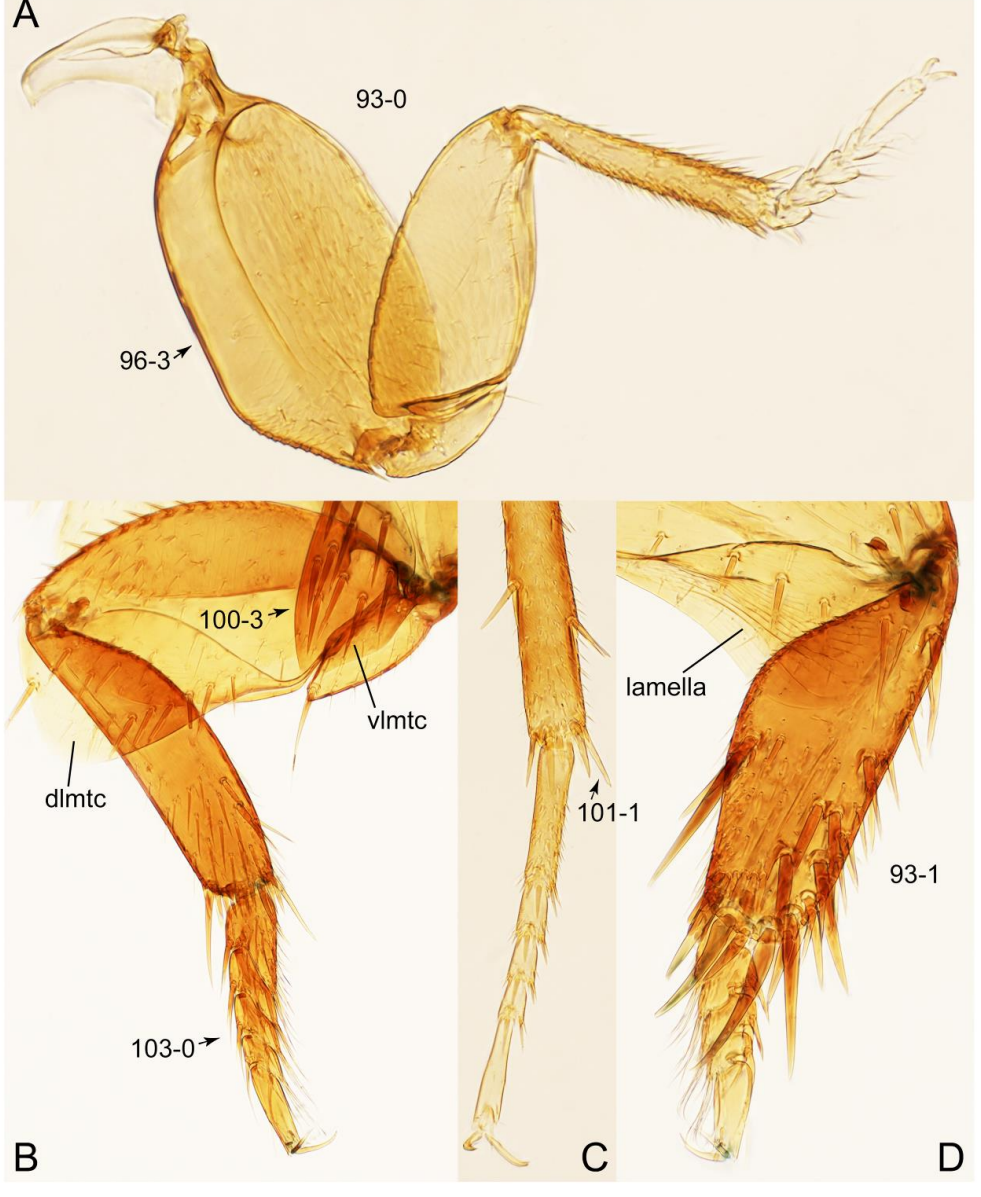
Photographs of body parts of Vatesini, enlarged. (**A**) right foreleg of *Mimocyptus* sp., dorsal view. (**B**) metatibia of *Vatesus praedatorius*, ventral view. (**C**) left metatarsus and metatibial spines of *Cilea limbifera*, ventral view. (**D**) left protibia of *Vatesus praedatorius*, dorsal view. Abbreviations: dlmtc, dorsal lamella of metacoxa; vlmtc, ventral lamella of metacoxa. Characters and character states (format X-X) are indicated on each figure.

**Figure 30:**
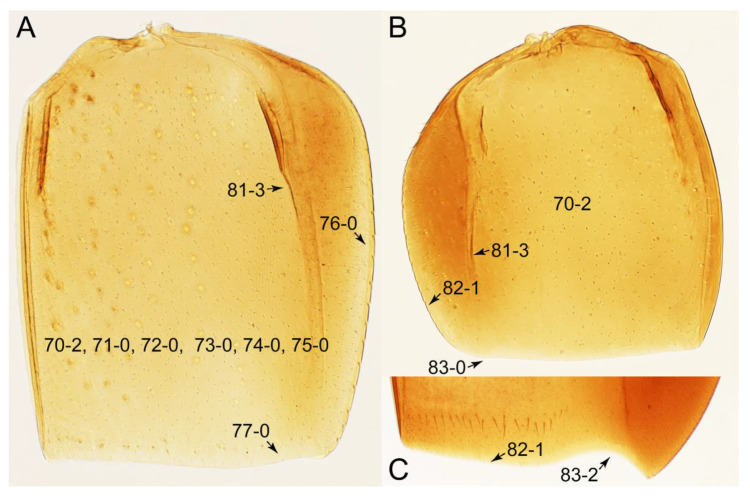
Photographs of body parts of Vatesini, elytra, enlarged. (**A**) left elytron of *Coproporus colchicus*, ventral view. (**B**) left elytron of *Mimocyptus* sp., dorsal view. (**C**) posterior margin of right elytron of *Vatesus gigas*, dorsal view. Characters and character states (format X-X) are indicated on each figure.

**Figure 31:**
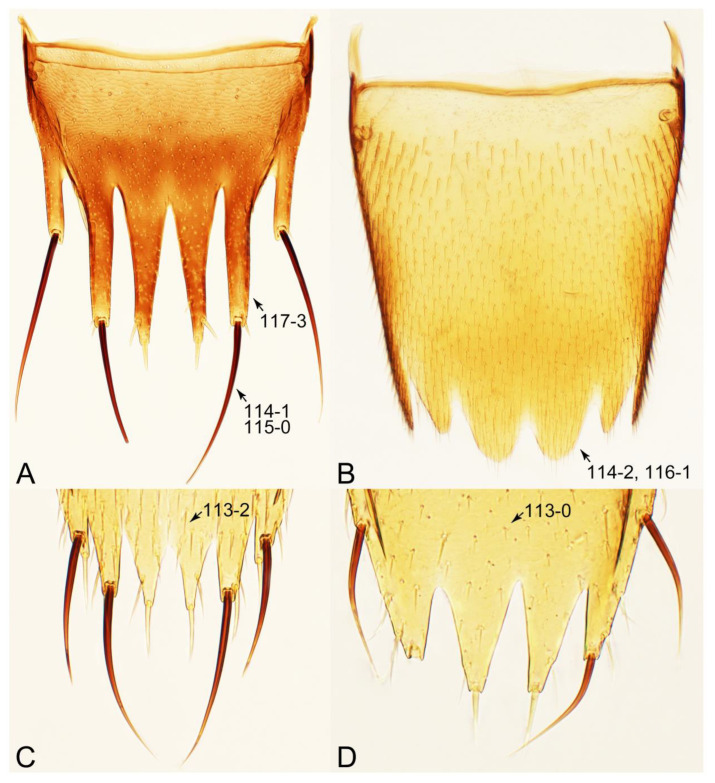
Photographs of body parts of Vatesini, tergite VIII, enlarged. (**A**) *Cilea limbifera*, female, dorsal view. (**B**) *Vatesus gigas*, male, dorsal view. (**C**) *Mimocyptus* sp., female, dorsal view. (**D**) *Coproporus colchicus*, female, dorsal view. Characters and character states (format X-X) are indicated on each figure.

**Figure 32:**
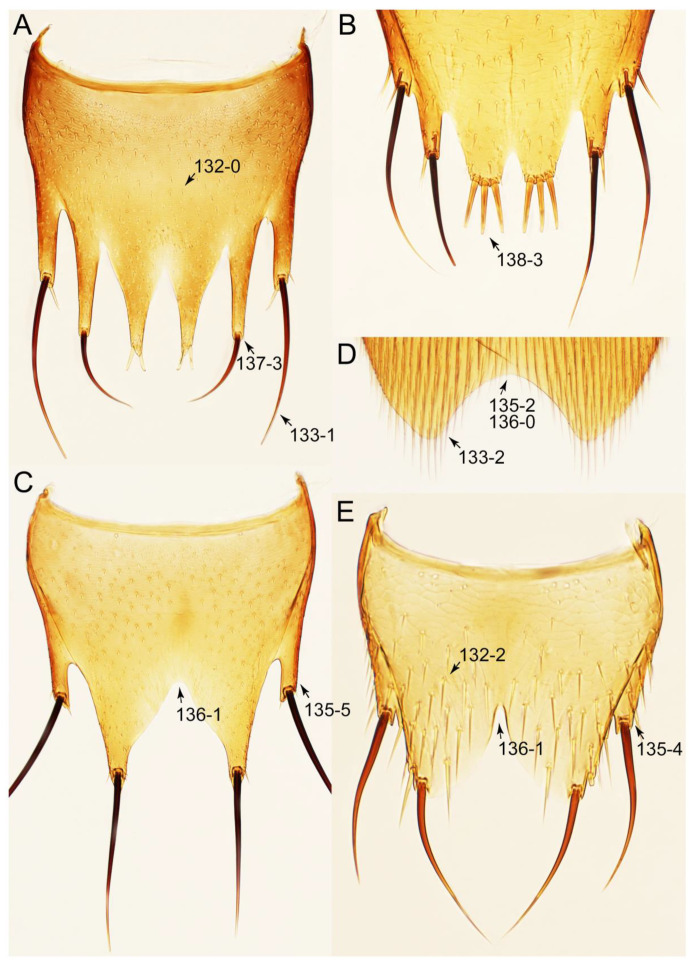
Photographs of body parts of Vatesini, sternite VIII, enlarged. (**A**) *Cilea limbifera*, female, ventral view. (**B**) *Coproporus rutilus* (Erichson), female, ventral view. (**C**) *Vatesus gigas*, male, ventral view. (**D**) *Cilea silphoides*, male, ventral view. (**E**) *Mimocyptus* sp., male, ventral view. Characters and character states (format X-X) are indicated on each figure.

**Figure 33:**
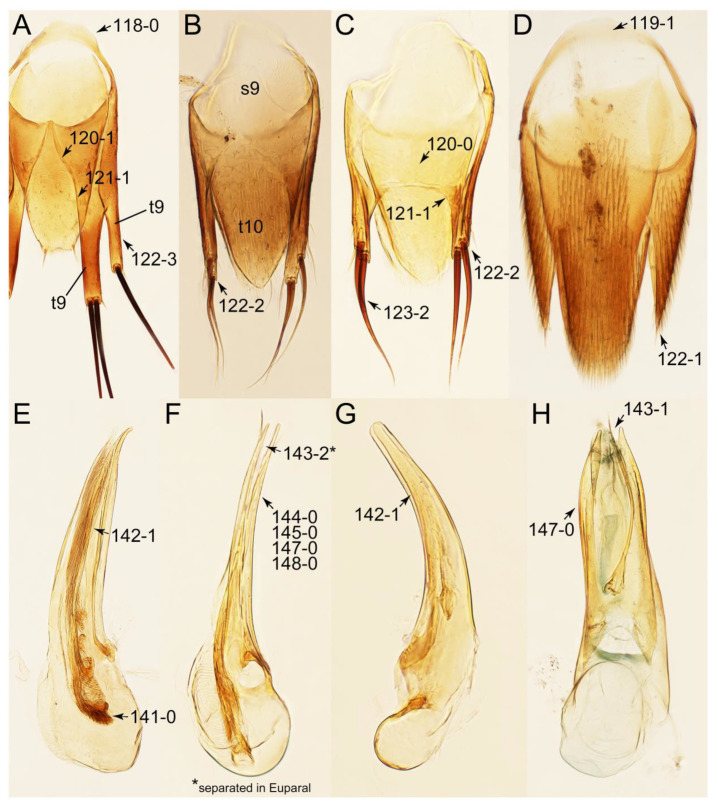
Photographs of body parts of Vatesini, male genital segments and aedeagus, enlarged. (**A**) tergite IX of *Cilea limbifera*, dorsal view. (**B**) tergites IX–X and sternite IX of *Coprotachinus habrocerinus*, dorsal view. (**C**) tergite IX of *Mimocyptus* sp., dorsal view. (**D**) tergite IX of *Vatesus gigas*, dorsal view. (**E**) aedeagus of *Cilea silphoides*, lateral view. (**F**) aedeagus of *Coprotachinus habrocerinus*, ventrolateral view. (**G**) aedeagus of *Mimocyptus* sp., lateral view. (**H**) aedeagus of *Vatesus praedatorius*, ventral view. Abbreviations: s9, sternite XI; t9–10, tergite IX–X. Characters and character states (format X-X) are indicated on each figure.

**Figure 34:**
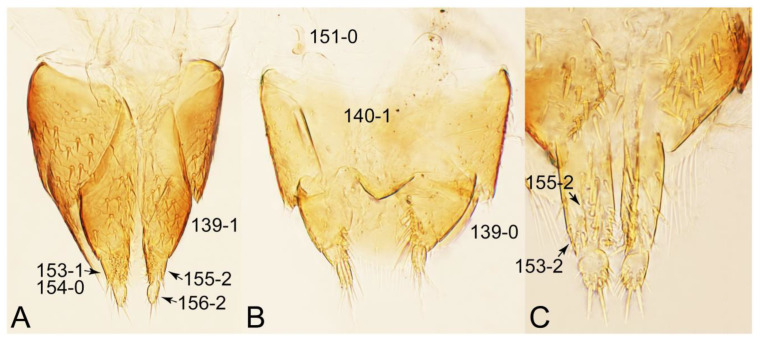
Photographs of body parts of Vatesini, female genital segments, enlarged. (**A**) *Coproporus laevis*, ventral view. (**B**) *Coprotachinus habrocerinus*, ventral view. (**C**) gonocoxites and gonostyli of *Cilea limbifera*, ventral view. Characters and character states (format X-X) are indicated on each figure.

**Figure 35:**
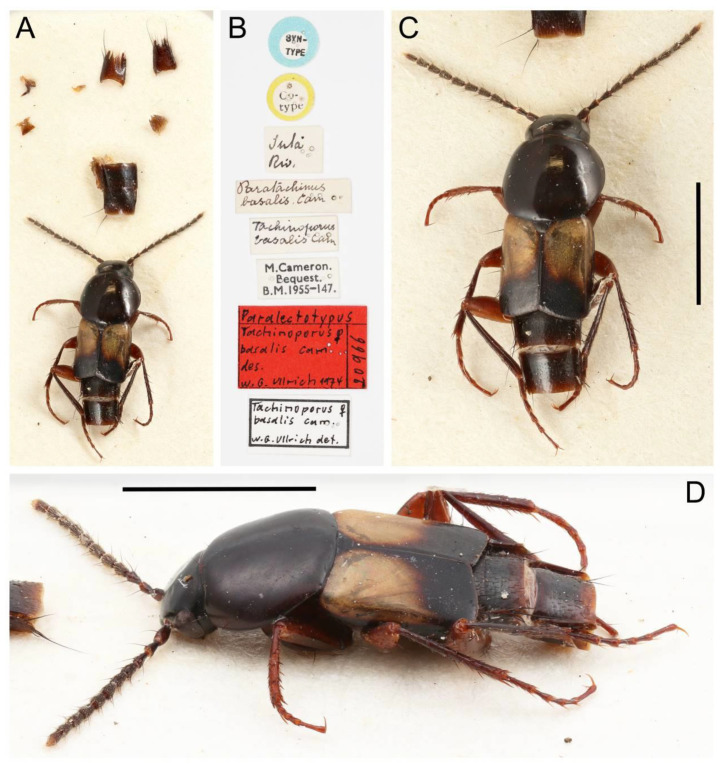
Photographs of Vatesini (*Tachinoporus basalis*, syntype, female). (**A**) habitus with dissected body parts glued to the paper card, dorsal view. (**B**) labels. (**C**) habitus, dorsal view. (**D**) habitus, dorsolateral view. Scale bars: 2.0 mm (**C**,**D**).

**Figure 36:**
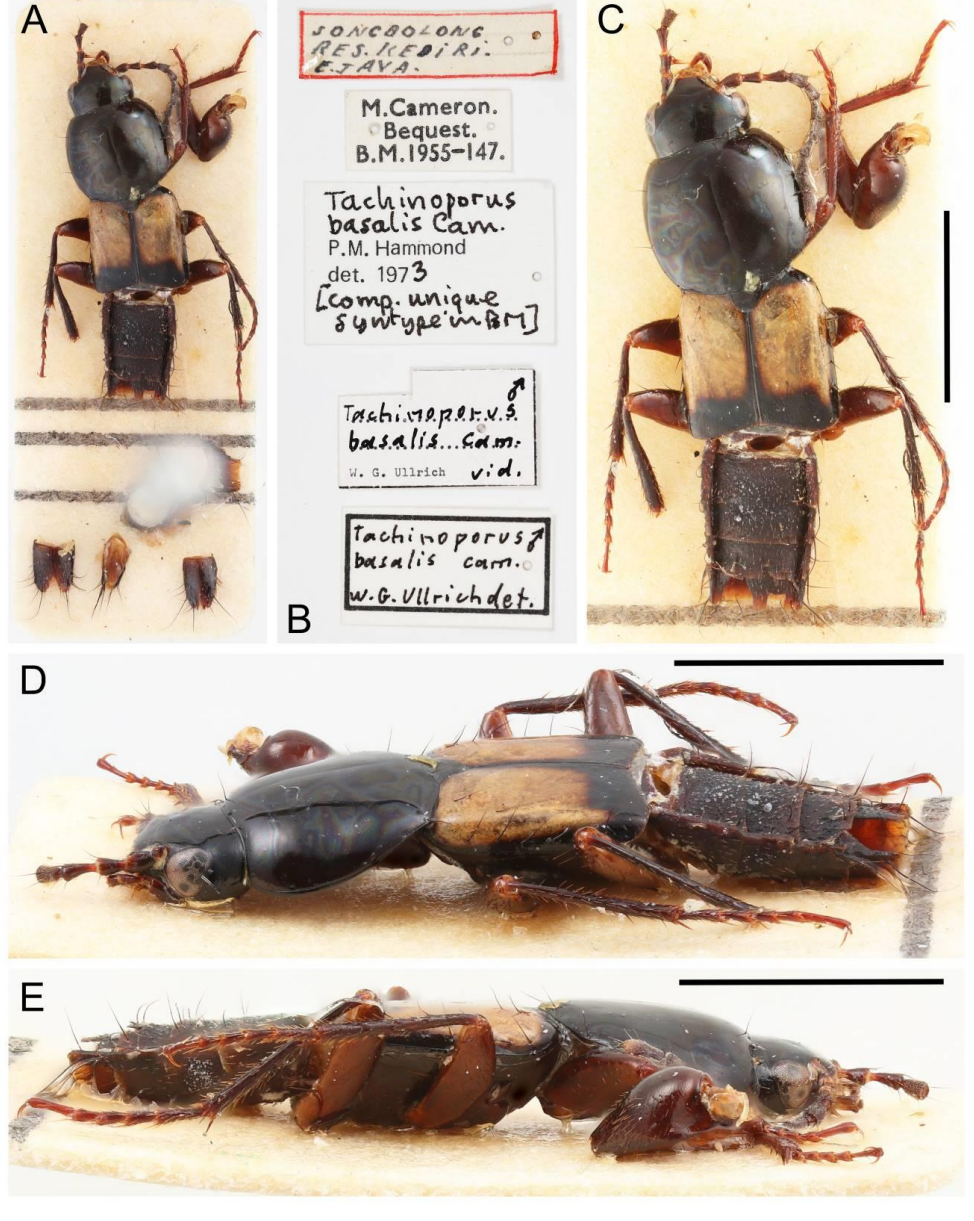
Photographs of Vatesini (*Tachinoporus basalis*, male). (**A**) habitus with dissected body parts glued to the paper card, dorsal view. (**B**) labels. (**C**) habitus, dorsal view. (**D**) habitus, dorsolateral view. (**E**) habitus, lateral view. Scale bars: 2.0 mm (**C**–**E**).

**Figure 37:**
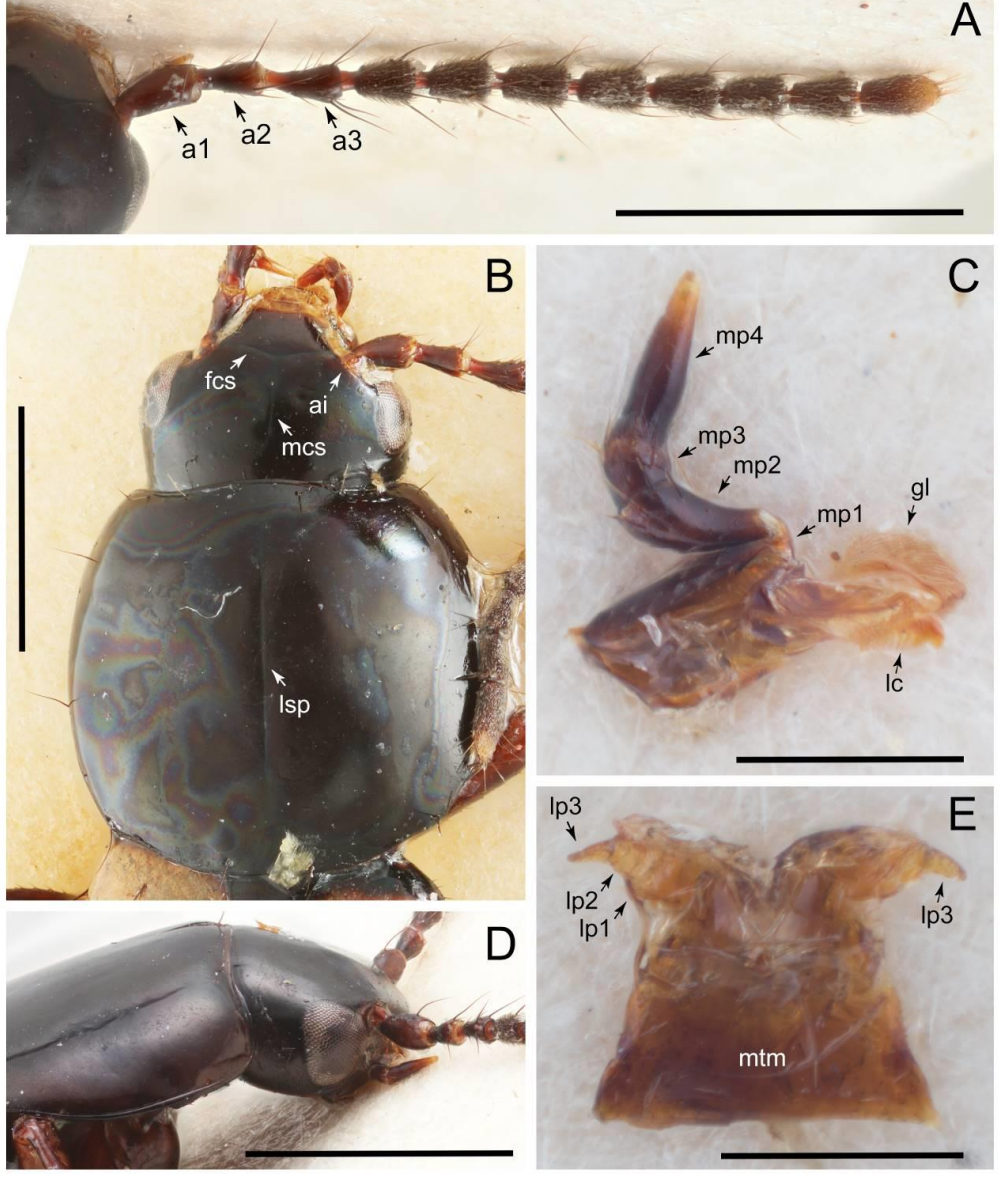
Photographs of Vatesini (*Tachinoporus basalis*). (**A**) right antenna of the syntype, dorsal view. (**B**) forebody, male, dorsal view. (**C**) right maxilla of the syntype, ventral view. (**D**) head and pronotum of the syntype, dorsolateral view. (**E**) labium and mentum of the syntype, ventral view. Scale bars: 1.0 mm (**A**,**B**,**D**); 0.3 mm (**C**,**E**). Abbreviations: a1–3, antennomere 1–3; ai, antennal insertion; fcs, frontoclypeal suture; gl, galea; lc, lacinia; lp1–3, labial palpomere 1–3; lsp, longitudinal sulcus on pronotum; mcs, midcranial suture; mp1–4, maxillary palpomere 1–4; mtm, mentum.

**Figure 38:**
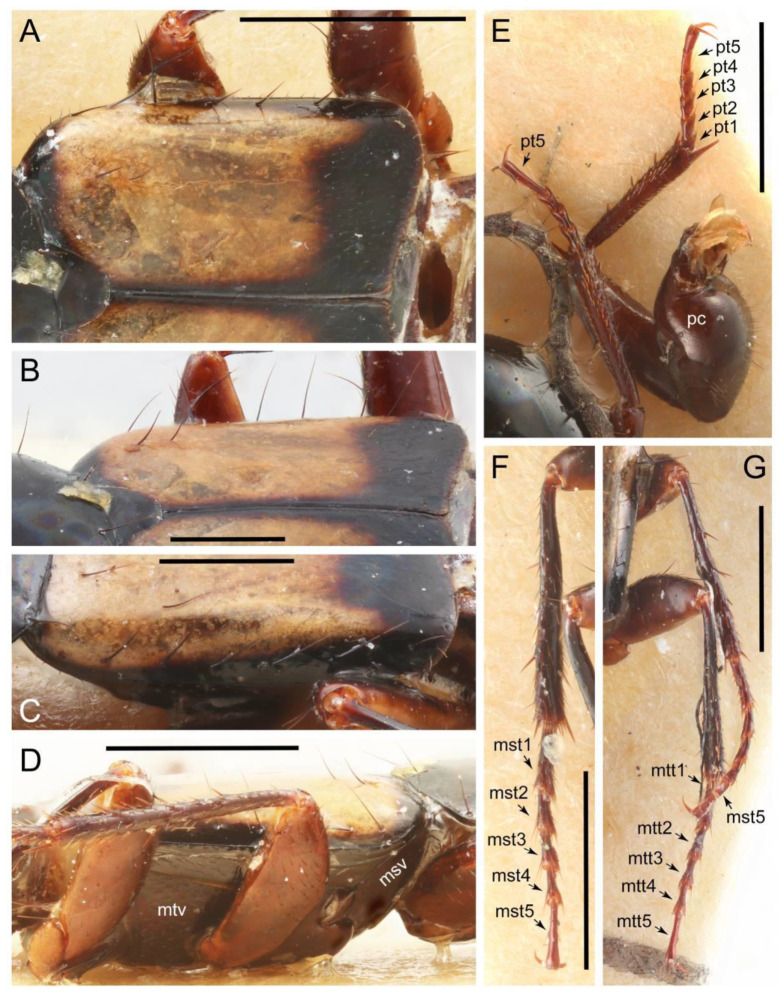
Photographs of Vatesini (*Tachinoporus basalis*, male). (**A**) scutellum and right elytron, dorsal view. (**B**) scutellum and right elytron, dorsolateral view. (**C**) left elytron, dorsolateral view. (**D**) meso- and metathorax, ventrolateral view. (**E**) forelegs. (**F**) left midleg, dorsal view. (**G**) right mid- and hindleg, dorsal view. Scale bars: 1.0 mm (**A**,**D**–**G**); 0.5 mm (**B**,**C**). Abbreviations: mst1–5, mesotarsomere 1–5; msv, mesoventrite; mtt1–5, metatarsomere 1–5; mtv, metaventrite; pt1–5, protarsomere 1–5.

**Figure 39:**
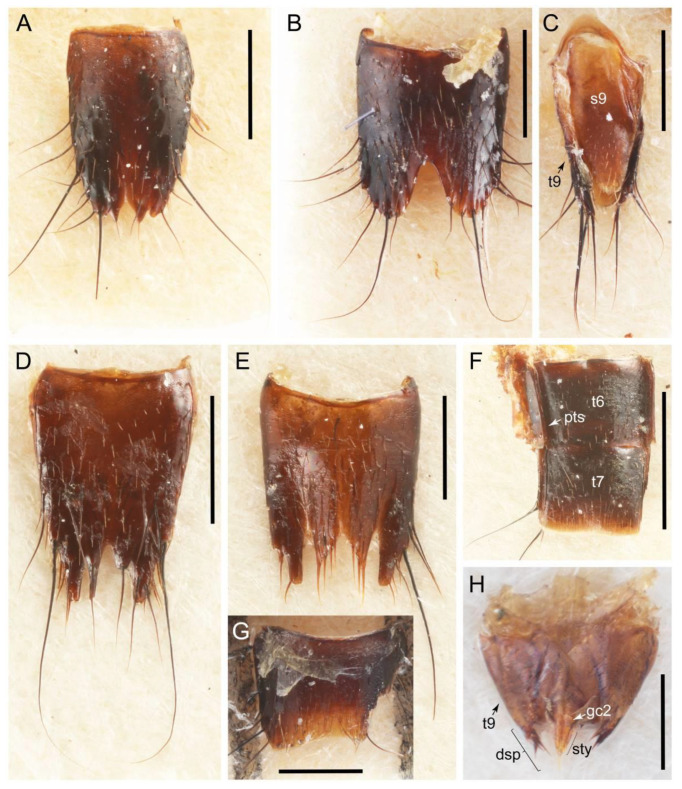
Photographs of Vatesini (*Tachinoporus basalis*). (**A**) male tergite VIII, dorsal view. (**B**) male sternite VIII, ventral view. (**C**) male tergite IX and sternite IX, ventral view. (**D**) female tergite VIII of the syntype, dorsal view. (**E**) female sternite VIII of the syntype, ventral view. (**F**) female tergites VI–VII and their associated paratergites of the syntype, dorsal view. (**G**) male sternite VII, ventral view. (**H**) female genital segments of the syntype, dorsal view. Scale bars: 0.5 mm (**A**–**E**,**G**); 1.0 mm (**F**); 0.3 mm (**H**). Abbreviations: dsp, developed short spines; gc2, gonocoxite II; pts, paratergites; s9, sternite IX; sty, gonostylus; t6–7, 9, tergite VI–VII, IX.

**Figure 40:**
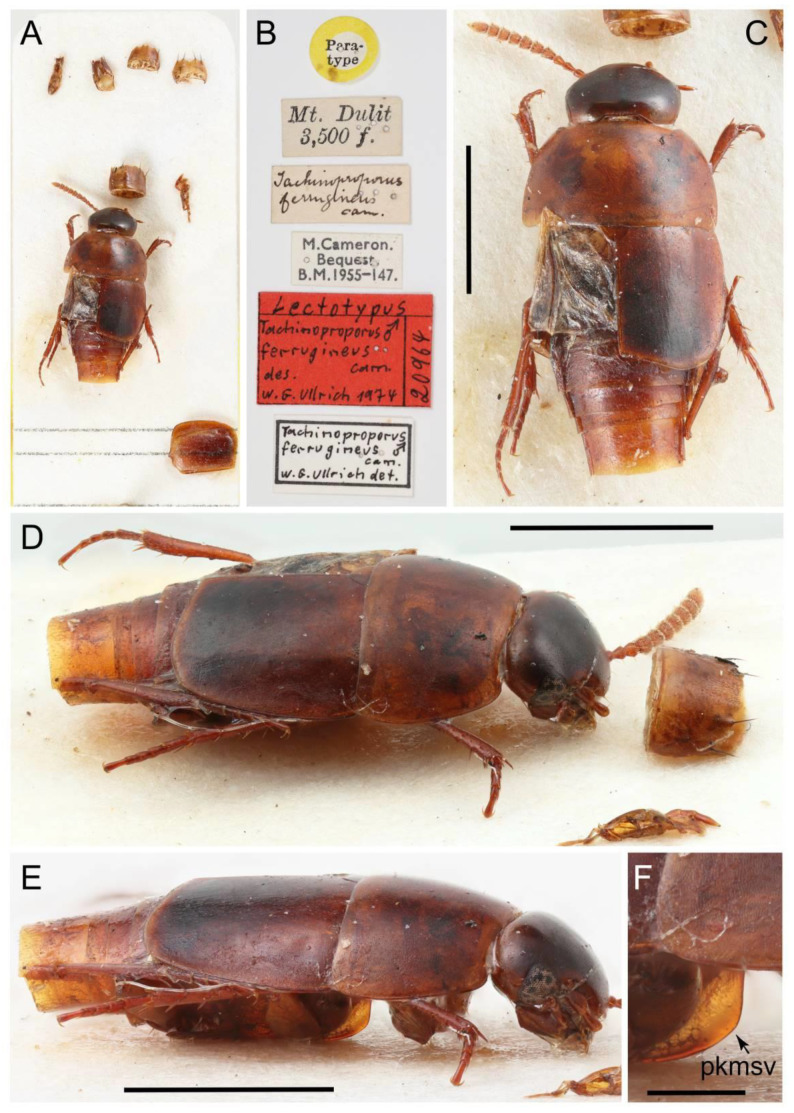
Photographs of Vatesini (*Tachinoproporus ferrugineus*, syntype, male). (**A**) habitus with dissected body parts glued to the paper card, dorsal view. (**B**) labels. (**C**) habitus, dorsal view. (**D**) habitus, dorsolateral view, and abdominal segment VII, ventrolateral view. (**E**) habitus, lateral view. (**F**) plate-like keel on mesoventrite, lateral view. Scale bars: 1.5 mm (**C**–**E**); 0.3 mm (**F**). Abbreviation: pkmsv, plate-like keel on mesoventrite.

**Figure 41:**
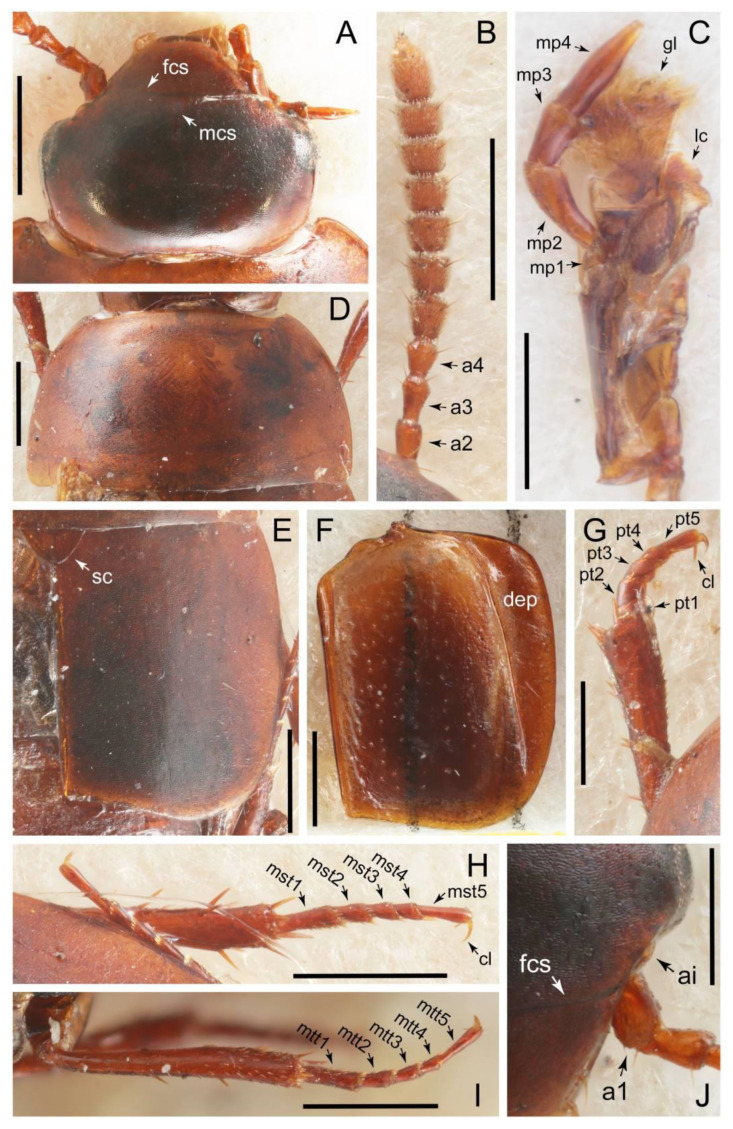
Photographs of Vatesini (*Tachinoproporus ferrugineus*, syntype, male). (**A**) head, dorsal view. (**B**) left antenna, dorsal view. (**C**) right maxilla, ventral view. (**D**) pronotum, dorsal view. (**E**) scutellum and right elytron, dorsal view. (**F**) left elytron, ventral view. (**G**) left foreleg, dorsal view. (**H**) right midleg, dorsal view. (**I**), left hindleg, dorsal view. (**J**), left antennal insertion of head, dorsal view. Scale bars: 0.5 mm (**A**,**B**,**D**–**F**,**H**,**I**); 0.3 mm (**C**,**G**,**J**). Abbreviations: a1–4, antennomere 1–4; ai, antennal insertion; cl, claw; dep, deeply folded elytral epipleuron; fcs, frontoclypeal suture; gl, galea; lc, lacinia; mcs, midcranial suture; mp1–4, maxillary palpomere 1–4; mst1–5, mesotarsomere 1–5; mtt1–5, metatarsomere 1–5; pt1–5, protarsomere 1–5; sc, scutellum.

**Figure 42:**
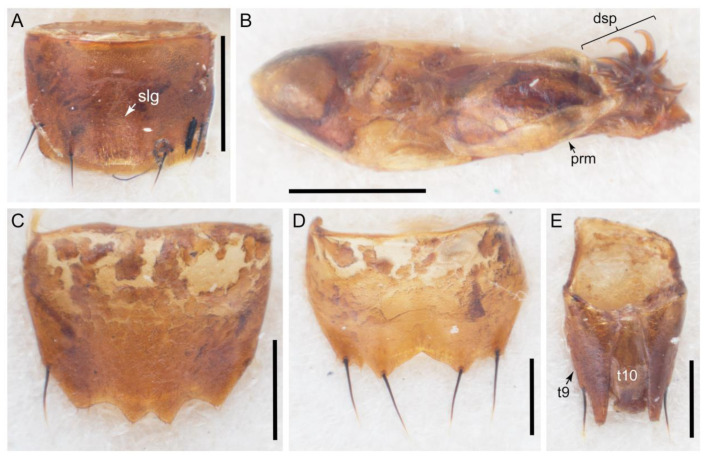
Photographs of Vatesini (*Tachinoproporus ferrugineus*, syntype, male). (**A**) sternite VII, ventral view. (**B**) aedeagus, ventral view. (**C**) tergite VIII, dorsal view. (**D**) sternite VIII, ventral view. (**E**) tergites IX–X, dorsal view. Scale bars: 0.5 mm (**A**); 0.3 mm (**B**–**E**). Abbreviations: dsp, developed short spines; prm, paramere; slg, shallow longitudinal groove on the sternite VII; t9–10, tergite IX–X.

Description: Body (Figures 21, 22, 35C,D, 36C–E and 40C–E) minute to large for tachyporines, sub-limuloid to streamlined, rather strongly to markedly convex above in cross section; forebody strongly glossy (Figures 21A–E,G,H and 22A,B) (with large micro-reticulation resulting in matte dorsum in *Tachinoproporus*; Figure 41D,E), generally glabrous (e.g., Figures 24D and 30A: 53-0, 74-0), occasionally covered with inconspicuous microsetae (Figure 25A: 74-1), without distinct punctation (e.g., Figures 23B and 24D: 3-0, 73-0). Head (Figures 23, 24A–C, 26A, 37B,D and 41A) with eyes incorporated into head capsule, lacking vertexal ocular seta; postocular areas with very weak neck-like narrowing, just behind of eyes in derived taxa (Figure 26A: 12-1), but sometimes absent (Figure 23A: 12-0). Antenna (e.g., Figures 23, 26D,G, 37A and 41B) usually short to very short, shorter than pronotal width or even shorter than head width in many taxa (e.g., Figures 22B, C and 23B: 20-1), but longer in earliest diverging lineages (e.g., Figure 21A, B); structure diverse, fili-moniliform to clubbed (e.g., Figures 26D, 37A and 41B), or myrmecophilous form (*Vatesus*; Figure 26G: 19-2), usually with clear patterns of dense and fine recumbent pubescence (except *Mimocyptus*), i.e., lacking such setae on basal three or four antennomeres (Figure 26D: 21-1). Maxillary palpus (Figures 27A–D, 37C and 41C) elongate, usually generally lacking ground setae, except for limited macrosetae (Figure 27A: 32-2), but with some exceptions (i.e., *Vatesus*, *Mimocyptus*; Figure 27C: 32-1); palpomere 3 widest at or near apex (Figures 27C: 30-0); palpomere 4 more or less pointed, widest at or near base (Figure 27A: 33-0), longer than penultimate palpomere in most taxa (Figure 27A,D: 34-2) (except in *Vatesus* and *Mimocyptus*; Figure 27C: 34-0). Maxillary lacinia (Figures 27A–D, 37C and 41C) without unarticulated large, long spine (Figure 27B: 37-0), with numerous short teeth or spines in most taxa (Figure 27B: 38-1), except in *Mimocyptus*. Labial palpi (Figure 26B,E,F: 46-1, 46-2) generally widely or relatively widely separated at base; palpomere 2 strongly to markedly transverse in crown genera (Figure 26E,F: 39-1), but elongate in *Cilea* and *Vatesus* (Figure 26B: 39-0); palpomere 3 elongate, usually widest at middle or near base, but not at base (Figure 26B, F: 40-1), with inconspicuous pores in many examples (Figure 26B: 43-1), outer margin usually with a few to several thick, short peg-like spines (Figure 26E: 42-1). Mentum (Figure 26F: 47-2, 48-0) weakly to moderately transverse, with truncate or rounded anterior margin. Pronotum (Figures 22A,B, 23B, 24, 27E–G, 37B and 41D) normal, more or less bell-shaped, without macrosetae on dorsum (except *Tachinoporus*; Figure 37B); anterior margin usually weakly to moderately concave, usually with angulate anterior angles (e.g., Figure 27G: 56-1); posterior margin in few taxa with conspicuous clusters of blackish pores (cf. Figure 54G: 58-1), but absent in most taxa (Figure 27E–G: 58-0). Pronotal hypomeron (Figure 27E–G) wide in basal third (Figure 27G: 60-2), strongly inflexed, not visible in lateral view, with weakly developed, short and rounded postcoxal process (e.g., Figure 27E: 61-1). Prosternum (Figures 24A–C and 27E–G) with sternacoxal ridge (*tsr*) very close to anterior margin of prosternum in medio-lateral areas (Figure 27E: 63-1), without prosternal process. Elytron (Figures 21A–E,G,H, 22A,B, 24D, 30, 35C,D 36C,D, 38A–C, 40C–E and 41E, F) short in crown group such as *Coproporus* (length/width: <1.3×; Figure 30A, B: 70-2), but moderately long in earliest diverging genera such as *Cilea*, basically lacking macrosetae on dorsum (Figures 24D and 30A: 75-0, 76-0, 77-0) (except *Tachinoporus*; Figure 38A–C), but with very narrow to narrow epipleural gutter (Figure 24D: 79-2) along outer margin in most cases; lateral side with epipleural ridge or keel, which is strongly to very strongly folded inward (Figures 24A,B and 30: 81-2, 81-3), resulting in thin, blade-like lateral and posterior elytral margins (Figure 30: 82-1); posterolateral margin in *Vatesus* distinctly sinuate (Figure 30C: 83-2). Mesoventrite (Figures 24A–C and 28A–D) with (Figure 28B,C: 85-1, 85-2) or without (Figure 24B: 85-0) weakly raised longitudinal median carina, markedly developed, plate-like in *Tachinoproporus* (Figure 40E,F). Intermesocoxal process with depressed isthmus in derived genera such as *Termitoplus* (Figure 28A,B,D: 88-4, 88-5). Metendosternite (Figure 28G,I) with anterior arms thick and lamellate (Figure 28G: 90-2), but inconspicuous in *Cilea*. Legs (e.g., Figures 21A,F, 22C, 24A–C, 25C, 28A,E, 29, 35C,D, 36C–E, 38D–G, 40C–E and 41G–I) usually short, rarely moderate in length; structure normal in most taxa, but myrmecophilous form in *Vatesus* (Figure 29B,D: 93-1); procoxae (Figures 22C, 24A–C, 25C, 29A and 38E) distinctly developed, very large, much larger than profemora (Figures 24C, 29A: 96-3); metacoxae (Figures 24A,B and 28A,B,E) medium-sized in each lateral half (Figure 24A: 98-1); metatibia with long spurs at apex, clearly longer than 2/5 of metatarsomere 1 (Figure 29C: 101-1); metatarsus long, more than half length but shorter than whole length of metatibia (e.g., Figures 28E and 29B: 103-0). Abdomen (e.g., Figures 21, 22, 25, 36C,D and 40C–E) moderately to very strongly tapering from base to apex, lacking blackish macrosetae dorsally (except abdominal terminalia); posterolateral margins of visible basal four segments usually extending posteriorly (Figures 25A,B and 28F: 106-1); paratergites comprised of single pair on segments III–VII in crown group (124-0), but with two pairs in earliest diverging lineages (Figure 39F, *pts*). Tergites III–VII (Figure 25A: 109-0) without macrosetae (except *Tachinoporus* and *Vatesus berghoffae*; Figure 35C,D); each tergite usually strongly transverse, but seemingly much less transverse in *Cileoporus* (Figure 21B) and *Tachinoporus* (Figure 35C). Tergite VIII (Figures 31, 39A,D and 42C: 114-1) with only a few to several distinct macrosetae (Figure 31A: 114-1), except *Vatesus* (Figure 31B: 114-2); posterior margin in both sexes usually with lobes (Figure 31B: 116-1, 117-3). Male tergite IX (Figures 33A–D, 39C and 42E) fused at base of ventral side, not divided by sternite IX (Figure 33D: 119-1); dorsal side (Figures 33A–D and 42E) abruptly divided apically in basal 1/3 (Figure 33A: 120-1) (not in *Mimocyptus*; Figure 33C: 120-0), usually forming on each side a slender lobe with pointed apex (Figure 33B,C: 122-2) (two pairs each in *Cilea*; Figure 33A: 122-3), or less slender lobe, merely elongate in limited taxa (i.e., *Cileoporus* and *Vatesus*; Figure 33D: 122-1); apical areas each with at most several long macrosetae (123-2) (except *Tachinoporus*; Figure 39C), restricted to apices (except for *Cileoporus*). Sternite III without longitudinal median carina. Male sternite VII not emarginate or concave (except *Cilea*), lacking peg-like setae (Figure 25C: 131-0). Sternite VIII (Figures 32, 39B,E and 42D) with only a few to several distinct macrosetae (Figure 32A: 133-1), except *Vatesus* (Figure 32D: 133-2) and *Tachinoporus* (Figure 42D); posterior margin in male (Figures 32C–E, 39B and 42D) with diverse structures, usually widely emarginate with weakly developed lobes (Figure 32E: 135-4), that of female (Figures 32A,B, 39B and 42D) with developed lobes and associated smaller internal lobes (Figure 32A: 137-3), each apex in female with single pair or three sensory setae (Figure 32B: 138-3) (except *Vatesus*). Male aedeagus (Figures 33E–H and 42B) narrowly elongate, usually slender, moderately curved or arcuate in lateral view; parameres longitudinally contiguous (Figure 33F: 143-2) to moderately separated (Figure 33H: 143-1), not flattened or plate-like (Figure 33F: 145-0). Female genitalia (Figures 34 and 39H) with gonocoxite II usually more or less triangular (Figure 34A: 153-1), but narrowly elongate in ‘lower’ genera (Figures 34C and 39H: 153-2), bearing both normal and curved setae (Figure 34A,C: 155-2).

Composition: Ten genera (1 extinct), with 290 species (2 extinct). See Table A1 for overview and distributions.

- *Cilea* Jacquelin du Val, 1856 [93]: 25.
  Type species: *Staphylinus silphoides* Linnaeus, 1767 [94]: 684.
- *Cileoporus* Campbell, 1994 [91]: 126.
  Type species: *Tachinus politus* Bernhauer, 1923 [95]: 59.
- *Coproporus* Kraatz, 1857 [96]: 399.
  Type species: *Coproporus colchicus* Kraatz, 1858 [97]: cxc.
- *Coprotachinus* Cameron, 1933a [98]: 44.
  Type species: *Tachinus congoensis* Cameron, 1926 [99]: 283 (= *Erchomus ampliatus* Fauvel, 1905 [100]: 198).
- *Mimocyptus* Cameron, 1919 [101]: 241.
  Type species: *Mimocyptus globulus* Cameron, 1919 [101]: 241.
- *Tachinoporus* Cameron, 1928 [11]: 447.
  Type species: *Tachinoporus basalis* Cameron, 1928 [11]: 448.
- *Tachinoproporus* Cameron, 1928 [11]: 449.
  Type species: *Tachinoproporus ferrugineus* Cameron, 1928 [11]: 449.
- *Termitoplus* Silvestri, 1946 [12]: 547.
  Type species: *Termitoplus grandis* Silvestri, 1946 [12]: 551.
- *Vatesus* Sharp, 1876 [92]: 201.
  Type species: *Vatesus latitans* Sharp, 1876 [92]: 202.
- †*Procileoporus* Yamamoto, 2016 [46]: 165.
  Type species: †*Procileoporus burmiticus* Yamamoto, 2016 [46]: 167.

Fossils: The extinct monotypic genus †*Procileoporus* was established based on †*P. burmiticus* Yamamoto, 2016 from mid-Cretaceous Burmese amber from Kachin State, northern Myanmar [46]. This extinct species was described on the basis of a rather poorly preserved male adult, but I now have several well-preserved adult fossils of the same species for future comparative study (Yamamoto, in prep.). As discussed in Yamamoto [46], †*Procileoporus* generally resembles the four vatesine genera, i.e., *Cilea*, *Cileoporus*, *Coproporus*, and *Coprotachinus*. Among them, †*Procileoporus* is particularly similar to *Cileoporus* in having a slender body and elongate elytron, together with similar configurations of the abdominal tergite IX in the male [46]. For these reasons, this fossil genus is herein unambiguously assigned to Vatesini sensu nov.

So far, only a single fossil has been definitively assigned to the extant genus *Coproporus*, namely *C. electron* Yamamoto, 2016, from early Middle Miocene Dominican amber [102].

Remarks: Until now, the tribe Vatesini has been comprised of only the single myrmecophilous genus *Vatesus*, associated with army ants, from the Neotropical region. However, my phylogenetic analyses did not show at all that it was a tribe-level group. Certainly, *Vatesus* was fully resolved among the genera of the *Coproporus*-related group of Tachyporini. Consequently, the present study greatly expands the concept of the tribe, encompassing the ten genera listed above. A close relationship between *Coproporus* and *Vatesus* has independently been suggested by larval and adult morphologies, respectively [25,27]. In light of the previous studies and current results, a newly defined Vatesini sensu nov., including the *Coproporus*-related taxa, is one of the key solutions to resolve the polyphyly of Tachyporini in the old sense (see further in Discussion).

It is noteworthy to mention here that there are two enigmatic monobasic tachyporine genera, i.e., *Tachinoporus* and *Tachinoproporus*, described from Malaysian Borneo. No additional specimen of either genera has been known since the original descriptions [11]. In this study, I was fortunately to be able to examine these type materials deposited in the BMNH, which had been under a long-term loan, resulting in limited available information for a long time. Consequently, there have been no photograph, line drawing, or redescription published for these taxa despite their importance in tachyporine systematics. Here, I provide informative photographs and detailed descriptions below, along with discussions of their phylogenetic affinities. However, I refrained from including the genera in my phylogenetic analyses because these specimens seem very fragile, and I was allowed only to observe them from dorsal and lateral views. Furthermore, it was either impossible to score some important morphological characters or difficult to score characters accurately. In this study, I did not detach the glued specimens from the paper cards, to avoid further damage to the specimens (e.g., erect setae, setose surface of the abdomen, repaired hindleg, and well-organized antennae). This also prevents degradation of DNA information of specimens through resolution with water. Unfortunately, some body parts, such as male genitalia, are missing or embedded in glue, preventing accurate scoring of morphological characters. Although the overall conditions of *Tachinoporus* and *Tachinoproporus* are rather poor, these specimens allow for the elucidation of tribal affinities, in addition to other information. In conclusion, they should belong to Vatesini sensu nov. based on the results of my morphological investigations (see below).

#### 3.2.8. Genus *Tachinoporus* Cameron, 1928

*Tachinoporus* Cameron, 1928: 447 [11] (Figures 35–39; Table A1)

Type species: *Tachinoporus basalis* Cameron, 1928 [11]: 448.

Differential diagnosis: This enigmatic genus can be distinguished from all remaining genera of Vatesini sensu nov. by the following combination of characters: Body large, slender, antennae longer than head and pronotum combined, devoid of dense and fine recumbent setae in basal three antennomeres, pronotum widest in middle, with longitudinal sulcus along midline, bearing stout setae along margins, elytron somewhat strongly elongate, with stout setae, legs long and slender, abdomen with two pairs of paratergites, and abdominal terminalia with very long macrosetae in both sexes.

Emended diagnosis based on dorsal and lateral views: Body large (more than 5.5 mm long), slender, rather streamlined, moderately depressed dorsoventrally (Figures 35A,C,D and 36A,C–E); surface strongly glossy, dorsum lacking microsetae, punctation, or micro-reticulation on forebody; head with arcuate sides, lacking narrowing of neck region, dorsum with markedly developed midcranial suture, extending posteriad beyond middle of vertex (Figure 37B); antenna filiform, not flattened nor modified, moderately longer than head and pronotum combined, basal three antennomeres devoid of dense and fine recumbent setae (Figure 37A); maxillary palpomeres 3 and 4 glabrous with several setae on surface near each apex (Figure 37C); pronotum as long as wide, widest in middle, surface with inconspicuous longitudinal sulcus along midline (*lsp*), sides strongly arcuate, all pronotal margins furnished with row of macrosetae (Figure 37B); elytron rather strongly elongate (length/width = ca. 2.0×), dorsoventrally flattened, with several macrosetae along lateral margin (Figure 38A–C); elytral epipleuron rather deeply folded inward, visible from lateral view (Figure 38D); legs long and slender (e.g., Figures 35A,C and 36A,C); abdomen slender, with two pairs of paratergites on segment III–VII (Figure 39F, *pts*), furnished with distinctly long macrosetae on both dorsal and ventral sides of abdomen; tergite VIII and sternite VIII somewhat strongly elongate, each furnished with several long macrosetae, some of which extremely long: male tergite VIII (Figure 39A) with weakly developed lobes, female tergite VIII (Figure 39D) with moderately to strongly developed lobes, male sternite VIII (Figure 39B) with posterior margin deeply and narrowly emarginate, female sternite VIII (Figure 39E) with weakly to moderately developed lobes, flanking distinct median emargination; male tergite IX (Figure 39C) with each side forming pointed lobe, with several macrosetae on its apical surface; female tergite IX (Figure 39H) subtriangular, weakly transverse, uniformly well sclerotized, with several spines at each apex.

Redescription based on dorsal and lateral views: Body (Figures 35A,C,D and 36A,C–E) large, narrowly elongate, moderately flattened dorsoventrally, *Cileoporus*-like, widest in pronotum. Dorsum strongly glossy; head and pronotum without ground setae, punctation, or grooves.

Head (Figure 37B,D) hexagonal, slightly transverse, moderately produced anteriorly, widest across eyes, with gently arcuate postocular sides; frontoclypeal suture (Figure 37B) complete, well-developed, shallowly grooved, weakly V-shaped; midcranial suture (Figure 37B, *mcs*) distinctly developed, extending posteriad beyond middle of vertex; neck constriction or narrowing completely absent in postocular areas (Figure 37B); dorsal surface lacking microsetae, punctation, or ocular seta (Figure 37B); ventral surface without infraorbital ridge extending ventrally from posterior margin of eye. Clypeus (Figure 37B) normal, not reflexed upward, narrowly produced anteriorly. Eyes (e.g., Figure 37A,B,D) moderate in size, somewhat inconspicuous, nearly even with surface of head capsule. Antennal insertions (Figure 37A,D) partially concealed under frontal shelf-like projections, located near anterior margins of eyes. Antenna (e.g., Figure 37A) comprised of 11 antennomeres, filiform, very slender, long, clearly longer than head and pronotum combined; antennomeres 4–11 (*a4–a11*) slightly flattened; basal three antennomeres glossy, devoid of dense and fine recumbent setae, remaining antennomeres densely covered with whitish microsetae. Maxillary palpus (Figure 37C) four-segmented, elongate, moderate in size and length, lacking recumbent microsetae on surface; palpomere 1 (*mp1*) very short; mp2 long, narrowly elongate, moderately curved, gradually strongly dilated apically, with several short setae on surface near apex; mp3 very short, as long as wide, much shorter than mp2, weakly dilated apically, with several setae on surface near apex; mp4 markedly long, approximately three times as long as mp3, moderately longer than mp2, moderately narrower than mp3, sides gradually and evenly tapering from middle to pointed apex. Labial palpus (Figure 37E) three-segmented, elongate, inconspicuous, lacking ground microsetae on surface; palpomere 1 (*lp1*) very short, only partially visible; lp2 slightly longer; lp3 slender, much longer and clearly narrower than lp2, as long as lp1, gradually narrowing toward apex, rather pointed, with more than ten pores on surface. Mentum (Figure 37E) moderately transverse, trapezoidal, with truncate anterior margin. Labium with ligula large, developed, extending laterally.

Pronotum (e.g., Figure 37B,D) oval, feebly transverse, widest in middle, much larger than head; sides strongly arcuate, evenly convex; each margin with a few macrosetae, but lacking ground microsetae; anterior margin weakly concave; anterolateral angles rounded; disc with shallow and very narrow longitudinal sulcus along midline (see, arrow of *lsp*), but reaching neither anterior nor posterior margins of pronotum. Scutellum (Figure 38A,B) visible dorsally, sub-triangular, as long as wide, with rounded apex. Elytron (e.g., Figure 38A–C) rather strongly elongate (length/width = ca. 2.0×, male, *n* = 1), subparallel sided, dorsoventrally flattened, only slightly longer than pronotum, with rounded posterior margin; lateral margins not arcuate, with a row of several long macrosetae; surface even and smooth, rather densely covered with vestigial ground microsetae. Elytral epipleuron (Figure 38D) relatively deeply folded inward, but visible from lateral view. Wings (Figure 38A,B) visible, probably fully developed. Mesoventrite (Figure 38D) with surface even and smooth, without carina or keel along midline.

Legs (e.g., Figures 35C, 36C, and 38E–G) long, distinctly slender, with irregularly distributed, somewhat inconspicuous spines on tibiae. Procoxae (e.g., Figure 38E) large, expanded; protibiae (e.g., Figure 38E) rod-like, with markedly long apical tibial spurs, longest spur much longer than protarsomere 1 (*pt1*); protarsi (e.g., Figure 38E) 5-segmented, narrow in both sexes, moderately shorter than protibiae, basal four tarsomeres each small and short, together moderately longer than pt5. Mesotibiae (e.g., Figure 38D,F) with developed spurs at each apex, but much shorter than mesotarsomere 1 (*mst1*); mesotarsi (e.g., Figures 36C–E and 38F,G) 5-segmented, very long, as long as mesotibiae, mst1 (Figure 38F) much longer than mst2, as long as mst5. Metacoxae transverse, moderate in size; metacoxal ventral lamella projecting posteriorly in mesial inner areas (Figure 38D); metatibiae with only weakly developed, very short spurs at apex; metatarsi (e.g., Figures 36C,E and 38F) 5-segmented, markedly long, slightly longer than metatibiae, basal metatarsomere much shorter than following three segments combined (see Campbell [103]). Claws (e.g., Figure 38E) simple, lacking conspicuous basal teeth. Empodial setae very short, inconspicuous, not extending distally beyond level of basal fourth of tarsal claw.

Abdomen (e.g., Figures 35C,D and 36C) narrowly elongate, slightly triangular, evenly tapering from base to apex; maximum width moderately narrower than elytra; surface sparsely covered with fine short setae, lacking V-shaped punctures, but with several macrosetae even on tergites. Abdominal segments III–VII each with two pairs of paratergites, large in size, vertical (Figure 39F). Abdominal segments III–VI including paratergites, with weakly produced posterolateral margins. Tergites lacking both pruinose spots and basolateral ridges (Figure 39F). Tergite III only feebly visible (in situ, syntype of female of *T. basalis*), moderately transverse.

Male. Tergite VIII (Figure 39A) rather strongly elongate, with only weakly developed lobes, bearing numerous blackish macrosetae, some of which are extremely long. Tergite IX (Figure 39C) with each side forming elongate lobe, bearing several macrosetae on apical third; ventral side basally fused, not divided by sternite IX at base. Sternite VI with posterior margin truncate, lacking peg-like setae. Sternite VII (Figure 39G) with posterior margin broadly emarginate and concave, surface lacking peg-like setae or spines. Sternite VIII (Figure 39B) moderately elongate, with posterior margin rather deeply and widely emarginate, bearing numerous blackish macrosetae, some extremely long. Sternite IX (Figure 39C) with posterior margin rounded. Genitalia missing; no information available.

Female. Tergite VIII (Figure 39D) strongly elongate, with moderately developed lobes, bearing numerous blackish macrosetae, some extremely long. Tergite IX (Figure 39H) subtriangular on each side, weakly transverse, uniformly well sclerotized; apices of each side with several short spines (see *dsp*); surface coarsely covered with large, transverse micro-reticulation. Sternite VIII (Figure 39E) moderately elongate, with weakly to moderately developed lobes, bearing numerous blackish macrosetae, some extremely long. Gonocoxites (Figure 39H) moderate in size, gonocoxite II subtriangular, relatively thick, large, as long as gonostylus, with normal, thin setae; gonostylus subconical, narrowly elongate, each apex with long, testaceous macroseta. Spermatheca not visible.

Composition: Only the type species is known [11,21].

Remarks: *Tachinoporus* is an enigmatic monobasic genus described from Malaysian Borneo [11]. Since the original description in the late 1920′s, there has been no information published regarding this genus, except for a few catalogues (e.g., [21,104]) Due to the significant lack of information, *Tachinoporus*, together with *Tachinoproporus* (redescribed below), has been known as one of the most problematic tachyporine genera, although it was placed in Tachyporini in the traditional sense [21]. In the present study, I provide sufficient photographs and descriptions for the genus to unravel its systematic identity. Based on morphological evidence, *Tachinoporus* can be placed in the newly revised concept of the tribe Vatesini sensu nov. based on the general *Cileoporus*-like habitus, the structures of the maxillary palpus and sternite VII in the male, and other morphological evidence described above.

Although I have excluded *Tachinoporus* from the phylogenetic analyses here, it is generally similar to *Cileoporus* based on the following features ([91]; this study): body slender, rather streamlined; antennae long, with basal three antennomeres devoid of dense and fine recumbent pubescence; pronotum widest at middle, not base or near base; abdomen slender, with distinctly long macrosetae on both dorsal and ventral sides; paratergites comprised of two pairs; tergite VIII and sternite VIII rather strongly elongate, each bearing extremely long macrosetae, and; tergite IX with each side bearing several macrosetae. It is unfortunate that the male genitalia of the single non-type male had originally been lost. However, *Tachinoporus* can be easily distinguished from *Cileoporus* by the following characters: midcranial suture much more developed, grooved, extending beyond middle of vertex; antennae filiform, thin, much longer; pronotum as long as wide, surface with inconspicuous longitudinal sulcus along midline, all pronotal margins furnished with row of macrosetae; elytra dorsoventrally flattened, with several macrosetae along lateral margins, and; tergite IX with each side modified to be lobe-like. Some of the characters listed above (e.g., the presence of longitudinal sulcus on pronotum) are quite unusual, or rarely seen, in the Tachyporinae sensu nov. Thus, there is no doubt that *Tachinoproporus* is indeed a valid and separate genus. Interestingly, *Cileoporus*, a suspected sister genus, is distributed in Central and South America, possibly implying a biogeographic connection between these two genera. Additional specimens will be needed to clarify more details, including the male genitalia, and to extract molecular information.

#### 3.2.9. *Tachinoporus basalis* Cameron, 1928

*Tachinoporus basalis* Cameron, 1928: 448 [11] (Figures 35–39; Table A1)

Type material: Syntype, 1 female: “SYN-|TYPE” < printed blue -bordered round label >, “Co-|type” < printed yellow-bordered round label >, “Tuta|Riv.” < handwritten on rectangular small white label >, “Paratachinus|basalis Cam” < handwritten on rectangular small white label >, “Tachinoporus|basalis Cam” < handwritten on rectangular small white label > “M.Cameron.|Bequest|B.M. 1955-147.” < printed on rectangular small white label >, “Paralectotypus|Tachinoporus ♀|basalis Cam.|des.|W. G. Ullrich 1974|20966 [written vertically]” < handwritten on rectangular small red label >, “Tachinoporus ♀|basalis Cam.|W. G. Ullrich det.” < handwritten on rectangular small white label > (BMNH). See Figure 35B.

Other material: 1 male: “*SONCBOLONG.*|*RES. KEDiRi.*|*E. JAVA*” < handwritten on rectangular small white label with red margin >, “M.Cameron.|Bequest|B.M. 1955-147.” < printed on rectangular small white label >, “Tachinoporus [handwritten]|basalis Cam. [handwritten]|P. M. Hammond|det. 1973 [3, handwritten]|[comp. unique [handwritten]|syntype in BM] [handwritten]” < printed on rectangular small white label >, “♂ [handwritten]|Tachinoporus [handwritten]|basalis Cam. [handwritten]|W. G. Ullrich vid. [vid., handwritten]” < printed on rectangular small white label, upper right corner rectangularly removed >, “Tachinoporus ♂|basalis Cam.|W. G. Ullrich det.” < handwritten on rectangular small white label > (BMNH). New to Indonesia. See Figure 36B.

Comments on material: In the original description of this species, only two female specimens were specified [11]. However, as indicated above, there is a pair of male and female specimens found in the BMNH. They are considered here as conspecific based on general morphological similarity. In both specimens, the ventral sides were not examined to avoid further damage to the valuable material. A paralectotype label written by Dr. Wolfgang G. Ullrich is attached with the female specimen (Figure 35B), which was collected from the type locality. However, the lectotype designation for the type species has never been formally published. In the present study, I am treating the female as a syntype.

In both specimens, the abdominal apices were dissected by a previous researcher and glued on the same paper card with the body (Figures 35A and 36A). In the male, the left foreleg was additionally removed from and attached near the body (Figure 36A,C). It is unfortunate that several body parts are missing in the male specimen, namely the left antenna beyond antennomere IV, left metatarsus, tergite VII, and the genitalia; in addition, tergite VI is heavily damaged. In the female, the left maxilla, labium including the mentum, and abdominal segments VI–VII were also removed from the body and mounted on the same paper card along with the body (Figure 35A).

Type locality: Borneo, Tutau River [11].

Distribution: Borneo (Sarawak, Malaysia), East Java (Indonesia: new country record).

Diagnosis: As for the genus (*vide supra*).

Redescription based on dorsal and lateral views: Measurements. Syntype: female (*n* = 1). Body length: 4.98 mm (excluding beyond abdominal segment V); head length: 0.87 mm; head width: 1.08 mm; antennal length: 2.56 mm (right); pronotal length: 1.50 mm; pronotal width: 1.70 mm; elytral length: 1.64 mm (right); elytral width: 0.84 mm (right); metatibial length: 1.51 mm (left). Non-type material: male (*n* = 1). Body length: 5.72 mm (excluding beyond abdominal segment VI); head length: 0.83 mm (basal area partially concealed by pronotum); head width: 1.12 mm; pronotal length: 1.60 mm; pronotal width: 1.68 mm.

Body (Figures 35A,C,D and 36A,C–E) large, exceeding 5.0 mm in length with complete abdomen. Color (e.g., Figures 35C,D and 36C) generally black to blackish brown, but elytra bicolored with yellowish brown basal areas and blackish brown apical areas; legs and; elytron of male with larger yellowish area than that of female.

Head (Figure 37B,D) medium in size, moderately transverse (width/length = 1.24×, female, n = 1); sides somewhat straight, gradually narrowed posteriorly; surface covered with very minute, dense, transverse micro-reticulation. Antenna (Figure 37A) markedly slender, notably long; antennomere 1 (a1) elongate, rather fusiform, widest in apical quarter, much longer than a2; a2 rather strongly dilated, much narrower than a1; a3 strongly dilated, moderately longer than a2 and a4, only slightly wider than a2; a4–a10 similar in shape and width, elongate, subcylindrical, weakly dilated anteriad; a4–a8 each with long, conspicuous stem; a11 elongate oval, symmetrical, only slightly longer than a10, weakly narrower than a10, with moderately rounded apex. Maxillary palpi (Figure 37C) with approximate relative length of each maxillary palpomere from base to apex: 3.3, 21.7, 7.8, 25.3; approximate relative width of each maxillary palpomere from base to apex: 5.6, 10.4, 8.9, 7.5.

Pronotum (e.g., Figure 37B,D) nearly as long as wide, rather strongly convex dorsally; surface densely covered with very fine, inconspicuous micro-reticulation, comprised of transverse striae. Elytron (e.g., Figure 38A–C) small, strongly elongate (width/length = 1.95×), with sparse yellowish vestigial microsetae; lateral margin lacking epipleural gutter (sensu Herman [25]), each with row of 6–8 blackish macrosetae (Figure 38B); anteromedial area furnished with two stout, but short, blackish macrosetae (Figure 38A,B); surface yellowish brown in apical 3/4 (female; Figure 35C) to 2/3 (male; Figure 38A), yellowish area slightly extending near lateral margin posteriorly, without micro-reticulation.

Legs (e.g., Figures 35C, 36C and 38E–G) long. Protibia (Figure 38E) with very long single apical spur, ca. 1.6 times longer than pt1 (left, male, n = 1). Metatarsus (Figures 36C,E and 38G) with approximate relative length of each metatarsomere from base to apex (left, female, n = 1): 38.5, 25.4, 28.3, 26.8, 44.2.

Abdomen (e.g., Figures 35C,D and 36C) somewhat strongly glossy, covered with transverse, fine, striae-like micro-reticulation. Abdominal tergites (Figure 35D) and sternites IV–V (only ones observable) with two to three pairs of blackish macrosetae.

Male. Tergite VIII (Figure 39A) with two pairs of weakly developed lobes located in mesial area of posterior margin, with addition of another lateral pair of very feebly developed protrusions, each bearing extremely long blackish macroseta, slightly exceeding maximum length of tergite VIII; mesial area shallowly acutely incised between inner lobes; inner lobes short, triangular, moderately longer and narrower than outer lobes, extending moderately beyond level of outer lobes; outer lobes much shorter, rather narrowly rounded; surface sparsely covered with short microsetae and approximately 16 macrosetae. Tergite IX (Figure 39C) with weakly developed secondary lobe near apices of each side. Sternite VII (Figure 39G) with approximately 7–8 pairs of macrosetae, one pair extremely long. Sternite VIII (Figure 39B) with posterior margin widely and deeply emarginate medially (*ca*. 1/3 length of sternite VIII in depth), with a lateral pair of protrusions; surface sparsely covered with short microsetae and approximately 16 macrosetae.

Female: Tergite VIII (Figure 39D) with two pairs of moderately developed lobes located in mesial area of posterior margin; mesial area acutely incised between inner lobes; inner lobes narrowly elongate, sharply pointed, slightly shorter and much narrower than lateral lobes; outer lobes narrowly elongate, extending slightly beyond level of inner lobes, with rounded apices; surface sparsely covered with short microsetae and approximately 13 macrosetae, one pair even slightly exceeding maximum length of tergite VIII. Sternite VIII (Figure 39E) with two pairs of moderately developed lobes located in mesial area of posterior margin, with addition of another lateral pair of feebly developed projection-like lobes, each projection bearing extremely long blackish macroseta, slightly shorter than maximum length of sternite VIII; mesial area moderately angulately incised between inner lobes; inner lobes triangular, moderately shorter than lateral lobes, with sharply pointed apices; outer lobes narrowly elongate, extending strongly beyond level of inner lobes, with nearly truncate, broadly rounded apices; surface sparsely covered with short microsetae and approximately 6 macrosetae in lateral half.

Remarks: Although the redescription above is based merely on the dorsal and lateral views, I was able to extract sufficient characters for this enigmatic taxon. The single male specimen is generally similar to the female syntype in many aspects. Nevertheless, there is a color variation found in elytra between these specimens: the male has a more extended yellowish area (Figures 36A,C,D and 38A–C) than that of the female (Figure 35A,C,D). Additionally, it should be noted that their localities are rather distant from each other (northern Borneo vs. East Java). Since there are only two specimens available at the present, they are tentatively considered as conspecific. Therefore, the male specimen is herein identified provisionally as *Tachinoporus basalis*. More material is needed to resolve this issue.

#### 3.2.10. Genus *Tachinoproporus* Cameron, 1928

*Tachinoproporus* Cameron, 1928: 449 [11] (Figures 40–42; Table A1)

Type species: *Tachinoproporus ferrugineus* Cameron, 1928 [11]: 449.

Differential diagnosis: This enigmatic genus can be distinguished from all remaining genera of Vatesini sensu nov. by the following combination of characters: Dorsum matte with micro-reticulation, antenna shorter than pronotal width, devoid of dense and fine recumbent setae in basal four antennomeres, pronotum widest at base, mesoventrite with plate-like keel along midline, elytra weakly elongate, abdomen without macrosetae with two pairs of paratergites, and male aedeagus (internal sac) with developed spines.

Emended diagnosis based on dorsal and lateral views: Body large (at least ca. 4.2 mm long), weakly depressed dorsoventrally (Figure 40A,C–E); surface matte with dense micro-reticulation, less glossy (Figure 41D, E), dorsum lacking microsetae, punctation, or macrosetae; head with arcuate sides, lacking narrowing of neck region; antenna fili-moniliform, not flattened or modified, moderately shorter than maximum width of pronotum, basal four antennomeres devoid of dense and fine recumbent setae (Figure 41B); maxillary palpomere 4 with sides gradually and evenly tapering from middle to apex (Figure 41C); pronotum semicircular, widest at base; elytron short, only weakly elongate (length/width = ca. 1.2×); elytral epipleuron deeply folded inward, resulting in thin, blade-like elytral margins (Figure 41F); mesoventrite with markedly developed, plate-like keel along midline (Figure 40E,F); abdomen strongly tapered posteriorly, with two pairs of paratergites on segments III–VI, lacking macrosetae (except near apex); tergite VIII and sternite VIII in male with only weakly developed lobes; tergite IX with each side forming somewhat pointed lobe, with single macrosetae at apical third on each lobe (Figure 42E); male aedeagus moderately elongate, only very weakly curved; parameres moderately separated from each other in mesial area (Figure 42B); internal sac with conspicuous curved spines (Figure 42B).

Redescription based on dorsal and lateral views: Male. Body (Figure 40A,C–E) large, broadly oval, weakly depressed dorsoventrally, *Coproporus*-like. Dorsum matte because of dense coverage of large micro-reticulation (Figure 41D, E); head, pronotum, and elytra uniformly glabrous.

Head (Figures 40C–E and 41A) moderately deflexed, transversely oval, widest across eyes, gradually narrowed posteriorly; frontoclypeal suture (Figure 41A) complete, very weakly curved; midcranial suture (Figure 41A) feebly developed; neck constriction or narrowing completely absent in postocular areas (Figure 41A); dorsal surface lacking microsetae or ocular seta (Figure 41A,J); ventral surface without infraorbital ridge extending ventrally from posterior margin of eye. Clypeus (Figure 41A) normal, not reflexed upward, rather moderately produced anteriorly. Eyes (Figures 40D,E and 41A) moderate in size, inconspicuous, barely protruding from outline of head. Antennal insertions (Figure 41A,J) partially concealed under frontal shelf-like projections, located near anterior margins of eyes. Antenna (e.g., Figure 41B) comprised of 11 antennomeres, slender, fili-moniliform, not flattened or modified, about 2/3 as long as maximum width of pronotum, and moderately longer than head width; basal four antennomeres glossy, devoid of dense and fine recumbent setae, remaining antennomeres densely covered with whitish microsetae. Maxillary palpus (Figure 41C) four-segmented, elongate, moderate size and length, lacking recumbent microsetae on surface; palpomere 1 (*mp1*) very short; mp2 narrowly elongate, weakly curved, gradually dilated apically, with a few short setae on apex; mp3 elongate, much shorter than mp2, moderately dilated apically, with a few short setae on apex; mp4 narrowly elongate, nearly twice as long as mp3, only slightly narrower than mp3, sides gradually and evenly tapering from middle to apex, with pointed apex. Labial palpus with elongate terminal palpomere.

Pronotum (Figures 40C–E and 41D) semicircular, broadly transverse, widest at base, much larger than head; sides strongly arcuate and anterior margin weakly concave, with angulate anterolateral angles; disc uniformly convex upward, margins without macrosetae, lacking ground microsetae. Scutellum (e.g., Figure 41E) dorsally visible, sub-triangular, weakly transverse, with rounded apex. Elytron (e.g., Figure 41E,F) short, convex upward, slightly longer than pronotum, with posterior margin rounded laterally, oblique near suture; surface smooth, lacking setae. Elytral epipleuron (Figure 41F) deeply folded inward, resulting in thin, blade-like lateral and posterior margins. Wings (Figure 40C) probably brachypterous associated with short elytra. Mesoventrite with markedly developed, plate-like keel along midline (Figure 40E,F).

Legs (e.g., Figures 40C–E and 41G–I) relatively short, slender, with irregularly distributed spines on tibiae. Procoxae conical, large; protibiae (Figures 40C–E and 41G) rod-like, robust, with long apical spurs, much longer than protarsomere 1 (*pt1*); protarsi (Figure 41G) 5-segmented, narrow, short, probably less than half length of protibiae, basal four tarsomeres each short, together slightly longer than protarsomere 5. Mesotibiae (Figures 40C–E and 41H) with spurs at apex, clearly shorter than mesotarsomere 1 (*mst1*); mesotarsi (Figure 41H) 5-segmented, longer than half length of mesotibiae, mst1 much longer than mst2, basal four tarsomeres combined much longer than mst5. Metacoxae transverse, moderate in size; metacoxal ventral lamella developed; metatibiae (Figures 40C–E and 41I) with short spurs at each apex; metatarsi (Figure 41I) 5-segmented, more than half length of metatibiae, basal metatarsomere much shorter than following three segments combined. Claws (Figure 41G,H) simple, lacking conspicuous basal teeth.

Abdomen (Figure 40C–E) triangular, evenly tapering from base to apex; surface sparsely covered with vestigial microsetae, lacking both V-shaped punctures and macrosetae (with macrosetae only segments VII–IX). Abdominal segments III–VI (Figure 40C) each with two pairs of paratergites, moderate in size. Abdominal segments V–VI including paratergites, with weakly produced posterolateral margins. Tergites lacking both pruinose spots and basolateral ridges. Tergite III (Figure 40C) partly visible, strongly transverse. Tergite VII (Figure 40D) simple, with posterior margin truncate. Tergite VIII (Figure 42C) with only weakly developed lobes, bearing single pair of blackish macrosetae, distinctly shorter than maximum length of tergite VIII. Tergite IX (Figure 42E) with each side forming pointed, bearing single macrosetae at apical third on each lobe; ventral side basally fused, not divided by sternite IX at base; dorsal side divided from base to apex, mesial margins diverging from near base. Tergite X (Figure 42E) normal, elongate. Sternite VI with posterior margin truncate, lacking peg-like setae. Sternite VII (Figure 42A) moderately produced posteriorly, surface weakly shallowly concave along midline in posterior half (*slg*), densely covered with posteriorly directed, yellowish short spines (not peg-like setae). Sternite VIII (Figure 42D) with weakly developed lobes, bearing two pairs of long, blackish macrosetae distinctly shorter than maximum length of sternite VIII. Male aedeagus (Figure 42B) with median lobe narrowly elongate, only very weakly curved ventrally. Parameres (Figure 42B, *prm*) simple, not distinctly flattened, very closely appressed to median lobe; moderately separated from each other in mesial area, but contiguous at apex, without setae or minute sensilla on surface. Internal sac of median lobe with numerous conspicuous, well-developed spines (Figure 42B, *dsp*).

Composition: Only the type species is known [11,21].

Remarks: *Tachinoproporus* is an enigmatic monobasic genus described from Malaysian Borneo [11]. As with *Tachinoporus*, there has been no available information for *Tachinoproporus* since the original description [11], making the genus completely enigmatic. Later workers have only cited the genus in a few catalogues (e.g., [21,104]), and *Tachinoproporus* was placed in Tachyporini in the old sense [21]. Here, the genus is unambiguously placed in the newly revised tribe Vatesini sensu nov. based on the general *Coproporus*-like habitus, short antenna, weakly elongate elytra with elytral epipleuron deeply folded inward, and other morphological evidence as described above.

The systematic placement of this genus within Vatesini sensu nov. seems to be firmly convincing in spite of its absence from the phylogenetic analyses here. Nevertheless, *Tachinoproporus* has several notable features that are not usually found in the tribe or even the subfamily. For example, the dorsal surface of the type species is matte because of the presence of large and dense micro-reticulation, whereas the remaining vatesines have strongly glossy bodies, especially on the head, pronotum, and elytra. Another interesting feature is the complete absence of macrosetae on the abdomen (except for the apex), because the members of Vatesini sensu nov. usually have such macrosetae on at least the ventrolateral sides of the abdomen. The most astonishing feature is the presence of a plate-like, markedly developed keel along the midline of the mesoventrite (Figure 40E,F *pkmsv*), as this condition has never been found in the other tachyporines during the course of my study. Even though some tachyporines have a carina on the mesoventrite, they are only weakly raised above and far from the condition seen in *Tachinoproporus*. Last but not least, the structure of the male genitalia is also unique within the subfamily. Indeed, the internal sac of median lobe in the type species has numerous, very strong, conspicuous spines (Figure 42B, *dsp*). This feature is not found in other tachyporines, but is similar to those found in other different subfamilies: *Trichophya* (Trichophyinae) or *Habrocerus* (Habrocerinae). For these reasons, the genus should be treated as valid and not synonymized under either *Cilea* or *Coproporus*.

#### 3.2.11. *Tachinoproporus ferrugineus* Cameron, 1928

*Tachinoproporus ferrugineus* Cameron, 1928: 449 [11] (Figures 40–42; Table A1)

Type material. Syntype, 1 male [maxilla and abdominal terminalia glued on the paper card near the body]: “Para-|type” < printed-bordered yellow round label >, “*Mt. Dulit*|*3500 f.*” < printed on rectangular small white label >, “Tachinoproporus|ferrugineus|Cam.” < handwritten on rectangular small white label >, “M.Cameron.|Bequest|B.M. 1955-147.” < printed on rectangular small white label >, “Lectotypus|Tachinoproporus ♂|ferrugineus|Cam.|des.|W. G. Ullrich 1974|20964 [written vertically]” < handwritten on rectangular red label >, “Tachinoproporus|ferrugineus ♂|Cam.|W. G. Ullrich det.” < handwritten on rectangular small white label > (BMNH). See Figure 40B.

Comments on type material: A total of four specimens, including both sexes, were specified in the original description (Cameron, 1928). However, only the single male specimen described here was found in the BMNH. A lectotype label written by Dr. Wolfgang G. Ullrich is attached with the specimen (Figure 40B), though this designation has not yet been formally published. In the present study, I treat it as one of the syntypes, refraining from assigning it as the lectotype.

The condition of this syntype is relatively good (Figure 40A,C–E), although the abdominal segments beyond VI, left elytron, and left maxilla were originally removed and mounted on the same paper card along with the body. Similarly, the right leg beyond metatibia was originally removed, but re-attached to the body in a reversed orientation (Figure 40A,C–E). The right antenna beyond antennomere 2 is missing.

Type locality: Borneo, Mt. Dulit, 3500 feet [11].

Distribution: Borneo (Sarawak, Malaysia).

Diagnosis: Same as for the genus.

Redescription based on dorsal and lateral views: Male (*n* = 1). Body (Figure 40A,C–E) 4.24 mm long (measured from anterior margin of head to abdominal apex, as preserved, excluding abdominal segment VII). Color (e.g., Figure 40C) uniformly reddish brown except head and posterior half of elytron darker.

Head (Figures 40C–E and 41A) large (0.85 mm long, 1.17 mm wide), moderately transverse (width/length = 1.38×); sides gradually narrowed posteriorly; midcranial suture (Figure 41A, *mcs*) visible anteriorly; surface covered with irregular pattern of micro-reticulation. Antenna (e.g., Figure 41A,B) 1.49 mm long; antennomere 1 (*a1*) elongate, moderately dilated apically, much longer than a2; a2 rather strongly dilated, shorter and narrower than a1; a3 strongly dilated, only slightly shorter than a4, slightly wider than a2; a4 strongly dilated, much shorter than a3, slightly broader than a3; a5 slightly longer than a6, moderately wider than a4; a6–a10 weakly to strongly transverse, gradually broadened toward apex; a11 elongate oval, symmetrical, approximately twice as long as a10, and slightly narrower, with moderately rounded apex. Maxillary palpus (Figure 41C) approximate relative length of each maxillary palpomere from base to apex: 2.7, 22.5, 12.8, 25.0; approximate relative width of each maxillary palpomere from base to apex: 6.3, 9.9, 8.8, 6.9.

Pronotum (Figures 40C–E and 41D) strongly transverse (width/length = 1.79×; 1.07 mm long, 1.92 mm wide), distinctly convex above in cross section, with sinuate posterior margin (Figure 41D); surface covered with irregular pattern of micro-reticulation. Elytron (e.g., Figure 41E,F) small (1.42 mm long, 1.20 mm wide: measured from right elytron), slightly elongate (length/width = 1.18 ×), without setae of any type; lateral margin lacking epipleural gutter (sensu Herman [25]); dorsal surface covered with large, hexagonal micro-reticulation.

Legs (e.g., Figures 40C–E and 41G–I) compact, with 0.97 mm long metatibia

Abdomen (Figure 40C–E) moderately glossy, but covered with transverse, large, wavy micro-reticulation. Tergite VIII (Figure 42C) with two pairs of weakly developed lobes located in mesial area of posterior margin, and lateral pair of very weakly developed projection-like lobes, each presumably bearing a long blackish macroseta which shorter than than maximum length of tergite VIII; inner pair of lobes short, rounded, slightly longer and narrower than lateral lobes, extending slightly beyond level of outer lobes; outer pair of lobes very short, broadly rounded; each lobe with a few short sensory setae along apex. Tergite IX (Figure 42E) with weak protrusion bearing a macroseta laterally at apical third of each pointed lobe. Sternite VIII (Figure 42D) with two pairs of weakly developed lobes flanking mesial area of posterior margin, each bearing a long blackish macroseta, with tiny lateral pair of very weakly developed projection-like lobes, located in between these lobes; inner pair of lobes short, triangularly pointed, somewhat conspicuous, much longer and wider than lateral lobes, strongly extending beyond level of outer lobes; outer pair of lobes very short, inconspicuous, rather narrowly rounded; mesial area relatively widely incised between inner lobes; each lobe with a few short sensory setae along apex. Aedeagus (Figure 42B) with median lobe not slender, rather thick, widest at basal third.

Female (after Cameron [11]). Tergite VIII with two pairs of long lobes, inner pair narrower and longer than outer pair. Sternite VIII with three pairs of long lobes, inner pair broader, separated from each other by deep oval excision, each apex bearing three sensory setae.

Remarks: Although the redescription above is based merely on the single male syntype from the dorsal and lateral views, I could successfully extract both generic and species characters for males. According to the original description [11], the abdominal segment VIII (both tergite and sternite) in the female possesses a few pairs of long lobes (tergite VIII with two pairs, while sternite VIII with three pairs). The inner pair of lobes of sternite VIII is broader than those of the outer pairs, and they are widely separated from each other, each having three spines at the apex (see Cameron [11]).

#### 3.2.12. Tribe Deropini Smetana, 1983

Deropini Smetana, 1983: 272 [23] (Figures 2–5 and 43–47; Table A1)

Type genus: *Derops* Sharp, 1889 [105]: 418.

Differential diagnosis: Deropini differs from all other tribes of Tachyporinae sensu nov. based on the following combination of characters: body not at all limuloid, dorsum densely finely pubescent, antennae and legs very long, head with slight but distinct neck well behind eyes, lacking midcranial suture, pronotum strongly constricted in posterior half, pronotal hypomeron not strongly inflexed and visible in lateral view, and male sternite VII with dozens of peg-like setae along posterior emargination (modified after Newton et al. [16]).

**Figure 43:**
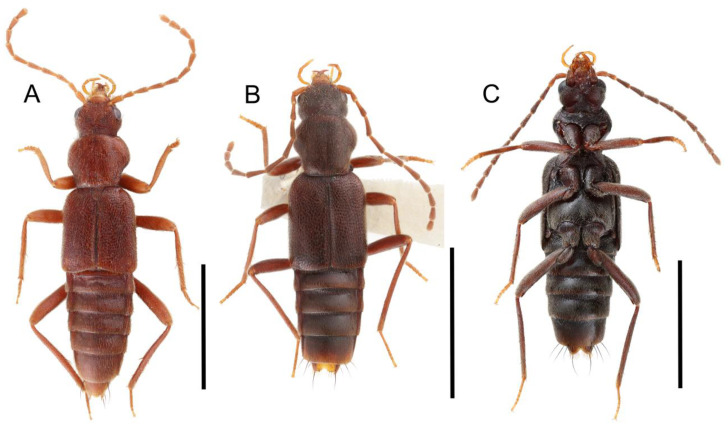
Habitus photographs of Deropini (*Derops*). (**A**) *Derops divalis* (Sanderson), dorsal view. (**B**) *Derops longicornis* Sharp, dorsal view. (**C**) *Derops yaeyamanus* Kishimoto, ventral view. Scale bars: 2.0 mm.

**Figure 44:**
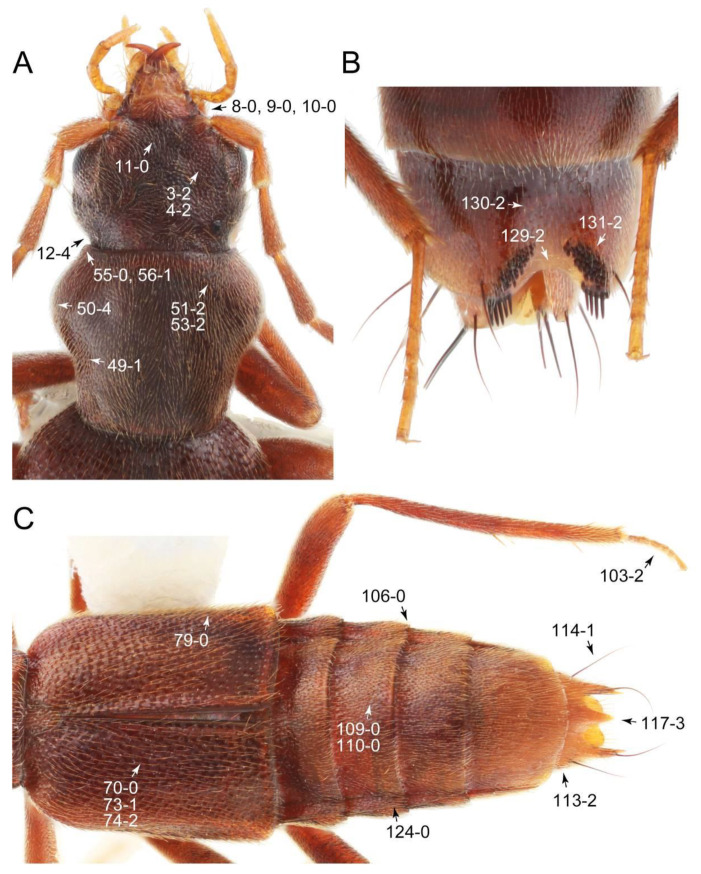
Photographs of body parts of Deropini (*Derops*), enlarged. (**A**) head and pronotum of *Derops longicornis*, dorsal view. (**B**) male sternite VII of *Derops longicornis*, ventral view. (**C**) elytra, hindleg, and abdomen of *Derops divalis*, female, dorsal view. Characters and character states (format X-X) are indicated on each figure.

**Figure 45:**
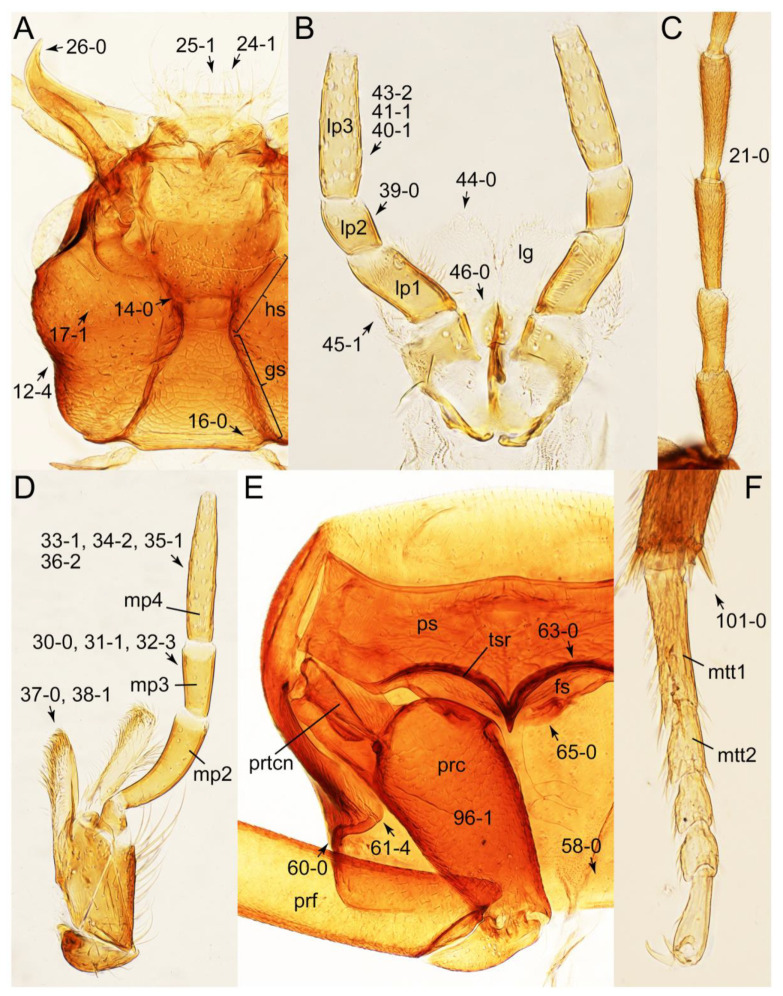
Photographs of body parts of Deropini (*Derops*), enlarged. (**A**) head and mouthparts of *Derops divalis*, ventral view. (**B**) labium of *Derops longicornis*, ventral view. (**C**) basal half of right antenna of *Derops longicornis*, ventral view. (**D**) left maxilla of *Derops longicornis*, ventral view. (**E**) prothorax and right procoxa of *Derops longicornis*, ventral view. (**F**) right metatarsus and metatibial spines of *Derops divalis*, dorsal view. Abbreviations: fs, furcasternum; gs, gular suture; hs, hypostomal suture; lg, ligula; lp1–3, labial palpomere 1–3; mp2–4, maxillary palpomere 2–4; mtt1–2, metatarsomere 1–2; prc, procoxa; prf, profemur; prtcn, protrochantin; ps, prosternum; tsr, transverse sternacoxal ridge of prosternum. Characters and character states (format X-X) are indicated on each figure.

Emended diagnosis: Body slender, not tachyporine-like (Figure 43 vs. e.g., Figures 14 and 48), dorsal surface densely finely pubescent (Figure 44: 4-2, 53-2, 74-2); antennae and legs very long (Figure 43), but with short metatarsi (Figure 44C: 103-2); head with slight but distinct neck well behind eyes (Figures 8A and 44A: 12-4), lacking midcranial suture; maxillary palpomere 3 glabrous (Figure 45D: 32-3); pronotum constricted basally, its base distinctly narrower than humeral width of elytra (Figure 44A: 49-1); pronotal hypomeron not strongly inflexed, completely visible in lateral view, with very large, triangular postcoxal process (Figure 45E: 61-4); male tergite IX abruptly divergent and separated in basal 1/3 (Figure 47F: 120-1), each apex forming single lobe (Figure 47F: 122-2), usually with only single to several macrosetae (Figures 47F: 123-2); sternite VII in both sexes with broad, semicircular protrusion along basomedial margin (Figure 47A: 128-1); male sternite VII emarginate and concave along posteromedial margin, with dozens of peg-like setae on each side of emargination (Figure 47A: 131-2); male aedeagus with flattened plate-like parameres (Figure 47F: 145-1). Modified after Smetana [23] and Newton et al. [16].

**Figure 46:**
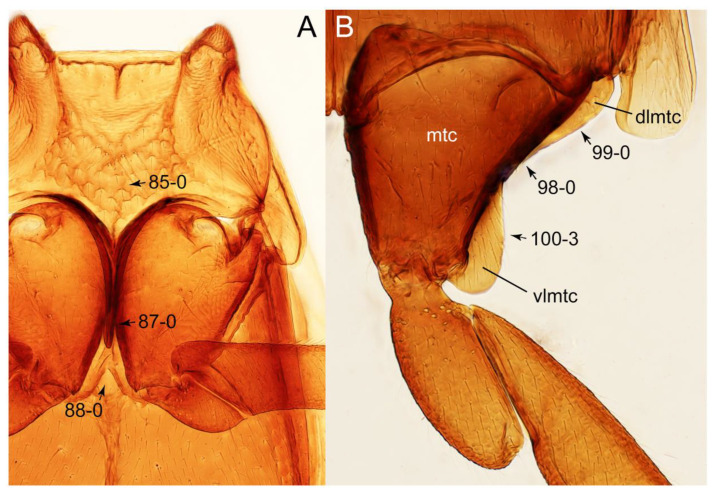
Photographs of body parts of Deropini (*Derops*), enlarged. (**A**) mesothorax and metathorax of *Derops longicornis*, ventral view. (**B**) left metacoxa of *Derops longicornis*, ventral view. Abbreviations: dlmtc, dorsal lamella of metacoxa; mtc, metacoxa; vlmtc, ventral lamella of metacoxa. Characters and character states (format X-X) are indicated on each figure.

**Figure 47:**
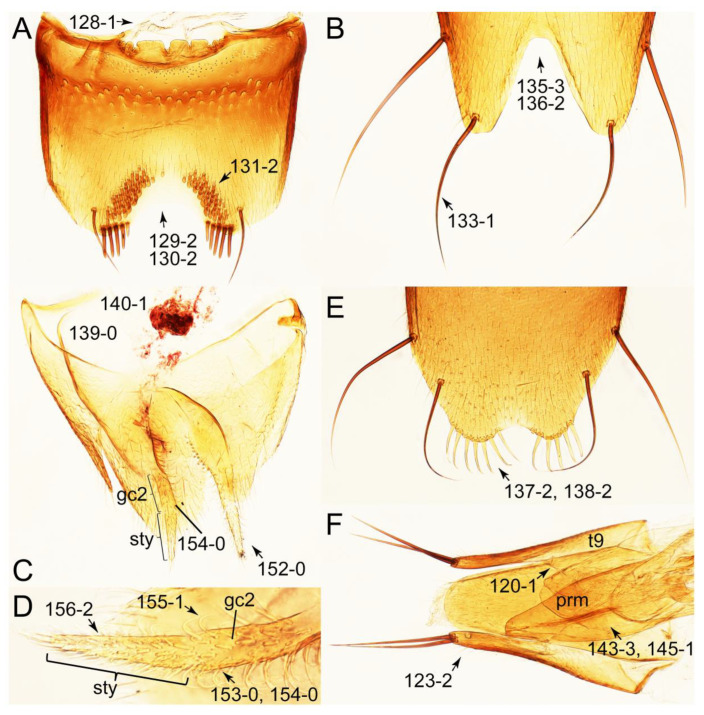
Photographs of body parts of Deropini (*Derops*), enlarged: *Derops longicornis*. (**A**) male sternite VII, ventral view. (**B**) male sternite VIII, ventral view. (**C**) female genital segments, dorsal view. (**D**) female gonocoxite II and gonostylus, dorsal view. (**E**) female sternite VIII, ventral view. (**F**) male tergites IX and X with aedeagus, ventral view. Abbreviations: gc2, gonocoxite II; prm, paramere; sty, gonostylus; t9, tergite IX. Characters and character states (format X-X) are indicated on each figure.

Description: Body (Figure 43) medium, slender, not typical tachyporine-like; surface uniformly covered with dense and fine setae (Figure 44: 3-2, 51-2, 73-1), with relatively conspicuous punctation (Figure 44: 4-2, 53-2, 74-2). Head (Figure 44A) lacking ocular seta and midcranial suture (Figure 44A: 11-0); postocular areas with neck-like narrowing in middle, well behind eyes (Figures 44A and 45A: 12-4). Antenna (Figures 43 and 45C) very long, filiform, without clear pattern of dense and fine recumbent pubescence (Figure 45C: 21-0). Maxillary palpus (Figure 45D: 32-3) elongate, glabrous; palpomere 3 widest at apex (Figure 45D: 30-0), distinctly shorter than palpomere 2 (≤0.7 ×; Figure 45D: 31-1); palpomere 4 sub-cylindrical, only weakly narrowed anteriorly, widest in middle (Figure 45D: 33-1), much longer than penultimate palpomere (Figure 45D: 34-2), with conspicuous scattered pores (Figure 45D: 36-2). Maxillary lacinia (Figure 45D) without unarticulated large, long spine (Figure 45D: 37-0), with numerous short teeth or spines (Figure 45D: 38-1). Labial palpi (Figure 45B: 46-0) narrowly separated at base, much shorter than length of longest labial palpomere; palpomere 3 elongate, widest in middle, not at base (Figure 45B: 40-1), as wide as penultimate palpomere (Figure 45B: 41-1), with conspicuous pores (Figure 45B: 43-2). Mentum weakly to moderately transverse, with truncate anterior margin. Pronotum (Figure 44A) constricted in posterior half, its base distinctly narrower than humeral width of elytra (Figure 44A: 49-1), without stout setae on dorsum; anterior margin truncate (Figure 44A: 55-0), with angulate anterior angles (Figure 44A: 56-1); posterior margin without conspicuous clusters of blackish pores (Figure 45E: 58-0). Pronotal hypomeron narrow, not widened posteriorly in basal third (Figure 45E: 60-0), not strongly inflexed, completely visible in lateral view, with very large, projecting triangular postcoxal process (Figure 45E: 61-4). Prosternum (Figure 45E) with sternacoxal ridge (*tsr*) moderately distant from anterior margin of prosternum in medio-lateral areas (Figure 45E: 63-0), with very short prosternal process. Elytron (Figures 43A,B and 44C) elongate, rather long, without macrosetae on dorsum and epipleural gutter along outer margin (Figure 44C: 79-0); lateral side with epipleural ridge or keel, which is only feebly folded inward. Mesoventrite (Figure 46A: 85-0) without longitudinal median carina. Metendosternite with anterior arms thin and inconspicuous. Legs (Figures 43, 44C, 45E,F and 46) very long, slender, with less developed scattered spines on tibiae; procoxae (Figure 45E: 96-1) medium, shorter than profemora; metacoxae (Figure 46B: 98-0) small in each outer lateral half; metatibiae with short metatibial spurs at apex, shorter than 2/5 of metatarsomere 1 (Figure 45F: 101-0); metatarsi (Figure 44C: 103-2) short, less than half length of metatibia. Abdomen (Figures 43 and 44C) tapering somewhat weakly posteriorly, with single pair of paratergites on segments III–VII (Figure 44C: 124-0). First three visible terga with transverse deep impressions (Figure 44C). Tergites III–VII (Figures 43A,B and 44C) without macrosetae (Figure 44C: 109-0), each rather weakly transverse (Figure 44C: 111-0). Tergite VIII (Figure 44C: 114-1) with only a few to several distinct macrosetae; posterior margin in male simple, unmodified, that of female deeply emarginate medially (Asian species) or modified into lobes (North American species; Figure 44C: 117-3). Male tergite IX (Figure 47F) seemingly contiguous and not fused at base of ventral side; dorsal side abruptly divided apically from near base (Figure 47F: 120-1), making each side elongate; apical areas each with at most several long macrosetae, restricted to apices (Figure 47F: 123-2). Sternite III with longitudinal median carina. Sternite VII in both sexes with semicircular protrusion along basomedial margin (Figure 47A: 128-1; see Zhao & Li [106]: Figure 2A); posteromedial margin in male emarginate and concave (Figures 44B and 47A: 129-2, 130-2), with dozens of characteristically arranged with peg-like setae (Figures 44B and 47A: 131-2). Sternite VIII (Figure 47B,E: 133-1) with only a few to several distinct macrosetae; posterior margin in male (Figure 47B) deeply incised or very strongly emarginate medially (Figure 47B: 135-3, 136-2), that of female (Figure 47E) nearly rounded, with slight emargination flanked by a pair of feebly developed projections (Figure 47E: 137-2), each apex with row of sensory setae (Figure 47E: 138-2). Male aedeagus (Figure 47F: 145-1) elongate, rather flattened, with plate-like, flattened parameres longitudinally contiguous (Figure 47F: 143-3). Female genitalia (Figure 47C) with gonocoxite II slender, narrowly elongate (Figure 47C: 153-0), bearing only curved setae (Figure 47D: 155-1).

Composition: One genus, with 20 species. See Table A1 for overview and distributions.

- *Derops* Sharp, 1889 [105]: 418

Type species: *Derops longicornis* Sharp, 1889 [105]: 418.

Fossils: No fossil Deropini are currently known.

Remarks: Deropini is the only tribe without any taxonomic change in this study. This monogeneric tachyporine tribe is significant because of the distinct, un-tachyporine habitus (Figure 43). The body shape is not at all limuloid but slender and narrowly elongate. The shape of the pronotum is also unique within the subfamily because it is strongly constricted in the basal half (Figure 44A). Due to the odd-looking habitus, *Derops*, the sole generic member of this tribe, had been placed in various subfamilies such as Oxytelinae or Phloeocharinae [16,23]. However, it is currently firmly placed within Tachyporinae in the old sense based on both morphological and molecular studies [6,7,23]. One of the most important characters which connects Deropini with Tachinusini stat. rev., sensu nov., within Tachyporinae sensu nov. is the presence of peg-like setae in the male sternite VII and genital features [23,43] (Figures 44B and 47B,F).

*Derops* has a highly disjunct distribution, most being recorded from East and Southeast Asia (Russian Far East, Korea, Japan, China, Vietnam, and India), but a single species occurs in the eastern part of USA [16,106]. Synonymy of the North American genus *Rimulincola* Sanderson, 1947 [107] under *Derops* is maintained here. *Derops* species are found in wet leaf litter and decayed vegetation in deep rock crevices or along streams, and occasionally in caves [16].

#### 3.2.13. Tribe Tachinusini Fleming, 1821 stat. rev., sensu nov.

Tachinusini Fleming, 1821: 49 [45] (Figures 1D, 2–5 and Figure 48, Figure 49, Figure 50, Figure 51, Figure 52, Figure 53, Figure 54, Figure 55, Figure 56, Figure 57, Figure 58, Figure 59, Figure 60 and Figure 61; Table A1)

=Megarthropsini Cameron, 1919 [101]: 231 syn. nov. (type genus: *Megarthropsis* Cameron, 1919 [101]: 231).

Type genus: *Tachinus* Gravenhorst, 1802 [41]: 134.

Differential diagnosis: Tachinusini stat. rev., sensu nov. differs from all other tribes of Tachyporinae sensu nov. based on the following combination of characters: forebody usually glabrous, head usually with neck-like narrowing just behind eyes, sometimes bearing ocular setae, antenna devoid of dense and fine recumbent pubescence in basal two or four antennomeres, maxillary palpomere 4 longer than penultimate palpomere, pronotal hypomeron with long postcoxal process, abdomen rarely with blackish macrosetae dorsally, and male sternite VII usually with a few to dozens of peg-like (or stout) setae along posterior margin.

Diagnosis: Body surface usually glabrous (Figures 50 and 51: 4-0, 53-0, 74-0); head with neck-like narrowing just behind eyes in most taxa (e.g., Figures 50, 51B,D and 58A: 12-3), occasionally much weaker (Figure 51A: 12-0); antenna devoid of dense and fine recumbent pubescence in basal two or four antennomeres (Figure 59C: 21-3); maxillary palpomere 3 distinctly shorter than palpomere 2, usually lacking ground setae (e.g., Figures 53C and 59A: 31-1, 32-2); maxillary palpomere 4 widest in between basal 1/6 to middle, longer than penultimate palpomere (Figures 53C and 59A: 33-1, 34-2); pronotal hypomeron with fully developed postcoxal process (Figures 54G and 59D,E: 61-5); abdomen without blackish macrosetae dorsally (except abdominal terminalia), with exceptions of *Nitidotachinus* and *Leucotachinus*; male tergite VIII with lobe-like modifications (Figure 55C: 116-1); male tergite IX abruptly divergent and separated dorsally in basal 1/3 (Figure 55E,F: 120-1), each apex usually forming single lobe (Figures 55G and 61E: 122-2), usually with only single to several macrosetae (Figures 55E,F and 61E: 123-2), except a few genera; male sternite VII usually emarginate and concave along posteromedial margin (Figures 52D, 55A and 61A: 129-1, 129-2, 130-1, 130-2), with variously arranged short and stout setae or peg-like setae (Figures 52D, 55A and 61A: 131-1, 131-2); male sternite VIII with posterior margin deeply emarginate or incised medially, usually associated with lobes (Figures 55B and 61B: 135-5, 136-1, 136-2); female sternite VIII with lobes of most taxa (Figures 55D and 61C: 137-3), with more than three sensory setae at apex of each lobe (Figures 55D and 61C: 138-4); male aedeagus with flattened plate-like parameres in most taxa (Figures 56B and 61F: 145-1).

**Figure 48:**
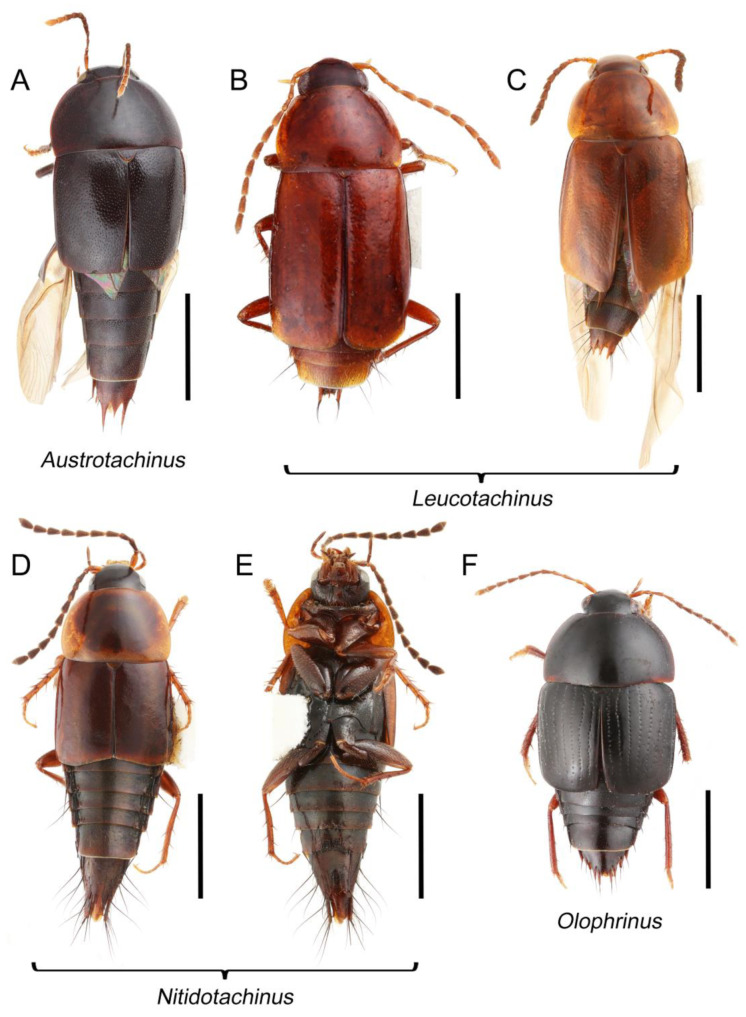
Habitus photographs of Tachinusini. (**A**) *Austrotachinus fuscipes*, dorsal view. (**B**) *Leucotachinus luteonitens* (Fairmaire & Germain) from Chile, dorsal view. (**C**) *Leucotachinus novitius* (Blackburn) from Australia, dorsal view. (**D**) *Nitidotachinus tachyporoides* (Horn), dorsal view. (**E**) *Nitidotachinus tachyporoides*, ventral view. (**F**) *Olophrinus* cf. *philippinus* Campbell, dorsal view. Scale bars: 2.0 mm (**A**,**F**); 1.5 mm (**B**–**E**).

**Figure 49:**
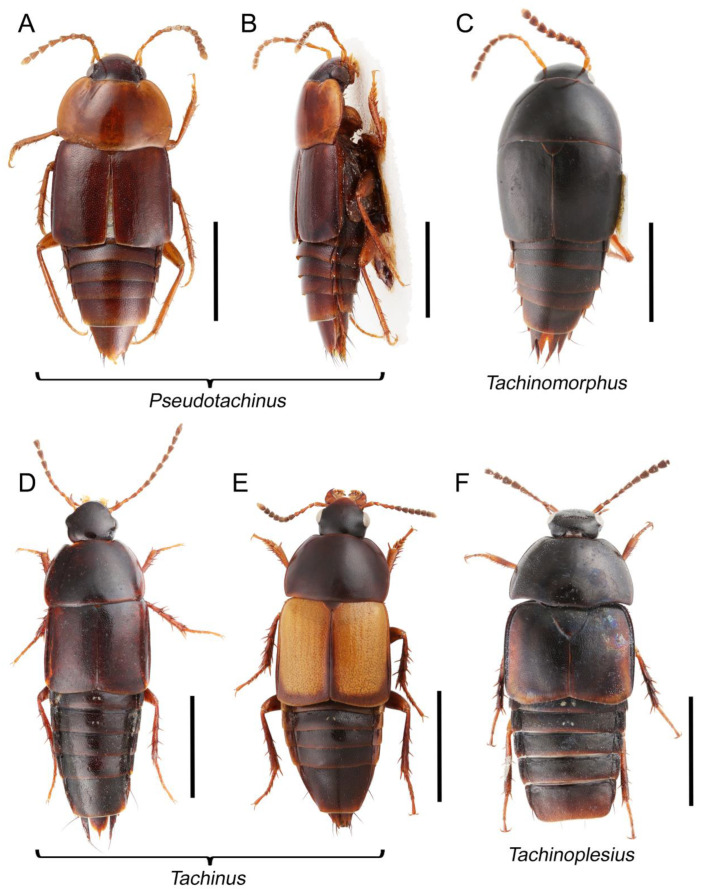
Habitus photographs of Tachinusini. (**A**) *Pseudotachinus besucheti* Schülke, dorsal view. (**B**) *Pseudotachinus besucheti*, dorsolateral view. (**C**) *Tachinomorphus grossulus* (LeConte), dorsal view. (**D**) *Tachinus rufipes* (Linnaeus), dorsal view. (**E**) *Tachinus fimbriatus* Gravenhorst, dorsal view. (F) *Tachinoplesius latipennis* Schülke, dorsal view. Scale bars: 2.0 mm (**A**,**B**,**D**,**F**); 1.5 mm (**C**), 3.0 mm (**E**).

**Figure 50:**
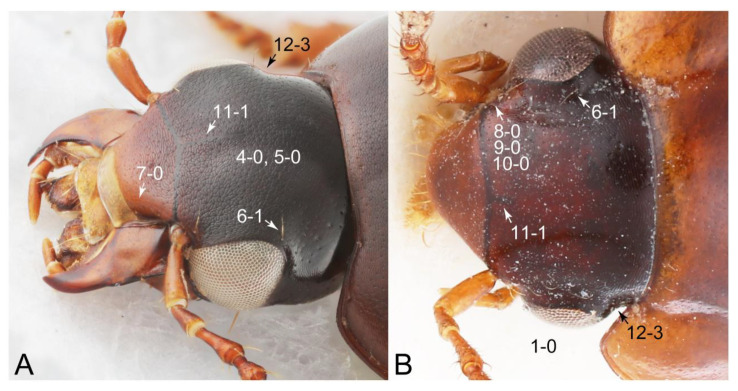
Photographs of body parts of Tachinusini, head, enlarged. (**A**) *Tachinus fumipennis*, dorsolateral view. (**B**) *Pseudotachinus besucheti*, dorsal view. Characters and character states (format X-X) are indicated on each figure.

**Figure 51:**
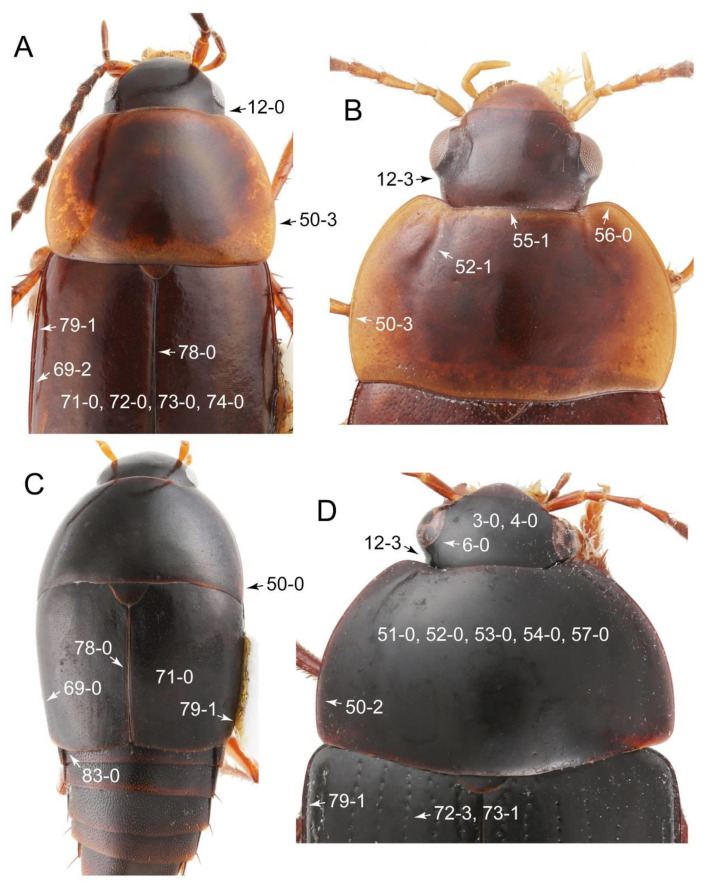
Photographs of body parts of Tachinusini, enlarged, dorsal view. (**A**) forebody of *Nitidotachinus tachyporoides*. (**B**) head and pronotum of *Pseudotachinus besucheti*. (**C**) forebody and part of abdomen of *Tachinomorphus grossulus*. (**D**) forebody of *Olophrinus* cf. *philippinus*. Characters and character states (format X-X) are indicated on each figure.

**Figure 52:**
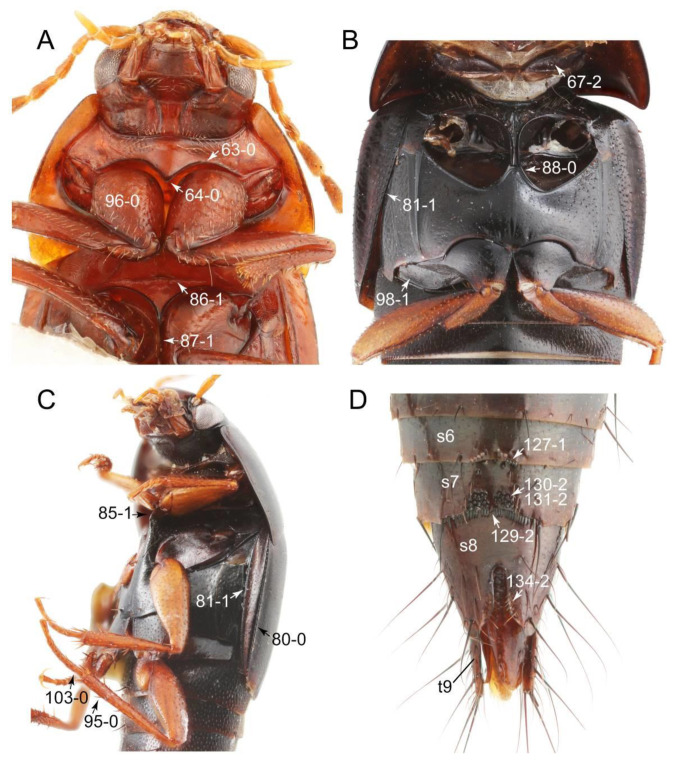
Photographs of body parts of Tachinusini, enlarged. (**A**) forebody of *Leucotachinus luteonitens*, ventral view. (**B**) mesothorax and metathorax of *Tachinoplesius latipennis*, ventral view. (**C**) forebody of *Tachinomorphus grossulus*, ventrolateral view. (**D**) male abdomen of *Nitidotachinus tachyporoides*, ventral view. Abbreviations: s6–8, sternite s6–8; t9, tergite IX. Characters and character states (format X-X) are indicated on each figure.

**Figure 53:**
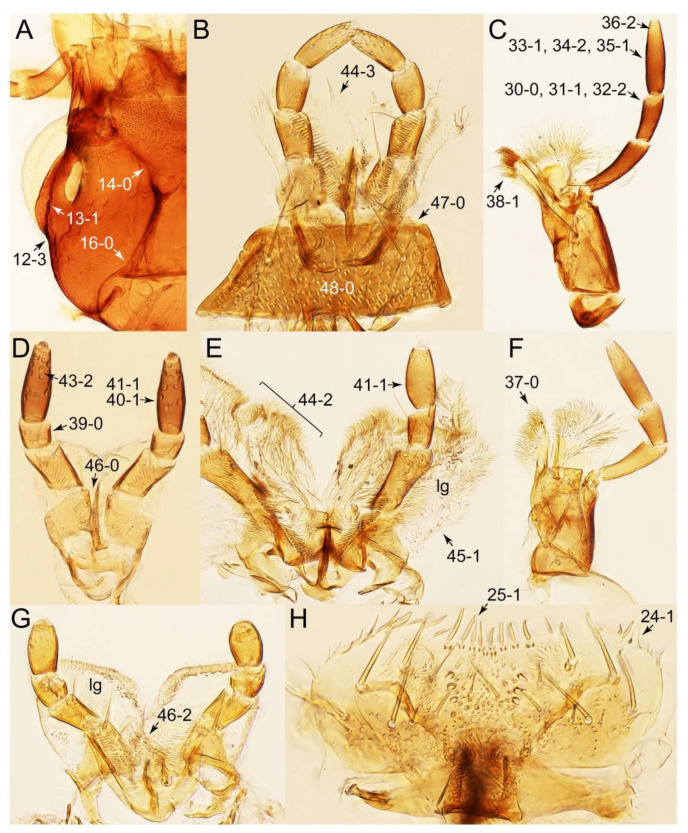
Photographs of body parts of Tachinusini, enlarged. (**A**) head of *Leucotachinus luteonitens*, ventral view. (**B**) labium and mentum of *Leucotachinus luteonitens*, dorsal view. (**C**) left maxilla of *Olophrinus* cf. *philippinus*, ventral view. (**D**) labium of *Nitidotachinus tachyporoides*, ventral view. (**E**) labium of *Olophrinus* cf. *philippinus*, ventral view. (**F**) left maxilla of *Tachinus rufipes*, ventral view. (**G**) labium of *Tachinomorphus grossulus*, ventral view. (**H**) labrum of *L*. *luteonitens*, dorsal view. Abbreviation: lg, ligula. Characters and character states (format X-X) are indicated on each figure.

**Figure 54:**
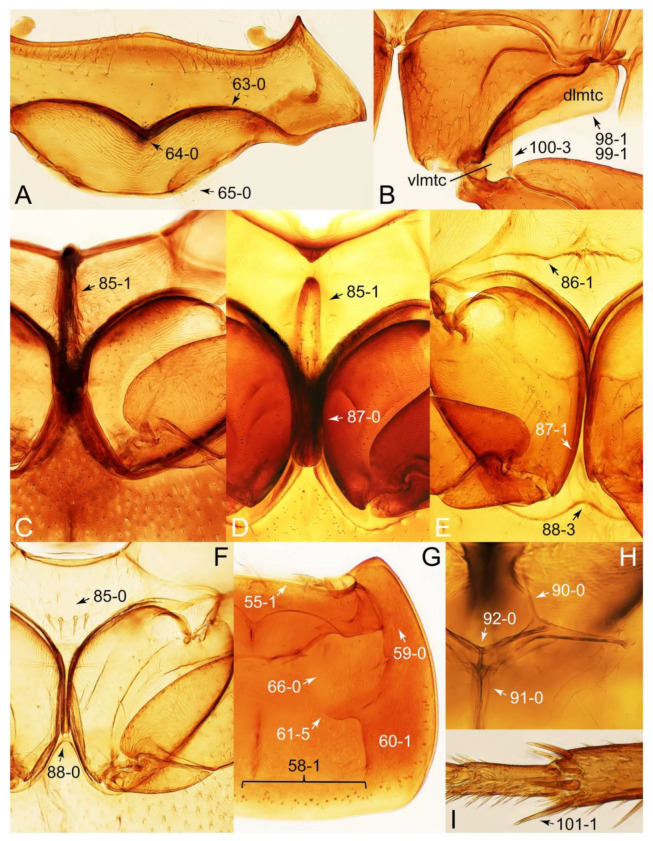
Photographs of body parts of Tachinusini, enlarged. (**A**) prosternum of *Leucotachinus luteonitens*, ventral view. (**B**) left metacoxa of *Leucotachinus luteonitens*, ventral view. (**C**) intermesocoxal processes of *Tachinomorphus grossulus*, ventral view. (**D**) intermesocoxal processes of *Olophrinus* cf. *philippinus*, ventral view. (**E**) intermesocoxal processes of *L*. *luteonitens*, ventral view. (**F**) intermesocoxal processes of *Tachinus fumipennis* Say, ventral view. (**G**) prosternum and pronotum of *Tachinus rufipes*, ventral view. (**H**) metendosternite of *Nitidotachinus scrutator* (Gemminger & Harold), dorsal view. I, metatarsomere 1 and metatibial spines of *Tachinomorphus grossulus*. Abbreviations: dlmtc, dorsal lamella of metacoxa; vlmtc, ventral lamella of metacoxa. Characters and character states (format X-X) are indicated on each figure.

**Figure 55:**
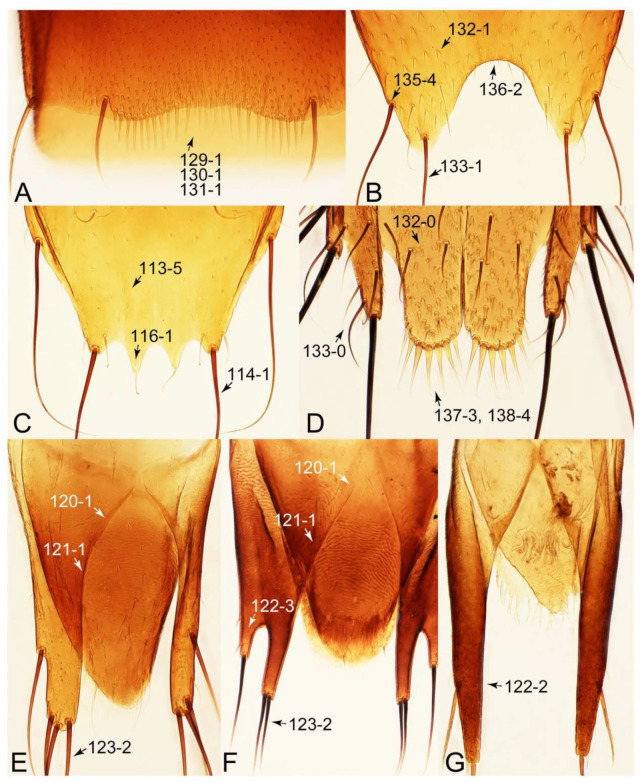
Photographs of body parts of Tachinusini, enlarged. (**A**) male sternite VII of *Tachinomorphus grossulus*, ventral view. (**B**) male sternite VIII of *Leucotachinus luteonitens*, ventral view. (**C**) male tergite VIII of *Leucotachinus luteonitens*, dorsal view. (**D**) female sternite VIII of *Nitidotachinus tachyporoides*, ventral view. (**E**) male tergite IX of *Leucotachinus luteonitens*, dorsolateral view. (**F**) male tergite IX of *Olophrinus* cf. *philippinus*, dorsolateral view. (**G**) male tergite IX of *Tachinomorphus grossulus*, dorsal view. Characters and character states (format X-X) are indicated on each figure.

**Figure 56:**
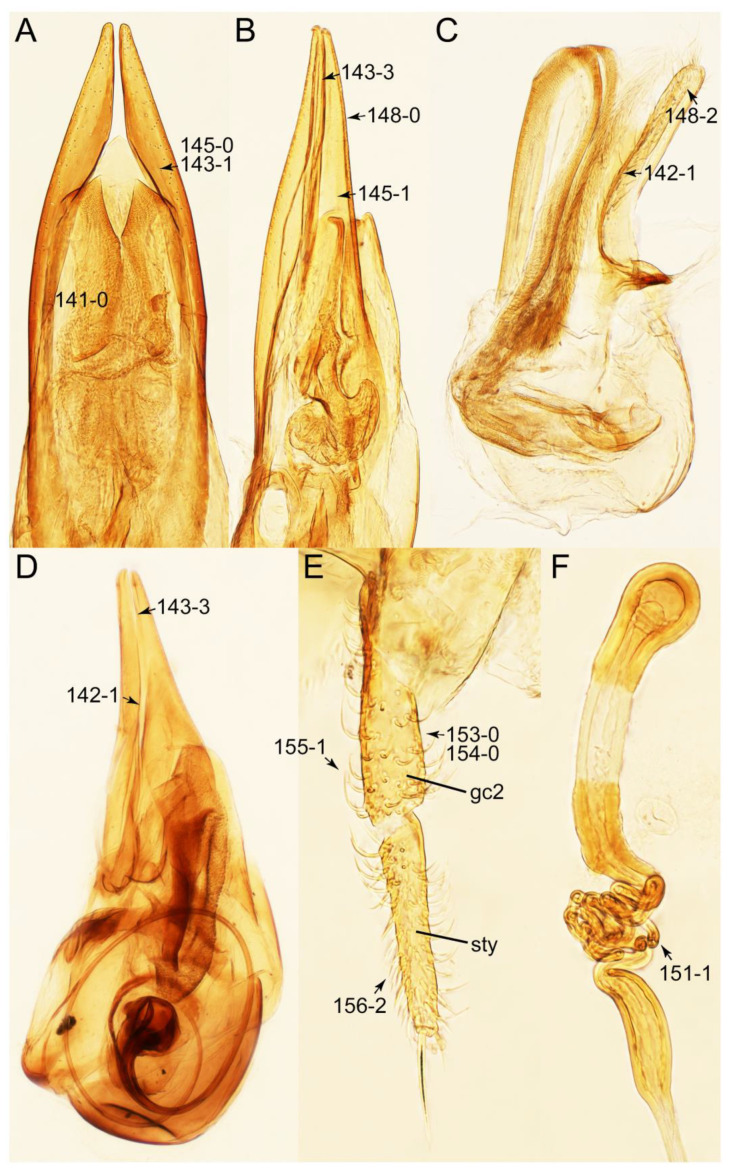
Photographs of body parts of Tachinusini, enlarged. (**A**) male aedeagus of *Leucotachinus luteonitens*, ventral view. (**B**) male aedeagus of *Nitidotachinus tachyporoides*, ventrolateral view. (**C**) male aedeagus of *Tachinomorphus grossulus*, lateral view. (**D**) male aedeagus of *Olophrinus* cf. *philippinus*, lateral view. (**E**) female gonocoxites and gonostylus of *Tachinus frigidus* Erichson, dorsal view. (**F**) female spermatheca of *Tachinus frigidus*. Characters and character states (format X-X) are indicated on each figure.

**Figure 57:**
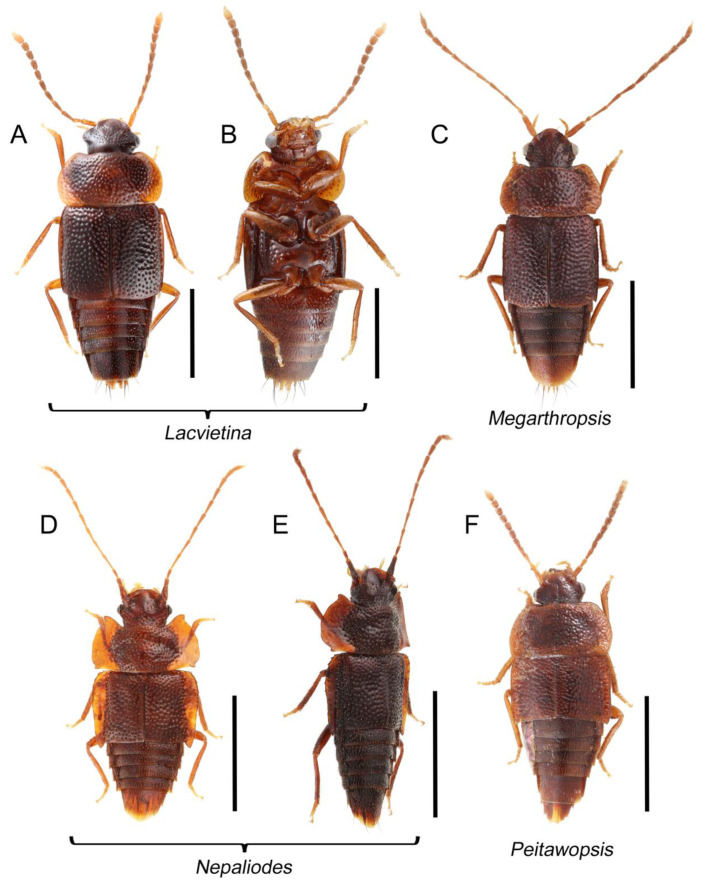
Habitus photographs of Tachinusini: members of the former Megarthropsini. (**A**) *Lacvietina takashii* (Hayashi), dorsal view. (**B**) *Lacvietina takashii*, ventral view. (**C**) *Megarthropsis decorata*, dorsal view. (**D**) *Nepaliodes solangelae* Herman, dorsal view. (**E**) *Nepaliodes solangelae*, dorsolateral view. (**F**) *Peitawopsis monticola* Smetana, dorsal view. Scale bars: 1.0 mm (**A**,**B**); 1.5 mm (**C**,**F**); 2.0 mm (**D**,**E**).

**Figure 58:**
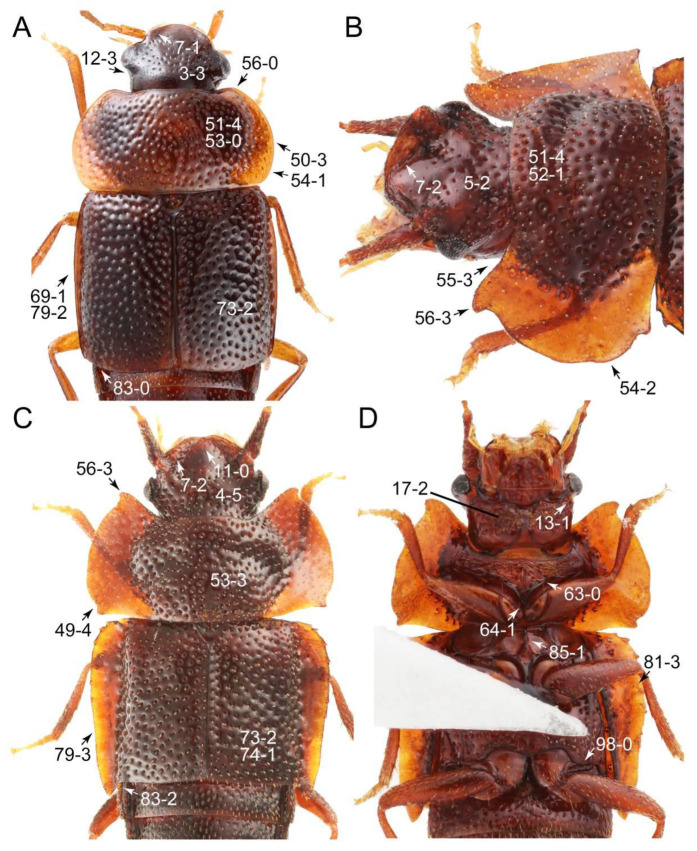
Photographs of body parts of Tachinusini: members of the former Megarthropsini, enlarged. (**A**) forebody of *Lacvietina takashii*, dorsal view. (**B**) head and pronotum of *Nepaliodes solangelae*, dorsolateral view. (**C**) forebody of *Nepaliodes solangelae*, dorsal view. (**D**) forebody of *Nepaliodes solangelae*, ventral view. Characters and character states (format X-X) are indicated on each figure.

**Figure 59:**
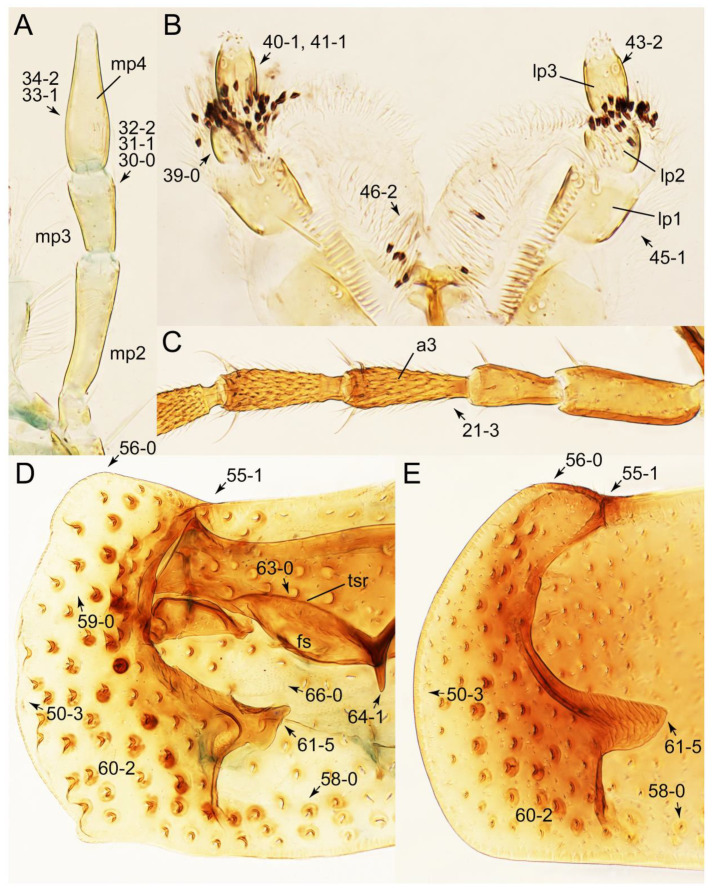
Photographs of body parts of Tachinusini: members of the former Megarthropsini, enlarged. (**A**) left maxillary palpus of *Megarthropsis decorata*, ventral view. (**B**) labium of *Lacvietina takashii*, ventral view. (**C**) basal half of right antenna of *Lacvietina takashii*, dorsal view. (**D**) prosternum and pronotum of *Megarthropsis decorata*, ventral view. (**E**) pronotum of *Lacvietina takashii*, ventral view. Abbreviations: a3, antennomere 3; fs, furcasternum; lp1–3, labial palpomere 1–3; mp2–4, maxillary palpomere 2–4; tsr, transverse sternacoxal ridge of prosternum. Characters and character states (format X-X) are indicated on each figure.

**Figure 60:**
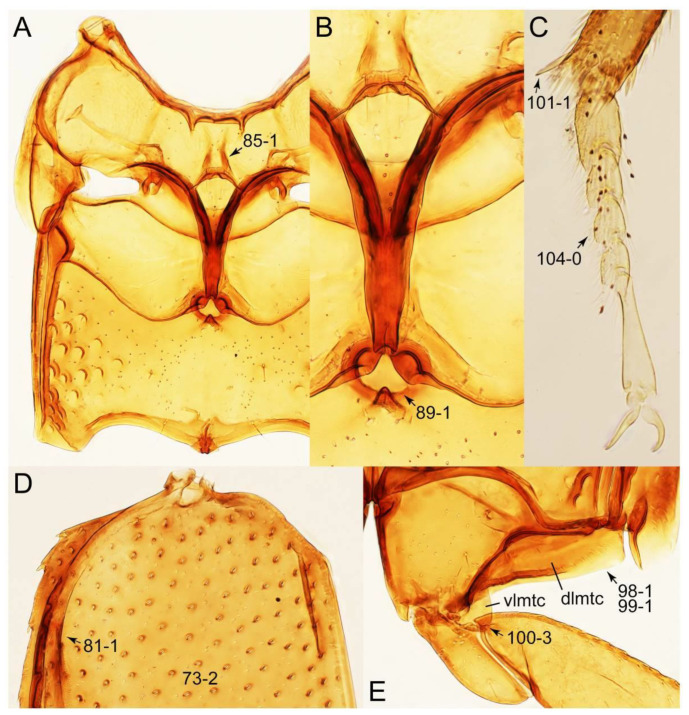
Photographs of body parts of Tachinusini: members of the former Megarthropsini, enlarged. (**A**) mesothorax and metathorax of *Lacvietina takashii*, ventral view. (**B**) intermesocoxal processes of *Lacvietina takashii*, ventral view. (**C**) right metatarsus and metatibial spines of *Lacvietina takashii*, dorsolateral view. (**D**) left elytron of *Lacvietina takashii*, dorsal view. (**E**) left metacoxa of *Lacvietina takashii*, ventral view. Abbreviations: dlmtc, dorsal lamella of metacoxa; vlmtc, ventral lamella of metacoxa. Characters and character states (format X-X) are indicated on each figure.

**Figure 61:**
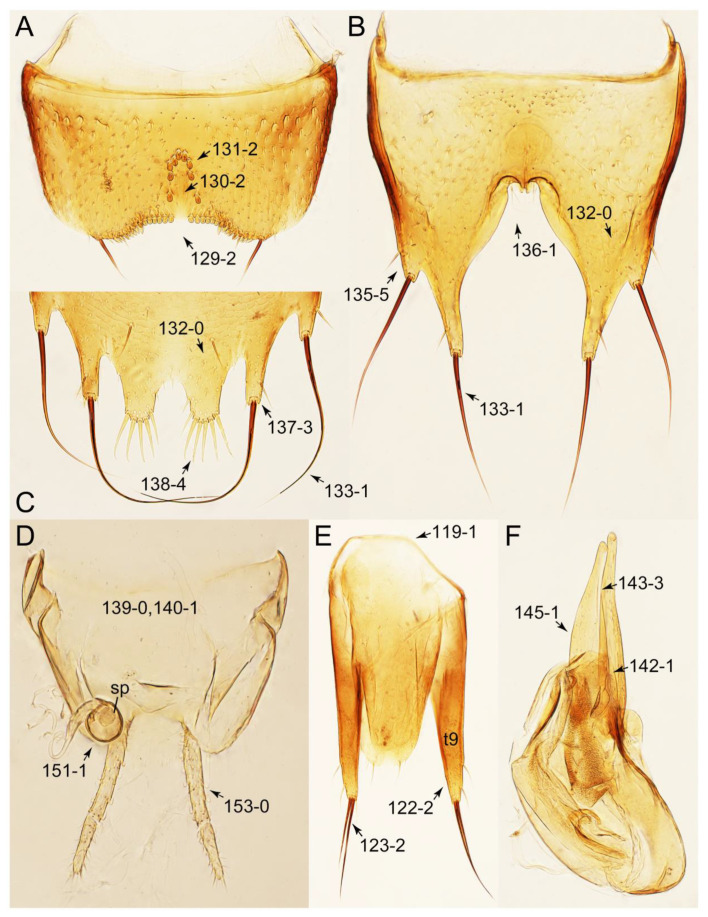
Photographs of body parts of Tachinusini: members of the former Megarthropsini, enlarged. (**A**) male sternite VII of *Lacvietina takashii*, ventral view. (**B**) male sternite VIII of *Lacvietina takashii*, ventral view. (**C**) female sternite VIII of *Lacvietina takashii*, ventral view. (**D**) female genital segments of *Lacvietina takashii*, ventral view. (**E**) male tergite IX and sternite IX of *Lacvietina takashii*, dorsal view. (**F**) male aedeagus of *Lacvietina takashii*, ventrolateral view. Abbreviations: sp, spermatheca; t9, tergite IX. Characters and character states (format X-X) are indicated on each figure.

Description: Body (Figures 48, 49 and 57) medium to large for tachyporines, sub-limuloid; surface generally glabrous (Figures 50 and 51: 4-0, 53-0, 74-0), occasionally covered with inconspicuous microsetae or short, modified setae (Figure 58B,C: 4-5, 53-3); punctation usually absent or inconspicuous (e.g., Figure 51: 3-0, 51-0, 73-0), but sometimes deep, pit-like (e.g., Figure 58A: 51-4, 73-2). Head (Figures 50, 51, 52A, 53A and 58) lacking ocular seta (see Figure 51D: 6-0) or with a short, thin one (Figure 50: 6-1); postocular areas usually with neck-like narrowing, just behind eyes (e.g., Figures 50, 51B,D and 58A: 12-3), but sometimes not (*Nitidotachinus*; Figure 51A: 12-0) or only slightly narrowed (12-1); clypeus in some taxa reflexed upward (Figure 58A–C: 7-1, 7-2). Antenna (Figures 48, 49, 57 and 59C) long to very long, filiform to fili-moniliform, with clear pattern of dense and fine recumbent pubescence, i.e., lacking such setae on basal two or four antennomeres (Figure 59C: 21-3). Maxillary palpus (Figures 53C,F and 59A: 32-2) elongate, generally lacking ground setae in most cases, except for limited stout setae; palpomere 3 widest at or near apex, distinctly shorter than palpomere 2 (≤0.7×; Figures 53C and 59A: 31-1); palpomere 4 widest in between basal 1/6 to middle (Figures 53C and 59A: 33-1), longer than penultimate palpomere (Figures 53C and 59A: 34-2), usually with conspicuous or inconspicuous scattered pores (Figure 53C: 36-2). Maxillary lacinia (Figure 53C,F) without unarticulated large, long spine (Figure 53F: 37-0), with numerous short teeth or spines in most taxa (Figure 53C: 38-1). Labial palpi (Figure 53B,D,E,G) generally widely separated at base (Figure 53B,D,E,G and 59B: 46-2), but narrowly separated in *Nitidotachinus* (Figure 53D: 46-0); palpomere 3 elongate, usually widest in middle or near base, but not at base (Figure 53D: 40-1), almost same width or only slightly narrower than penultimate palpomere (Figure 53D,E: 41-1), with conspicuous pores in most cases (Figure 53D: 43-2). Mentum (Figure 53B: 47-0, 48-0) weakly to moderately transverse, with truncate anterior margin. Pronotum (Figures 51 and 58) of typical Tachyporinae sensu nov. form except in *Nepaliodes* (Figure 58C: 49-4), usually widest between basal 1/4 to middle (Figures 51A,B and 58A: 50-3) [widest at or near base in *Tachinomorphus* (Figure 51C: 50-0) and *Olophrinus* (Figures 51D: 50-0)]; disc without stout setae (Figure 51D: 53-0); anterior margin weakly to moderately concave in most examples (Figures 51B and 59D,E: 55-1), usually with rounded anterior angles (Figures 51B, 58A and 59D,E: 56-0); posterior margin sometimes sinuate with conspicuous clusters of blackish pores (Figure 54G: 58-1). Pronotal hypomeron (Figures 54G and 59D,E) moderate to wide in basal third (Figures 54G and 59D,E: 60-1, 60-2), strongly inflexed, not visible in lateral view, with fully developed, long postcoxal process (Figures 54G, 59D, E: 61-5). Prosternum (Figures 54A and 59D) with sternacoxal ridge (*tsr*) moderately distant from anterior margin of prosternum in medio-lateral areas (Figures 54A and 59D: 63-0), rarely with somewhat long prosternal process (Figure 59D: 64-1). Elytron (Figures 48A–D,F, 49, 51A,C,D, 57A,C–F and 58A,C) elongate, rather long, lacking macrosetae on dorsum, but with very narrow to wide epipleural gutter along outer margin (e.g., Figures 51A,D and 58A,C: 79-1, 79-2, 79-3); lateral side with epipleural ridge or keel moderately to very strongly folded inward (e.g., Figures 52B,C and 58D: 81-1, 81-3); posterolateral margin sinuate in a few genera (Figure 58C: 83-2). Mesoventrite with (Figures 54C,D and 60A: 85-1) or without (Figure 54E,F: 85-0) longitudinal median carina. Metaventrite (Figures 52B,C and 60A) occasionally punctate (members of former Megarthropsini syn. nov. (see Figure 60A) and subgenus *Tachinoderus* of genus *Tachinus*), rarely medially carinate on intermetacoxal process ([42]: Figures 188 and 189). Metendosternite (Figure 54H: 90-0) with anterior arms thin to relatively thick, but not strongly lamellate. Legs (e.g., Figures 48D–F, 49A,B,D–F, 52A–C, 57 and 58) moderately long, robust; procoxae (e.g., Figure 52A: 96-0) expanded, large; protarsus in male frequently wider and expanded with adhesive setae ([108]: figures 2 and 3); metacoxae (Figures 52B, 58D, and 60E: 98-0, 98-1) small to medium in each outer lateral half; metatibia with long spurs at apex, clearly longer than 2/5 of metatarsomere 1 (Figures 54I and 60C: 101-1); metatarsus (e.g., Figure 52C: 103-0) short to long, shorter than length of metatibia. Abdomen (Figures 48, 49, 52D and 57) weakly to moderately tapering posteriorly, with single pair of paratergites on segments III–VII, lacking blackish macrosetae dorsally (except abdominal terminalia), with exceptions of *Nitidotachinus* and *Leucotachinus*. Tergites III–VII without macrosetae, except *Nitidotachinus* and *Leucotachinus*. Tergite VIII (Figure 55C: 114-1) with only a few to several distinct macrosetae, except *Nitidotachinus*; posterior margin in both sexes usually with lobes (Figure 55C: 116-1), frequently rather strongly modified. Male tergite IX (Figures 55E–G, 61E) fused at base of ventral side, not divided by sternite IX (Figure 61E: 119-1); dorsal side (Figure 55E,F: 120-1) abruptly divided apically in from base, usually forming a slender lobe with pointed apex on each side (Figures 55G and 61E: 122-2) (two each in *Olophrinus*; Figure 55F: 122-3), or less slender, merely elongate in limited taxa (*Nitidotachinus*; Figure 55E: 122-1; [109]: Figure 68); apical areas each with at most several long macrosetae, generally restricted to apices (Figures 55E,F and 61E: 123-2). Sternite III with or without longitudinal median carina. Male sternite VI occasionally with characteristically arranged peg-like setae (Figure 52D: 127-1). Sternite VII in both sexes without semicircular protrusion along basomedial margin as in Figure 47A (128-1), but occasionally with basal lateral pair of protrusions (e.g., Figure 61A); posteromedial margin in male emarginate and concave (Figures 52D, 55A, 61A: 129-1, 129-2, 130-1, 130-2), with several to more than a dozen short and stout characteristically arranged setae or peg-like setae (Figures 52D, 55A and 61A: 131-1, 131-2). Sternite VIII (Figures 55B,D and 61B,C) with only a few to several distinct macrosetae (Figures 55B and 61B,C: 133-1), except *Nitidotachinus* (Figure 55D: 133-0); posterior margin in male (Figures 55B and 61B: 136-1, 136-2) deeply emarginate or incised medially, along with feebly to strongly developed lobes (e.g., Figures 55B, 61B: 135-4, 135-5), that of female (Figures 55D and 61C) nearly rounded or modified: usually with developed lobes and associated smaller internal lobes (Figures 55D and 61C: 137-3), but rarely with only a pair of feebly developed projections, each apex in female typically with row of more than three sensory setae in fan-like arrangement (Figures 55D and 61C: 138-2, 138-4). Male aedeagus (Figures 56A–D and 61F) elongate, usually with more or less wide and flattened, plate-like parameres (Figure 61F: 145-1); parameres longitudinally contiguous or nearly contiguous (Figure 56B: 143-3), rarely separated as in *Leucotachinus* (Figure 56A: 143-1). Female genitalia (Figures 56E, 61D) with gonocoxite II usually slender, narrowly elongate (Figures 56E and 61D: 153-0) (but rarely rather thick, somewhat triangular), bearing only curved setae in most taxa (Figure 56E: 155-1). Spermatheca (Figures 56F and 61D: 151-1) sometimes complicated, frequently associated with basal coils.

Composition: Fifteen genera (3 extinct), with 371 species (7 extinct). The members of the former Megarthropsini syn. nov. are also included. See Table A1 for overview and distributions. See also Appendix A.

- *Austrotachinus* Steel, 1956 [110]: 13.
  Type species: *Austrotachinus fuscipes* Steel, 1956 [110]: 14.
- *Lacvietina* Herman, 2004 [25]: 39.
  Type species: *Lacvietina cuprina* Herman, 2004 [25]: 44.
- *Leucotachinus* Coiffait & Sáiz, 1968 [111]: 411.
  Type species: *Tachinus luteonitens* Fairmaire & Germain, 1862 [112]: 425.
- *Megarthropsis* Cameron, 1919 [101]: 231.
  Type species: *Megarthropsis decorata* Cameron, 1919 [101]: 232.
- *Nepaliodes* Coiffait, 1977 [113]: 272.
  Type species: *Nepaliodes variolosa* Coiffait, 1977 [113]: 272.
- *Nitidotachinus* Campbell, 1993 [109]: 522.
  Type species: *Tachinus tachyporoides* Horn, 1877 [114]: 94.
- *Olophrinus* Fauvel, 1895 [115]: 280.
  Type species: *Olophrinus striatus* Fauvel, 1895: 281.
- *Peitawopsis* Smetana, 1992 [116]: 199.
  Type species: *Peitawopsis monticola* Smetana, 1992 [116]: 204.
- *Pseudotachinus* Cameron, 1932 [117]: 398.
  Type species: *Pseudotachinus niger* Cameron, 1932 [117]: 398.
- *Tachinomorphus* Kraatz, 1859 [118]: 54.
  Type species: *Tachinus fulvipes* Erichson, 1840 [119]: 921.
- *Tachinoplesius* Bernhauer, 1936 [120]: 326.
  Type species: *Tachinoplesius turneri* Bernhauer, 1936 [120]: 327.
- *Tachinus* Gravenhorst, 1802 [41]: 134.
  Type species: *Staphylinus rufipes* Linnaeus, 1758 [38]: 423.
  - Subgenus *Latotachinus* Ullrich, 1975 [121]: 282.
    Type species: *Tachinus punctiventris* Sharp, 1888 [122]: 385.
  - Subgenus *Tachinoderus* Motschulsky, 1858 [123]: 217.
    Type species: *Tachinoderus longicornis* Motschulsky, 1858 [123]: 218.
  - Subgenus *Tachinus* Gravenhorst, 1802 [41]: 134.
    Type species: *Staphylinus rufipes* Linnaeus, 1758 [38]: 423.
- †*Hesterniasca* Zhang, Wang & Xu, 1992 [124]: 279.
  Type species: *Hesterniasca obesa* Zhang, Wang & Xu, 1992 [124]: 279.
- †*Mesotachinus* Tikhomirova, 1968 [50]: 148.
  Type species: †*Mesotachinus major* Tikhomirova, 1968 [50]: 148.
- †*Protachinus* Cai, Yan, Beattie, Wang & Huang, 2013 [52]: 651.
  Type species: *Protachinus minor* Cai, Yan, Beattie, Wang & Huang, 2013 [52]: 652.

Fossils: The Mesozoic fossils of the newly recognized subtribe Tachinusini stat. rev., sensu nov. have been well documented, mainly from the Jurassic-Cretaceous deposits in China. All named extinct taxa in this tribe are based on compression fossils. Unfortunately, most fossil taxa described by early paleontologists require more detailed studies, and many of these fossils were published with short descriptions, incomplete illustrations, and possibly inaccurate observations [47]. Two Mesozoic fossil genera, †*Hesterniasca* and †*Protachinus*, have been placed in Tachyporini under the old sense of the tribe [52,125]. Both genera were considered close relatives to recent members of *Tachinus* [52,125]. In this study, I tentatively follow their systematic placements based on close external similarities with Tachinusini stat. rev., sensu nov. Although the Lower Cretaceous †*Hesterniasca* from the Yixian Formation of northeastern China has an unusual combination of characters for the extant *Tachinus*-related genera (i.e., setose body with contiguous mesocoxae, lacking sexual dimorphisms in tergite VIII and sternite VIII), it generally agrees well with Tachinusini stat. rev., sens. nov. in having long and sub-parallel terminal maxillary palpomeres, longer antennae, broadly arcuate pronotal lateral margins, elongate elytron, posteriorly broadened elytra, only weakly posteriorly tapered abdomen with strongly transverse abdominal tergites III–VI, and the absence of distinct macrosetae on the body, especially those of the abdomen [125]. Similarly, the Upper Jurassic monotypic genus †*Protachinus* from the Talbragar Fish Bed in New South Wales of Australia also possesses some ambivalent morphological features as in †*Hesterniasca*, namely the contiguous mesocoxae, abdominal tergites III–VI each with a pair of curved basolateral ridges, without secondary sexual modifications in tergite VIII and sternite VIII. Nevertheless, †*Protachinus* can be undoubtedly assigned to Tachinusini stat. rev., sensu nov. on the basis of the general body shape, broadly arcuate lateral margins of the pronotum, elongate elytron, posteriorly broadened elytra, only weakly posteriorly tapered abdomen with strongly transverse abdominal tergites III–VI, and the absence of distinct macrosetae on the body, especially those of the abdomen [52]. The presence of distinct discal rows of striae in †*Protachinus* is unusual in Tachyporinae sensu nov., although such elytral striae can be found in this newly recognized tribe as in *Olophrinus* (Figures 48F and 51D; [126,127]). Another extinct genus, †*Mesotachinus* from the Middle–Late Jurassic Karabastau Formation of Karatau in southern Kazakstan [50], with three species, is somewhat problematic, and requires further study and redescriptions of all species using modern techniques including high resolution imaging and SEM (scanning electron microscopy). According to Cai et al. [52], †*Protachinus* resembles †*Mesotachinus* as they share some important morphological features, namely, general body shape, small triangular head, transverse pronotum, contiguous pro- and mesocoxae, truncate elytra, and tapered abdomen. Moreover, this fossil genus has a distinctly transverse abdominal segment IV. In the present study, †*Mesotachinus* is tentatively placed in Tachinusini stat. rev., sensu nov., but future study is needed to clarify its true systematic placement. Some undescribed tachyporines that are being putatively considered here as tachinusines are known from the Middle Jurassic of China, including the misidentified omaliine-like ‘†*Protostaphylinus mirus* Lin, 1976′ [128] (see Cai & Huang [51] for details). Because the additional specimen recorded as †*P*. *mirus* [51,129] seems to be Tachyporinae sensu nov., but the holotype may be attributed to a different subfamily (i.e., Omaliinae [51]). They will definitely be important as future subjects in clarifying tachyporine evolution, although these fossil taxa are removed from the above list and Table A1 to avoid confusion.

In contrast, the Cenozoic fossils of Tachinusini stat. rev., sensu nov. are surprisingly much rarer. For example, only a single, unnamed, *Tachinus* fossil has been known from the mid-Eocene Baltic amber [64]. Additionally, †*Tachinus sommatus* Scudder, 1900, has been described from compression fossils from the Eocene Florissant deposit in Colorado, USA [21,68], but there is no convincing evidence that they truly belong to *Tachinus*.

Remarks: A newly resurrected family-group name, Tachinusini stat. rev., sensu nov. [20,53,130] is used here to accomodate the *Tachinus*-related genera and the South Asian tribe Megarthropsini syn. nov. The latter tribe has been defined with a combination of the following characters within Tachyporinae [25]: body densely punctured; head with the presence of a neck, with reflexed and explanate anterolateral cephalic margin; elytron with emarginate posterolateral margin; and male aedeagus with ventrobasal groove on median lobe. However, the general morphological structures of the megarthropsines fully agree with Tachinusini stat. rev., sensu nov. For example, the structures of the genital segments and genitalia of both sexes in the megarthropsines are almost identical to those in Tachinusini stat. rev., sensu nov. Additionally, the neck-like narrowing is here confirmed as present throughout the tribe (e.g., Figure 51B,D cf. 58A). Other characters defining Megarthropsini syn. nov. can be considered as more or less derived features (such as deep punctures or reflexed clypeal margins), and they are not necessarily important to maintain the distinct tribal status. In addition to observation of morphological characters, the resulting tree supports this conclusion, although the Tachinusini clade was not supported by bootstrap support value pending future studies if Megarthropsini should eventually be considered as a sister taxon of Tachinusini at a subtribe level (Figures 2–4). A few morphological features of the megarthropsine genus *Lacvietina* are superficially similar to some of the members of the subgenus *Tachinoderus* of the genus *Tachinus* in having the long and slender antennae, punctate elytra and metaventrite, and short metatarsi (cf. [131,132]). However, *Lacvietina* can clearly be differentiated from *Tachinoderus* by having the punctate head and pronotum, deep metaventral pit adjacent to apex of the mesosternal process, and reflexed anterolateral margin (clypeus) of the head (Figures 57A, 58A, and 60A,B; cf. [25,133]). They are frequently found from wet micro-environment in forests. In my collecting experience, *Lacvietina takashii* (Hayashi, 2003) [134] and *Tachinus* (*Tachinoderus*) *iriomotensis* Li, 1994 [135] were found together from very wet leaf litter along stream in Iriomote-jima Is., Okinawa, Japan. Hence, several similar morphologies may be explained by convergence as a result of the adaptations to the semi-aquatic environment. I refrained from using *Tachinoderus* in the phylogenetic analyses here, because I could not obtain enough samples of *T*. (*T*.) *iriomotensis*, especially those of the females. A hypothesis of the provisionally distant relationship between *Lacvietina* (or entire Megarthropsini) and *Tachinoderus* will be tested in future, using all subgenera of *Tachinus* (Yamamoto, in prep.).

#### 3.2.14. Tachyporinae, *incertae sedis*: List of Genera with Unknown Systematic Placements within Tachyporinae sensu nov.

- †*Abscondus* Tikhomirova, 1968 [50]: 151.
  Type species: *Abscondus regularis* Tikhomirova, 1968 [50]: 152.
- †*Tachyporoides* Tikhomirova, 1968 [50]: 150.
  Type species: *Tachyporoides villosus* Tikhomirova, 1968 [50]: 151.

Remarks: Two fossil genera from the Middle–Late Jurassic Karabastau Formation are provisionally considered as “*incertae sedis*” within Tachyporinae sensu nov., following the taxonomic treatments in Herman [21]. It is almost impossible to extract adequate morphological information from published sources [50], or even from the poorly preserved compression type specimens. Cai et al. [125] briefly mentioned that †*Abscondus* is similar to †*Hesterniasca*, an extinct member of Tachinusini stat. rev., sensu nov. Nonetheless, some of the important characters, including those of the maxillary palpus, are not preserved in the type material, preventing further discussion. †*Tachyporoides* also remains enigmatic because of the significant lack of morphological information in both the fossil and the original description [50]. As suggested by a close similarity to *Tachyporus* [50], it may belong to Tachyporina stat. nov., sensu nov. (Tachyporini sensu nov.) on the basis of general body shape, large mesocoxae, rather short elytra, and pubescent body. However, the presence of the postcoxal process of the prosternum and seemingly wider tergites are unusual for this subtribe. In any case, further evidence is needed for the taxonomic placement of †*Tachyporoides*. These fossil taxa should be re-examined with modern imaging techniques and SEM according to the current scientific standard [47].

#### 3.2.15. Subfamily Mycetoporinae Thomson, 1859 stat. nov.

Mycetoporinae Thomson, 1859: 46 [136] (Figures 1E, 2–5, 62–73 and Figure S2A; Table A2)

Type genus: *Mycetoporus* Mannerheim, 1831 [137]: 476.

Differential diagnosis: Mycetoporinae stat. nov. differ from all other subfamilies of Staphylinidae based on the following combination of characters: body slender and fusiform, head small, frequently elongate, with distinct and complete ridge below eye, antennal insertions fully exposed, located anterior to eyes, mandibles falciform without inner teeth, elytron with longitudinally raised sutural edge, epipleural keel not folded inward, procoxae very large and strongly expanded, tarsal formula 5-5-5, metacoxae markedly large, metatarsi longer than metatibiae, and abdomen with two pairs of paratergites, having very fine ‘brick-wall’ pattern on intersegmental membranes.

**Figure 62:**
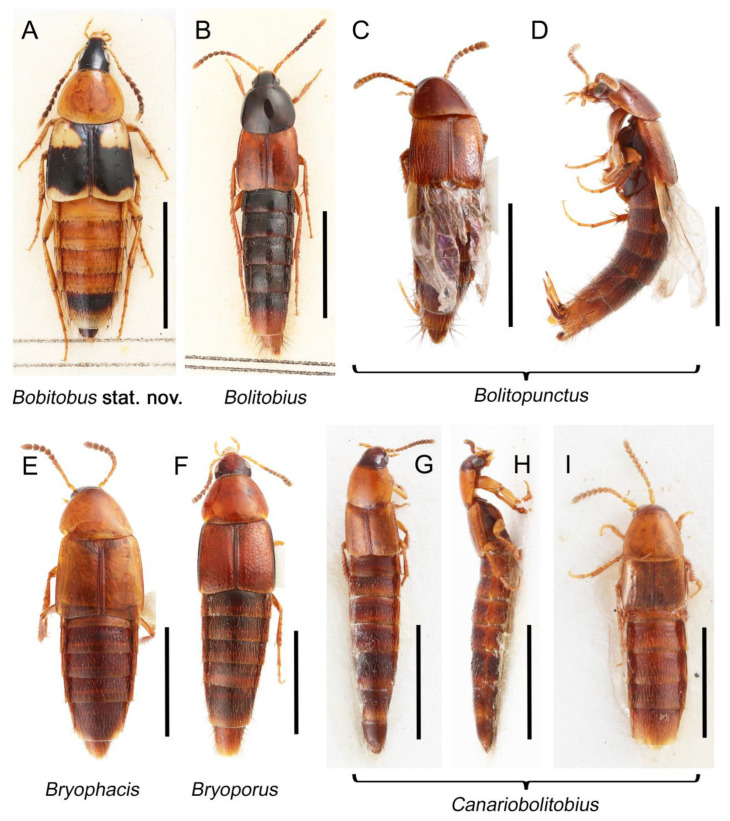
Habitus photographs of Mycetoporinae. (**A**) *Bobitobus lunulatus* (Linnaeus), dorsal view. (**B**) *Bolitobius castaneus*, dorsal view. (**C**) *Bolitopunctus* sp., dorsal view. (**D**) *Bolitopunctus* sp., lateral view. (**E**) *Bryophacis smetanai* Campbell, dorsal view. (**F**) *Bryoporus rufescens* LeConte, dorsal view. (**G**) *Canariobolitobius filicornis* (Wollaston) specimen 1, dorsal view. (**H**) *Canariobolitobius filicornis* specimen 1, lateral view. (**I**), *Canariobolitobius filicornis* specimen 2, dorsal view. Scale bars: 3.0 mm (**A**,**B**); 1.5 mm (**C**–**E**,**G**,**H**); 2.0 mm (**F**); 1.0 mm (**I**).

**Figure 63:**
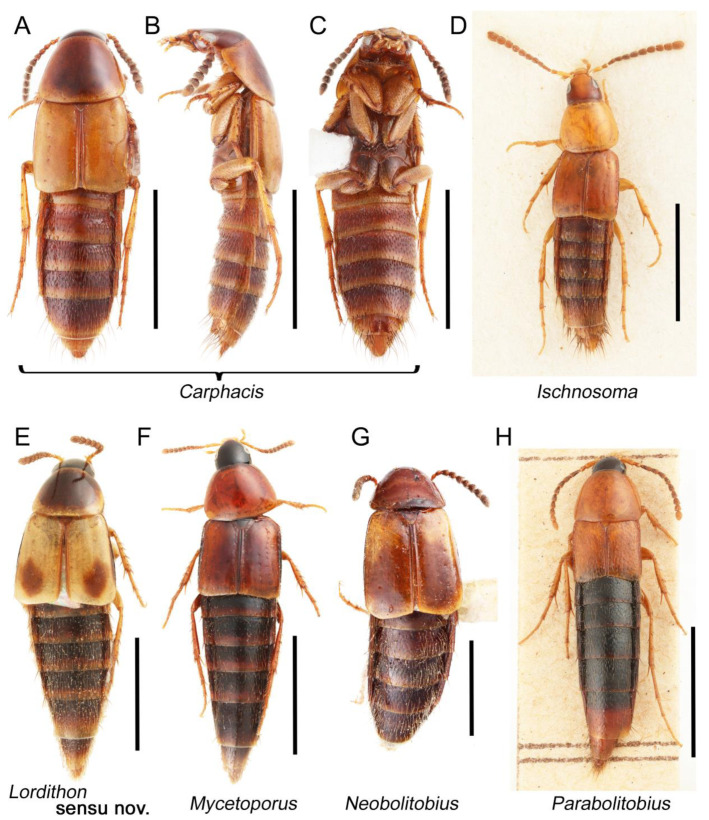
Habitus photographs of Mycetoporinae. (**A**) *Carphacis effrenatus* Herman, dorsal view. (**B**) *Carphacis effrenatus*, lateral view. (**C**) *Carphacis effrenatus*, ventral view. (**D**) *Ischnosoma splendidum* (Gravenhorst), dorsal view. (**E**) *Lordithon thoracicus thoracicus* (Fabricius), dorsal view. (**F**) *Mycetoporus punctus* (Gravenhorst), dorsal view. (**G**) *Neobolitobius varians* (Hatch), dorsal view. (**H**) *Parabolitobius formosus* (Gravenhorst), dorsal view. Scale bars: 2.0 mm (**A**–**C**,**F**); 1.5 mm (**D**,**E**,**G**); 3.0 mm (**H**).

**Figure 64:**
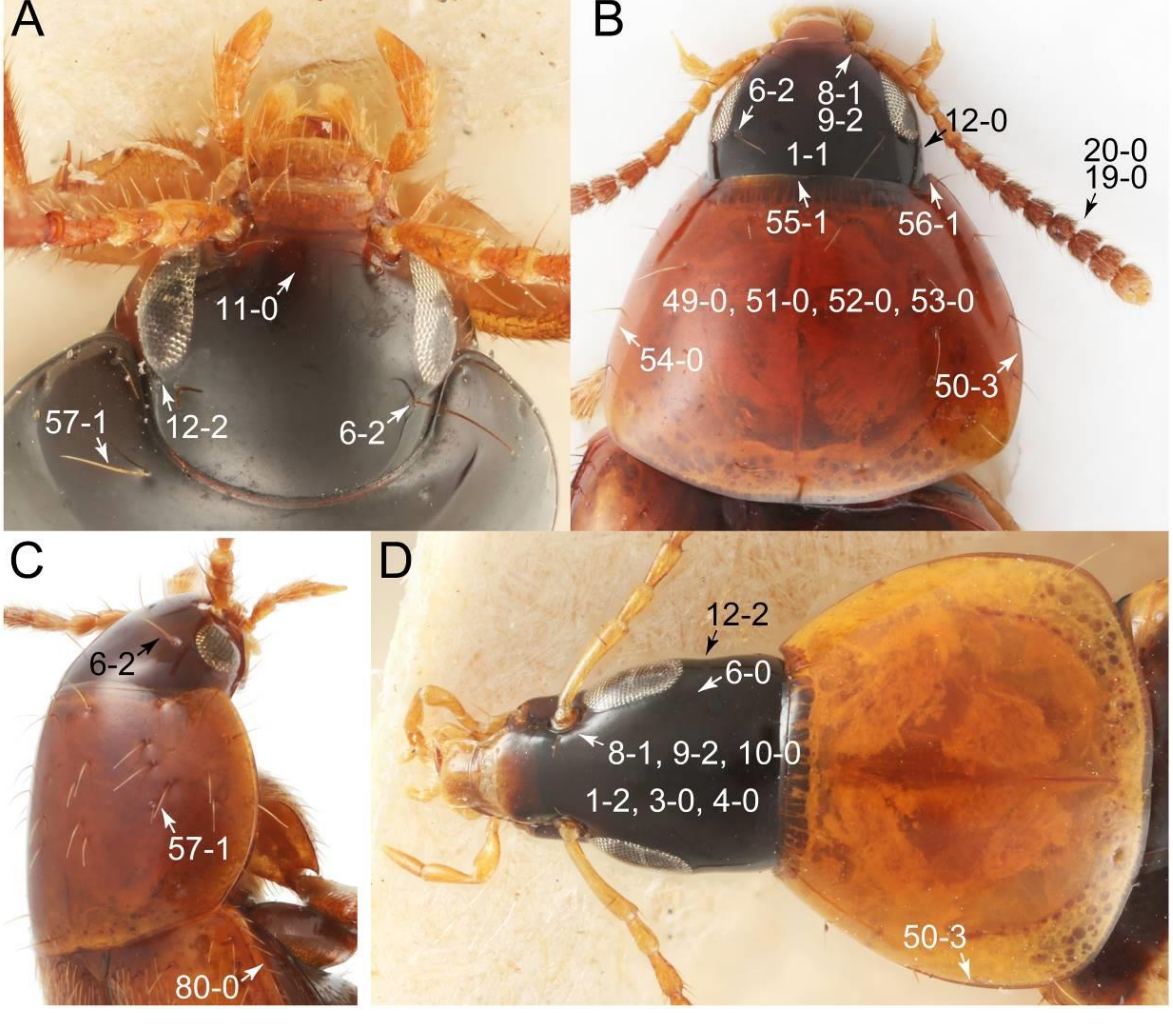
Photographs of body parts of Mycetoporinae, head and pronotum, enlarged. (**A**) *Bolitobius castaneus*, frontal view. (**B**) *Mycetoporus punctus*, dorsal view. (**C**) *Bolitopunctus* sp., dorsolateral view. (**D**) *Bobitobus lunulatus*, dorsal view. Characters and character states (format X-X) are indicated on each figure.

**Figure 65:**
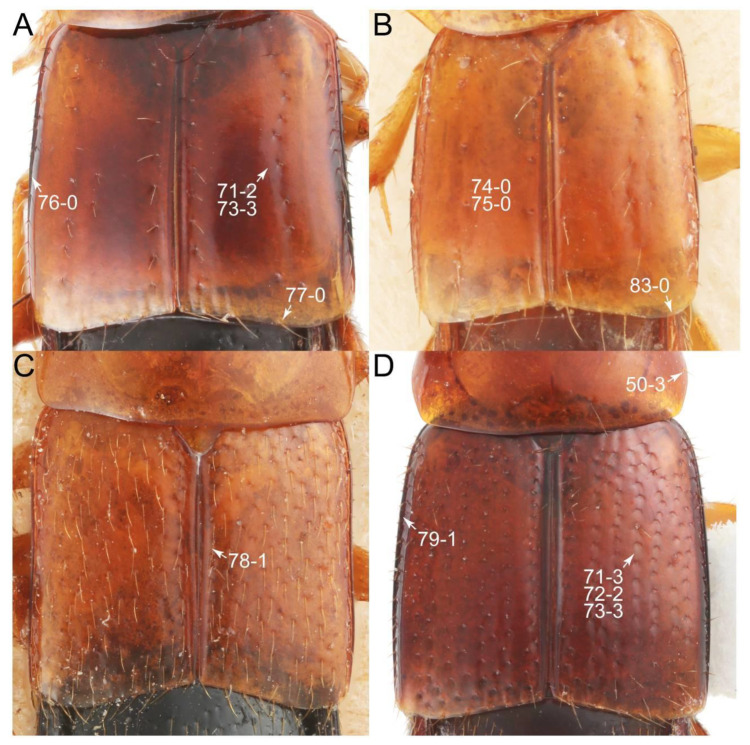
Photographs of body parts of Mycetoporinae, elytra, enlarged, dorsal view. (**A**) *Mycetoporus punctus*. (**B**) *Ischnosoma splendidum*. (**C**) *Parabolitobius formosus*. (**D**) *Bryoporus rufescens*. Characters and character states (format X-X) are indicated on each figure.

**Figure 66:**
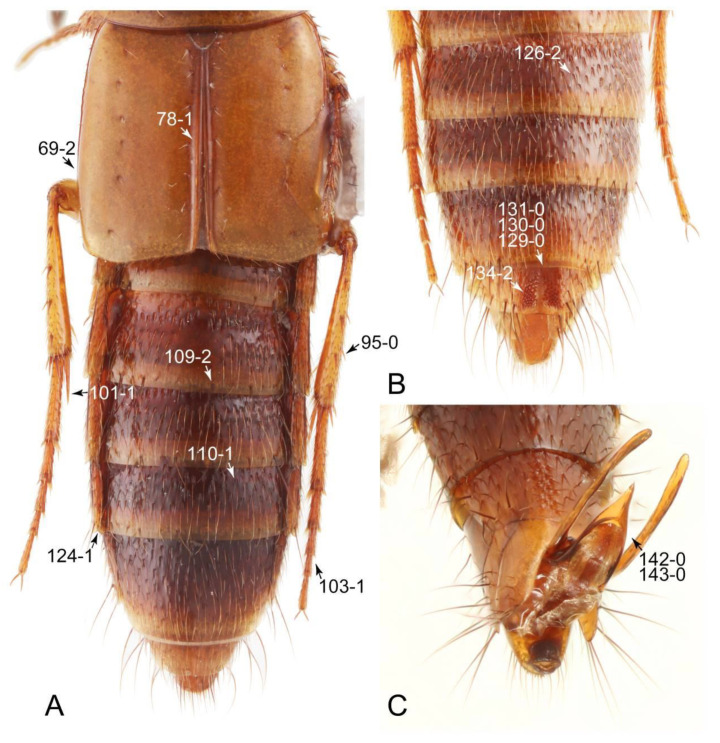
Photographs of body parts of Mycetoporinae, enlarged. (**A**) elytra, hindlegs, and abdomen of *Carphacis effrenatus*, dorsal view. (**B**) male abdomen of *Carphacis effrenatus*, ventral view. (**C**) male abdominal terminalia and genitalia of *Bolitopunctus* sp., ventral view. Characters and character states (format X-X) are indicated on each figure.

**Figure 67:**
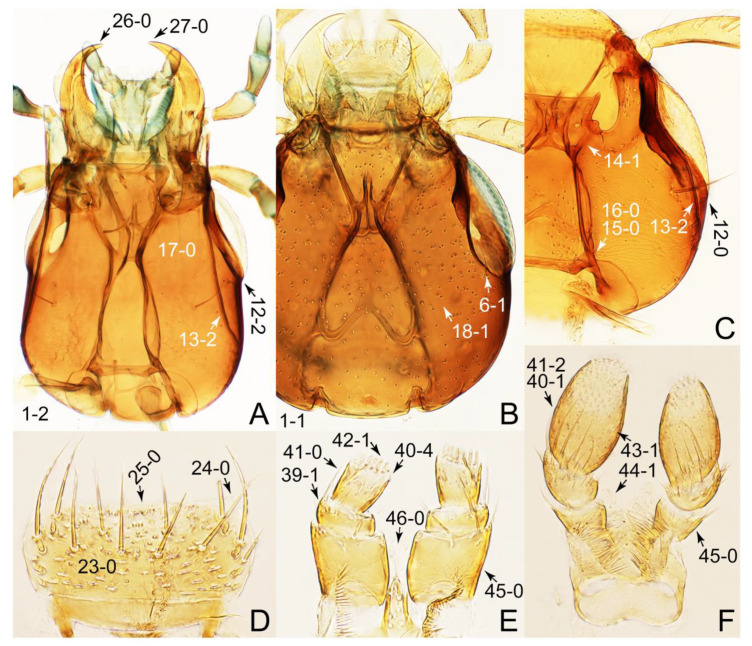
Photographs of body parts of Mycetoporinae, enlarged. (**A**) head of *Carphacis effrenatus*, ventral view. (**B**) head of *Bryophacis smetanai*, dorsal view. (**C**) head of *Bryoporus* cf. *orientalis* Cameron, ventral view. (**D**) labrum of *Bryoporus* cf. *orientalis*, dorsal view. (**E**) labium of *Bryoporus* cf. *orientalis*, ventral view. (**F**) labrum of *Parabolitobius formosus*, ventral view. Characters and character states (format X-X) are indicated on each figure.

**Figure 68:**
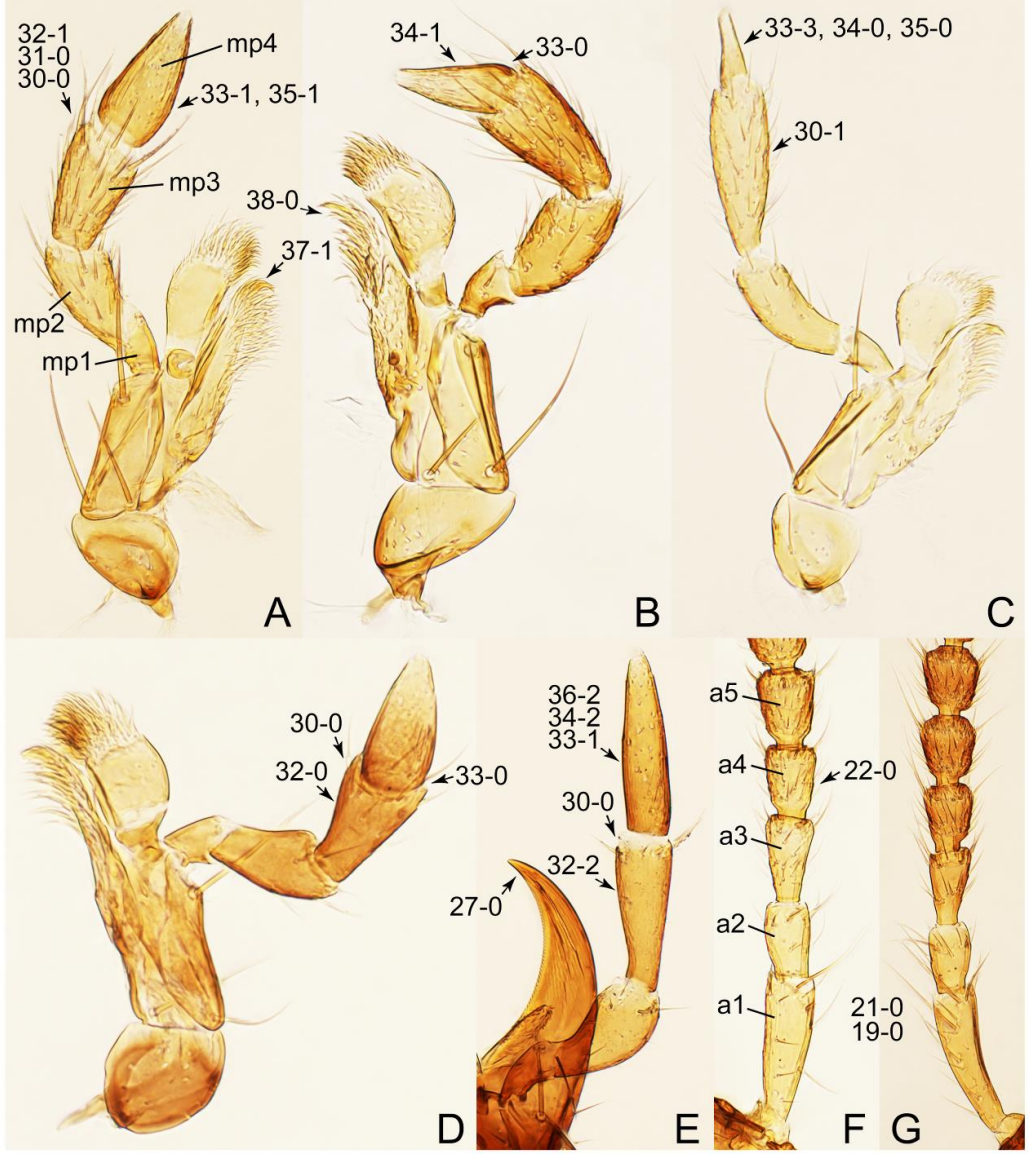
Photographs of body parts of Mycetoporinae, enlarged. (**A**) right maxilla of *Bolitobius castaneus*, ventral view. (**B**) right maxilla of *Bryoporus* cf. *orientalis*, dorsal view. (**C**) right maxilla of *Mycetoporus punctus*, ventral view. (**D**) left maxilla of *Lordithon thoracicus thoracicus*, ventral view. (**E**) left maxillary palpus and left mandible of *Bobitobus lunulatus*, ventral view. (**F**) basal half of left antenna of *Mycetoporus punctus*, dorsal view. (**G**) basal half of right antenna of *Bryoporus* cf. *orientalis*, ventral view. Abbreviations: a1–5, antennomere 1–5; mp1–4, maxillary palpomere 1–4. Characters and character states (format X-X) are indicated on each figure.

**Figure 69:**
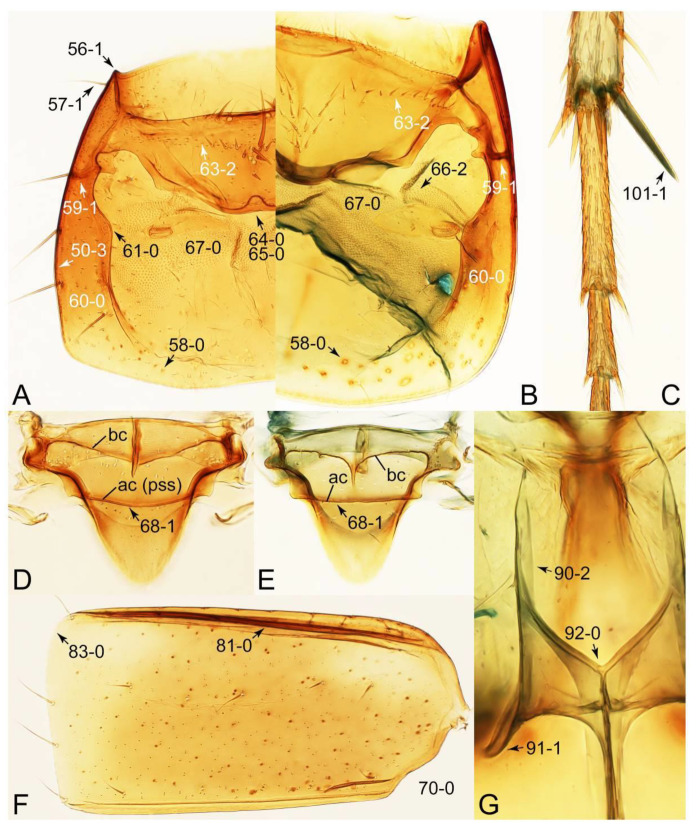
Photographs of body parts of Mycetoporinae, enlarged. (**A**) prosternum and pronotum of *Bryoporus* cf. *orientalis*, ventral view. (**B**) prosternum and pronotum of *Carphacis effrenatus*, ventral view. (**C**) left metatarsus and metatibial spines of *Carphacis effrenatus*, dorsal view. (**D**) scutellum of *Bolitobius castaneus*, dorsal view. (**E**) scutellum of *Carphacis effrenatus*, dorsal view. (**F**) left elytron of *Ischnosoma splendidum*, dorsal view. (**G**) metendosternite of *Carphacis effrenatus*, dorsal view. Abbreviations: ac, apical carina; bc, basal carina; pss, prescutoscutellar suture. Characters and character states (format X-X) are indicated on each figure.

**Figure 70:**
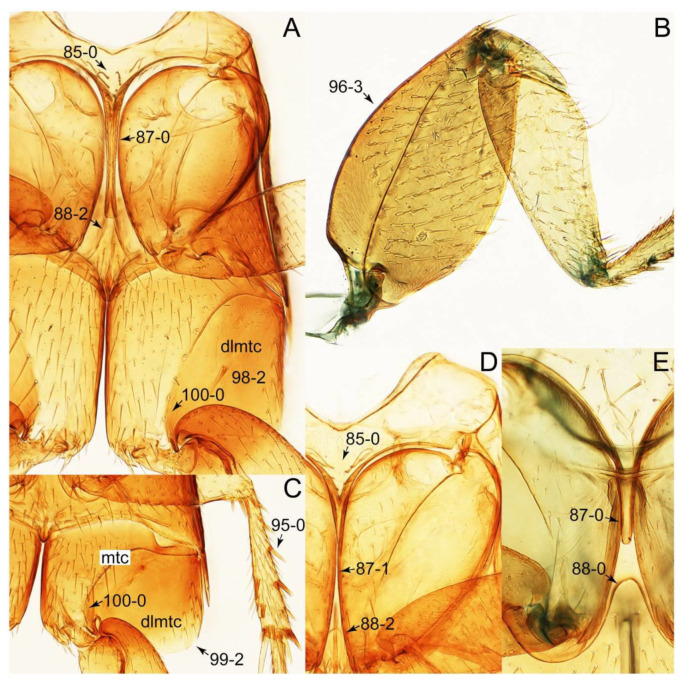
Photographs of body parts of Mycetoporinae, enlarged. (**A**) mesothorax and metathorax of *Ischnosoma splendidum*, ventral view. (**B**) right foreleg of *Bryoporus rufescens*, frontal view. (**C**) left mesotibia and metacoxa of *Mycetoporus punctus*, ventral view. (**D**) intermesocoxal processes of *Parabolitobius formosus*, ventral view. (**E**) intermesocoxal processes of *Carphacis effrenatus*, ventral view. Abbreviations: dlmtc, dorsal lamella of the metacoxa; mtc, metacoxa. Characters and character states (format X-X) are indicated on each figure.

**Figure 71:**
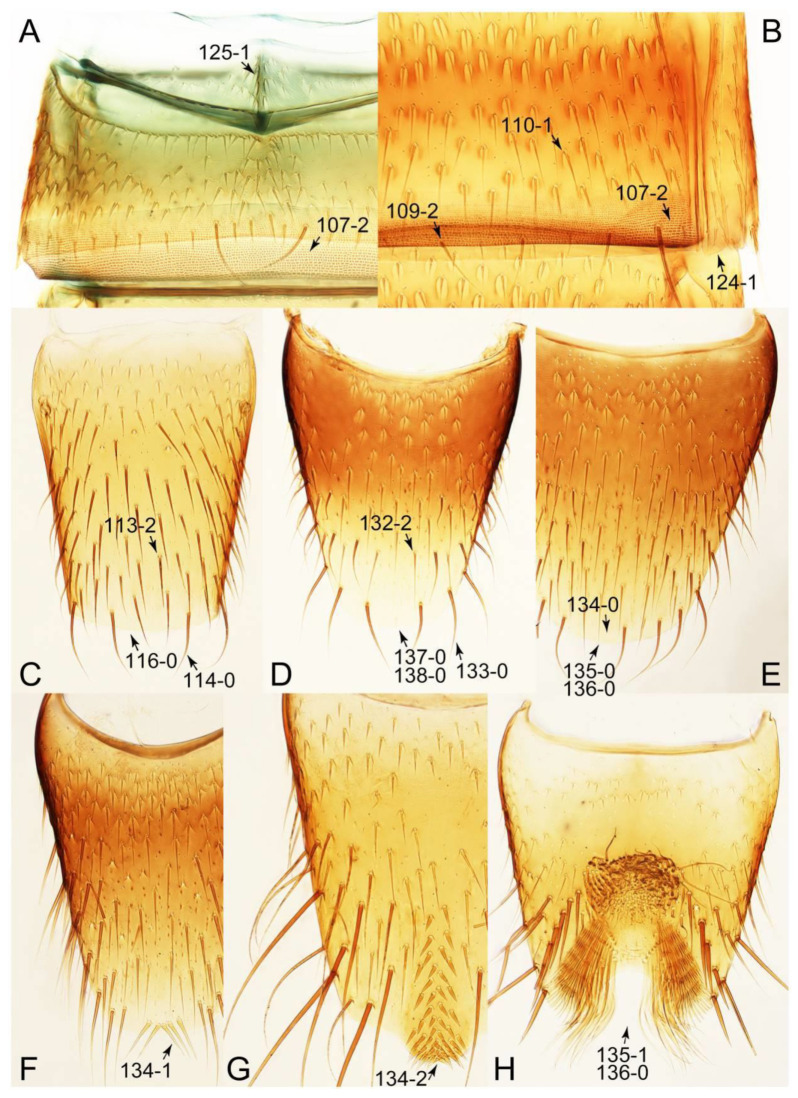
Photographs of body parts of Mycetoporinae, enlarged. (**A**) sternite III of *Carphacis effrenatus*, ventral view. (**B**) posterior margin of tergite V of *Carphacis effrenatus*, dorsal view. (**C**) male tergite VIII of *Mycetoporus punctus*, dorsal view. (**D**) female sternite VIII of *Lordithon thoracicus thoracicus*, ventral view. (**E**) male sternite VIII of *Mycetoporus punctus*, ventral view. (**F**) male sternite VIII of *Bryoporus* cf. *orientalis*, ventral view. (**G**) male sternite VIII of *Bolitobius castaneus*, ventral view. (**H**) male sternite VIII of *Ischnosoma splendidum*, ventral view. Characters and character states (format X-X) are indicated on each figure.

**Figure 72:**
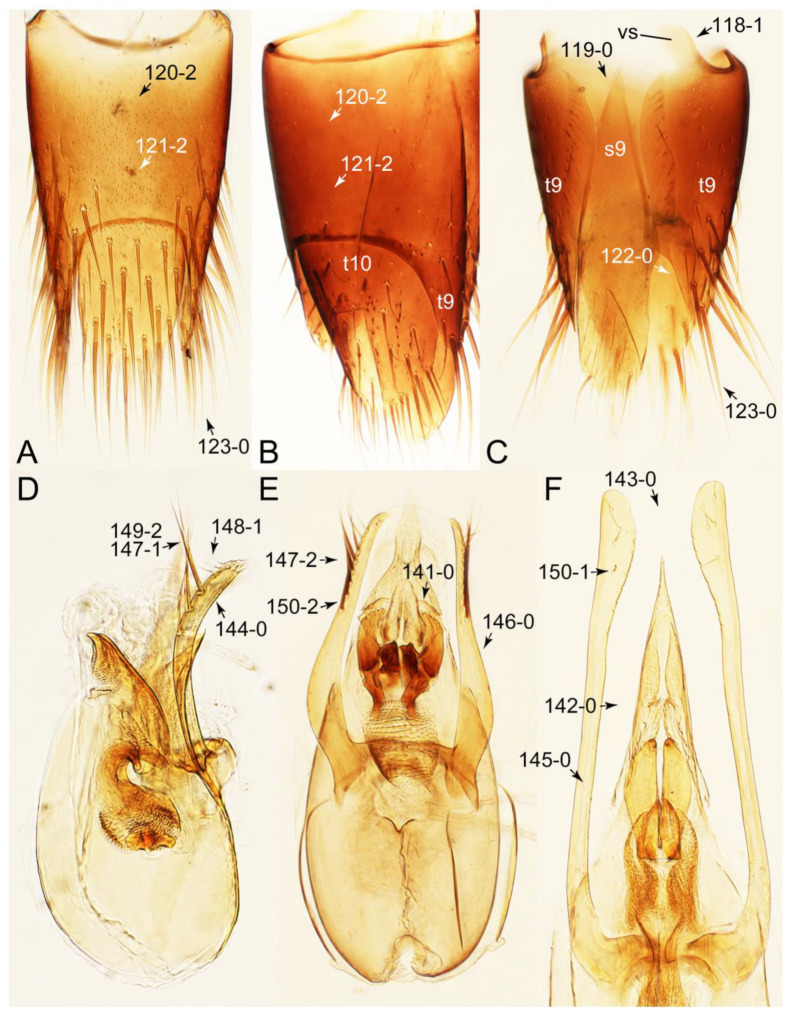
Photographs of body parts of Mycetoporinae, male genital segments and aedeagus, enlarged. (**A**) tergites IX–X of *Bryoporus* cf. *orientalis*, dorsal view. (**B**) tergites IX–X of *Bobitobus lunulatus*, dorsolateral view. (**C**) tergites IX and sternite IX of *Ischnosoma splendidum*, ventral view. (**D**) aedeagus of *Bryophacis smetanai*, lateral view. (**E**) aedeagus of *Bobitobus lunulatus*, ventral view. (**F**) aedeagus of *Parabolitobius formosus*, ventral view. Abbreviations: s9, sternite IX; t9–10, tergite IX–X; vs, ventral strut. Characters and character states (format X-X) are indicated on each figure.

Emended diagnosis: Body narrowly elongate, fusiform, somewhat strongly tapered (Figure 62 and Figure 63); head and pronotum uniformly glabrous, except for some macrosetae (not as ground setation) (e.g., Figure 64: 4-0); head small, usually longer than wide (e.g., Figure 64B,D: 1-1, 1-2), with neither midcranial suture nor distinct neck constriction (e.g., Figures 64 and 67C: 11-0, 12-0), ventral side with distinct and complete ridge below eye (Figure 67A,C: 13-2); ocular puncture, usually with its associated seta, located near posterior of each eye (Figures 64A,B and 67B: 6-1, 6-2); antennal insertion fully exposed, located anterior to eye (e.g., Figure 64B,D: 8-1, 9-2, 10-0); antennae unmodified, not extremely slender and verticillate; mandibles without inner teeth (e.g., Figures 67A and 68D: 26-0, 27-0); maxillary palpus 4-segmented; labial palpus 3-segmented, insertions narrowly separated (e.g., Figure 67E: 46-0); pronotum widest in basal 1/4 to middle (e.g., Figure 64B,D: 50-3); pronotal hypomeron narrow, with transverse ridge at apical 1/3 to 1/4 (Figure 69A,B: 59-1); procoxal cavities fully open behind (Figure 69A,B: 66-2); mesospiracular peritremes not well sclerotized, mostly membranous (Figure 69A,B: 67-0); elytron frequently with impressed sutural striae and epipleural keel (Figures 64C, 65, 66A and 69F: 71-2, 71-3, 80-0), with longitudinally raised sutural edge (e.g., Figure 65 and Figure 66A: 78-1); legs with strong tibial spines (e.g., Figure 66A: 95-0); procoxa very large, strongly expanded, with exposed protrochantin (Figures 63C and 70B: 96-3); metacoxae markedly large (e.g., Figure 70A,C: 98-2); tarsi 5-5-5, with very long metatarsus (e.g., Figure 66A: 103-1); abdomen usually covered with roughly V-shaped setigerous punctures (e.g., Figure 66A,B: 110-1), with six visible sternites and two pairs of paratergites on segments III–VII (Figures 63C and 71B: 124-1); intersegmental membranes with very fine ‘brick-wall’ pattern (Figure 71A,B: 107-2); tergite VIII and sternite VIII usually simple, without sexual dimorphisms (Figure 71C–E: 116-0, 135-0, 137-0), but with some exceptions (Figure 71F–H: 134-1, 134-2, 135-1); male tergite IX continuous dorsally in basal half (Figure 72A,B: 120-2, 121-2), with ventral struts (Figure 72C, vs: 118-1), basally separated by sternite IX (Figure 72C: 119-0); male aedeagus with simple and separated parameres (Figure 72E,F: 142-0, 143-0), each paramere bearing longitudinal row of setae (Figure 72D–F: 147-1, 147-2).

**Figure 73:**
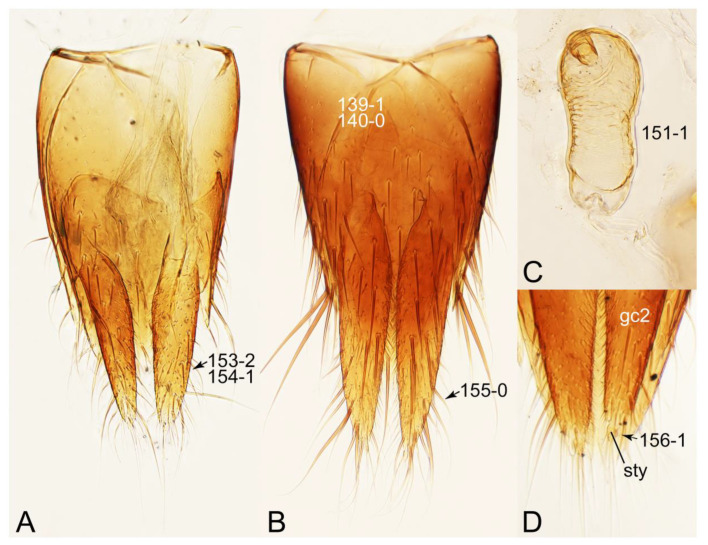
Photographs of body parts of Mycetoporinae, female genital segments and genitalia, enlarged. (**A**) genital segments of *Bryophacis smetanai*, ventral view. (**B**) genital segments of *Ischnosoma splendidum*, dorsal view. (**C**) spermatheca of *Bryoporus* cf. *orientalis*. (**D**) gonocoxite II and gonostylus of *Neobolitobius varians*, ventral view. Abbreviations: gc2, gonocoxite II; sty, gonostylus. Characters and character states (format X-X) are indicated on each figure.

Description: Body (Figures 62 and 63) small to medium, fusiform, narrowly elongate, slender; surface strongly glossy; head and pronotum without ground microsetae and punctation (e.g., Figure 64: 3-0, 4-0, 51-0, 53-0); color (Figures 62 and 63) frequently at least partially bright, occasionally having markings or patterns on dorsum. Head (Figures 64 and 67A–C) clearly smaller than pronotum, as long as wide to narrowly elongate, usually longer than wide (Figures 64B,D and 67A: 1-1, 1-2); ocular setae (see Campbell [32]: figures 7–9) located near base of eyes on dorsal head surface (Figure 64A–C: 6-2), except most members of *Bobitobus* stat. rev. (Figure 64D: 6-0); frontal suture present, but lacking midcranial suture (Figures 64A and 67B: 11-0); tempora or postocular areas without neck constriction (e.g., Figures 64B and 67C: 12-0), but sometimes associated with only weak neck-like narrowing just behind eyes (e.g., Figures 64D and 67A: 12-2); occiput with pair of distinct notches dorsally along posterior margin (Figure 67A,B); ventral side with complete longitudinal basolateral ridges along eyes (Figure 67A,C: 13-2). Dorsal tentorial arms (Figure 67B: 18-0) usually absent or strongly reduced, but developed in earliest diverging genera *Parabolitobius* and *Bolitobius*. Hypostomal sutures (Figure 67C: 14-1) fully separated, short, each straight. Gular sutures (Figure 67A,C) widely and fully separated (Figure 67C: 15-0), each rather long, more or less straight (Figure 67C: 16-0). Antennal insertion (Figures 64, 67B, C) located at, or anterior to, anterior margin of eyes (Figure 64D: 10-0), easily visible from above (Figures 64D: 9-2). Antenna (Figures 62, 63, 64B and 68F,G) rather long, slender, fili-moniliform, not verticillate (Figures 64B, 68F, G: 19-0), lacking clear pattern of dense and fine recumbent pubescence (Figure 68F,G: 21-0), except *Neobolitobius*. Labrum (Figures 64A,B and 67D: 24-0, 25-0) usually lacking setose or spinose processes in middle and lateral areas along anterior margin. Mandible (Figures 67A,B and 68D) falciform, sharply pointed, lacking subapical inner tooth (Figures 67A,B and 68D: 26-0, 27-0), with rather small molar lobe. Maxillary palpus (Figures 64, 67A and 68A–D) 4-segmented, moderately long; palpomere 3 not distinctly shorter than maxillary palpomere 2 (>0.7×; Figure 68A: 31-0); palpomere 4 usually with pores (e.g., Figure 68E: 36-2). Labial palpus (Figure 67A,E,F) 3-segmented, thick, relatively conspicuous; palpomere 3 (Figure 67F: 40-1) widest around middle or near base, not at base, in most taxa, with inconspicuous pores (Figure 67F: 43-1); insertions of labial palpi only narrowly separated (e.g., Figure 67E: 46-0). Ligula (glossae) small, restricted to median area, occasionally associated with medial premental lobes (*Bryoporus*), not extending beyond each labial palpus laterally (Figure 67E,F: 45-0). Pronotum (Figures 64 and 65D) widest in between basal 1/4 to middle (Figure 64B,D: 50-3); surface even and smooth, with characteristically arranged macrosetae (Figure 64A,C). Pronotal hypomeron narrow (Figure 69A,B: 60-0), strongly inflexed and not visible in lateral view (Figure 69A,B: 60-0), with transverse ridge at apical 1/3 to 1/4 (Figure 69A,B: 59-1); postcoxal process (Figure 69A: 61-0) absent. Procoxal cavities (Figure 69A,B: 66-2) nearly fully open behind. Mesospiracular peritremes (Figure 69A,B: 67-0) not well sclerotized, membranous. Scutellum (e.g., Figures 65, 66A and 69D,E) with prescutoscutellar suture (*pss*) (sensu Blackwelder [30]: Figure 4A,C) here referred to as apical carina or ridge (Figure 69D,E, *ac*) distant from base, lying near middle of scutellum (Figure 69D,E: 68-1); basal carina or ridge (Figure 69D,E, *bc*) with variations among genera from linear to curved, pointed, and divided [32,33]. Elytron (e.g., Figures 65, 66A and 69F) relatively long, elongate (length/width: >1.8×; Figure 69F: 70-0), exposing most of abdomen; lateral side with epipleural ridge or keel (Figure 64C: 80-0), epipleuron only very weakly or not at all folded inward (Figure 69F: 81-0); sutural edge clearly longitudinally raised (Figures 65C and 66A: 78-1); surface with rows of setigerous punctures (see Figure 65A,D: 71-2, 71-3). Mesocoxal cavities (Figure 70A,D,E) large, occupying larger area on metaventrite (Figure 70A). Metendosternite (Figure 69G) without median process (Figure 69G: 92-0), but with posterolateral arms (Figure 69G: 91-1) and lamellate anterior arms (Figure 69G: 90-2). Legs (Figures 62, 63 and 66A) with 5-5-5 tarsal formula and strongly developed tibial spines (Figure 70C: 95-0); protrochantin well exposed; procoxae (Figures 63C, 70B) very large, strongly expanded, even larger than profemora (Figure 70B: 96-3); mesocoxae (Figure 70A,D,E) narrowly separated in most taxa (Figure 70A,E: 87-0), rarely contiguous (Figure 70D: 87-1); metacoxae (Figure 70A,C) markedly large (Figure 70A and Supplementary Figure S2: 98-2), contiguous, without ventral lamellae (Figure 70A, C: 100-0); metatarsi (Figure 66A,B) distinctly long, much longer than metatibiae (Figure 66A: 103-1). Abdomen (Figures 62, 63, 66A and 71A,B) gradually tapering from base to apex, with six visible sternites; two pairs of paratergites (Figures 66A and 71B) per segment on segments III–VII (Figure 71B: 124-1); intersegmental membranes with very fine ‘brick-wall’ pattern (Figure 71A,B: 107-2); surface frequently covered with setigerous V-shaped punctation punctures (Figures 66A,B and 71B: 110-1); tergite VII sometimes with distinct row of white palisade setae along posterior margin ([138]: Figure 15); tergite VIII and sternite VIII usually simple without sexual dimorphisms (Figure 71C–E: 116-0, 135-0, 137-0), but occasionally modified (Figure 71H: 135-1) and/or with characteristically arranged thin or peg-like setae (Figures 66B and 71F–H: 134-1, 134-2). Male genital segments (abdominal segments IX and X): tergite IX (Figure 72A–C) continuous dorsally in basal half (Figure 72A,B: 120-2, 121-2), basally separated by sternite IX (Figure 72C: 119-0), with ventral struts (Figure 72C: 118-1). Male genitalia: aedeagus (Figures 66C and 72D–F) with parameres simple, widely separated from each other and from median lobe (Figure 72F: 142-0, 143-0), with rows of parameral setae (Figure 72D,E: 147-1, 147-2); internal sac without prominent spines (Figure 72E: 141-0). Female genital segments (Figure 73A,B,D: 139-1) moderately to strongly elongate; basal area of tergite IX well sclerotized (Figure 73B: 140-0). Female genitalia: gonocoxites (Figure 73A,B,D) present, distinct and large, well sclerotized; gonocoxite II thick, narrowly elongate (Figure 73A: 153-2), well-sclerotized, much larger than gonostylus (Figure 73A: 154-1), usually covered with normal, non-curved setae (see Figure 73B: 155-0); gonostylus (Figure 73A,B,D) minute, inconspicuous (Figure 73D: 156-1), with apical seta.

Composition: Sixteen genera (4 extinct), with 444 species (10 extinct). See Table A2 for overview and distributions. The type species of *Mycetoporus* and *Ischnosoma* and their type species are followed the decision (Opinion 1726) made by the ICZN [139].

- *Bobitobus* Tottenham, 1939 [140]: 225 stat. rev.
  Type species: *Staphylinus lunulatus* Linnaeus, 1767 [94]: 684.
- *Bolitobius* Leach, 1819 [141]: 176.
  Type species: *Megacronus castaneus* Stephens, 1832 [142]: 166.
- *Bolitopunctus* Campbell, 1993 [33]: 29.
  Type species: *Bryoporus muricatulus* Hatch, 1957 [143]: 127.
- *Bryophacis* Reitter, 1909 [144]: 102.
  Type species: *Bolitobius rufus* Erichson, 1839 [145]: 407.
- *Bryoporus* Kraatz, 1857 [96]: 452.
  Type species: *Tachinus cernuus* Gravenhorst, 1806 [58]: 31.
- *Canariobolitobius* Schülke, 2004 [146]: 1024.
  Type species: *Bolitobius filicornis* Wollaston, 1864 [147]: 560.
- *Carphacis* Gozis, 1886 [148]: 14.
  Type species: *Staphylinus striatus* Olivier, 1795 [149]: (42): 28.
- *Ischnosoma* Stephens, 1829 [150]: 22.
  Type species: *Tachinus splendidus* Gravenhorst, 1806 [58]: 24.
- *Lordithon* Thomson, 1859 [136]: 47 sensu nov.
  Type species: *Oxyporus pygmaeus* Fabricius, 1777 [151]: 242 (= *Oxyporus thoracicus* Fabricius, 1777 [151]: 242).
- *Mycetoporus* Mannerheim, 1831 [137]: 476.
  Type species: *Tachinus punctus* Gravenhorst, 1806 [58]: 27.
- *Neobolitobius* Campbell, 1993 [33]: 35.
  Type species: *Lordithon varians* Hatch, 1957 [143]: 128.
- *Parabolitobius* Li, Zhao & Sakai, 2000 [152]: 11.
  Type species: *Megacronus prolongatus* Sharp, 1888 [122]: 460.
- †*Cuneocharis* Ryvkin, 1990 [153]: 64.
  Type species: †*Cuneocharis elongatus* Ryvkin, 1990 [153]: 65.
- †*Glabrimycetoporus* Yue, Zhao & Ren, 2009 [154]: 64.
  Type species: †*Glabrimycetoporus amoenus* Yue, Zhao & Ren, 2009 [154]: 67.
- †*Ryvkinius* Herman, 2001 [67]: 55 (= †*Mesoporus* Ryvkin, 1990 [153]: 63).
  Type species: †*Mesoporus gracilis* Ryvkin, 1990 [153]: 64; preoccupied, nec. Cameron [155].
- †*Undiatina* Ryvkin, 1990 [153]: 62.
  Type species: †*Undiatina pilosa* Ryvkin, 1990 [153]: 63.

Fossils: The fossil records of Mycetoporinae stat. nov. are relatively rare and sparse from both the Cenozoic and Mesozoic. Only a single extinct genus †*Glabrimycetoporus* Yue, Zhao & Ren, from the Lower Cretaceous Yixian Formation of China, has provisionally been assigned as a fossil taxon of Mycetoporinae stat. nov. Based on the sufficient evidence shown in Yue et al. [154], the systematic placement of this fossil genus is justified. Ryvkin [153] described the fossil genus †*Cuneocharis* Ryvkin, from the Lower Cretaceous of Daya in Russia, as a member of Mycetoporinae stat. nov. (originally treated as Bolitobiini). This taxonomic assignment may be correct, although the taxon needs redescription with detailed imaging. He additionally established the two Lower Cretaceous tachyporine genera in the same paper and placed them in Tachyporini under the traditional sense, namely †*Undiatina* Ryvkin from Daya and †*Ryvkinius* Herman (= *Mesoporus* Ryvkin) from Semyon (both in Russia). Although these three genera were each originally placed in a specific tribe, Herman [21] treated them as *incertae sedis*. In the present study, †*Undiatina* and †*Ryvkinius* are provisonally treated as Mycetoporinae stat. nov. based on their markedly large mesocoxae and metacoxae. Furthermore, †*Ryvkinius* has a long abdomen with a longitudinal shallow groove covered with small spines on abdominal sternite VIII [153]. Although no fossil mycetoporines are recorded from Mesozoic amber, I have a *Bolitobius*-like specimen in mid-Cretaceous Kachin amber from northern Myanmar (Yamamoto, in prep.).

Several named Cenozoic taxa definitely need further studies with modern scientific techniques to confirm their systematic placements at genus or even subfamily level [47]. Four *Lordithon* species have been described from the Eocene Florissant deposit in Colorado, USA [21,68]: †*Lordithon durabilis* (Scudder, 1900) [68], †*L. funditus* (Scudder, 1900) [68], †*L. lyelli* (Scudder, 1900) [68], and †*L. stygis* (Scudder, 1900) [68]. Another mycetoporine species, †*Mycetoporus demersus* Scudder, 1900 [68], has also been described from the same deposit [68]. Unfortunately, few amber inclusions of Mycetoporinae stat. nov. have been known, with only a single described species, i.e., †*Bolitobius groehni* Schülke, 2000 [156] from mid-Eocene Baltic amber [156]. Nonetheless, I have seen several mycetoporine species in Baltic amber.

Remarks: The tribe Mycetoporini (= Bolitobiini) is here raised to subfamily status following my results and several previous studies (e.g., [6,7]), as Mycetoporinae stat. nov. The overall morphology of Mycetoporinae stat. nov. is apparently quite different from that of the true tachyporines. In fact, important characters, except for the rather strongly tapered abdomen, are shared between the two subfamilies (see Discussion). Therefore, the removal of Mycetoporini from Tachyporinae sensu nov. is justified to make monophyletic taxonomic groups.

#### 3.2.16. Genus *Bobitobus* Tottenham, 1939 stat. rev.

*Bobitobus* Tottenham, 1939: 225 [140] (Figures 3 and 4, 62A, 64D, 68E and 72B,E; Table A2)

Type species: *Staphylinus lunulatus* Linnaeus, 1767 [94]: 684.

Diagnosis: See Campbell [157] and Li et al. [158].

Composition: Thirty-four species, as listed below.

1. *arcuatus* (Solsky, 1871 [159]: 238), comb. nov. (*Bolitobius*, cited as *Boletobius*). Distribution: Russia (East & West Siberia, Far East), Japan (Hokkaidô), South Korea, China (Jilin).
  = *ohbayashii* (Li & Zhao, 1999 [158]: 250) (*Lordithon*).
2. *cinctus* (Gravenhorst, 1802 [41]: 193), comb. nov. (*Tachinus*). Distribution: Canada, USA.
  = *atricaudatus* (Say, 1823 [160]: 158) (*Tachinus*).
  = *gentilis* (LeConte, 1863 [161]: 31) (*Bolitobius*, cited as *Boletobius*).
3. *copulatus* (Luze, 1902 [162]: 110), comb. nov. (*Bolitobius*). Distribution: Russia (Siberia), Japan.
  = *luzei* (Bernhauer, 1929 [163]: 186) (*Bolitobius*).
  = *hokkaidensis* (Li & Sakai, 1996 [164]: 254) (*Lordithon*).
4. *daimio* (Sharp, 1888 [122]: 456), comb. nov. (*Bolitobius*). Distribution: Japan, South Korea.
5. *distinctus* (Schubert, 1906 [165]: 381), comb. nov. (*Bolitobius*). Distribution: Pakistan, India.
6. *elegantulus* (Li & Sakai, 1996 [164]: 251), comb. nov. (*Lordithon*). Distribution: Japan.
7. *femoralis* (Cameron, 1932 [117]: 339), comb. nov. (*Bolitobius*). Distribution: India (Sikkim, Darjeeling).
8. *fungicola* (Campbell, 1982 [157]: 67), comb. nov. (*Lordithon*). Distribution: Canada, USA.
9. *idahoae* (Hatch, 1957 [143]: 130), comb. nov. (*Lordithon*). Distribution: Canada, USA.
10. *imitator* (Luze, 1901 [166]: 735), comb. nov. (*Bolitobius*). Distribution: Russia (Siberia), Japan (Honshû).
  = *hosodai* (Katayama & T. Ito, 2010 [167]: 296) (*Lordithon*).
11. *indubius* (Luze, 1901 [166]: 734), comb. nov. (*Bolitobius*). Distribution: Russia (Siberia).
12. *irregularis* (Weise, 1877 [168]: 93), comb. nov. (*Bolitobius*). Distribution: Japan, China.
13. *kantschiederi* (Bernhauer, 1915 [169]: 268), comb. nov. (*Bolitobius*). Distribution: Uzbekistan.
14. *kelleyi* (Malkin, 1944 [170]: 26), comb. nov. (*Bolitobius*, cited as *Boletobius*). Distribution: Canada, USA.
  = *angularis* (Sachse, 1852 [171]: 122) (*Bolitobius*, cited as *Boletobius*) [preoccupied, nec. Stephens [142]: 173 (*Bolitobius*)].
  = *bimaculatus* (Couper, 1865 [172]: 61) (*Bolitobius*, cited as *Boletobius*) [preoccupied, nec. Schrank [173]: 644 (*Staphylinus*)].
15. *longiceps* (LeConte, 1863 [161]: 32), comb. nov. (*Bolitobius*, cited as *Boletobius*). Distribution: Canada, USA.
  = *elefas* (Bernhauer, 1912 [174]: 681) (*Bolitobius*).
16. *lunulatus* (Linnaeus, 1767 [94]: 684), comb. nov. (*Staphylinus*). Distribution: Europe, Russia, Caucasus.
  = *atricapillus* (Fabricius, 1775 [175]: 267) (*Staphylinus*).
  = *austriacus* (Schrank, 1781 [176]: 237) (*Staphylinus*).
  = *atricapillus* (Zetterstedt, 1828 [177]: 65) (*Tachinus*) [preoccupied, nec. Fabricius [175]: 267 (*Staphylinus*)].
17. *maacki* (Solsky, 1871 [159]: 236), comb. nov. (*Bolitobius*, cited as *Boletobius*). Distribution: Russia (East Siberia).
18. *nigricollis* (J. Sahlberg, 1880 [178]: 104), comb. nov. (*Bolitobius*). Distribution: Russia (Siberia).
  = *lgockii* (Bernhauer, 1928 [179]: 13) (*Bolitobius*).
19. *niponensis* (Cameron, 1933 [180]: 170), comb. nov. (*Bolitobius*). Distribution: Japan, China.
20. *notabilis* (Campbell, 1982 [157]: 76), comb. nov. (*Lordithon*). Distribution: Canada, USA.
21. *obsoletus* (Say, 1832 [181]: 51), comb. nov. (*Tachinus*). Distribution: Canada, USA.
  = *sellatus* (Sachse, 1852 [171]: 122) (*Bolitobius*, cited as *Boletobius*).
22. *oregonus* (Campbell, 1982 [157]: 82), comb. nov. (*Lordithon*). Distribution: Canada, USA.
23. *praenobilis* (Kraatz, 1879 [182]: 121), comb. nov. (*Bolitobius*). Distribution: Russia (Siberia).
24. *principalis* (Sharp, 1888 [122]: 456), comb. nov. (*Bolitobius*). Distribution: Japan.
25. *pulchellus* (Mannerheim, 1830 [137]: 64), comb. rev. (*Bolitobius*). Distribution: Europe, Ukraine, Russia (European, East Siberia, Far East).
26. *puncticeps* (Luze, 1901 [166]: 733), comb. nov. (*Bolitobius*). Distribution: Russia (Siberia), China (Heilongjiang).
27. *quaesitor* (Horn, 1877 [114]: 119), comb. nov. (*Bolitobius*). Distribution: Canada, USA.
  = *rostratus* (LeConte, 1863 [161]: 32) (*Bolitobius*, cited as *Boletobius*) [preoccupied, nec. Motschulsky [183]: 573 (*Bolitobius*)].
28. *rostratus* (Motschulsky, 1860 [183]: 573), comb. nov. (*Bolitobius*, cited as *Boletobius*). Distribution: Russia (Siberia), Armenia, Turkey.
29. *ruficeps* (Bernhauer, 1938 [184]: 20), comb. nov. (*Bolitobius*). Distribution: China (Manchuria).
30. *semirufus* (Sharp, 1888 [122]: 457), comb. nov. (*Bolitobius*). Distribution: Japan, South Korea, China.
31. *speciosus* (Erichson, 1839 [145]: 277), comb. nov. (*Bolitobius*, cited as *Boletobius*). Distribution: Europe, Russia (European, Siberia).
32. *takashii* (Katayama & T. Ito, 2010 [167]: 294), comb. nov. (*Lordithon*). Distribution: Japan.
33. *vandykei* (Dethlefsen, 1946 [185]: 71), comb. nov. (*Bolitobius*). Distribution: Canada, USA.
34. *variegatus* (Bernhauer, 1902 [186]: 698), comb. nov. (*Bolitobius*). Distribution: Turkey.

Notes on generic name: See details in Blackwelder ([104]: 79).

Remarks: The genus *Lordithon* has long been divided into two subgenera, namely *Lordithon* sensu str. and *Bobitobus* [21,70,157,158,187]. The latter contains far fewer species and has a relatively limited distributional range, basically limited to the Holarctic Region (Table A2). The subgenus *Bobitobus* was mainly defined by the distinctly elongate head capsule and longer tempora, as well as some other characters of the head and antennae [158]. Nevertheless, such subgeneric division had later been rejected by Schülke [188]. He synonymized the subgenus under *Lordithon* without formal analysis because *Bobitobus* can merely be recognized by its ‘elongate’ head capsule and variations found in the entire *Lordithon*, considering there is only weak evidence in support of the validity of maintaining it as a distinct subgenus [188].

Interestingly, however, in my phylogenetic analyses, *Lordithon* sensu str. (*Lordithon thoracicus thoracicus*) and *Bobitobus* (*B. lunulatus* comb. nov. = *L. lunulatus*) were found to be distant from each other, scattered among the other ‘higher’ mycetoporine taxa (Figures 2–4), although major nodes of Mycetoporinae stat. nov. were not supported by the bootstrap support values, and the study design was not aimed for clarifying the internal relationship of this newly recognized subfamily. Therefore, it is noted that this relationship needs to be further tested with more *Lordithon* species in phylogenetic analysis. Compared to the non-monophyly of the genus *Lordithon*, the Oriental and Nearctic *Bryoporus* species were united into a single clade in the resulting tree (Figures 2–4).

Nevertheless, there are several morphological evidences of the distinctness of *Bobitobus* found in this study. It is noteworthy to mention here that I could not find any trace of ocular seta near the base of the eyes on the dorsal head surface of *Bobitobus* in the specimens deposited in the FMNH. As far as I know, the presence of these setae is a universal condition among Mycetoporinae stat. nov. (Figure 64A–C), but there is no trace of such setae in *Bobitobus* (Figure 64D). Nevertheless, a few rare cases in *Lordithon* sensu str. have been known that such ocular setae are absent or nearly absent as in *Lordithon lewisi* (Cameron, 1933) [158,180]. Furthermore, the structure of maxillary palpi between *Bobitobus* and *Lordithon* is also different (Figures 68D,E). They are moderately elongate and pubescent with somewhat thick maxillary palpomeres 3 and 4 in *Lordithon* sensu str. (Figure 68D), whereas these segments are nearly glabrous and slender, much narrower in *Bobitobus* than the conditions seen in *Lordithon* sensu str. (Figure 68E). The general shape of maxillary palpomeres 4 is also different; it is rather strongly narrowed toward apex from the base in *Lordithon* sensu str. (Figure 68D: 33-0), but the palpomere is sub-parallel sided in posterior half in *Bobitobus* (Figure 68E: 33-1; see also [157,158]). Of note, the general shape of labial palpus distinctly differs between them. The labial palpomere 1 is elongate as long as the apical palpomere in *Lordithon* sensu str. ([157]: Figure 50), while it is transverse and much shorter than the terminal one in *Bobitobus* ([157]: Figure 49). Additionally, the labial palpomere 3 can be clearly differentiated from each other as it is much narrower than the palpomere 2 in *Lordithon* sensu str. ([157]: Figure 50), but it is much larger and longer, as wide as the penultimate palpomere in *Bobitobus* ([157]: Figure 49).

In the present phylogenetic analyses, the type species of *Lordithon* sensu str. (*L*. *thoracicus*) formed a sister group with *Bryophacis* (*Bryophacis smetanai* Campbell, 1993 [33]) by moderate branch support (Figure 3). This relationship was supported by four homoplasious synapomorphies (Figure 4): 6-1, head with thin and short ocular seta; 32-0, maxillary palpomere 3 moderately pubescent, with less than 20 setae (> 10); 101-0, apical metatibial spurs short, less than 2/5 of metatarsomere 1; 147-1, paramere with less than five setae. *Bryophacis*, however, is easily separated from *Lordithon* sensu str. by having the more densely pubescent maxillary palpomeres 2 and 3, and the shorter maxillary palpomere 4 in comparison with the penultimate palpomere [16,33].

Based on the evidence discussed above, I remove *Bobitobus* stat. rev. from the synonymy of the genus *Lordithon* sensu nov. and raise it herein to generic rank. Although the intermediate shape of the head capsule has been known in some *Bobitobus* [157], as well as the absence of ocular setae rarely in some *Lordithon* sensu str. [158]. The combination of the discussed characters is useful in recognition of these two taxa as far as I know. Future investigations are particularly needed to assess if these generic assignments are correct for the non-type species of both genera. It would be also desired to investigate whether further generic separation (e.g., South American taxa [188]) from *Lordithon* sensu nov. is plausible or not.

### 3.3. Identification Keys

#### 3.3.1. Key to Subfamilies of Tachyporine Group of Subfamilies

1. Body various, sometimes distinctive; antennae inserted to vertex, posterior to anterior margins of each eye ([189]: Figure 1.13.1); elytra with posterolateral margins frequently sinuate (cf. Figures 25A and 30C: 83-1, 83-2); elytral epipleural keel absent; male paramere with velum [190,191]; female without gonocoxites (except Gymnusini) … Aleocharinae

- Body narrowly elongate, fusiform, or sublimuloid; antennae inserted to anterior margins of each eye; elytra with posterolateral margins rarely sinuate; elytral epipleural keel present OR absent; male paramere simple, lacking velum; female with well-sclerotized gonocoxites … 2

2. Pronotal postcoxal process separated basally by a suture ([16]: Figure 63.22) … Olisthaerinae

- Pronotal postcoxal process not separated basally by a suture … 3

3. Antennae extremely slender and verticillate ([189]: Figures 1.22.1, 9.21.1); neck constriction present, strong, distinct all around ([191]: Figure 1.22.1); maxillary palpus 5-segmented, maxillary palpomere 4 (*mp4*) large and spindle-shaped, mp5 minute and aciculate ([28]: Figure 75) … Trichophyinae

- Antennae with variations, rarely extremely slender and verticillate (Habrocerinae only; [189]: Figure 1.21.2); neck constriction present OR absent ([191]: Figure 1.21.3); maxillary palpus 4-segmented … 4

4. Neck with strong constriction, distinct all around; elytral epipleural keel absent … Phloeocharinae

- Neck without constriction (sometimes with narrowing); elytral epipleural keel present (Figure 52C: 80-0) … 5

5. Metacoxa uniformly covered with extended ventral lamella (or plates, *vlmtc*; Figure S3B) … 6

- Metacoxa without OR with small ventral lamella (see Supplementary Figures S2 and S3A) … 7

6. Antennae extremely slender and verticillate (*Habrocerus*) OR fili-moniliform (*Nomimocerus*) [189,192]; male genitalia modified to complex structure [192] … Habrocerinae

7. Body fusiform, slender (Figures 62 and 63); head with distinct and complete ridge below eye (Figure 67A, C: 13-2); elytron with longitudinally raised sutural edge (Figures 65C and 66A: 78-1); metacoxae extremely large (Figure 70A); male parameres widely separated from each other, setose (Figure 72D–F) … Mycetoporinae stat. nov.

- Body usually sublimuloid, except *Derops* (Figure 43A,B); head without ridge below eye (Figure 26A: 13-0) (rarely with short, incomplete ridge: *Leucotachinus* and *Nepaliodes*; Figures 53A and 58D: 13-1); elytron without longitudinally raised sutural edge (Figure 7D: 78-0); metacoxae medium to small (Figure 52B); male parameres very closely appressed to median lobes (Figure 33E,G), glabrous (*Tachinomorphus* with minute setulae; Figure 56C: 148-2) … Tachyporinae sensu. nov.

#### 3.3.2. Key to Tribes of Tachyporinae

1. Habitus distinctive (Figure 43A,B); pronotum strongly constricted in posterior half (Figure 44A: 49-1) … Deropini

- Habitus usually sublimuloid; pronotum not constricted in posterior half… 2

2. Elytra pubescent (Figures 7B and 15C: 74-2); antenna basally without clear borderline of dense and fine recumbent setae (Figure 9D: 21-0); maxillary palpomere 3 setose, longer than palpomere 4 (Figures 10A–D and 16E: 32-1) … Tachyporini sensu nov.

- Forebody glabrous (not covered with long ground setae); antenna basally with clear borderline of dense and fine recumbent setae (Figures 26D and 59C: 21-1, 21-3); maxillary palpomere 3 glabrous (except *Mimocyptus*, *Leucotachinus*), shorter than palpomere 4 (except *Mimocyptus*, *Vatesus*) (Figures 27A, 45D, 53C and 59A: 32-2, 32-3, 34-2) … 3

3. Antennae longer than width of pronotum; elytron much longer than wide (length/width: >1.3×); pronotal hypomeron with long and projecting postcoxal process (Figures 54G and 59D,E: 61-5); male sternite VII (*s7*) with posterior margin emarginate, bearing peg-like setae (most) or short and stout setae (Figures 52D, 55A and 61A: 131-1, 131-2) … Tachinusini stat. rev., sensu nov.

- Antennae usually shorter than width of pronotum (except *Cilea*, *Cileoporus*, and *Tachinoporus*) (Figure 23B: 20-1); elytron usually slightly longer than wide (length/width: <1.3×, except *Cilea*, *Cileoporus*, and *Tachinoporus*) (Figure 30A,B: 70-2); pronotal hypomeron with short and rounded postcoxal process (Figure 27E–G: 61-1); male sternite VII with posterior margin usually truncate (occasionally emarginate, i.e., *Cilea*), without peg-like setae or stout setae (Figure 25C: 131-0) … Vatesini sensu nov.

#### 3.3.3. Key to Subtribes and Extant Genera of Tachyporini

1. Forebody densely pubescent (Figure 15B, C); protibia with a row of comb-like close-spaced spines along outer margin (Figure 17B,C: 97-1); pronotum without arranged macrosetae; metatarsus very long, much longer than whole length of metatibia (Figure 15D: 103-1); abdomen without paratergites (Figure 15D: 124-2) … 2 (Euconosomatina stat. rev., sensu nov.)

- Forebody not entirely pubescent, usually glabrous on head and pronotum (Figure 7A–D); protibia without a row of comb-like close-spaced spines along outer margin; pronotum with arranged macrosetae (Figures 7A and 8A); metatarsus long, but clearly shorter than whole length of metatibia (Figure 7E: 103-0); abdomen with single pair of paratergites (Figure 7E: 124-0) … 3 (Tachyporina stat. nov., sensu nov.)

2. Labial palpomere 3 distinctly expanded, crescent-shaped ([44]: Figure 1) … *Euconosoma*

- Labial palpomere 3 not expanded, conical (Figure 16F) … *Sepedophilus*

3. Maxillary palpomere 3 widest at or near apex (Figure 10C,D: 30-0) … 4

- Maxillary palpomere 3 widest around middle, not at or near apex (Figure 10A,B: 30-1) … 5

4. Body strongly convex in cross-section (Figure 6B); antenna modified, distinctly short, as long as head width (Figures 7C and 9C: 19-2, 20-1) … *Lamprinus*

- Body not strongly convex in cross-section (Figures 6A and 7D); antenna less modified, much longer than head width (Figure 6A) … *Lamprinodes*

5. Metatarsomere 3 with ventrally modified projection ([22]: Figure 18); pronotum widest between basal 1/5 and basal 1/4 (Figure 8A: 50-2); elytra with long and thick macrosetae (Figure 8A,D) …*Symmixus*

- Metatarsomere 3 normal, without ventrally modified projection; pronotum not widest between basal 1/5 and basal 1/4; elytra with inconspicuous macrosetae (Figure 7B) … 6

6. Body small (length: <1.4 mm [61]), narrow, very slender (Figure 6C); maxillary palpomere 4 short, but very thick, widened at base (Figure 10B and Supplementary Figure S1A); pronotum widest between basal 1/4 to middle (Figure 7B: 50-3); elytron without epipleural gutter along outer margin (Figure 7B: 79-0) … *Palporus* stat. nov.

- Body small to medium (length: ≥1.4 mm), somewhat slender (Figure 6F); maxillary palpomere 4 narrowly elongate, much narrower (Figure 10A); pronotum widest between base and basal 1/5; elytron with very narrow and inconspicuous epipleural gutter along outer margin (cf. Figure 7D: 79-1) … *Tachyporus* sensu nov.

#### 3.3.4. Key to Extant Genera of Vatesini

1. Elytron moderately elongate (length/width: 1.3–1.8×, if dissected) (Figure 21B); pronotum widest between basal 1/4 to middle (Figure 37B) … 2

- Elytron only slightly elongate (length/width: <1.3×, if dissected) (Figure 30A,B); pronotum widest between base and basal 1/5 … 4

2. Body very slender, streamlined (Figures 21B and 36C); abdominal terminalia with extremely long macrosetae (Figures 21B and 39A–E); tergite VIII and sternite VIII narrowly elongate, with short and indistinct lobes (if present, see Figure 39A,D,E); male tergite IX with each apex elongate, not modified to two lobes ([91]: Figure 62) … 3

- Body not slender, sublimuloid (Figure 21A); abdominal terminalia without extremely long macrosetae (Figures 31A and 32A,C); tergite VIII and sternite VIII only weakly elongate to transverse, with long and distinct lobes (Figures 31A, 32A, C); male tergite IX with each apex modified to two lobes (Figure 33A: 122-3) … *Cilea*

3. Pronotum and elytra with erect macrosetae (Figures 37B and 38A–C); pronotal disc with shallow and very narrow longitudinal sulcus along midline (Figure 37B, *lsp*); distributed in Oriental region… *Tachinoporus*

- Pronotum and elytra without erect macrosetae (Figure 21B); pronotal disc without longitudinal sulcus along midline; distributed in Central and South America … *Cileoporus*

4. Antennae and legs with distinct modifications (Figures 26G and 29B,D: 19-2, 93-1) … *Vatesus*

- Antennae and legs without distinct modifications … 5

5. Body minute (length: ca. 1.2 mm) (Figure 21G); midcranial suture absent (Figure 23A: 11-0); male tegite IX with basal 1/3 dorsally contiguous (Figure 33C: 120-0) … *Mimocyptus*

- Body small to large, not minute; midcranial suture present (Figure 23B: 11-1) … 6

6. Forebody matte, not glossy (Figures 40C–E and 41D,E); mesoventrite with extremely large plate-like keel (Figure 40E,F, *pkmsv*); abdomen in basal half without blackish macrosetae (Figure 40C); male aedeagus with conspicuous spines (Figure 42B, dsp) … *Tachinoproporus*

- Forebody glossy; mesoventrite without plate-like keel; abdomen in basal half with blackish macrosetae (Figure 21C–F,H); male aedeagus without spines … 7

7. Body strongly convex dorsally (Figure 21H); head much smaller than pronotum; antennae shorter than width of head (Figure 23B) … *Termitoplus*

- Body not strongly convex dorsally (Figure 21C–E); head moderately smaller than pronotum; antennae longer than width of head (Figure 21C–F) … 8

8. Body rather flattened dorsally (Figure 21E); forebody with bluish metallic sheen; mesoventrite without any trace of longitudinal carina (Figure 24B: 85-0); distributed only in Africa … *Coprotachinus*

- Body moderately convex dorsally (Figure 21C,D); forebody without bluish metallic sheen; mesoventrite with weakly raised longitudinal carina (Figures 24C, 28B: 85-1); distribution cosmopolitan … *Coproporus*

#### 3.3.5. Key to Extant Genera of Tachinusini

1. Head and pronotum with deep, pit-like punctation (Figure 58A–C); clypeal anterior margin weakly to strongly reflexed upward (Figure 58A–C: 7-1, 7-2) … 2 (members of the former Megarthropsini)

- Head and pronotum without deep, pit-like punctation (Figure 51); clypeal anterior margin not reflexed upward (Figure 50: 7-0) … 5

2. Habitus distinctive (Figure 57D, E), with highly and very strongly extended pronotal and elytral margins (Figure 58); antenna with apically tapered scape (Figure 58B,C) … *Nepaliodes*

- Habitus less distinctive (Figure 57A,C,F), with slightly to strongly extended pronotal and elytral margins (Figure 58A); antenna with subparallel-sided scape (Figure 59C) … 3

3. Metaventrite with intermesocoxal pit adjacent to apex of mesosternal process (Figure 60A, B: 89-1) … *Lacvietina*

- Metaventrite without intermesocoxal pit … 4

4. Head with postocular vertical carina (Figure 58D: 13-1); antennae long, very slender, reaching beyond posterior margin of elytra (Figure 57C) … *Megarthropsis*

- Head without postocular vertical carina; antennae short, slender, reaching only to near middle of elytra (Figure 57F) … *Peitawopsis*

5. Abdomen setose with macrosetae on tergites IV–VII (Figure 48D); head without any type of neck or narrowing (Figure 51A: 12-0) … *Nitidotachinus*

- Abdomen usually without macrosetae on tergites IV–VII; head with neck or narrowing (Figures 50 and 51B,D: 12-3) … 6

6. Elytron with distinct rows of punctures (Figures 48F, 51D); male tergite IX with each apex distinctly modified to two lobes (Figure 55F: 122-3) … *Olophrinus*

- Elytron usually without distinct rows of punctures (Figure 51C); male tergite IX with each apex not modified to two separate lobes (Figure 55E, G) … 7

7. Elytron long, concealing more than half of abdomen (Figure 48B,C); head with a pair of ridges basolaterally along underside of eyes (Figure 53A: 13-1); mesoventrite with transverse ridge, lying near base of intercoxal process (Figure 52A and 54E: 86-1) … *Leucotachinus*

- Elytron moderately long, not concealing half of abdomen; head without a pair of ridges basolaterally along underside of eyes; mesoventrite without transverse ridge … 8

8. Elytra covered with reduced, modified setae (Figure 48A); ligula with pair of medial lobes ([110]: Figure 2); distributed in Australia … *Austrotachinus*

- Elytra not covered with reduced, modified setae; ligula without pair of medial lobes; distribution nearly all outside Australia … 9

9. Pronotum widest at base (Figures 49C and 51C); main part of mesoventrite with strongly raised longitudinal carina (Figures 52C and 54C: 85-1); male parameres densely covered with minute sensilla or filiform setulae (Figure 56C: 148-2) … *Tachinomorphus*

- Pronotum widest between basal 1/4 to middle (Figure 51B: 50-3); main part of mesoventrite without strongly raised longitudinal carina (Figures 52B and 54F: 85-0); male parameres without minute sensilla … 10

10. Head with narrow furrows along inner edge of eyes (Figures 50B and 51B); pronotum with furrows near anterolateral margins (Figure 51B: 52-1); mesoventrite with longitudinal carina on mesoventral process and its basal area only ([193]: Figure 2E,F) … *Pseudotachinus*

- Head without narrow furrows along inner edge of eyes; pronotum usually without furrows near anterolateral margins (shallow and inconspicuous, if present) … 11

11. Pronotum with rather strongly sinuate posterior margin (Figure 49F); scutellum markedly enlarged, but with exceptions [194] (Figure 49F); mesoventrite with deep longitudinal furrow along mesoventral process (Figure 52B); male sternite VII with posterior margin very broadly weakly emarginate, bearing inconspicuous modified setae ([194]: Figure 3A,B); distributed in Africa … *Tachinoplesius*

- Pronotum with truncate, rounded, or only weakly sinuate posterior margin; scutellum small to medium (Figure 49D,E); mesoventrite without deep longitudinal furrow along mesoventral process; male sternite VII with posterior margin usually somewhat narrowly strongly emarginate, bearing conspicuous modified setae, frequently furnished with dozens of strong peg-like setae; widespread, but not in Africa … *Tachinus*

#### 3.3.6. Key to Extant Genera of Mycetoporinae

The following key is partly based on Campbell [32,33,157] and Newton et al. [16].

1. Head distinctly elongate, with long tempora, lacking ocular setae (Figures 62A and 64D: 6-0); antenna basally distinctly separated from base of mandible by anterior lobe of gena (Figure 64D); antennomere 1 very long and slender (Figure 64D); maxillary palpomeres 3 and 4 combined long and slender (Figure 68D) … *Bobitobus* stat. rev.

- Head not distinctly elongate, usually only weakly to moderately elongate (Figure 67A,B), with short tempora, mostly having ocular setae (Figure 64A–C: 6-2); antenna basally contiguous with base of mandible (Figure 64A); antennomere 1 less elongate, thicker (Figure 68F,G); maxillary palpomeres 3 and 4 combined usually somewhat shorter and wider (Figure 68A,B,D) … 2

2. Maxillary palpomere 4 narrower and slender, not beyond more than 1/2 as wide as penultimate palpomere (Figure 68C) … 3

- Maxillary palpomere 4 thicker, more than 1/2 as wide as penultimate palpomere (Figure 68A,B,D) … 4

3. Meso- and metatibial apices bordered by ctenidium of evenly arranged dense equal length spines with two larger and one smaller apical spur ([32]: Figures 103–105); antennae long, even longer than head and pronotum combined (Figure 63D); maxillary palpomeres 2 and 3 densely finely pubescent ([32]: figures 38 and 39); scutellum with basal carina linear ([32]: Figures 76, 77) … *Ischnosoma*

- Meso- and metatibial apices bordered by more irregularly arranged numerous unequal spines ([32]: Figures 99–102); maxillary palpomeres 2 and 3 sparsely, coarsely pubescent ([32]: Figures 31–37); antennae shorter than head and pronotum combined (Figure 63F); scutellum with basal carina acutely pointed medially ([32]: Figures 74 and 75) … *Mycetoporus*

4. Meso- and metatibial apices with ctenidium of equal length spines, plus 2–3 long spurs, forming a straight edge ([33]: Figures 103–105) … 5

- Meso- and metatibial apices with numerous unequal spines, forming a jagged edge ([33]: Figures 99–102) … 6

5. Elytron with 5–6 irregular longitudinal rows of setigerous punctures (Figure 65D); male sternite VIII with patch of 2 or 3 pairs of oblique setae near middle of posterior margin (Figure 71F: 134-1); abdomen rather strongly tapered posteriorly (Figure 62F) … *Bryoporus*

- Elytron evenly densely punctate (Figure 65C); male sternite VIII without characteristically arranged microsetae on postero-medial area [152,195]; abdomen weakly tapered posteriorly (Figure 63H) … *Parabolitobius*

6. Elytron evenly densely punctate (Figure 62C); labial palpomere 1 short, strongly transverse ([33]: Figure 91) … *Bolitopunctus*

- Elytron not evenly densely punctate; labial palpomere 1 weakly transverse to elongate … 7

7. Labial palpomere 3 longer than labial palpomeres 1 and 2 combined (Figure 67F) … 8

- Labial palpomere 3 shorter than labial palpomeres 1 and 2 combined (cf. Figure 67E) … 10

8. Antenna with preapical antennomeres broadly transverse ([138]: Figure 8); abdomen sub-parallel sided in basal 2/3 (Figures 63A,C and 66A) … *Carphacis*

- Antenna rather slender, with preapical antennomeres not broadly transverse; abdomen tapering posteriorly … 9

9. Body large (length: 5.6–10.8 mm [16]); antenna without clear micropubescence borderline, density increasing apically; maxillary palpomere 3 pubescent; pronotum and elytra with dense waves of microsculapture visible only under high magnification (above 100x) [16,33]; elytron usually with more than 6 irregular longitudinal rows of setigerous punctures; male sternite VII with distinct patterns of modified setae (Figure 71G: 134-2); distributed in Holarctic region … *Bolitobius*

- Body medium to relatively large (length: <5.6 mm [16,33]); antenna with clear micropubescence borderline, lacking dense and fine recumbent setae on basal four antennomeres ([33]: Figure 104); maxillary palpomere 3 nearly glabrous; pronotum and elytra with distinct waves of coarse, transverse microsculapture easily visible with low magnification (24x) [16,33]; male sternite VII without distinct patterns of modified setae; distributed in North America only … *Neobolitobius*

10. Maxillary palpomere 3 moderately coarsely pubescent ([33]: Figures 99 and 100); maxillary palpomere 4 shorter than penultimate palpomere ([33]: Figures 99 and 100); labial palpomere 1 very large, even larger than labial palpomeres 2 and 3 combined ([33]: Figures 87 and 88); scutellum with basal carina distinctly divided medially ([33]: Figure 130) … *Bryophacis*

- Maxillary palpomere 3 with sparse setae only; maxillary palpomere 4 equal in length or longer than penultimate palpomere (Figure 68D); labial palpomere 1 large, but shorter and smaller than labial palpomeres 2 and 3 combined; scutellum with basal carina obtusely convex in middle ([157]: Figure 59) … 11

11. Head with long, strong ocular setae; elytra with rather conspicuous microreticulation; abdomen sub-parallel sided in basal 2/3; distributed in Canary Islands … *Canariobolitobius*

- Head with rather short, inconspicuous ocular setae (rarely absent, e.g., *L. lewisi* [158]); elytra without microreticulation; abdomen strongly tapering posteriorly; distribution cosmopolitan … *Lordithon* sensu nov.

## 4. Discussion

### 4.1. Non-Monophyly of Tachyporinae and Tachyporini

Among one extinct and 32 extant subfamilies of Staphylinidae [1,20], Tachyporinae are one of the most problematic subfamily-level taxa [3,6,7]. Indeed, this problem has been recognized by earlier studies based on evidence from both adults and larvae [26,27,28], but more comprehensive details have been unknown until recently in the absence of broad taxon sampling of Staphylinidae in phylogenetic analyses. Two recent large-scale phylogenies based on molecular data have provided strong evidence for the polyphyly of Tachyporinae [6,7], and greatly expanded our views on tachyporine phylogeny in spite of their weak gene samplings. Both studies have shown that the subfamily is actually divided into two phylogenetically distant clades: the tribe Mycetoporini is widely separated from the remaining core tachyporine members [6,7]. Although the tachyporines without Mycetoporini appear to be monophyletic, it should be mentioned that neither of these studies included Megarthropsini [6,7]. Furthermore, Vatesini was absent from McKenna et al. [6], preventing the assessment of the true monophyly for the tachyporine clade in sensu stricto.

Here, I focused on Tachyporinae phylogeny and classification for the first time to tackle these issues, incorporating the largest and most comprehensive taxon sampling of tachyporine taxa ever in history. By using museum collections including historical material, it was possible to directly examine all forty extant tachyporine genera (except *Urolitus* syn. nov., literature only), with the addition of two fossil genera. Of these, a total of 57 species in 38 genera of Tachyporinae were used in the phylogenetic analyses. Although my dataset is limited to adult morphology, it provides a backbone to the phylogeny and relationships within the tachyporines. My results were consistent with the hypothesis of a non-monophyletic Tachyporinae, with a similar topology to that obtained by McKenna et al. [6] and Lü et al. [7]: Mycetoporini appearing in a phylogenetically distant position from the rest of the tachyporines (Figures 2–4). Four tribes, namely Deropini, Tachyporini, Megarthropsini, and Vatesini, together formed a monophylum (Figures 2–4). However, non-monophyly of the largest tribe Tachyporini was again shown in the resulting tree and appeared in nine distinct clades (Figure 2). My results suggested that the current classifications of both Tachyporinae and Tachyporini are more or less artificial based on similar-looking taxa, rather than grouped together under a phylogenetic framework.

### 4.2. Revised Status of Mycetoporini as a Subfamily

The present and previous studies consistently demonstrated that Mycetoporini is significantly dissimilar from the remainder of Tachyporinae [6,7]. In light of this, I herein revise the traditional concept of Tachyporinae and exclude all members of Mycetoporini from Tachyporinae. Both lineages are monophyletic and morphologically distinctive, and each should be considered at the subfamily rank. Yet, Mycetoporini cannot be assigned to any of the other subfamilies of Staphylinidae; therefore, it is now raised from a tribal rank to the 34th recognized subfamily Mycetoporinae stat. nov. within Staphylinidae, after following the treatment of Staphylininae and their allies in Tihelka et al. [1].

Overall, morphological features clearly support this treatment. For example, mycetoporines are usually defined by a series of distinctive characters that clearly separate them from Tachyporinae sensu. nov., such as: head usually elongate, lacking midcranial suture, ventral side with distinct ridge below eye; antennal insertion fully exposed; ligula small, restricted to median area; pronotal hypomeron with transverse ridge at apical 1/3 to 1/4 (see Figure 69A,B); elytron frequently with impressed sutural striae, with longitudinally raised sutural edge; metacoxae markedly large; abdomen usually covered with V-shaped setigerous punctures, having intersegmental membranes with ‘brick-wall’ pattern; tergite VIII and sternite VIII without lobe-like modifications; male tergite IX continuous dorsally in basal half, with ventral struts, basally separated by sternite IX; parameres separated from median lobe, each with a row of parameral setae at least partially aligned; gonocoxite II very large in comparison to gonostylus, bearing only normal setae. These features are not at all, or very rarely, found in Tachyporinae sensu. nov. Given the overall topology of my phylogenetic tree and consideration of these morphological characters, this taxonomic treatment can be justified, as previously suggested by Gusarov [196].

### 4.3. New Higher Classification of Tachyporinae

With the exclusion of Mycetoporini from the traditional concept of Tachyporinae, not only the taxonomic treatment of Mycetoporini but also that of the remaining tachyporine clade is now revised. The new concept of this subfamily is unique among the other staphylinid subfamilies in having the following morphological characteristics: head with midcranial suture; ligula transverse and markedly large; tergite VIII and sternite VIII frequently with lobe-like modifications; parameres very closely appressed to median lobe, with apically contiguous to only weakly separated apical inner margins; gonocoxite II small in comparison with gonostylus, bearing curved setae. These features define the tachyporines more accurately than the traditional circumscription, which was merely based on characters such as the more or less tapered abdomens and more or less retractile head.

My analyses and examinations of the specimens resolve the backbone relationships within Tachyporinae sensu nov. The new division of this problematic group significantly improves its classification by naming monophyletic units as tribes, with diagnostic characters for identification. Until the present study, a total of five tribes have been recognized within Tachyporinae, including Mycetoporini. Here, I have incorporated only four tribes into the subfamily: Tachyporini sensu nov. (Tachyporina stat. nov., sensu nov. and Euconosomatina stat. rev., sensu nov.), Vatesini sensu nov., Deropini, and Tachinusini stat. rev., sensu nov. (= Megarthropsini syn. nov.). All but Deropini are now redefined under the new concepts mentioned in detail above.

The monogeneric tribe Vatesini is combined together with the *Coproporus*-related genera of Tachyporini in the traditional sense, and it was fully nested within this clade (Figure 2). Interestingly, however, a close relationship between *Coproporus* and the sole vatesine genus *Vatesus* has been suggested by morphological evidence for both adults and larvae [25,27]. It is notable that *Vatesus* and *Coproporus* were shown to be sister groups supported by four unique synapomorphies based on a dataset comprised of 27 larval morphological characters ([27]: see also Figures 1 and 2 in Ashe [28]). Later, a similar hypothesis was obtained from the morphology-based parsimony analysis using 19 adult characters [25]. *Coproporus* fell into the same clade as *Vatesus*. However, Herman’s study was focused only on Megarthropsini, and he did not intend to examine the tribal relationships within Tachyporinae ([25]: 64). Nevertheless, he mentioned the close similarity of adult morphologies between *Coproporus* and *Vatesus* by removing the autapomorphic features of *Vatesus* acquired through their adaptative evolution with army ants ([25]: 65). Consequently, my result is not very surprising, but the more detailed comparison made here provides firm evidence for supporting this conclusion.

Another small tribe, Megarthropsini, is no longer considered as a valid tribe in this study. Herman [25] made a comprehensive revision of Megarthropsini and also provided a preliminary phylogenetic hypothesis. His phylogenetic analysis recovered Megarthropsini as a monophyletic tribe with Deropini as its sister group. I agree with the opinion that the megarthropsines are distinct among other tachyporines in having raised and explanate lateral margin of the frons, pronotum or elytra and aside from these characters, there are several additional characteristic features that are uniquely found in the tribe [25]. According to Herman [25], an important character state that supports Megarthropsini and Deropini as sister groups is the presence of a broad, distinct neck. This state, however, is also seen in the Tachinus-related genera (e.g., Figure 51B,D). Thus, this proposed relationship is less strongly supported than Herman suggested. Indeed, most characters and even the general habitus of the megarthropsines agree well with those of the *Tachinus*-related genera. My phylogenetic analyses have shown the monophyly of Megarthropsini, but it was resolved as a part of the former Tachyporini (*Tachinus*-related genera), reflecting the morphological similarities between them. Consequently, the tribal status should no longer be maintained; and thus, Megarthropsini syn. nov. is now synonymized under Tachinusini stat. rev., sensu nov.

Tachyporini in the traditional sense has been the most problematic tribe among the tachyporine tribes; it has been inferred to be paraphyletic without any synapomorphy for a long time. Such a conclusion was drawn from both morphological and molecular evidence [6,7,25,27,28,196]. Compared to above mentioned Ashe & Newton [27] and Herman [25], more data were used in Ashe [28] to investigate the internal relationships within the Tachyporine Group of subfamilies based on 133 adult characters and 27 larval characters. In his study, both Tachyporinae and Tachyporini were not supported to be monophyletic [28]. More recently, two molecular-based studies have demonstrated such paraphyly in Tachyporini with respect to Deropini [6] or Tachyporini and Vatesini [7]. Only two molecular markers, i.e., 28S ribosomal RNA and the nuclear protein-coding carbamoyl-phosphate synthase domain (CAD), were used in McKenna et al. [6], whereas six genes were analyzed in Lü et al. [7]: two nuclear protein-coding genes, CAD and wingless (Wg); two nuclear non-coding genes, 28S and 18S rDNA; two mitochondrial genes, one coding Cytochrome b (Cyt b) and one non-coding 16S rDNA. The results of both studies on the tachyporines resemble each other, although that of McKenna et al. [6] should be considered weakly supported in this respect. Nonetheless, these studies targeted various phylogenetic and evolutionary questions within Staphylinoidea or Staphyliniformia, and one can conclude that no study had actually focused on the monophyly of Tachyporini previously. After my thorough study and reclassification, Tachyporini is now treated in a strictly limited sense. Tachyporini sensu nov. contains only eight genera (one extinct) with two newly assigned subtribes: Tachyporina stat. nov., sensu nov. and Euconosomatina stat. rev., sensu nov. Considering the distinctive features of *Sepedophilus* and its relatives (e.g., absence of paratergites), it seems reasonable to apply these subtribal divisions. The remaining members of the former Tachyporini were placed in different clades in my analyses and therefore in other tribes, producing a reclassification of the subfamily. Of note, the monophyly of Tachinusini stat. rev., sensu nov. as defined here was not supported with high support by the molecular analyses of McKenna et al. [6] as *Tachinus* was separated from the *Leucotachinus* + *Austrotachinus* clade. Nevertheless, the study design of McKenna et al. [6] was not at all aimed at Tachyporinae, more specifically the traditional sense of Tachyporini, with only two molecular genes.

With the revised tribal classification of Tachyporinae sensu nov., the long-standing problem of the circumscription of the subfamily and tribal concepts is revisited. There are still several small topics to work on. For example, the next immediate targets are revisiting the systematic validity and placements of the fossil taxa, especially those of the compression fossils. Additionally, phylogenetic assessment of *Lordithon* sensu nov. and *Bobitobus* stat. rev. in Mycetoporinae stat nov. and the subgenera of *Tachinus* (Tachinusini stat. rev., sensu nov.) are also important tasks. I hope that the new classification will be a backbone to Tachyporinae systematics and applied to evolutionary and ecological studies.

### 4.4. Sister Group of Tachyporinae

Although Tachyporinae have been placed in the Tachyporine Group for a long time, it has been a great challenge to identify a potential sister taxon of Tachyporinae with much confidence, as the subfamily had seemed to be polyphyletic. Consequently, Thayer [3] summarized a more or less ambiguous phylogenetic schema for the Tachyporine group based on previously published literature due to the ‘non-monophyletic’ Tachyporinae.

Interestingly, two recent molecular phylogenetic studies suggested the carrion beetle family Silphidae as a possible candidate for the sister group of Tachyporinae sensu str. [6,7]. In McKenna et al. [6] where Tachyporinae sensu str. was hypothesized to be a close relative of Silphidae, but neither were closely related to the remaining Tachyporine Group of subfamilies, namely: Aleocharinae, Habrocerinae, Olisthaerinae, Phloeocharinae, and Trichophyinae. The clade Tachyporinae sensu str. + Silphidae was resolved in a cluster comprised of eleven other staphylinid subfamilies which included Aleocharinae [6]. In contrast, Tachyporinae sensu str. formed a clade together with Silphidae and altogether were sister to Phloeocharinae (*Phloeocharis*) in Hunt et al. [197] and Lü et al. [7]. A recent phylogenomic analysis recovered a slightly different phylogeny by analyzing 95 protein-coding genes in 373 beetle species, with limited staphylinoid taxa [198]: Tachyporinae sensu str. formed a sister group to the clade Silphidae + the Oxyteline Group of subfamilies (Apateticinae, Scaphidiinae, and Osoriinae). A close affinity between Silphidae and the Oxyteline Group had earlier been refuted by Grebennikov & Newton [2] based on morphology, but this subfamily group should also be studied in the future as a possible alternate sister taxon to the silphids.

In my analyses, the Tachyporinae sensu str. + Silphidae clade was corroborated by one unique synapomorphy and eight homoplasious synapomorphies (Figure 4), and this lineage was widely separated from Phloeocharinae (*Phloeocharis*), fully consistent with the hypothesis of McKenna et al. [6]. Despite my results and the several previous works mentioned above, the close relationship with the silphids should be carefully assessed in the future by analysing broader taxonomic sampling and larger datasets. More assessment is needed to address this issue and to determine whether to incorporate Silphidae into Staphylinidae as a separate rove beetle subfamily.

### 4.5. Sister Group of Mycetoporinae

The newly recognized subfamily Mycetoporinae stat. nov. is apparently monophyletic, but with uncertainty about its sister taxon with a handful recent studies assessing its phylogenetic position [6,7,196]. Indeed, McKenna et al. [6] showed conflicting phylogenetic relationships between the Bayesian and maximum likelihood analyses. In the Bayesian inference tree of McKenna et al. ([6]: Figure 3C), Mycetoporinae stat. nov. formed a sister group with Pseudopsinae (with weak support), subsequently followed by Micropeplinae [6]; these together formed the sister group of the rather unresolved large cluster of subfamilies including the clade Olisthaerinae + (Paederinae + Staphylininae). By contrast, in the maximum likelihood phylogram ([6]: Figure 4B), they recovered the mycetoporines and Pseudopsinae as sister taxa (with weak support), sister to Olisthaerinae (with no support) and all of them sister to Paederinae + Staphylininae (with no support). On the other hand, Lü et al. [7] recovered a similar, but slightly different, topology from the maximum likelihood tree of McKenna et al. [6]: Mycetoporinae stat. nov. was resolved as having a sister group relationship to the grade formed by Olisthaerinae, altogether sister to Paederinae + Staphylininae, although all those nodes had bootstrap support values far below 50.

In congruence with Lü et al. [7], I have recovered generally similar relationships within the selected staphylinid and silphid taxa, resolving Mycetoporinae stat. nov. and Olisthaerinae as sister taxa with moderate support (Figure 3). Staphylininae were recovered as sister to Mycetoporinae stat. nov. and Olisthaerinae combined, fully consistent with Lü et al. [7], though with no support. Overall, the general morphological features of Mycetoporinae stat. nov. are much more similar to Staphylininae or Olisthaerinae than to Tachyporinae sensu nov. or Pseudopsinae, regardless of the notable morphological differences between Staphylininae and Olisthaerinae, particularly based on the structures of mouthparts, prothorax including prosternum, scutellum, elytra, and metacoxae. Given that there are only a few previous studies supporting the close similarity among the mycetoporines, Staphylininae (plus Paederinae), and Olisthaerinae, my results require further data to support this hypothesis; and thus, it should be considered as preliminary and tentative. Yet, these subfamilies will certainly stand as the most plausible candidates for the sister taxon of Mycetoporinae stat. nov.

### 4.6. Evolutionary Origins and Fossil Records

My updated higher phylogeny and classification of Tachyporinae also triggered substantial changes for evolutionary origins in each taxonomic category. In this study, I reconsider the taxonomic placements of all extinct tachyporine genera as listed above in the Taxonomy section, following such taxonomic changes.

No staphylinid fossil records earlier than the Jurassic nor molecular dating supporting the existence of the tachyporines in the Triassic have been found [7]. On the basis of currently available information, the evolutionary origin of Tachyporinae sensu. nov. can reliably be dated back to at least the Late Jurassic, but based on some problematic or undescribed fossils, it likely dates as far back as the Middle Jurassic, while that of Mycetoporinae stat. nov. can now be traced back to the Lower Cretaceous ([153,154]; see above).

Within Tachyporinae sensu. nov., only a single tribe, Deropini, currently lacks any fossil record at the tribal level, while the three other tachyporine tribes have both Mesozoic and Cenozoic fossils. Note that Tachinusini stat. rev., sensu nov. putatively contains a likely erroneously identified Middle Jurassic fossil specimen of ‘†*Protostaphylinus mirus*’, as the oldest tachyporine, with some undescribed fossils from the Middle Jurassic Daohugou, northeastern China [51]. Regardless of the systematic positions of these fossils, Tachinusini stat. rev., sensu nov. indeed has a deep evolutionary origin as indicated by the discovery of †*Protachinus* from the Upper Jurassic of Australia [52]. My phylogenetic analyses and observations provide strong support for Deropini and Tachinusini stat. rev., sensu nov. being sister groups. If correct, then the evolutionary history of Deropini may be dated back to the Late or Middle Jurassic as well. By contrast, the remaining two tachyporine tribes have much shallower geological origins based on fossils, but older examples are presumably just undiscovered, based on deep nodes of the phylogeny presented here. Vatesini sensu nov. has only a single Mesozoic genus †*Procileoporus* from mid-Cretaceous Kachin amber from northern Myanmar [46]. Given the uncertainty associated with the paucity of fossil records for the vatesines, more discoveries of both impression and amber fossils are desired in spite of their difficulty in identification. With only a single Mesozoic occurrence, Tachyporini sensu nov. also has poor fossil evidence, represented by the extant genus *Tachyporus* (Tachyporina stat. nov., sensu nov.) from Upper Cretaceous New Jersey amber from the USA ([56]; this study). Nevertheless, Tachyporini sensu nov. or even Euconosomatina stat. rev., sensu nov. will still possibly be found from Burmese (Kachin) amber in the near future considering the enormous and significant fossil records of rove beetles from this deposit. If my phylogenetic hypothesis and its major branching pattern is reasonable, the evolutionary origins of both Vatesini sensu nov. and Tachyporini sensu nov. could be much older, probably tracing back to the Late or Middle Jurassic.

In conclusion, I posit that every tribe-level lineage of Tachyporinae sensu nov. was already established by the Late Jurassic, and the subfamily likely must have originated during the Middle or possibly Early Jurassic.

## 5. Conclusions

From the phylogenetic analyses and morphological investigations representing all fossil and extant genera, the following conclusions can be drawn:

1. Non-monophyly of current Tachyporinae and Tachyporini is again supported. To improve the higher classification of this subfamily, Mycetoporini is removed from Tachyporinae and considered as a newly recognized rove beetle subfamily Mycetoporinae. I reclassify Tachyporinae into four monophyletic tribes: Tachyporini, Vatesini, Deropini, and Tachinusini. The tribe Megarthropsini is synonymized under Tachinusini. Within Tachyporini, two subtribes are newly established: Tachyporina and Euconosomatina. The resulting topology is as follows: Tachyporini + (Vatesini + (Deropini + Tachinusini)).
2. Most tachyporine and mycetoporine genera, including disjunctly distributed *Leucotachinus*, are found to be monophyletic, except *Lordithon*, *Sepedophilus*, and *Tachyporus*. With some taxonomic changes, all genera of both subfamilies appear to be monophyletic for now. After direct examination of the two enigmatic extant genera *Tachinoporus* and *Tachinoproporus*, both are indeed true tachyporines and should be considered valid genera. A total of 36 (†7) genera of Tachyporinae and 16 (†4) genera of Mycetoporinae are recognized here.
3. The carrion beetle family Silphidae (*Silpha*) and Tachyporinae might be sister groups, whereas Mycetoporinae forms a sister group relationship with Olisthaerinae. These results support the polyphyly of the Tachyporine Group of subfamilies consistent with several previous studies (e.g., [6,7,196]). Nevertheless, further study is needed to corroborate these hypotheses.
4. Re-examination of fossil records of both Tachyporinae and Mycetoporinae dramatically changes the known evolutionary history of each taxon: Tachinusini (~ Upper Jurassic, but likely Middle Jurassic [51]), Vatesini (~ mid-Cretaceous), and Tachyporini (~ Upper Cretaceous). Deropini so far has no fossil record, but must have originated in the Late Jurassic, or earlier, in accordance with its sister tribe Tachinusini. In contrast, the origin of Mycetoporinae can be reliably traced back only to the Early Cretaceous.
5. My backbone phylogeny of Tachyporinae may play an important role for evolutionary ecology or inventory surveys in the future. For example, semi-aquatic taxa are found only in the Deropini + Tachinusini clade, while myrmecophily and termitophily occur only in Vatesini and Tachyporini. Mycetoporinae species are frequently associated with fresh mushrooms. The clarification of morphological adaptations of these taxa would be an interesting and valuable subject for further study.

## Data Availability

All data generated or analyzed during this study are included in this published article (and its Supplementary Information files). Higher-resolution figures have been deposited in the figshare repository (https://doi.org/10.6084/m9.figshare.14179529; accessed on 9 April 2021). The figures and supplementary materials are also available on the Zenodo repository (https://doi.org/10.5281/zenodo.4660863; accessed on 9 April 2021).

## Supplementary Materials

The following are available online at https://www.mdpi.com/2079-7737/10/4/323/s1, File S1: List of examined material and literature in Word format (.docs), File S2: List of examined material and literature in PDF (Portable Document Format) format (.pdf), File S3: Character matrix for phylogenetic analyses in nexus format (.nex), with resulting parsimonious trees, File S4: Character matrix for phylogenetic analyses in TNT format (.tnt), Figures S1–S3: Supplementary figures in PDF format with figure captions (.pdf).

## Appendix A. Additional Note

During at a proofreading stage of this manuscript, a taxonomic paper describing three new species belonging to the subgenus *Latotachinus* Ullrich of the genus *Tachinus* Gravenhorst (Tachinusini stat. rev., sensu nov.) from China was published [199]. Following this taxonomic addition, I made minor amendments on the species counts in the present paper.

## Appendix B. List of Characters

Morphological characters and character states used in my phylogenetic analyses: Each taxonomic category mentioned here is treated in the newly revised sense following my taxonomic changes.

Head:

1. Head, shape (dorsal and ventral views): (0) as long as wide to transverse, oval to hexagonal, not rectangular (Figures 16A and 50B); (1) elongate, slightly to moderately longer than wide (Figure 64B); (2) markedly elongate, much longer than wide (Figures 64D and 67A); (3) as long as wide, rectangular.

Head shapes of Tachyporinae are usually slightly to moderately transverse, whereas those of Mycetoporinae are usually elongate (extreme cases seen in *Bobitobus*; Figure 64D), with only a few exceptions.

2. Head, ocelli (dorsal view): (0) absent; (1) present.

3. Head, uniform punctation on vertex (modified from Herman [25]: character 0) (dorsal view): (0) absent (Figures 7C, 23B, 51D and 64D); (1) fine; (2) medium (Figure 44A); (3) large (Figure 58A).

The dorsal surface of the head of Tachyporinae generally lacks punctation, however, that of Deropini is covered with moderate punctation, and of some Tachinusini (members of the former Megarthropsini) with much larger and deeper punctation.

4. Head, microsetae [dorsal view]: (0) absent (Figures 7C, 50A, 51D, and 64D); (1) present, uniformly and sparsely covered with thin, minute, and inconspicuous setae; (2) present, uniformly (at least apical half of head dorsum) and densely covered with thin, rather conspicuous setae (Figures 15B and 44A); (3) present, only partially covered; (4) present, uniformly covered with extremely short, thick, but vestigial setae (nearly absent); (5) present, uniformly covered with extremely short, thick, distinctly modified setae (Figure 58C; [25]: Figure 185).

The surface of head is glossy and glabrous in Mycetoporinae but in Tachyporinae have a series of variations as listed above. The dorsal surface of the head is glabrous in Tachyporina, Vatesini, and most taxa of Tachinusini. It is, however, densely pubescent in Deropini and Euconosomatina.

5. Head, surface [dorsal view]: (0) even (Figures 7C and 50A); (1) weakly rough, with indentations; (2) moderately rough or tuberculate (Figure 58B); (3) dorsum with numerous, long, distinct, longitudinal carinules; (4) dorsum with deep, oblique grooves.

In Tachyporinae, the surface of head is generally even, but that of some Tachinusini (members of the former Megarthropsini) is rough (Figure 58B; [25]: Figure 184).

6. Head, ocular seta near posterior of each eye (sensu Campbell [42]) (dorsal view): (0) absent (Figures 16A, 51D and 64D); (1) present, thin, short (Figures 50 and 67B); (2) present, much thicker and longer, conspicuous (Figure 64A–C).

In some outgroups and Mycetoporinae, there is a pair of ocular setae near the posterior margins of the eyes. However, *Bobitobus* (*B. lunulatus*) lacks such setae (Figure 64D). I found a pair of short ocular setae in *Bryophacis smetanai* (Figure 67B: 6-1), although it has been considered as absent [33]. Within Tachyporinae, some members of Tachinusini (members of the former Megarthropsini) also have such setae, but they are much shorter and less conspicuous than those of typical mycetoporines.

7. Clypeal anterior margin (modified from Herman [25]: character 5) (dorsal view): (0) various, not reflexed upward (Figure 50A); (1) only weakly reflexed upward; (2) strongly reflexed upward (Figure 58B,C).

In the limited taxa of Tachinusini, the anterior margin of the clypeus is reflexed above (see Herman [25]).

8. Frontal shelf (shelf-like projection at each side of frons) [dorsal and lateral views]: (0) present, covering antennal insertion (Figures 44A and 50B); (1) absent (Figure 64B,D).

There are more or less developed frontal shelves in Tachyporinae, but they are absent in Mycetoporinae.

9. Antennal insertions (dorsal view): (0) partially exposed, more or less visible from above, but at least concealed partially under frontal shelf or part of frons (Figures 41J, 44A and 50B); (1) nearly fully exposed, but slightly concealed under very weakly developed projection of frons; (2) fully exposed, completely visible from above, not concealed under frontal shelf or part of frons (Figure 64B,D); (3) not visible from above, completely concealed under strongly developed frontal shelf.

The antennal insertions are more or less (= moderately) visible in Tachyporinae, whereas they are fully exposed in Mycetoporinae (spherical insertion fully or almost fully visible).

10. Antennal insertion, location (modified from Grebennikov & Newton [2]: character 94) (dorsal view): (0) antennae inserted at, or anterior to, anterior margin of eyes (Figures 44A, 50B and 64D); (1) antennae inserted posterior to anterior margin of eyes ([189]: Figure 1.13.1); (2) antennae vertically inserted posterior to anterior margin of eyes (Figure 26G).

In Tachyporinae, antennae are generally inserted at, or anterior to, the anterior margin of the eyes. However, the head capsule of two tachyporine genera, namely *Vatesus* (Vatesini) and *Lamprinus* (Tachyporina), are deformed in associations with their myrmecophilous lifestyles. This results in peculiar antennal insertions which are located posterior to the anterior margin of the modified, vertical eyes.

11. Midcranial suture (sensu Naomi [200]: Figure 1B) (dorsal view): (0) absent (Figures 15A, 23A, 44A, 58C and 64A); (1) present (Figures 7A, 23B, and 50).

There is a well-developed midcranial suture on the frons in most taxa of Tachyporinae (Figure 7A), although some tachyporine genera, mostly specialized taxa such as myrmecophiles, lack such sutures. In one myrmecophilous species, *Lamprinodes saginatus* (Tachyporina), it is very short and indistinct, but it is considered here as present.

12. Postocular area, neck or narrowing (dorsal and ventral views): (0) absent (Figures 9A, 16A, 23A, 51A, 64B, and 67C); (1) present: head capsule very slightly narrowed at middle of postocular area (Figure 26A); (2) present, narrowed only weakly just behind eyes (Figures 8A,B, 64A and 67A); (3) present, narrowed moderately just behind eyes at sides only (Figures 50, 51B,D, 53A and 58A); (4) present, narrowed distinctly at middle of postocular area, all around (Figures 44A and 45A); (5) present, neck strongly constricted just behind eyes, distinct all around; (6) present, neck strongly constricted at middle of postocular area, distinct all around.

The conditions of the postocular areas vary greatly among the selected taxa used here. In some outgroups, there is strong neck constriction behind the eyes, e.g., Neophoninae, Pseudopsinae, and Trichophyinae. In Mycetoporinae, such constriction in the neck region is absent or alternatively weakly narrowed, starting immediately behind the eyes. Several different conditions of this character state were found in Tachyporinae. For example, the head lacks narrowing or constriction in most taxa of Tachyporina, some Vatesini, and *Nitidotachinus* (Tachinusini). Within Vatesini, only *Termitoplus* and *Coproporus* have a weak narrowing in the middle of the postocular area. In general, Tachinusini has a moderately narrowed neck region, starting from just behind the eyes. A similar condition was found in Deropini, but they are narrowed from the middle of the postocular area to the base of the head capsule.

13. Head capsule, with a pair of longitudinal ridges basolaterally along underside of eyes (ventral view): (0) absent (Figure 26A); (1) present, incomplete (Figures 53A and 58D); (2) present, complete (Figure 67A,C).

The complete and distinct infraorbital ridge can be found in some outgroups (i.e., Olisthaerinae, Staphylininae, and Aleocharinae) and Mycetoporinae. There is no such ridge found in Tachyporinae, although *Leucotachinus* and *Nepaliodes* in Tachinusini possess a pair of incomplete, somewhat weak wavy ridges.

14. Hypostomal sutures, including traces (sensu Naomi [200]: Figure 1A) (ventral view): (0) curved or angulate (Figures 10E, 16B, 45A, and 53A); (1) straight (Figure 67C); (2) fused for part of their length, without any trace at least partially; (3) absent.

In some outgroups and Mycetoporinae, the hypostomal sutures are generally not curved, each forming a nearly straight line. In contrast, they are curved, or forming a distinct angle, in Tachyporinae. This condition is possibly a universal condition in the subfamily, and the hypostomal sutures in Tachyporini form a pair of strong angles with the submentum.

15. Gular sutures (sensu Naomi [200]: Figure 1A) (ventral view) (Grebennikov & Newton [2]: character 98): (0) well separated throughout (Figures 10E and 67C); (1) fused for part of their length.

16. Gular sutures (ventral view): (0) not markedly curved or divergent posteriorly (Figures 10E, 45A and 67C); (1) markedly strongly curved or divergent posteriorly (Figure 16B); (2) fused for part of their length.

In *Euconosoma*, *Sepedophilus* (both Euconosomatina), and *Vatesus* (Vatesini), the gular sutures are markedly divergent basally.

17. Postgena (ventral view): (0) without minute setae (Figure 67A); (1) with minute setae (Figures 24B and 45A); (2) with strong setae (Figure 58D).

Some members of Mycetoporinae, Vatesini, Tachinusini, and Deropini have scattered minute setae on the postgena, but they are absent in most other tachyporines.

18. Dorsal tentorial arms (sensu Naomi [200]: Figure 1B; Naomi [201]: Figure 2A) (dorsal view): (0) present, usually forming a pair of tentorial pits (Figures 9A and 16A); (1) absent or strongly reduced (Figure 67B).

As a general trend, the dorsal tentorial arms are strongly reduced in most members of Mycetoporinae, but I found dorsally pointed arms in a few mycetoporine genera shown in the phylogeny, namely *Parabolitobius* and *Bolitobius*. On the other hand, they are well-developed in Tachyporinae, and even reach the inner surface of the dorsum, resulting in a pair of tentorial pits in most cases.

Antennae.

19. Antenna, structure (dorsal and ventral views): (0) normal, moniliform to filiform (Figures 9D, 64B and 68F,G); (1) weakly modified in association with social insects (i.e., flagellomeres weakly flattened and contiguous, concealing each stem pedicels; Figure 9C); (2) strongly modified in association with social insects (i.e., flagellomeres strongly flattened and adjacent ones contiguous, concealing pedicels) (Figure 26G); (3) weakly clavate or clubbed apically (Figure 23B); (4) extremely slender, verticillate.

In Tachyporinae, the general shape of the antennae is filiform to fili-moniliform Although this condition may be considered as “incrassate” (Lawrence et al. [202]: character 38-2; Lawrence & Ślipiński [203]: 35), I maintain the usage both filiform and moniliform here. *Mimocyptus* and *Termitoplus* (both Vatesini) have weakly clubbed antennae, unusual within the subfamily. *Lamprinodes* (Tachyporina) has slightly flattened, modified antennae in association with myrmecophilous lifestyles [15]. The extreme cases, markedly telescoped, modified, myrmecophilous antennae [15], are found in *Lamprinus* (Tachyporina) and *Vatesus* (Vatesini). The presence of very slender, verticillate antennae characterizes the subfamily Trichophyinae and the genus *Habrocerus* (Habrocerinae) Habrocerinae.

20. Antenna, length compared to head width width (dorsal and ventral views): (0) not distinctly short, clearly longer than head width (Figures 15A and 64B); (1) distinctly short, as long as, or only slightly longer than, head width (Figure 23B).

Mycetoporinae can be characterized by the presence of long and slender antennae, but those of the tachyporine taxa vary greatly from very short (some Vatesini) to markedly long (Deropini and some Tachinusini). A notably longer condition of the antennae can be seen in semi-aquatic taxa, whereas very short antennae are generally found in myrmecophilous or termitophilous taxa, seeming to have a protective function. In Vatesini, the presence of shorter antennae, as long as or shorter than pronotal width, is a general common state in the tribe, and is shared among the crown group.

21. Antennal micropubescence distribution (dorsal and ventral views): (0) without clear borderline, density increasing apically (Figures 9C,D, 17A, 45C and 68F,G); (1) with clear borderline, lacking dense and fine recumbent setae on basal three antennomeres (a1–a3) (Figure 26D); (2) with clear borderline, lacking dense and fine recumbent setae on a1–a4; (3) with clear borderline, lacking dense and fine recumbent setae on a1–a2 (Figure 59C); (4) with clear borderline, lacking dense and fine recumbent setae on a1–a5; (5) with clear borderline, lacking dense and fine recumbent setae on a1–a8; (6) without clear borderline, almost same density from base to apex.

The distributional patterns of the antennal micropubescence have been used in generic classification and identification of Tachyporinae (e.g., [91]). Although there is no setal borderline of the antennae observed in most Mycetoporinae, a rather clear setal borderline was found in *Neobolitobius*, and was considered as ‘2′. In Tachyporinae, this character is highly variable among the tribes, genera, and species. Within Tachyporini, the antennae lack such borderlines, with gradually increasing density of microsetae towards the apex. In contrast, Vatesini has three different types of antennal micropubescence patterns (21: 0-2), antennae without dense and fine recumbent setae on the basal three antennomeres is the most common state in this tribe (21-1). Within Tachinusini, the antennae lack such recumbent setae on the basal four antennomeres in most cases (21-2), but *Nitidotachinus* and members of the former Megarthropsini lack these setae on only the basal two antennomeres (21-3). Deropini has a densely pubescent antenna, but there is no clear borderline found in the basal third (21-0).

22. Antennomere 4. (0) without markedly long setae (Figure 68F); (1) with markedly long setae, longer than 2.2 times of width of antennomere IV.

Two subfamilies, i.e., Trichophyinae and Habrocerinae, share the characteristic verticillate antennae, bearing very long setae sparsely on each antennomere ([189]: Figure 1.21.2).

Mouthparts.

23. Labrum, divided into two halves by medial longitudinal suture (modified from Ashe [28]: character 104): (0) neither divided nor thinner longitudinally along midline of labrum (Figure 67D); (1) not divided, but thinner longitudinally along midline of labrum; (2) longitudinally divided along midline of labrum.

In Habrocerinae, the labrum is divided into two halves by a medial longitudinal suture ([192]: Figure 1C).

24. Labrum, anterior margin, lateral areas, setose or spinose processes composed of filiform setulae (dorsal view) (modified from Ashe [28]: character 1): (0) absent or very weakly developed, only sparsely distributed (Figure 67D); (1) well-developed, conspicuous (Figures 9B, 16D, 26C, 45A, and 53H).

In general, the Tachyporinae have a pair of well-developed setose or spinose processes on the anterior margin of the labrum, whereas most Mycetoporinae lack such setose processes. However, it should be tested in future if these setose processes are homologous because of the variations on the locations, size, and structures.

25. Labrum, anterior margin, medial area (dorsal and ventral views) (Ashe [28]: character 93): (0) without row of flattened, spatulate or modified setae (Figure 67D); (1) with a row of flattened, spatulate or modified setae (Figures 9B, 16D, 26C, 45A, and 53H).

Most of the outgroups and Mycetoporinae lack a row of flattened, spatulate, or modified setae in the anteromedial margin of the labrum, but Tachyporinae typically have the peculiar, modified setae.

26. Right mandible, number of subapical teeth (modified from Ashe [28]: character 15) (dorsal and ventral views): (0) absent (Figures 9B, 16A, 45A, and 67A); (1) present, one small; (2) present, one large; (3) present, two large.

In Tachyporinae and Mycetoporinae, there is no subapical tooth along the inner edge of the right mandible. However, four subfamilies (Phloeocharinae, Aleocharinae, Trichophyinae and Olisthaerinae) in the Tachyporine Group of subfamilies have such subapical teeth.

27. Left mandible, number of subapical teeth (modified from Ashe [28]: character 16) (dorsal and ventral views): (0) absent (Figures 9B, 16A, 67A, and 68E); (1) present, one large; (2) present, one large, one small; (3) present, two large.

In Tachyporinae and Mycetoporinae, there is no subapical tooth along the inner edge of the left mandible. However, four subfamilies (Phloeocharinae, Aleocharinae, Trichophyinae and Olisthaerinae) in the Tachyporine Group of subfamilies have such subapical teeth.

28. Mandible, spinose molar lobe (modified from Ashe [28]: character 17) (ventral view): (0) present (Figures 9B, 16C and 67A); (1) absent.

Except for *Oxypoda* sp. in the ‘higher’ group of Aleocharinae and *Quedius paradisi* Hatch in Staphylininae, all taxa studied here have a spinose molar lobe in each mandible. They are well-developed in Tachyporinae, but much smaller in Mycetoporinae (although it is difficult to separate the coding).

29. Maxillary palpus, structure: (0) 4-segmented; (1) 5-segmented.

The maxillary palpus is comprised of five palpomeres in Trichophyinae: the fourth maxillary palpomere is large and spindle-shaped, but the terminal, fifth palpomere is minute and hyaline [16]. Both Mycetoporinae and Tachyporinae have 4-segmented maxillary palpi.

30. Maxillary palpomere 3, shape (dorsal and ventral views): (0) widest at or near apex (Figures 10C,D, 27C, 45D, 53C, 59A and 68A,B,D,E); (1) widest around middle (Figures 10A, 16E and 68C).

Most Mycetoporinae have an apically broadened maxillary palpomere 3. In contrast, two types of palpomere 3 were found in Tachyporinae. Within Tachyporini, all taxa, except *Lamprinus* and *Lamprinodes* have state 1. The remaining three tribes, Vatesini, Deropini, and Tachinusini, had state 0.

31. Maxillary palpomere 3, length compared to palpomere 2 [dorsal and ventral views]: (0) not distinctly shorter than maxillary palpomere 2 (>0.7×) (Figures 10A, 27B,C and 68A,D); (1) distinctly shorter than maxillary palpomere 2 (≤0.7×) (Figures 27A, 45D, 53C and 59A).

In Mycetoporinae, Tachyporini, and nearly all of the outgroups, maxillary palpomere 3 is not distinctly shorter than the maxillary palpomere 2. In contrast, some members of Vatesini (*Cileoporus*, *Cilea*, and *Termitoplus*), Deropini, and Tachinusini have palpomere 2 distinctly longer than 3.

32. Maxilla, maxillary palpomere 3, micropubescence setation (dorsal and ventral views): (0) moderately pubescent, with less than 20 setae (>10) (Figure 68D); (1) strikingly densely setose, covered with dozens of setae (>20) (Figures 10A, 16E, 27C and 68A); (2) only sparsely setose, with single to ten scattered stout setae, usually restricted to apex of palpomere (Figures 27A, 53C, 59A, and 68E); (3) completely glabrous (Figure 45D).

This character is variable among the members of Mycetoporinae (32: 0-2). The maxillary palpomere 3 in Tachyporini is densely pubescent, whereas the other tachyporine tribes have some variations. In most Vatesini, the palpomere is nearly glabrous, bearing a few scattered setae, but it is moderately pubescent in *Mimocyptus* and densely pubescent in *Vatesus* (in *Termitoplus*, they are very short, but sparsely scattered). In Tachinusini, all genera have a sparsely setose palpomere except *Leucotachinus* (moderately pubescent). Uniquely, palpomere 3 of Deropini is completely glabrous.

33. Maxilla, maxillary palpomere 4, shape (dorsal and ventral views): (0) moderately to strongly pointed apically, widest at or near base (Figures 10C, 16E, 27A, and 68B,D); (1) only weakly to strongly narrowing apically in apical half, greatest width in basal 1/6 to middle (Figures 45D, 53C, 59A and 68A,E); (2) widest near middle, abruptly narrowed near apex; (3) markedly slender, aciculate (Figure 68C).

Among the taxa of Mycetoporinae, three different types of the maxillary palpomere 4 were found. *Parabolitobius*, *Bolitobius*, and *Bobitobus* (*B. lunulatus*) were coded as ‘1′, while *Mycetoporus* and *Ischnosoma* have unusually slender, aciculate palpomere 4, scored as ‘3′. The rest of Mycetoporinae were coded as ‘0′ as they have strongly pointed ones. In Tachyporinae, Tachyporini and Vatesini were coded as ‘0′, whereas Deropini and Tachinusini, were scored as ‘1′.

34. Maxilla, maxillary palpomere 4, length (dorsal and ventral views): (0) short, much shorter than palpomere 3 (0.2–0.75×) (Figures 10C, 16E, 27C and 68C); (1) moderate, subequal or slightly shorter than palpomere 3 (>0.75–1.1× ≤) (Figure 68B); (2) long, clearly longer than palpomere 3 (>1.1×) (Figures 27A,D, 45D, 53C, 59A and 68E); (3) distinctly short, much shorter than palpomere 3 (<0.2×).

In Mycetoporinae, this character state was generally coded as either ‘1′ or ‘2′, with the exceptions of *Mycetoporus* and *Ischnosoma* (both coded as ‘0′). It was coded as ‘0′ in Tachyporini, whereas it was generally scored as ‘2′ for Vatesini, Deropini, and Tachinusini, except *Vatesus* (34-0) and *Mimocyptus* (34-1) (both Vatesini).

35. Maxilla, maxillary palpomere 4, maximum width in apical half (dorsal and ventral views): (0) moderately to distinctly narrower than maximum width of palpomere 3 (Figures 10C, 16E, 27C and 68C); (1) the same as, or only slightly narrower, than maximum width of palpomere 3 (Figures 45D, 53C and 68A).

This character state was generally coded as ‘0′ in Mycetoporinae, Tachyporini, and Vatesini and as ‘1′ in Deropini and Tachinusini.

36. Maxilla, maxillary palpomere 4, pores (dorsal and ventral views): (0) absent (Figures 10C and 16E); (1) present, with only a few small, inconspicuous pores; (2) present, with several to dozens of inconspicuous, or conspicuous, pores (Figures 45D, 53C, and 68E).

Nearly all members of Mycetoporinae have several to dozens of the inconspicuous, or conspicuous, pores on the maxillary palpomere 4. The presence of such pores are widely found in the Deropini + Tachinusini clade. However, the rest of Tachyporinae generally lack the pores on the palpomere 4, with some exceptions in Vatesini.

37. Maxilla, lacinia, apical unarticulated large, long spine (Grebennikov & Newton [2]: character 124) (dorsal and ventral views): (0) absent (Figures 16E, 27B, 45D and 53F); (1) present (Figures 10C and 68A).

In Mycetoporinae and Tachyporina, there is a conspicuous, well-developed long spine located at the apex of the maxillary lacinia. In *Sepedophilus* (Euconosomatina), it is so small and inconspicuous that it is considered as absent here. I could not find such a long spine in the tribes Vatesini (except *Mimocyptus*), Deropini, and Tachinusini.

38. Maxilla, lacinia, length of at least some of teeth or spines (dorsal and ventral views): (0) longer than maximum width in larger part of apical third of lacinia (Figures 16E, 36C and 68B); (1) as long as or shorter than maximum width in larger part of apical third of lacinia (Figures 27B, 45D, and 53C).

In Mycetoporinae (except *Mycetoporus* and *Ischnosoma*) and Tachyporini, some of the teeth or spines along the inner margin of the maxillary lacinia are longer than the maximum width of the apical third of the lacinia. However, those of Vatesini, Deropini, and Tachinusini are shorter because of the shorter teeth (spines) and much broader apex of the lacinia. Among these three tribes, the only exception was *Mimocyptus* (Vatesini), which was scored as ‘0′.

39. Labium, labial palpomere 2, shape (dorsal and ventral views): (0) elongate to only weakly transverse (Figures 16F, 26B 45B, 53D, and 59B); (1) strongly to markedly transverse (Figures 26E,F and 67E).

All members of Mycetoporinae, except *Mycetoporus*, have a strongly transverse labial palpomere 2. Within Tachyporinae, the earlier-branching Vatesini and all Tachyporini have elongate to only weakly transverse ones, whereas the remaining tachyporine taxa were scored as ‘1′.

40. Labium, labial palpomere 3, shape (dorsal and ventral view): (0) widest at base (Figures 9E, 16F and 26E); (1) widest around middle or near base, not at base (Figures 26B,F, 45B, 53D,E, 59B and 67F); (2) narrowly elongate, straight, slender, subparallel-sided; (3) narrowly elongate, curved, slender, subparallel-sided; (4) nearly subparallel-sided, thick, weakly broadened apically, with truncate apex (Figure 67E); (5) distinctly expanded, crescent-shaped.

Most members of Mycetoporinae were coded as ‘1′, with two exceptions. Most Tachyporinae were scored as either ‘0′ or ‘1′. All members of Tachyporini, except *Euconosoma* (Euconosomatina), were coded as ‘0′. *Euconosoma* has a distinctly widened, laterally compressed, crescent-shaped labial palpomere 3 (see Campbell [55]: Figure 2). In contrast, the tribes Vatesini, Deropini, and Tachinusini were coded as ‘1′ except *Mimocyptus* (Vatesini), which was scored as ‘0′.

41. Labium, labial palpomere 3, maximum width (dorsal and ventral views): (0) distinctly to moderately narrower than maximum width of palpomere 2 (<0.75×) (Figures 9E, 16F, 26E and 67E); (1) almost same width or only slightly narrower than maximum width of palpomere 2 (0.75× ≤, <1.10×) (Figures 26B,F, 45B, and 59B); (2) moderately wider than maximum width of palpomere 2 (1.10–1.50×) (Figure 67F); (3) distinctly wider than maximum width of palpomere 2 (>1.5×).

Three states were found in Mycetoporinae, and they were scored from ‘0′ to ‘2′. In Tachyporinae, Tachyporini was generally coded as ‘0′, whereas Deropini and Tachinusini were mostly coded as ‘1′. Vatesini were either ‘0′ or ‘1′. *Euconosoma* (Euconosomatina) has a distinctly widened labial palpomere 3, and was scored as ‘3′ ([44]: Figure 1; [55]: Figure 2).

42. Labium, labial palpomere 3, with a few to several thick, short peg-like spines on surface (dorsal and ventral views): (0) absent (Figures 16F and 26B); (1) present (Figures 26E and 67E).

Some genera of Mycetoporinae and Vatesini have a few to several thick, short peg-like spines on the outer margin of the labial palpomere 3 (*lp3*) (e.g., Ashe [28]: Figure 84). The location of these spines has some variations from near lateral base to the apex of lp3. However, it is difficult to assess the homology of this character state, I tentatively scored them the same state (although it is highly unlikely homologous).

43. Labium, labial palpomere 3, pores (dorsal and ventral views): (0) absent; (1) present, inconspicuous (Figures 9E, 16F, 26B, and 67F); (2) present, conspicuous (Figures 45B, 53D and 59B).

In Mycetoporinae, some Vatesini, and *Sepedophilus* (one species only, Euconosomatina), there are inconspicuous pores scattered on the labial palpomere 3. Contrary to these taxa, I observed much larger and conspicuous pores on this palpomere in Deropini and most Tachinusini.

44. Labium, ligula, shape (ventral view): (0) large, simple, as long as wide to transverse (Figures 9E and 45B); (1) small, simple, restricted to median area (Figure 67F); (2) pair of medial lobes moderately present (Figure 53E); (3) pair of medial lobes weakly present, with spines on the lobes (Figures 16F and 53B); (4) markedly narrowly elongate, pointed apically; (5) large, transeverse, with spines in middle.

In Mycetoporinae, the general shape of the ligula (glossae and paraglossae) is small and simple, although they occasionally have moderately developed medial premental lobes (*Bryoporus*). In Tachyporinae, the ligula is much larger and wider than that of Mycetoporinae. Interestingly, some *Sepedophilus* (Euconosomatina) have a pair of weakly developed medial premental lobes, occasionally together with a few conspicuous peg-like spines at each apex ([28]: Figure 86). It should be also mentioned that *Austrotachinus* ([110]: Figure 2) and *Olophrinus* (Figure 53E) (both Tachinusini) each have a pair of the moderately developed medial premental lobes, and were scored as ‘2′.

45. Labium, ligula, extent (dorsal and ventral views): (0) small, slightly extended laterally, but not beyond each labial palpus (Figure 67E,F); (1) medium to large, clearly extended laterally beyond each labial palpus (Figures 9E, 16F, 26B, 45B, 53E, and 59B); (2) elongate and very large, but limited to median area only, not extended laterally beyond middle of each labial palpus.

Mycetoporinae have a small ligula, whereas those of Tachyporinae are much wider and larger, extending laterally beyond the outer margins of each labial palpus (e.g., Ashe [28]: Figures 84–86).

46. Labium, insertions of labial palpi (modified from Grebennikov & Newton [2]: character 131) (dorsal and ventral views): (0) narrowly to moderately separated, closer than length of longest labial palpomere, sometimes even narrower than maximum width of labial palpomere 1 (Figures 45B, 53D, and 67E); (1) more widely separated, slightly narrower than, or subequal to, narrower, or subequal, to longest labial palpomere (Figures 9E, 16F and 26E); (2) widely separated, clearly wider than longest labial palpomere (Figures 26B,F, 53G and 59B).

In Mycetoporinae, the insertions of the labial palpi are close, much closer than the length of longest labial palpomere. Some variation was found in Tachyporinae. Within Tachyporini, they were rather widely separated in most cases (except in some Euconosomatina). In Vatesini, the labial palpi are generally widely separated, except in *Mimocyptus*. They are very narrowly separated in Deropini, while Tachinusini has much wider separation, except in the semi-aquatic genus *Nitidotachinus*.

47. Mentum, anterior margin (ventral view): (0) more or less truncate (Figure 53B); (1) broadly shallowly emarginate; (2) broadly rounded (Figure 26F); (3) broadly strongly emarginate; (4) pointed in middle.

In Mycetoporinae, the anterior margins of the mentum are always truncate. However, some Tachyporinae have a broadly rounded anterior margin of the mentum (e.g., Euconosomatina and part of Vatesini).

48. Mentum, shape (ventral view): (0) weakly to moderately transverse, <2.85× as wide as long (Figures 26F and 53B); (1) strongly transverse, ≥ 2.85 × as wide as long; (2) distinctly narrowed towards apex.

In Mycetoporinae, the ratio of the mentum is more than 2.85 times as wide as long in most cases, with the exceptions of *Bobitobus* and *Mycetoporus*. In Tachyporini, they are strongly transverse, but those of Vatesini, Deropini, and most of Tachinusini (except *Olophrinus* and *Tachinomorphus*) have a relatively transverse mentum, here considered as ‘0′.

Thorax and legs.

49. Pronotum, shape (dorsal view): (0) not constricted or distinctly narrowed (Figure 64B); (1) abruptly narrowed posteriorly, moderately constricted in posterior half (Figure 44A); (2) weakly constricted laterally at base; (3) weakly constricted anteriorly and posteriorly; (4) forming unique corners along plate-like lateral projections (Figure 58C).

In Deropini, the pronotum is unusually strongly constricted in the posterior half. Furthermore, two genera of Tachinusini (*Megarthropsis* and *Nepaliodes*) have only weakly constricted pronotum along the plate-like lateral margins, differing completely from the modifications of Deropini.

50. Pronotum, maximum width, location (determined from pinned specimens) [dorsal view]: (0) at base (Figures 15C, 24D, and 51C); (1) between near base and basal 1/5 (Figures 7D and 27G); (2) between basal 1/5 and basal 1/4 (Figures 8A, 51D); (3) between basal 1/4 to middle (Figures 7B, 24A, 51A,B, 58A, 59D,E, 64B,D, 65D, and 69A); (4) in anterior half, but not near anterior margin (Figure 44A); (5) in anterior half, near anterior margin.

In Mycetoporinae, the maximum width of the pronotum is located between the basal 1/4 to middle because of the strongly rounded posterior pronotal margins. A series of variations are found in Tachyporinae: Tachyporina was coded as ‘1′, with the exception of *Palporus* and *Symmixus*, which were considered as ‘3′ and ‘2′, respectively; Euconosomatina was scored as ‘0′; Vatesini was coded with ‘0′, ‘1′, or ‘3′; Deropini was considered as ‘4′; Tachinusini was mostly scored as ‘3′, but some were considered to be either ‘2′ (*Olophrinus*) or ‘0′ (*Tachinomorphus*).

51. Pronotum, apical half, uniform punctation (modified from Herman [25]: character 0) (dorsal view): (0) absent (Figures 7D, 24D, 51D, and 64B); (1) fine; (2) dense, but shallow (Figure 44A); (3) coarse and small but relatively deep; (4) coarse and large, deep, pit-like (Figure 58A,B).

All taxa of Mycetoporinae lack uniform punctation, and the surface of the forebody is strongly glossy. In Tachyporinae, the tribes Tachyporini and Vatesini also do not have conspicuous punctures on the forebody. However, some members of Tachinusini (= Megarthropsini) have well-developed, pit-like punctures. The pronotum of Deropini is covered with dense but shallow punctation.

52. Pronotum, surface [dorsal view]: (0) more or less even (Figures 51D and 64B); (1) rough, with indentations (Figures 51B and 58B).

Excepting some outgroups and part of Tachinusini (e.g., Herman [25]: Figure 183), the surface of the pronotum is even dorsally without grooves. There are some variations of indentations found in limited in- and out-group taxa. Since it is difficult to assess the homology, they were treated as a single state.

53. Pronotum, microsetae, except for characteristically arranged macrosetae [dorsal view]: (0) glabrous (Figures 7D, 24D, 51D, 58A, and 64B); (1) pubescent, sparsely covered with very minute setae; (2) pubescent, densely covered with thin, moderately long setae (Figures 15B and 44A); (3) pubescent, covered with extremely short, thick, distinctly modified, vestigial setae (Figure 58C).

In Mycetoporinae, the surface of the pronotum is uniformly glabrous, with characteristically arranged, marginal macrosetae. Within Tachyporini, it is generally glabrous in Tachyporina, whereas the setae are moderately long and conspicuous in Euconosomatina. In Vatesini, it is completely glabrous, except in *Cilea*. The pronotum of Deropini is densely pubescent, and genenerally glabrous in Tachinusini sens. str. Notably, however, *Nepaliodes* in Tachinusini has distinctly short, modified setae on the pronotum ([25]: Figure 186).

54. Pronotum, dorsolateral marginal areas, modification (dorsal view): (0) normal, not modified (Figures 51D and 64B); (1) weakly explanate (Figure 58A); (2) strongly explanate (Figure 58B); (3) foveate; (4) serrate.

In limited Tachinusini (= Megarthropsini), the dorsolateral marginal areas of the pronotum are weakly to strongly modified, and the most extreme case is found in *Nepaliodes* ([25]: Figure 183).

55. Pronotal apex (= anterior margin) (dorsal view): (0) truncate (Figure 44A); (1) weakly to moderately concave (Figures 7A, 18A, 51B, 27E, 54G, 59D,E, and 64B); (2) strongly concave; (3) distinctly modified, markedly strongly concave (Figure 58B).

As a general trend, the anterior margin of the pronotum is generally weakly to strongly concave in Mycetoporinae and Tachyporinae. However, it is truncate in Deropini, *Vatesus gigas* (Vatesini), and some mycetoporines.

56. Pronotum, anterior/front angles (dorsal and ventral views): (0) broadly rounded (Figures 51B, 58A, and 59D,E); (1) angulate (Figures 27G, 44A, 64B and 69A); (2) sharply pointed (Figure 7A, 15A, 18A); (3) narrowly rounded, strongly produced [25] (Figure 58B,C).

The anterior angles of the pronotum vary greatly as follows: from rounded (Tachinusini), angulate (Mycetoporinae, Vatesini (except *Vatesus gigas*), and Deropini), sharply pointed (Tachyporini), to having a peculiar form (*Nepaliodes* in Tachinusini).

57. Pronotum, macrosetae [dorsal view]: (0) absent (Figures 15C and 51D); (1) present, characteristically arranged (Figures 7A, 64A,C, and 69A).

Characteristically arranged setae on the pronotal margins were found in Mycetoporinae and Tachyporina. In *Leucotachinus* (Tachinusini), margins of the pronotum are furnished with series of discrete punctures, each bearing a short seta [91], but they are minute and considered as absent.

58. Pronotum, transverse cluster of blackish to blackish-brown small pores near posterior margin [dorsal and ventral views]: (0) absent (Figures 11A, 18A,B, 27E,G, 45E, 59D,E, and 69A,B); (1) present (Figure 54G).

This character state can only be seen in slide-mounted specimens. In some Vatesini and most members of Tachinusini, there are some transversely scattered clusters of blackish brown pores located near the posterior margins of the pronotum. Other taxa have much more weakly pigmented or ambiguous pores, or even lack such pigmented pores. The structural details and the assessment of its homology are needed to be assessed in future.

59. Pronotal hypomeron, transverse ridge (ventral view): (0) absent (Figures 11A, 18B, 27F, 54G and 59D); (1) present, usually located in anterior 1/3 to 1/4 of each pronotal hypomeron (Figure 69A,B).

In Mycetoporinae and some outgroups, there is a prominent ridge located in the anterior area of each pronotal hypomeron, observable in ventral view.

60. Pronotal hypomeron, width in basal third (ventral view): (0) narrow (Figures 45E, 69A, B); (1) moderate (Figures 11A and 54G); (2) wide (Figures 18B, 27G, and 59D,E).

The maximum width of the pronotal hypomeron greatly varies among the studied taxa. For example, it is narrow in Mycetoporinae and Deropini; moderate in most Tachyporina (except *Symmixus*) and most members of Tachinusini; and wide in *Symmixus*, Euconosomatina, Vatesini, and some Tachinusini.

61. Pronotal hypomeron, postcoxal process (ventral view): (0) absent, at most only very feebly projecting inward (Figure 69A); (1) weakly developed (wider than long), with rounded apex (Figure 27E); (2) weakly developed (wider than long), with truncate apex (Figure 18A); (3) weakly developed (wider than long), with pointed apex; (4) moderately developed (as long as wide to slightly longer than wide) (Figures 18B and 45E); (5) fully developed (clearly longer than wide), usually with rounded or narrowly rounded apex (Figures 11A, 54G and 59D,E).

A series of variations were found in the lengths and structures of the pronotal postcoxal processes on the pronotal hypomeron. They are completely absent in Mycetoporinae, whereas they are weakly developed in Vatesini, moderately to strongly projecting in Tachyporina and the clade Deropini + Tachinusini. Interestingly, there are some variations found in Euconosomatina. In *Sepedophilus littoreus*, it is strongly tapered with a thin attachment. In this study, the attachment was not considered as a special feature because it is fully concealed under the procoxae, rather than forming a part of the lobe-like structure. The significant variations of the pronotal postcoxal processes found in the studies taxa suggests that their assessment of homology is needed. Nonetheless, it was not possible to do so in this study due to the difficulty for assessing such a morphologically diverse in- and out-group taxa.

62. Pronotal hypomeron, pronotal postcoxal process and its related areas, base: (0) without suture; (1) with suture that separates postcoxal process from rest of pronotum, resulting in a slightly movable process.

The base of the postcoxal process and related areas of the pronotum is separated by a suture from the rest of the pronotum in Olisthaerinae (see Newton et al. [16]: Figure 63.22).

63. Prosternum, transverse sternacoxal ridge (*tsr*) (sensu Clarke [204]: Figure 2E), medio-lateral areas (ventral view): (0) ridge distinct, moderately distant from anterior margin of prosternum (Figures 11A, 45E, 52A, 54A, 58D and 59D); (1) ridge very close to anterior margin of prosternum (Figures 18C, 24A and 27E); (2) ridge indistinct, trace only, moderately distant from anterior margin of prosternum (Figure 69A,B); (3) ridge far distant from anterior margin of prosternum.

In Mycetoporinae, the transverse sternacoxal ridge (sensu Clarke [204]: Figure 2E) is merely a trace with a row of setae, which is moderately distant from the anterior margin of the prosternum (Figure 69A,B). Tachyporina, Deropini, and most members of Tachinusini have the prominent (not reduced) sternacoxal ridges moderately distant from the anterior margins of the prosternum. In contrast, Euconosomatina and Vatesini have the ridges located very close to the anterior margins of the prosternum.

64. Prosternum, strongly developed prosternal process (ventral view): (0) absent (Figures 18A, 52A, 54A, and 69A); (1) present, partially separating procoxae (Figures 58D and 59D); (2) present, fully separating procoxae.

In Mycetoporinae and Tachyporinae, the medial prosternal process is usually at most only feebly developed. However, some Tachinusini (members of the former Megarthropsini) have a relatively developed prosternal process partially separating the procoxae.

65. Furcasternum, posterior margin (ventral view): (0) various, moderately produced posteriorly, shallowly emarginate to broadly rounded (Figures 11A, 18C, 45E, 54A, and 69A); (1) laterally expanded, contiguous with pronotal projections of hypomeron (Figure 18A).

Uniquely in Tachyporinae, a few *Sepedophilus* (Euconosomatina) have a laterally expanded, modified posterior margin of the furcasternum, thereby closing the procoxal cavities.

66. Procoxal cavities (ventral view): (0) externally open behind (coxa only partially encircled by pronotal postcoxal process and modified furcasternum) (Figures 11A, 18C, 27E, 54G, and 59D); (1) externally completely closed behind, coxa fully encircled by postcoxal process of pronotum and modified furcasternum (Figure 18A); (2) nearly fully or fully open behind, lacking pronotal postcoxal process (Figure 69B).

In several outgroups and Mycetoporinae, the procoxal cavities are fully opened behind because of the absence of the pronotal postcoxal processes. However, in Tachyporinae, they are not fully opened behind because of the weakly to strongly developed pronotal postcoxal processes. In three species of *Sepedophilus* (Euconosomatina), they are fully encircled by the postcoxal process of the pronotum and the modified furcasternum; thus, they are completely closed behind.

67. Mesospiracular peritremes (msp) (sensu Blackwelder [30]: Figure 3A,E), degree of sclerotization [ventral view]: (0) only weakly sclerotized or even membranous (Figure 69A,B); (1) partially well sclerotized; (2) fully well sclerotized, conspicuous (Figures 18A and 52B).

In Mycetoporinae, they are only weakly sclerotized and membranous, but those of most Tachyporinae are large and well sclerotized, except *Leucotachinus* (Tachinusini) (the limited areas with spiracles are sheet or plate-like, extending inward, whereas the out-groups and Mycetoporinae have smaller sheet or plate-like fields, replaced with membranes).

68. Scutellum, prescutoscutellar suture (pss) (sensu Blackwelder [30]: Figure 4A,C) lying transversely [dorsal view]: (0) located near base (Figure 11E); (1) located apically, near middle of scutellum (Figure 69D,E).

In the two subfamilies Mycetoporinae and Staphylininae (*Quedius*), there are two transverse ridges, namely: basal carina (Figure 69D,E, *bc*) and apical carina (Figure 69D,E, ac) (sensu Campbell [32]). The structure of basal carina has been used as one of the diagnostic features of the genera of Mycetoporinae [32,33]. I consider the position of apical carina (or prescutoscutellar suture, sensu Blackwelder [30]: Figure 4A,C) as a subfamily-level character state. In Mycetoporinae, apical carina is distant from the base of the scutellum and located apically near middle of the scutellum, whereas that of Tachyporinae is lying near the base of the scutellum.

69. Elytra (as a pair), overall shape (dorsal view): (0) narrowed posteriorly (Figures 15C, 24D and 51C); (1) widest in middle (Figures 21A and 33B); (2) posteriorly broadened (Figures 51A and 66A).

In Mycetoporinae, the elytra are posteriorly broadened (although *Parabolitobius* is nearly parallel sided). Within Tachyporinae, they are narrowed posteriorly, or widest around the middle of the elytra, in Tachyporini and Vatesini. The elytra are posteriorly broadened in Deropini. Although there are some variations, posteriorly broadened elytra are most common in Tachinusini.

70. Elytron, ratio of length/width: (0) > 1.8× (Figures 44C, 69F); (1) 1.3–1.8× (Figures 11E and 18F); (2) < 1.3× (Figure 30A,B).

The ratio of the elytral maximum length and width were measured usually from slide specimens. A narrowly elongate elytron was found in Mycetoporinae, and therefore, coded as ‘0′. Most taxa of Tachyporini have moderately long elytra, and were scored as ‘1′. Two types of elytra were found in Vatesini: moderately long in earliest diverging genera (*Cileoporus* and *Cilea*, and short in the rest of the tribe. Within the Deropini + Tachinusini clade, the general state is having moderately to strongly elongate elytra. Of note, *Sepedophilus nigeriensis* (= ‘*Urolitus*’ *nigeriensis*; Euconosomatina) was measured using a habitus drawing from the original description [54].

71. Elytron, longitudinal rows of setigerous punctures (excluding ground setae) (dorsal view): (0) absent (Figures 11E, 24D, 30A and 51A,C); (1) present, less than three rows of setigerous punctures; (2) present, three rows of setigerous punctures (Figure 65A); (3) present, more than three rows of setigerous punctures (Figure 65D).

In Mycetoporinae, there are a few to several longitudinal rows of setigerous punctures on each elytron, although they are sometimes difficult to determine (for *Parabolitobius*, see Li et al. [152]: Figures 8 and 12). In this study, each puncture bearing a stout seta was considered for this character state.

72. Elytra, evenness (excluding sutural edge of each elytron) (dorsal view): (0) even (Figures 18F, 30A and 51A); (1) uneven, wrinkled; (2) uneven, with indistinct rows of punctures without deep grooves (Figure 65D); (3) uneven, with distinct rows of punctures with weak to deep longitudinal depressions (Figure 51D); (4) uneven, weakly costate; (5) distinctly uneven, strongly costate.

The elytral surface greatly varies from even to uneven because of the presence or absence of indentations. Since the difficulty of the accurate assessment of homology, they are scored as a single character here. In some genera of Mycetoporinae, there are indistinct rows of punctures without deep grooves. Within Tachyporinae, *Olophrinus* (Tachinusini) is an unusual genus that has several longitudinal striae on each elytron based on distinct discal rows of punctures [126,127]. In *Olophrinus*, each puncture is connected with a longitudinal dent-like impression, forming a shallow groove for each puncture row. This condition is probably not homologous to that in *Neophonus*, but both tentatively scored as the same state due to the difficulty of the accurate assessment of the homology. The extinct tachyporine genus †*Protachinus* from the Late Jurassic Talbragar Fish Bed in New South Wales, Australia, has similar elytral longitudinal striae [52]. The rest of the extant and extinct tachyporines lack such rows of longitudinal striae on the elytra.

73. Elytra, punctation (including setigerous punctures) (dorsal view): (0) absent or fine (Figures 18F, 24D, 30A and 51A); (1) shallow, pit-like (Figures 44C and 51D); (2) deep, pit-like (Figures 58A,C and 60D); (3) somewhat indistinct, mostly distorted (Figure 65A,D).

Some members of Mycetoporinae have somewhat indistinct, mostly distorted punctation on the elytra. In Tachyporinae, they are generally smooth without conspicuous punctation. However, those of Deropini are covered with somewhat deep punctures. In Tachinusini, the general punctation on the elytra is either absent or shallow, but some have deep punctation and *Olophrinus* has longitudinal rows of the punctures as mentioned above (character 72).

74. Elytra, ground microsetae (excluding rows of setigerous punctures) (dorsal view): (0) glabrous (Figures 24D, 30A, 51A, and 65B); (1) vestigial (Figures 8D, 25A and 58C); (2) pubescent (Figures 7B, 11E, 15C, 18F, and 44C).

The elytra of Tachyporini are generally covered with microsetae, whereas the remaining tachyporines lack such elytral microsetae. It should be mentioned, however, that Deropini is densely pubescent, and some Tachinusini have very short, modified setae.

75. Elytron, blackish macrosetae on median area (excluding lateral, inner, or posterior margins) (dorsal view): (0) absent (Figures 30A and 65B); (1) present (Figures 7B, 8A, and 11E).

In most taxa of Tachyporina, the blackish macrosetae are located on the median area of each elytron, but they are absent in the two myrmecophilous genera: *Lamprinodes* and *Lamprinus*.

76. Elytron, blackish macrosetae along lateral margin [dorsal and lateral views]: (0) absent (Figures 15C, 24D, 30A, and 65A); (1) present (Figures 7D, 11E, and 18F).

Within Tachyporinae, the members of Tachyporina and a few *Sepedophilus* species of Euconosomatina have a row of blackish macrosetae located along the lateral margin of each elytron. Additionally, an enigmatic tachyporine genus *Tachinoporus* has a row of five setae laterally on each elytron [11].

77. Elytron, blackish macrosetae along posterior margin [dorsal and lateral views]: (0) absent (Figures 15C, 30A, and 65A); (1) present (Figures 11E and 18F).

Within Tachyporinae, the members of Tachyporina and *Sepedophilus* (*scriptus* species group only; Euconosomatina) have blackish macrosetae arranged along the posterior margins of the elytra. Although I considered them present here in the myrmecophilous *Lamprinodes* of Tachyporina, they are short and rather inconspicuous.

78. Elytra, sutural edge, longitudinally raised suture [dorsal view]: (0) absent or only feebly raised (Figures 7D, 24D, and 51A,C); (1) present, moderately somewhat broadly raised (Figures 65C and 66A); (2) present, markedly strongly raised.

Mycetoporinae have a longitudinally raised suture along the inner edge of each elytron, whereas such raised sutures are absent in Tachyporinae.

79. Elytra, outer margin, epipleural gutter (see Herman [25]) [dorsal view]: (0) absent (Figures 7B, 15C, and 44C); (1) present, very narrow, inconspicuous (Figures 7D, 51A,D, and 65D); (2) present, narrow (Figures 24D and 58A); (3) present, wide, prominent, and resulting in lateral margins of elytra being concave and/or strongly explanate (Figure 58C).

A very narrow, inconspicuous epipleural gutter on the lateral margin of each elytron is found in Mycetoporinae, Tachyporina (except *Palporus*), some Vatesini, and most of Tachinusini. The remaining Vatesini have a narrow epipleural gutter along each elytral outer margin. Such epipleural gutter is absent in Deropini, Euconosomatina, a few members of Vatesini, and *Palporus* (Tachyporina). The strongly expanded, markedly wide epipleural gutters are found in limited members of Tachinusini (= Megarthropsini).

80. Elytral epipleural ridge/keel (Ashe [28]: character 46; Grebennikov & Newton [2]: character 165) (lateral view): (0) present (Figures 52C and 64C); (1) absent.

The elytral epipleural ridge/keel is absent in the outgroup subfamilies Aleocharinae (*Gymnusa* and *Oxypoda*), Phloeocharinae (*Phloeocharis*), Trichophyinae (*Trichophya*), and Staphylininae (*Quedius*).

81. Elytra, folded inward elytral epipleuron, degree of development (ventral view): (0) not or only feebly folded; (1) moderately folded (Figures 18F, 52B,C and 60D); (2) strongly folded (Figure 24A); (3) very strongly, distinctly folded (Figures 24B, 30A,B and 58D).

In Mycetoporinae, the elytral epipleuron is only weakly folded inward, whereas those of Tachyporinae are clearly folded, with a few variations as follows: only feebly folded (Deropini); moderately folded (Tachyporini and most Tachinusini), strongly folded (Vatesini and part of Tachinusini).

82. Elytra, lateral and posterior margins (dorsal view): (0) normal, thick (Figure 15C); (1) thin, blade-like (Figures 25A and 30B,C); (2) of normal thickness on lateral margins, but exceptionally thin in posterior areas, resulting in semitransparent posterior margins of the elytra (Figure 8E).

Vatesini and the two genera of Tachinusini have thin, blade-like edges of the lateral and posterior margins of the elytra. In *Symmixus* (Tachyporina), the posterior areas of the elytra are remarkably thin, resulting in semitransparent posterior margins.

83. Elytra, posterolateral margin (modified from Ashe [28]: character 45) (dorsal view): (0) not sinuate (Figures 11E, 15C, 24D, 30B, 51C, 58A, 65B, and 69F); (1) weakly to moderately sinuate; (2) strongly sinuate (Figures 25A, 30C and 58C).

The posteriorly sinuate margin of the elytron is one of the characters which defines the subfamily Aleocharinae. However, this condition can also be found in Trichophyinae, *Vatesus* (Vatesini), and some Tachinusini (members of the former Megarthropsini).

84. Subapical bulge on each elytron near the posterolateral corner (sensu Hansen [204]) (dorsal view): (0) absent; (1) present.

The presence of a subapical bulge near the posterolateral corner on each elytron is considered one of the possible unique synapomorphies of Silphidae [205].

85. Mesoventrite, longitudinal median carina (ventral view): (0) absent (Figures 11D, 24A,B, 46A, 54F and 70A,D); (1) present, on main part of mesoventrite (Figures 19A, 24C, 28B, 54C,D, 58D and 60A); (2) present, only on mesoventral process, not on main part of mesoventrite (Figure 28C).

There is no longitudinal median carina in Mycetoporinae, whereas this character may have independently evolved several times in Tachyporinae based on the phylogenetic trees presented in this study. In Euconosomatina, all of the *Sepedophilus* taxa studied here have a longitudinal carina on the mesoventrite, but *Euconosoma* lacks this derived character. Within Vatesini, four genera, namely *Cilea*, *Mimocyptus*, *Termitoplus*, and *Coproporus*, have such a carina, whereas it is completely absent in *Cileoporus*, *Vatesus*, and *Coprotachinus*. In *Cilea* (Figure 28C) and *Pseudotachinus* [193,206], however, the carina is found only on the mesosternal process and its basal area, and therefore I have scored them as “2”. Notably, *Tachinoproporus* (Vatesini) has a very strongly developed, plate-like carina. Among the studied taxa of Tachinusini, seven genera, i.e., *Tachinomorphus*, *Olophrinus*, *Austrotachinus*, *Lacvietina*, *Megarthropsis*, *Peitawopsis* and *Nepaliodes*, each have a longitudinal carina on the mesoventrite, and I have scored them as “1”. I could not find this type of carina in Tachyporina and Deropini.

86. Mesoventrite, transverse, long, and straight ridge, lying near base of intercoxal process (ventral view): (0) absent; (1) present (Figures 52A and 54E).

This character was only found in *Leucotachinus* (Tachinusini).

87. Mesocoxae, separation (modified from Ashe [28]: character 58) (ventral view): (0) narrowly to moderately separated (Figures 19A, 24B, 28C, 46A, 54D and 70A,E); (1) partially contiguous (at least 1/5 of mesocoxal length) (Figures 52A, 54E and 70D).

In Mycetoporinae, only *Parabolitobius* and *Mycetoporus* have partially contiguous mosocoxae, whereas they are narrowly separated, divided by the intercoxal processes of the mesoventrite in the remaining mycetoporines. Within Tachyporinae, contiguous mesocoxae are quite unusual and only seen in *Coprotachinus*, *Mimocyptus* (both Vatesini), and part of *Leucotachinus* (Tachinusini).

88. Metaventrite, intermesocoxal process, length (modified from Ashe [28]: character 116) (ventral view): (0) short to subequal to that of intercoxal process of mesoventrite (Figures 19A, 24A, 28C, 46A, 52B, 54F, and 70E); (1) much longer than intercoxal process of mesoventrite; (2) extremely short, only slightly protruding apically (Figure 70A,D); (3) absent or nearly absent, only feebly confirmed (Figure 54E); (4) distinctly short, but with developed and only shallowly depressed isthmus (sensu Maruyama [207]: Figure 7B) projecting apically (Figure 28D); (5) absent or only feebly discernible, but with developed and only shallowly depressed isthmus (sensu Maruyama [207]: Figure 7B) projecting apically (Figures 24B,C and 28A,B).

Within Mycetoporinae, only *Mycetoporus* has a distinctly short, nearly absent, intermesocoxal process of the metaventrite. Most tachyporines have well-developed intermesocoxal processes, but they are nearly absent in some genera of Vatesini, and part of *Leucotachinus* (Tachinusini). Additionally, there is a developed isthmus (sensu Maruyama [207]: Figure 7B) in some genera of Vatesini and *Nitidotachinus* (Tachinusini).

89. Metaventrite, intermesocoxal pit (Herman [25]: character 12) (ventral view): (0) absent; (1) present (Figure 60B).

A deep metaventral pit adjacent to the apex of the mesoventral process is an autapomorphy of *Lacvietina* (Tachinusini) [25].

90. Metendosternite, anterior arms (Naomi [208]: Figure 1B) (dorsal and ventral views): (0) thin, inconspicuous (Figure 54H); (1) relatively thick; (2) thick, well-developed, sometimes lamellate (Figures 11B, 18D, 28G, and 69G); (3) absent.

The metendosternite is well-developed and strongly lamellate in Mycetoporinae. However, there are a few variations in Tachyporinae from non-developed (most members of both Deropini and Tachinusini) to well-developed and slightly lamellate (most members of both Tachyporini and Vatesini).

91. Metendosternite, posterolateral arms (Naomi [208]: Figure 1C) (dorsal and ventral views): (0) absent (Figures 11B, 28G and 54H); (1) present, with developed lamellae (Figure 69G).

Mycetoporinae have a pair of heavily lamellate posterolateral arms in the metendosternite, connected with the anterior (distal) arms by a large lamella. However, I could not find such arms in Tachyporinae.

92. Metendosternite, median process (Naomi [208]: Figure 1H) ([28]: character 61) (dorsal and ventral views): (0) absent (Figures 11B, 28G, 54H and 69G); (1) present (Figure 28I).

Some outgroup taxa have a single, apically pointed process in the middle of the metendosternite. However, this median process is absent in both Mycetoporinae and nearly all taxa of Tachyporinae. Uniquely, *Coprotachinus* (Vatesini) has a short, undeveloped, median process on the metendosternite. This is the only example among the studied tachyporines.

93. Legs, form: (0) normal, typical form (Figure 29A); (1) modified, myrmecophilous form (e.g., flattened, robust, with developed lamellae with protective function, see below) (Figures 12A and 29D).

Within Tachyporinae, a similar type of modified legs was found in the two myrmecophilous genera *Lamprinus* (Tachyporina) and *Vatesus* (Vatesini). In both genera, all legs are short and thick, with strongly developed lamellae extended along the inner margin of each femur. The tibiae are also somewhat flattened and laterally expanded. Furthermore, tarsi are short and thickened, each tarsus appearing compact, reinforced. These leg modifications are known to be adaptations to a myrmecophilous lifestyle, and are considered to perform a protective function against ants [15].

94. Legs, length: (0) moderate to long; (1) distinctly short, compact.

The legs of Mycetoporinae are generally very long, but some variations are found in Tachyporinae. For example, Deropini and *Tachinoporus* (Vatesini) have very slender and long legs. Such long legs were also observed in *Sepedophilus* (Euconosomatina), particularly the hindlegs in which the hindtarsi are exceedingly long. In contrast, the crown genera of Vatesini have distinctly short and compact legs. Two genera of Tachyporina (*Lamprinus* and *Lamprinodes*) also possess thick and strong, but distinctly short, legs in association with myrmecophily.

95. Tibia, distributed stout spines (modified from Ashe [28]: character 47) (dorsal and ventral views): (0) present, conspicuous, irregularly distributed (Figures 12A, 28E, 52C, 66A and 70C); (1) present, inconspicuous, regularly distributed in rows; (2) absent.

It is noteworthy that the mega-diverse ‘higher’ group of Aleocharinae lacks irregularly distributed stout spines on the tibiae, together with some members of the Tachyporine Group of subfamilies, namely Phloeocharinae (*Phloeocharis*) and Trichophyinae (*Trichophya*). All members of Mycetoporinae and Tachyporinae have irregularly distributed, tibial spines. Only a single spine on each tibia was found in *Mimocyptus* (Vatesini), but it was scored here as ‘0′.

96. Procoxa, size (dorsal and ventral views): (0) large, as same as or slightly smaller than profemur (Figure 52A); (1) medium, much smaller than profemur (Figure 45E); (2) very small, globular; (3) distinctly large, much larger than profemur (Figures 12A, 24C, 29A, and 70B).

Mycetoporinae can also be characterized by the presence of markedly large, expanded procoxae. Within Tachyporinae, most tachyporines have large and well-developed procoxae, but Deropini have much smaller ones in proportion to their profemora. The myrmecophilous genus *Lamprinus* (Tachyporina) and the members of Vatesini possess very large procoxae.

97. Protibia, outer margin with longitudinal row of close-spaced spines (modified from Ashe [28]: character 100) (dorsal and ventral views): (0) absent (Figure 12A); (1) present (Figure 17B,C).

This character state is a unique synapomorphy of Euconosomatina, with the only probable exception being the monobasic fossil genus †*Palaeosepedophilus* from Eocene Baltic amber [82].

98. Metacoxae lateral half, size (including dorsal lamellae; see Figure S2) [ventral view]: (0) small (Figures 46B and 58D); (1) medium, much smaller than meso- and metathorax combined (Figures 11C, 15D, 18E, 24A, 52B, 54B, and 60E); (2) markedly large, moderately smaller to nearly as same size as meso- and metathorax combined (Figure 70A and Supplementary Figure S2A).

See morphological definition of Supplementary Figure S2A. The metacoxae of Mycetoporinae are markedly large and conspicuous, whereas they are medium in most taxa of Tachyporinae. However, Deropini and *Nepaliodes* (Tachinusini) have smaller metacoxae.

99. Metacoxae, dorsal lamellae (or plates, *dlmtc*) on metacoxae, lateral areas (ventral view): (0) absent or only weakly developed (Figure 46A); (1) moderately developed (Figures 54B and 60E); (2) well-developed (Figures 18E, 24A, 28E, and 70C).

See morphological definition of Supplementary Figure S2B. The metacoxae of the studied taxa are usually transverse, and at least partially, plate-like. The ‘dorsal plate’ of the metacoxae is generally large and well-developed in Mycetoporinae, Tachyporini, and Vatesini, but they tend to be rather small in size among the members of the Deropini + Tachinusini clade.

100. Metacoxae, ventral lamellae (or plates, vlmtc) on metacoxae (modified from Ashe [28]: character 73) (ventral view): (0) absent (Figures 28H and 70A,C); (1) present, lateral areas, only weakly lamellate, not projecting posteriorly, limited to mesial margins; (2) present, lateral areas, only weakly lamellate, projecting posteriorly, but not developed along mesial margins; (3) present, mesial inner areas, moderately to strongly lamellate, projecting posteriorly, as well as weakly to moderately developed lamellae along lateral margins, partially concealing metatrochanters (Figures 12B, 18E, 28E, 29B, 46B, 54B and 60E); (4) present, very widely expanded, markedly developed.

See morphological definition of Supplementary Figure S2B. Three of these character states (100-1, 100-2, 100-4) only occur in outgroup taxa (see Supplementary Figure S3). The metacoxa of *Olisthaerus substriatus* Paykull, 1790 [85] (Olisthaerinae) is shown in Cai et al. ([209]: Figure 3I). Compared to the transversely developed ‘dorsal plate’, the ‘ventral plate’ is found near the inner area of each metacoxae, usually partially concealing the metatrochanters. Several morphological variations of the ventral plates were found as listed above. Mycetoporinae lacks the ‘ventral plates’ on the metacoxae, whereas all studied taxa of Tachyporinae, except part of *Vatesus* (Vatesini), have developed lamellae on each metacoxa. These lamellae project posteriorly and are weakly to moderately expanded along the outer margins of the metacoxae in Tachyporinae, but such lamellae are completely absent in *Vatesus gigas*.

101. Metatibia, metatibial spurs at apex, length in comparison with metatarsomere 1 (dorsal and ventral views): (0) short, less than 2/5 of metatarsomere 1 (Figures 17D and 45F); (1) long, clearly longer than 2/5 of metatarsomere 1 (Figures 28E, 29C, 54I, 60C, 66A and 69C), but shorter than metatarsomere 1; (2) long, much longer than metatarsomere 1; (3) absent.

Mycetoporinae were scored as either ‘0′ or ‘1′. The latter may be a common state in this subfamily. Regarding Tachyporinae, this character is relatively stable within certain tribes. Most members of Tachyporini and all Deropini were coded as ‘0′. Compared to these tribes, Vatesini and Tachinusini were scored as ‘1′ in this study.

102. Tarsal formula (modified from Ashe [28]: character 50): (0) 5-5-5; (1) 3-3-3.

Both Mycetoporinae and Tachyporinae have a typical 5-5-5 tarsal formula, but *Symmixus* (Tachyporina) appears to have 4-segmented tarsi because of the shortened fourth tarsomeres [22].

103. Metatarsus, length (dorsal and ventral views): (0) long, more than half length of metatibia, but shorter than whole length of metatibia (Figures 7E, 25C, 28E, 29B, and 52C); (1) markedly long, more than whole length of metatibia (Figures 15D and 66A); (2) short, shorter than half length of metatibia (Figure 44C).

The metatarsus of Mycetoporinae is markedly long, exceeding the entire length of metatibia. Within Tachyporinae, such long metatarsi were found only in Euconosomatina. In contrast, some taxa in the clade Deropini + Tachinusini have much shorter metatarsi and were therefore scored as ‘2′.

104. Metatarsomere 3, structure: (0) simple, without ventrally modified projection (Figure 60C); (1) complex, with ventrally modified projection.

*Symmixus* (Tachyporina) has a ventrally modified projection on metatarsomere 3 ([22]: Figure 18).

Abdomen.

105. Abdomen (dorsal view): (0) largely exposed dorsally; (1) moderately exposed dorsally; (2) slightly exposed, largely concealed by elytra.

Within Tachyporinae, only *Leucotachinus* (Tachinusini) has the abdomen largely concealed by long elytra.

106. Abdominal segments V–VI, including paratergites, posterolateral margins (dorsal and ventral views): (0) normal, not extending posteriorly (Figures 7E, 15D and 44C); (1) pointed, slightly but clearly extending posteriorly (Figures 25A,B and 28F); (2) not determined (paratergites absent, not fused with tergite and sternite, separated by sutures); (3) not determined (paratergites absent, completely fused with their tergite and sternite, forming ring-like abdominal segments).

The posterolateral margins of the abdominal segments V–VI are slightly but clearly extended posteriorly in most Vatesini, as well as in *Lamprinus* (Tachyporina) and *Nepaliodes* (Tachinusini).

107. Intersegmental membranes, between abdominal segments III–VI, pattern [dorsal and ventral views]: (0) without ‘brick-wall’ pattern (Figure 12C); (1) with deformed, or somewhat irregular ‘brick-wall’ patterns; (2) with transverse, somewhat rectangular ‘brick-wall’ patterns (Figure 71A,B).

In most outgroup taxa and Mycetoporinae, there is a clear ‘brick-wall’ pattern (sensu Newton et al. [16]) found in the intersegmental membranes between abdominal segments III–VI. However, I have never seen such patterns in Tachyporinae based on the examined material.

108. Abdominal tergite III (dorsal view): (0) well sclerotized; (1) weakly sclerotized or membranous.

The weakly sclerotized abdominal tergite III is considered to be one of the 13 possible unique synapomorphies of Silphidae [205].

109. Abdominal tergites IV–V, macrosetae (dorsal view): (0) absent (Figures 25A and 44C); (1) present, only on posterolateral edges of each tergite (Figures 7E and 15C); (2) present on middle and posterolateral edges of each tergite (Figures 66A, 71B).

Most members of Mycetoporinae have several macrosetae both on the middle of abdominal tergites IV–V and their posterolateral edges. In Tachyporini, these macrosetae are located only on the posterolateral edges of each tergite. Meanwhile, they are completely absent in the rest of Tachyporinae, except for *Nitidotachinus* (Tachinusini), *Leucotachinus* (Tachinusini), *Tachinoporus* (Vatesini), and *Vatesus berghoffae* (Vatesini).

110. Abdominal tergites IV–VI in posterior half (dorsal view): (0) lacking setigerous V-shaped punctures (Figures 7E, 25A, and 44C); (1) covered with setigerous V-shaped punctures (Figures 66A and 71B).

Most mycetoporines have setigerous V-shaped punctures covering abdominal tergites IV–VI, at least in the posterior half of each tergite. This type of pore is not common in Tachyporinae, and it is only seen in a few genera of Vatesini and Tachinusini.

111. Abdominal tergite IV, width (dorsal view): (0) weakly to moderately transverse, less than 2.5 times of its length; (1) strongly transverse, more than 2.5 times of its length.

The maximum width of the tergite IV in Vatesini and Tachinusini is more than 2.5 times wider than its length. However, the remaining Tachyporinae and Mycetoporinae each have a narrower, less transverse, tergite IV.

112. Abdominal tergites VIII–IX (Ashe [28]: character 105): (0) not incorporated into genital apparatus; (1) incorporated into genital apparatus.

The abdominal tergites VIII–IX are incorporated into the genital apparatus in Habrocerinae.

113. Abdominal tergite VIII (male/female), posterior half (excluding macrosetae) [dorsal view]: (0) covered with minute, vestigial microsetae, lacking V-shaped punctures (Figure 31D); (1) covered with minute, vestigial microsetae in V-shaped punctures; (2) covered with short to moderately long setae, lacking V-shaped punctures (Figures 12E, 19C, 31C, 44C, and 71C); (3) covered with short to long setae in V-shaped punctures; (4) covered with short, but strong, thick setae, lacking V-shaped punctures; (5) absent, glabrous (Figure 55C).

In Mycetoporinae, tergite VIII is covered with V-shaped punctures, each usually bearing a short to long seta. However, some mycetoporines lack such V-shaped punctures. In contrast, there are several variations found in Tachyporinae. For example, Tachyporini is generally covered with vestigial to moderately long setae, lacking V-shaped punctures. In Vatesini, tergite VIII is usually covered with minute, vestigial microsetae, lacking V-shaped punctures, with the exceptions of *Coprotachinus*, *Mimocyptus*, and part of *Vatesus*. A similar pattern was found in Tachinusini, which are covered with the minute, vestigial microsetae, lacking V-shaped punctures, with the exceptions of *Nitidotachinus*, *Leucotachinus*, *Peitawopsis*, and *Tachinomorphus*. Within the Deropini-Tachinusini clade, Deropini is the only lineage having short to moderately long setae, lacking V-shaped punctures. It should also be noted that the ground setae of *Megarthropsis* (Tachinusini) are almost lacking. Because of the male abdominal modification, only the female of *Nomimocerus* (Habrocerinae) was considered for this character.

114. Abdominal tergite VIII (male/female), macrosetae (dorsal view): (0) with several or more macrosetae (Figures 19C and 71C); (1) with only a few to several distinct, thick, and long macrosetae (excluding sensory setae, see Yamamoto & Maruyama [210]: Figure 14; occasionally covered together with a few to several small setae) (Figures 31A, 44C and 55C); (2) macrosetae absent (Figure 31B).

Mycetoporinae and Tachyporini have several or more macrosetae on tergite VIII, although these macrosetae in Mycetoporinae are rather difficult to distinguish from the other ground setae. The tachyporine tribes Vatesini, Deropini, and Tachinusini are mostly scored as ‘1′, with the exceptions of *Vatesus gigas* (Vatesini) and *Nitidotachinus* (Tachinusini). Because of the abdominal modifications, only the female of *Nomimocerus* (Habrocerinae) was considered for this character. *Silpha* (Silphidae) was coded here as ‘2′, as I considered them absent because they are too thin to be considered macrosetae.

115. Abdominal tergite VIII (male/female), longest seta, length (dorsal view): (0) shorter than tergite VIII (Figures 12E and 31A); (1) much longer than tergite VIII.

In *Cileoporus* (Vatesini), the longest seta on tergite VIII exceeds the entire length of tergite VIII. *Tachinoporus* (Vatesini) also have long macrosetae on tergite VIII (Figure 39A, D).

116. Abdominal tergite VIII, male, posterior margin (dorsal view): (0) truncate, rounded, or weakly pointed (Figure 70C); (1) lobes developed (Figures 31B and 55C); (2) not confirmed due to highly modified structures of genital segments.

Simple structures were generally found from the male abdominal tergite VIII in the selected outgroups and Mycetoporinae, as they lack developed lobes along the posterior margins of this segment. Contrary to these examples, two tachyporine tribes, namely Vatesini and Tachinusini, have more or less developed lobes. The remaining tachyporine tribes, i.e., Tachyporini and Deropini, have a simple posterior margin of tergite VIII as in most outgroups. In *Austrotachinus* (Tachinusini), the apex of tergite VIII is emarginate, but has a pair of weakly developed ‘lobes’ laterally.

117. Abdominal tergite VIII, female, posterior margin (dorsal view): (0) truncate, rounded, or weakly emarginate medially; (1) pointed medially; (2) deeply notched or emarginate medially; (3) lobes present (Figures 12E, 19C, 31A and 44C).

The posterior margin of the female tergite VIII is simple in the outgroups as well as Mycetoporinae, whereas Tachyporinae, except for part of Deropini, has more or less developed lobes along the posterior margin of this segment.

118. Abdominal tergite IX, male, ventral struts (modified from Ashe [28]: character 71) (dorsal and ventral views): (0) absent (Figures 20F and 33A); (1) present (Figure 72C); (2) not confirmed due to highly modified structures of genital segments.

The ventral struts here refer to the structure of anteriorly directed extra extensions of male tergite IX as figured in Klimaszewski et al. ([196]: Figure 3.5). In most cases, the presence of ventral struts is tightly associated with the following character 119 (i.e., 119-0), but they are different characters because I here considered extra extensions only and did not consider medial connection of the anteroventral margins of the halves of tergite IX. In some outgroups and Mycetoporinae, a pair of ventral struts were confirmed for tergite IX, although they are only weakly developed in two mycetoporines, *Bryoporus rufescens* and *Carphacis*. This structure is uniformly absent in all tribes of Tachyporinae.

119. Abdominal tergite IX, male, base of ventral side (dorsal and ventral views): (0) divided by sternite IX (Figure 72C); (1) basally fused, not divided by sternite IX (Figures 20F, 33D and 61E); (2) not confirmed due to highly modified structures of genital segments.

The ventral side of the base of tergite IX is divided by sternite IX in most outgroups and Mycetoporinae. However, it is fused and not divided in nearly all taxa of Tachyporinae. In Deropini, the ventral lobes of tergite IX are not fused as figured in Smetana ([23]: Figure 4).

120. Abdominal tergite IX, male, structure of dorsal side in basal 1/3 [dorsal view]: (0) longitudinally contiguous (Figures 20A and 33C); (1) divided apically, each side divergent (Figures 33A, 47F and 55E,F); (2) completely continuous (Figure 72A,B); (3) lobes widely separated from each other at base, each lobe directed inward toward apex, with straight sides; (4) not confirmed due to highly modified structures of genital segments.

Several configurations were found in the basal 1/3 of male tergite IX. It is completely continuous, plate-like, in Mycetoporinae, whereas two types of variations were observed in Tachyporinae: the basal 1/3 of male tergite IX is longitudinally contiguous in Tachyporini and *Mimocyptus* (Vatesini), whereas it is divided posteriorly in the basal area, with sides diverging in Vatesini (except *Mimocyptus*), Deropini, and Tachinusini.

121. Abdominal tergite IX, male, structure of dorsal side from basal 1/3 to basal half (dorsal view): (0) longitudinally contiguous (Figure 20A); (1) separated from each other (Figures 33A,C and 55E,F); (2) completely continuous (Figure 72A,B); (3) not confirmed due to highly modified structures of genital segments.

A generally similar result was obtained with the above character (character 120), although it is slightly different.

122. Abdominal tergite IX, male, each apical form [dorsal and lateral views]: (0) short, rounded to pointed apically (Figure 72C); (1) more elongate, with elongate apex (Figures 20A and 33D); (2) elongate, much more slender, forming a lobe, sharply pointed at apex (Figures 33B,C,55G and 61E); (3) elongate, markedly slender, forming two distinct lobes, each sharply pointed at apex (Figures 33A and 55F); (4) not confirmed due to highly modified structures of genital segments.

In Mycetoporinae, the apices of male tergite IX are short and pointed apically. In contrast, a total of three forms listed above were found in Tachyporinae. A pair of elongate apices on this segment were found in Tachyporini and Deropini, but all three types were found in Vatesini, here coded from ‘1′ to ‘3′. The crown group of Vatesini has elongate, lobe-like apices, whereas they are merely elongate and less pointed in the earliest diverging lineage (*Cileoporus*). Interestingly, the vatesine genus *Cilea* has two distinct lobes on each lateral half of the segment. A similar state was found in Tachinusini, and only *Olophrinus* has two pairs of developed lobes as in *Cilea*.

123. Abdominal tergite IX, male, each apical area, macrosetae (dorsal and lateral views): (0) with several or more macrosetae, uniformly covering apical areas (Figures 20A and 72A,C); (1) with numerous macrosetae, restricted only to near and/or near apex; (2) with only single to several prominent macrosetae, sometimes together with a few small setae, restricted to apex (Figures 33C, 47F, 55E,F, and 61E); (3) distinct macrosetae absent; (4) with only a few setae, but not located on apex; (5) not confirmed due to highly modified structures of genital segments.

In Mycetoporinae and Tachyporini, each apical part of the male tergite IX has several or more macrosetae, uniformly covering apical areas. However, Vatesini, Deropini, and Tachinusini have only a single to several prominent macrosetae, restricted to the apex, although they are sometimes associated with a few to several small setae. Notably, the setose male tergite IX was only found in a few tachinusine genera.

124. Paratergites on abdominal segments V–VI (modified from Ashe [28]: character 62) (dorsal and lateral views): (0) single pair (Figures 7E, 25A and 44C); (1) two pairs (Figures 66A and 71B); (2) zero (absent), but abdominal segments are not fused (Figure 15D); (3) zero (absent), abdominal segments are completely fused, ring-like.

Members of Mycetoporinae have two pairs of well-developed paratergites on abdominal segments III–VII. Compared to mycetoporines, there were significant variations found within Tachyporinae. In general, Tachyporinae have only a single pair of paratergites for these segments, but occasionally have two pairs (‘lower’ group of Vatesini) or none (only in Euconosomatina, see also Discussion).

125. Abdominal sternite III, longitudinal median basal carina (ventral view): (0) absent (Figure 12C); (1) present (Figures 19B and 71A).

There is a longitudinal median carina found in the middle of sternite III in Mycetoporinae, Euconosomatina, Deropini, and some members of Tachinusini.

126. Abdominal sternites IV–V, punctures (ventral view): (0) absent to fine, inconspicuous (Figure 25C); (1) coarse, conspicuous, semicircular, pit-like; (2) coarse, conspicuous, V-shaped (Figure 66B).

V-shaped punctures were found in Mycetoporinae, some outgroups, and only limited members of Tachyporinae (Vatesini and Tachinusini).

127. Abdominal sternite VI, male, peg-like setae, characteristically arranged along posteromedial margin (ventral view): (0) absent; (1) present (Figure 52D).

Only two studied taxa of Tachinusini have characteristically arranged peg-like setae along the posteromedial margins of sternite VI in the male.

128. Abdominal sternite VII (male/female), basomedial margin (observable when dissected) (ventral view): (0) without a broad, semicircular protrusion; (1) with a broad, semicircular protrusion (Figure 47A).

In Deropini, there is a peculiar semicircular protrusion located along the basomedial margin of sternite VII in both sexes (Figure 47A; Smetana [23]: Figure 1; Naomi [43]: Figure 3B; Zhao & Li [106]: Figure 2A). A similar, or more pointed, protrusion is occasionally found along each basolateral margin of the sternite VII in Tachinusini (Figure 61A; e.g., Herman [25]: Figure 110), but I am not scoring them here because of the significant variation.

129. Abdominal sternite VII, male, posteromedial margin, emargination [ventral view]: (0) not emarginate (Figure 66B); (1) only weakly emarginate (Figure 55A); (2) moderately to strongly emarginate (Figures 44B, 47A, 52D, and 61A); (3) deeply emarginate, with a pair of large triangular lateral lobes.

The members of Tachinusini have an emargination along the posteromedial margin of male sternite VII. Additionally, a similar emargination was found in *Cilea limbifera* (Vatesini) and two Mycetoporinae, namely *Bolitobius* and *Ischnosoma*. Interestingly, in *Cilea silphoides* (Linnaeus) sternite VII forms two halves of the lateral lobes, resulting in a deep emargination between them. The conditions of *C. silphoides* and *Nomimocerus* (Habrocerinae) are probably not homologous.

130. Abdominal sternite VII, male, posteromedial margin, concavity (ventral view): (0) not concave (Figure 66B); (1) very weakly concave (Figure 55A); (2) moderately to strongly concave (Figures 44B, 47A, 52D and 61A).

The posteromedial margin of sternite VII in the male is usually moderately to strongly concave in Tachinusini, although only weakly concave in some members. Similar concavities were found in a few taxa of Mycetoporinae. However, they are probably not homologous to those of Tachyporinae.

131. Abdominal sternite VII, male, short stout or peg-like setae, characteristically arranged along posteromedial margin [ventral view]: (0) absent (Figures 25C, 66B); (1) present, more than three to dozens of stout setae (not modified as peg-like setae), arranged (Figure 55A); (2) present, more than several to dozens of peg-like setae, arranged, directed posteriorly (Figures 44B, 47A, 52D and 61A).

Most taxa of Tachinusini have several to dozens of characteristically arranged peg-like setae (sensu Hammond [211]) along the posteromedial margin of male sternite VII. In some tachinusines, there are not well-developed into peg-like setae, i.e., *Pseudotachinus* and *Tachinomorphus*.

132. Abdominal sternite VIII (male/female), posterior half, uniform setae (excluding macrosetae) (ventral view): (0) covered with minute, vestigial microsetae, lacking V-shaped punctures (Figures 32A, 55D, and 61B,C); (1) covered with minute, vestigial microsetae in V-shaped punctures (Figure 55B); (2) covered with short to moderately long setae, lacking V-shaped punctures (Figures 12D, 19D,E, 32E, and 71D); (3) covered with short to long setae in V-shaped punctures; (4) absent, glabrous.

In Mycetoporinae, tergite VIII is covered with short to long setae, with or without V-shaped punctures. There are several variations found in Tachyporinae. For example, Tachyporini is generally covered with short to moderately long setae, lacking V-shaped punctures. In Vatesini, tergite VIII is usually covered with the minute, vestigial microsetae, lacking V-shaped punctures, with the exceptions of *Coprotachinus*, *Mimocyptus*, and *Vatesus*. A similar pattern was found in Tachinusini, which are covered with minute, vestigial microsetae, generally lacking V-shaped punctures, with some exceptions. Within the Deropini + Tachinusini clade, Deropini is the only tribe having moderately long ground setae, lacking V-shaped punctures. Because of the abdominal modification, only the female of *Nomimocerus* (Habrocerinae) was considered for this character.

133. Abdominal sternite VIII (male/female), macrosetae (ventral view): (0) with several or more macrosetae (Figures 12D, 19D,E, 55D and 71D); (1) with only a few to several distinct, thick, and long macrosetae (excluding sensory setae, see Yamamoto & Maruyama [210]: Figure 14; occasionally covered with a few to several small setae) (Figures 32A, 47A, 55B and 61B,C); (2) macrosetae absent (Figure 32D).

Mycetoporinae and Tachyporini have several or more macrosetae on tergite VIII of both sexes, although these macrosetae in Mycetoporinae are rather difficult to distinguish from the ground setation. Within a clade comprised of the three tribes, namely Vatesini, Deropini, and Tachinusini, this character was scored as ‘1′, with the exceptions of *Vatesus gigas* (Vatesini) and *Nitidotachinus* (Tachinusini). Because of the abdominal modification, only the female was considered for *Nomimocerus* (Habrocerinae). *Silpha* (Silphidae) was here coded as ‘2′, as I considered them absent because they are too thin to be considered macrosetae.

134. Abdominal sternite VIII, male, characteristically arranged microsetae or peg-like setae on postero-medial area (excluding macrosetae) (dorsal view): (0) absent (Figure 71E); (1) present, only at apex (Figure 71F); (2) present, numerous, longitudinally distributed (Figures 52D, 66B and 71G).

In some genera of Mycetoporinae, some characteristically arranged microsetae, or peg-like setae, are found on sternite VIII of the male. They are either restricted to the apex (*Bobitobus* and *Bryoporus*) or cover a much wider area (*Bolitobius*, *Bolitopunctus*, *Carphacis*, and *Ischnosoma*). Within Tachyporinae, only *Nitidotachinus* (only one species, *N*. *tachyporoides*) has such clusters of microsetae along inner margins of the deep emargination of sternite VIII.

135. Abdominal sternite VIII, male, posterior margin (ventral view): (0) truncate, rounded, only weakly emarginate, or weakly pointed (Figure 71E); (1) rather narrowly, moderately emarginate medially (Figure 71H); (2) rather widely deeply emarginate medially (Figures 19E and 32D); (3) much more deeply incised or very strongly emarginate medially (Figure 47B); (4) rather widely deeply emarginate medially, with a few feebly developed lobes laterally (Figures 32E and 55B); (5) lobes developed (Figures 32D and 61B); (6) not confirmed due to highly modified structures of genital segments.

In general, a series of simple structures were found in male sternite VIII within the selected outgroups and most members of Mycetoporinae which lack well-developed lobes along the posterior margins of male sternite VIII. However, the apex is generally more or less modified into lobes in Tachyporinae, with several variations as listed above. In Tachyporini and *Vatesus* (Vatesini), they were uniformly deeply, rather widely emarginate medially. In Vatesini, three types of modifications were found, namely ‘2′, ‘4′, and ‘5′. All three species of Deropini unusually lack such lobe-like modifications, and are coded here as ‘3′. The most common state of Tachinusini is ‘5′ which have well-developed lobes. Within Tachinusini, there are a few feebly developed lobes posterolaterally in *Nitidotachinus*, *Pseudotachinus*, and *Leucotachinus luteonitens*, here scored as ‘4′.

136. Abdominal sternite VIII, male, central area (ventral view): (0) not distinctly deeply and narrowly incised (Figures 19E, 32D, 71E, H); (1) distinctly deeply and narrowly incised (Figure 32C,E and 61B); (2) distinctly deeply and relatively widely incised or emarginate (Figures 32C,E, 47B, 55B and 61B).

In some members of Vatesini, Deropini, and Tachinusini, sternite VIII in the male is deeply incised or emarginate.

137. Abdominal sternite VIII, female, posterior margin (ventral view): (0) truncate, rounded, weakly emarginate medially, or weakly pointed (Figures 19D and 71D); (1) deeply emarginate or incised medially; (2) nearly rounded, with only a pair of feebly developed projections (Figures 12D and 47E); (3) lobes present, including small internal lobes (Figures 32A, 55D, and 61C); (4) strongly pointed medially.

The posterior margin of female sternite VIII is simple in the outgroups and Mycetoporinae, whereas Tachyporinae have several variations. For example, in Tachyporini, it is merely truncate or rounded in Euconosomatina, whereas it is slightly different in Tachyporina, nearly rounded but generally having a pair of feebly developed posteriorly directed projections. In Tachyporina, a pair of similar projections was found in Deropini and *Leucotachinus* (Tachinusini) along the nearly rounded posterior margins. The rest of Tachinusini and all of Vatesini, have a few pairs of well-developed lobes.

138. Abdominal sternite VIII, female, arranged sensory setae along lobes or in alternative positions on posterior margin (ventral view): (0) prominent lobes absent, lacking rows of minute sensory setae (Figure 71D); (1) prominent lobes absent, with single row of minute sensory setae, distributed uniformly along posterior margin; (2) prominent lobes absent, with single row of minute sensory setae distributed along projections or in alternative positions on posterior margin, but limited to median area of posterior margin (Figures 12D, 19D and 47E); (3) prominent lobes present, inner lobes with only a pair or three minute sensory setae around apex of each lobe (Figure 32B); (4) prominent lobes present, inner lobes with rows of more than three, minute sensory setae in fan-like arrangement, located in apical area of each lobe (Figures 55D and 61C).

In the aleocharine taxa used here, a row of the minute sensory setae (sensu Yamamoto & Maruyama [210]: Figure 14) widely covers the posterior margins of sternite VIII in the female. Compared to the outgroups and Mycetoporinae, there are a few variations found in Tachyporinae. Two tribes, Tachyporini and Deropini, lack well-developed lobes, but a single row of minute sensory setae rather narrowly covers the middle area of the posterior margins. All members of Vatesini, except *Vatesus gigas*, have pairs of well-developed lobes and their associated inner lobes, with only a pair, or at most three, minute sensory setae located around the apex of each lobe. Tachinusini has a similar character state to Vatesini, but generally has more than three setae at the apex of each inner lobe. The condition of *Austrotachinus* (Tachinusini) is rather difficult to interpret and was tentatively scored here as ‘4′ because it has a row of dense minute setae (although it is located between lateral inner lobes, instead of near the apices of each inner lobe).

139. Abdominal genital segments in female, general shape (excluding exposed gonocoxites and styli) (dorsal and ventral views): (0) transverse to only weakly elongate (Figures 12G, 20E, 34B, 47C and 61D); (1) moderately to strongly elongate (Figures 34A and 73B).

The outgroups and Mycetoporinae generally have moderately to strongly elongate genital segments in the female. In contrast, they are transverse to only weakly elongate in almost all members of Tachyporinae with the exception of *Vatesus* (Vatesini). A vatesine species, *Coproporus laevis*, was considered here as having narrowly elongate female genital segments.

140. Abdominal genital segments in female, basal part [dorsal and ventral views]: (0) well sclerotized (Figure 73B); (1) only weakly sclerotized and/or partially membranous (Figures 12G, 20E, 34B, 47C, and 61D).

In Mycetoporinae, the basal part of the female genital segments is well sclerotized. However, in Tachyporinae, they are only weakly sclerotized and even partially membranous in some cases, with only a handful of exceptions in this study (i.e., *Vatesus* in Vatesini and *Leucotachinus luteonitens* in Tachinusini).

Genitalia.

Male:

141. Internal sac, spines (modified from Ashe [28]: character 77): (0) absent (Figures 12F, 20B,C, 33E, 56A and 72E); (1) few or insignificant; (2) many and prominent.

In Trichophyinae and Habrocerinae, the internal sac includes numerous, prominent large spines. Some outgroups also have a few or insignificant spines on the internal sac. The vatesine genus *Tachinoproporus* has such prominent spines (Figure 42B, *dsp*) although this structure needs further observation.

142. Parameres, mesial area, degree of attachment to median lobe (modified from Jenkins Shaw et al. [212]: character 67) (lateral and parameral views): (0) paramere(s) well separated from median lobe (Figures 66C, 72F); (1) parameres very closely appressed to median lobe (Figures 33E,G, 56C,D and 61F); (2) parameres absent; (3) not confirmed because of highly modified structures of genitalia.

All examined taxa of Tachyporinae in this study have a pair of parameres which are closely appressed to the median lobe of the aedeagus for nearly its whole length. In Mycetoporinae and the outgroups, they are generally well separated from the median lobe. The configuration and structure of the male genitalia of Habrocerinae are complicated, and the presence of parameres has not been confirmed in that subfamily [192].

143. Parameres, apical inner margins (parameral view): (0) widely separated from each other (Figures 66C and 72F); (1) moderately separated (Figures 33H and 56A); (2) only narrowly separated longitudinally (Figures 12F, 20B and 33F); (3) contiguous longitudinally, nearly contiguous, or nearly fused (Figures 20C, 47F, 56B,D, and 61F); (4) completely fused, forming single structure; (5) parameres absent; (6) not confirmed due to highly modified structures of genitalia.

All members of Mycetoporinae have a pair of widely separated parameres, however, those of Tachyporinae are much more narrowly separated, with three variations from ‘1′ to ‘3′.

144. Parameres, apical half, structure (lateral and parameral views): (0) simple (Figures 20B, 33F, and 72D); (1) complex, modified; (2) very complex, multi-articulated; (3) parameres absent; (4) not confirmed due to highly modified structures of genitalia.

The general structures of the parameres in Mycetoporinae and Tachyporinae are exclusively simple, ranging from clavate to plate-like. Multi-articulated, complex parameres can only be found in Aleocharinae.

145. Parameres, shape (ventral view): (0) not flattened or plate-like ventrally (Figures 12F, 20B, 33F, 56A and 72F); (1) widely or rather widely flattened or plate-like ventrally (Figures 47F, 56B and 61F); (2) parameres absent; (3) not confirmed due to highly modified structures of genitalia.

Within Tachyporinae, the general shape of parameres is relatively diverse with several variations, though flattened or plate-like parameres can be found uniformly in the Deropini + Tachinusini clade (except *Leucotachinus luteonitens* in Tachinusini). The mycetoporine genus *Parabolitobius* has a pair of apically flattened and enlarged parameres, but it was coded here as ‘0′ based on general overall shape and structure. In *Tachinomorphus* (Tachinusini), parameres are flattened but fused longitudinally in basal area.

146. Parameres, velum (modified from Ashe [28]: character 82): (0) absent (Figure 72E); (1) present; (2) parameres absent; (3) not confirmed due to highly modified structures of genitalia.

Aleocharinae has a velum on each paramere [190], but it is completely absent, or not confirmed, in the other staphylinid groups, including Mycetoporinae and Tachyporinae.

147. Parameres, number of setae on each paramere (excluding minute sensilla): (0) zero (absent) (Figures 12F, 20B, and 33F,H); (1) present, less than five (Figure 72D); (2) present, five or more (Figure 72E); (3) parameres absent; (4) not confirmed due to highly modified structures of genitalia.

All members of Tachyporinae lack setae on each paramere, however, Mycetoporinae have a great variation in number, length, and distribution of such setae.

148. Parameres, minute sensilla or filiform setulae: (0) absent (Figures 12F, 20B, 33F and 56B); (1) present, a few to several (Figure 72D); (2) present, numerous (Figure 56C); (3) parameres absent; (4) not confirmed due to highly modified structures of genitalia.

Within Tachyporinae studied here, *Tachinomorphus* (Tachinusini) was the only taxon that has numerous filiform setulae on each paramere. The rest of the Tachyporinae lack these setulae. More than half of the taxa of Mycetoporinae have such minute sensilla, but they are insignificant in most cases, with only a few to several sensilla or setulae.

149. Parameres, setae, arrangement (excluding minute sensilla): (0) absent; (1) present, lacking a row of parameral setae; (2) present, each with a row of parameral setae at least partially aligned (Figure 72D); (3) parameres absent; (4) not confirmed due to highly modified structures of genitalia.

In the outgroups, parameral setae do not form a row, but alternatively are scattered on the apex of each paramere. However, Mycetoporinae have a distinct, longitudinal row of these setae, at least partially aligned.

150. Parameres, setae, location (excluding minute sensilla): (0) absent; (1) present, limited only to apex of paramere (Figure 72F); (2) present, not limited to apex of paramere (Figure 72E); (3) parameres absent; (4) not confirmed due to highly modified structures of genitalia.

The parameral setae are located in the apices of the parameres in the outgroups. In Mycetoporinae, they are either limited to the apices or are much more widely distributed on the parameres.

Female:

151. Spermatheca: (0) absent, or very weakly sclerotized, inconspicuous, oblong to cresent shaped (Figure 34A); (1) well sclerotized, comparatively complex or forming a distinctly complicated structure, usually associated with coils (Figures 56F, 61D and 73C).

In some Mycetoporinae and Tachyporinae (most taxa of Tachinusini), a well sclerotized spermatheca with a complex structure, such as basally nested coils, was found.

152. Gonocoxites I and/or II (modified from Ashe [28]: character 89) (dorsal view): (0) present (Figure 47C); (1) present, but reduced to spinose process; (2) absent.

A single taxon used here, *Oxypoda* sp. (Aleocharinae: Oxypodini), does not have clearly developed, lobe-like gonocoxites. This condition is universal among the diverse members of the ‘higher’ group of Aleocharinae.

153. Gonocoxite II, shape (dorsal view): (0) slender, narrowly elongate (Figures 12G, 20E, 47D, 56E, 61D); (1) rather thick, more or less triangular (Figure 34A); (2) thick, narrowly elongate (Figures 34C and 73A); (3) thick, narrowly elongate, fused with gonocoxite I; (4) flattened, rectangular; (5) lobe-like gonocoxite II absent.

A thick and narrowly elongate gonocoxite II is uniformly found in Mycetoporinae. In Tachyporinae, it is generally much more slender, with the exception of most taxa of Vatesini and some Tachinusini. Two types of gonocoxite II were found in Vatesini: (i) rather thick, more or less triangular (*Coproporus*, *Coprotachinus*, *Mimocyptus*, *Termitoplus*, and *Vatesus gigas*); (ii) thick, narrowly elongate (*Cilea* and *Vatesus praedatorius*).

154. Gonocoxite II, size in comparison with gonostylus (dorsal view): (0) small (Figures 12G, 20D, 34A, 47C,D, and 56E); (1) large (Figure 73A); (2) lobe-like gonocoxite II absent.

The overall size of gonocoxite II in the outgroups, including Mycetoporinae, is usually large and prominent in comparison with the gonostylus. In contrast, they are much smaller in Tachyporinae.

155. Gonocoxite II (I and II, if they are fused together), setae [dorsal view]: (0) covered with normal to stout setae (Figure 73B); (1) covered with curved setae (strongly bent backwards) (Figures 12G, 20D, 47D, and 56E); (2) covered with both normal and curved setae (Figure 34A,C); (3) covered with long, strong setae; (4) covered with only a few scattered setae; (5) lobe-like gonocoxite II absent.

Gonocoxite II is covered with normal to stout setae in Mycetoporinae, but that of Tachyporinae are generally uniformly covered with curved setae. Although the presence of curved setae may be a universal condition in the entire subfamily (except *Peitawopsis* in this study), both normal and curved setae were found together on gonocoxite II in Vatesini.

156. Gonostylus (modified from Grebennikov & Newton [2]: character 260) (dorsal view): (0) absent; (1) present, but minute and inconspicuous (Figure 73D); (2) present, large and conspicuous (Figures 12G, 20D, 34A, 47D, and 56E); (3) strongly sclerotized, relatively large, sharply pointed.

Mycetoporinae only has a pair of weakly developed, minute gonostyli, but those of Tachyporinae are much larger and conspicuous.

## Appendix C. Table

**Table A1:** General overview of Tachyporinae sensu nov. under the new classification.

New Classification	Former Tribal Assignment	Described Species	Distribution
Tachyporini MacLeay, 1825 [40] sensu nov.		**510 (†6)**	AUS, ETH, MAD, NEA, NEO, OCE, ORI, PAL
-Subtribe Tachyporina MacLeay, 1825 [40] stat. nov., sensu nov.		144 (†4)	NEA, NEO, ORI, PAL
1. *Lamprinodes* Luze, 1901 [57]	Tachyporini	3	PAL
2. *Lamprinus* Heer, 1839 [59]	Tachyporini	1	PAL
3. *Palporus* Campbell, 1979 [61] stat. nov.	Tachyporini	2	PAL; intro. AUS, NEA, OCE
4. *Symmixus* Bernhauer, 1915 [63]	Tachyporini	2	ORI, PAL
5. *Tachyporus* Gravenhorst, 1802 [41] sensu nov. [= †*Mesotachyporus* Gusarov, 2000 [56] syn. nov.]	Tachyporini	136 (†4)	NEA, NEO, ORI, PAL
-Subtribe Euconosomatina Cameron, 1918 [44] stat. rev., sensu nov.		366 (†2)	AUS, ETH, MAD, NEA, NEO, OCE, ORI, PAL
6. *Euconosoma* Cameron, 1918 [44]	Tachyporini	4	ORI, PAL
7. *Sepedophilus* Gistel, 1856 [84] [= *Urolitus* Silvestri, 1947 [54] syn. nov.]	Tachyporini	361 (†1)	AUS, ETH, MAD, NEA, NEO, OCE, ORI, PAL
8. †*Palaeosepedophilus* Paśnik & Kubisz, 2002 [82]	Tachyporini	†1	Europe (Baltic Sea Coast) [mid-Eocene Baltic amber]
Vatesini Seevers, 1958 [13] sensu nov.		**290 (†2)**	AUS, ETH, MAD, NEA, NEO, OCE, ORI, PAL
1. *Cilea* Jacquelin du Val, 1856 [93]	Tachyporini	19	ETH, MAD, NEA, NEO, ORI, PAL
2. *Cileoporus* Campbell, 1994 [91]	Tachyporini	2	NEO
3. *Coproporus* Kraatz, 1857 [96]	Tachyporini	229 (†1)	AUS, ETH, MAD, NEA, NEO, OCE, ORI, PAL
4. *Coprotachinus* Cameron, 1933 [98]	Tachyporini	7	ETH
5. *Mimocyptus* Cameron, 1919 [101]	Tachyporini	2	AUS, ORI
6. *Tachinoporus* Cameron, 1928 [11]	Tachyporini	1	ORI
7. *Tachinoproporus* Cameron, 1928 [11]	Tachyporini	1	ORI
8. *Termitoplus* Silvestri, 1946 [12]	Tachyporini	1	NEO
9. *Vatesus* Sharp, 1876 [92]	Vatesini	27	NEO
10. †*Procileoporus* Yamamoto, 2016 [46]	Tachyporini	†1	Myanmar (mid-Cretaceous Kachin amber)
Deropini Smetana, 1983 [23]		**20**	NEA, ORI, PAL
1. *Derops* Sharp, 1889 [105]	Deropini	20	NEA, ORI, PAL
Tachinusini Fleming, 1821 [45] stat. rev., sensu nov.		**371 (†7)**	AUS, ETH, MAD, NEA, NEO, ORI, PAL
1. *Austrotachinus* Steel, 1956 [110]	Tachyporini	1	AUS
2. *Lacvietina* Herman, 2004 [25]	Megarthropsini	9	ORI, PAL
3. *Leucotachinus* Coiffait & Sáiz, 1968 [111]	Tachyporini	3	AUS, NEO
4. *Megarthropsis* Cameron, 1919 [101]	Megarthropsini	8	ORI
5. *Nepaliodes* Coiffait, 1977 [113]	Megarthropsini	2	ORI, PAL
6. *Nitidotachinus* Campbell, 1993 [109]	Tachyporini	16	NEA, ORI, PAL
7. *Olophrinus* Fauvel, 1895 [115]	Tachyporini	15	ORI, PAL
8. *Peitawopsis* Smetana, 1992 [116]	Megarthropsini	3	PAL
9. *Pseudotachinus* Cameron, 1932 [117]	Tachyporini	5	ORI, PAL
10. *Tachinomorphus* Kraatz, 1859 [118]	Tachyporini	21	AUS, ETH, MAD, NEA, ORI, PAL
11. *Tachinoplesius* Bernhauer, 1936 [120]	Tachyporini	9	ETH
12. *Tachinus* Gravenhorst, 1802 [41]	Tachyporini	273 (†1)	NEA, NEO, ORI, PAL
- Subgenus *Latotachinus* Ullrich, 1975 [121]	Tachyporini	7	PAL
- Subgenus *Tachinoderus* Motschulsky, 1858 [123]	Tachyporini	60	NEA, ORI, PAL
- Subgenus *Tachinus* Gravenhorst, 1802 [41]	Tachyporini	204 (†1)	NEA, NEO, ORI, PAL
13. †*Hesterniasca* Zhang, Wang & Xu, 1992 [124]	N/A	†2	China (Lower Cretaceous) *
14. †*Mesotachinus* Tikhomirova, 1968 [50]	N/A	†3	Kazakhstan (Middle–Upper Jurassic) *
15. †*Protachinus* Cai, Yan, Beattie, Wang & Huang, 2013 [52]	Tachyporini	†1	Australia (Upper Jurassic) *
Tachyporinae, *incertae sedis*		†3	
1. †*Abscondus* Tikhomirova, 1968 [50]	N/A	†2	Kazakhstan (Middle–Upper Jurassic) *
2. †*Tachyporoides* Tikhomirova, 1968 [50]	N/A	†1	Kazakhstan (Middle–Upper Jurassic) *
TOTAL: **36 (†7) genera**		**1194 (†18)**	

The data shown here is based on an unpublished database compiled by A. F. Newton (FMNH), with permission (as of 4 March 2021). See also Appendix A for a note on the species number of *Latotachinus*. Abbreviations: AUS, Australia; ETH, Ethiopian; MAD, Madagascar; NEA, Nearctic; NEO, Neotropical; OCE, Oceanic; ORI, Oriental; PAL, Palaearctic. N/A: not applicable. The bold numers indicate a total number of species in each family-group category. * The fossil taxa indicated here with the asterisks are compression fossils.

**Table A2:** General overview of Mycetoporinae stat. nov. (members of the former Mycetoporini in Tachyporinae) under the new classification.

New Classification	Former Tribal Assignment	Described Species	Distribution
1. *Bobitobus* Tottenham, 1939 [140] stat. rev.	Mycetoporini	34	NEA, ORI, PAL
2. *Bolitobius* Leach, 1819 [141]	Mycetoporini	21	PAL; intro.? NEA
3. *Bolitopunctus* Campbell, 1993 [33]	Mycetoporini	2	NEA
4. *Bryophacis* Reitter, 1909 [144]	Mycetoporini	12	NEA, PAL
5. *Bryoporus* Kraatz, 1857 [96]	Mycetoporini	37	ETH, NEA, NEO, ORI, PAL
6. *Canariobolitobius* Schülke, 2004 [146]	Mycetoporini	1	PAL
7. *Carphacis* Gozis, 1886 [148]	Mycetoporini	16	NEA, PAL
8. *Ischnosoma* Stephens, 1829 [150]	Mycetoporini	118	AUS, ETH, NEA, NEO, ORI, PAL
9. *Lordithon* Thomson, 1859 [136] sensu nov.	Mycetoporini	97 (†4)	AUS, NEA, NEO, ORI, PAL
10. *Mycetoporus* Mannerheim, 1831 [137]	Mycetoporini	88 (†1)	NEA, PAL
11. *Neobolitobius* Campbell, 1993 [33]	Mycetoporini	1	NEA
12. *Parabolitobius* Li, Zhao & Sakai, 2000 [152]	Mycetoporini	13 (†1)	PAL
13. †*Cuneocharis* Ryvkin, 1990 [153]	N/A	†1	Russia (Upper Jurassic) *
14. †*Glabrimycetoporus* Yue, Zhao & Ren, 2009 [154]	Mycetoporini	†1	China (Lower Cretaceous) *
15. †*Ryvkinius* Herman, 2001 [67]	N/A	†1	Russia (Upper Jurassic) *
16. †*Undiatina* Ryvkin, 1990 [153]	N/A	†1	Russia (Upper Jurassic) *
TOTAL: **16 (†4) genera**		**444 (†10)**	

The data shown here is based on an unpublished database compiled by A. F. Newton (FMNH), with permission (as of 4 March 2021). Abbreviations: AUS, Australia; ETH, Ethiopian; NEA, Nearctic; NEO, Neotropical; ORI, Oriental; PAL, Palaearctic. N/A: not applicable. The bold numers indicate the total numbers of genera and species in Mycetoporinae. * The fossil taxa indicated here with the asterisks are compression fossils.
