# Correction: Yamamoto, S. Tachyporinae Revisited: Phylogeny, Evolution, and Higher Classification Based on Morphology, with Recognition of a New Rove Beetle Subfamily (Coleoptera: Staphylinidae). *Biology* 2021, *10*, 323

**DOI:** 10.3390/biology11081124

**Published:** 2022-07-27

**Authors:** Shûhei Yamamoto

**Affiliations:** 1 Integrative Research Center, Field Museum of Natural History, 1400 S Lake Shore Drive, Chicago, IL 60605, USA; syamamoto@fieldmuseum.org; 2 The Hokkaido University Museum, Hokkaido University, Kita 8, Nishi 5, Kita-ku, Sapporo 060-0808, Japan

The author would like to make the following corrections to his published paper [1]. The corrections include:

On page 1, in the Abstract, replace the sentence “*Urolitus* syn. nov. is placed as a junior synonym of *Sepedophilus*, and *Palporus* stat. nov. is raised to a distinct genus from a subgenus of *Tachyporus* sensu. nov., and †*Mesotachyporus* syn. nov. is treated as synonym of the latter.” with “*Urolitus* syn. nov. is placed as a junior synonym of *Sepedophilus*. Additionally, *Palporus* stat. nov. is raised to a distinct genus from a subgenus of *Tachyporus* sensu. nov., and †*Mesotachyporus* syn. nov. is synonymized with the latter.”.

On page 1, in the Introduction, replace the sentence “Among one extinct and 32 extant subfamilies [1], Tachyporinae are a medium-sized group at the subfamily level and currently comprised of 1638 species (28 extinct) of 40 genera (12 extinct) in five tribes as of 9 April 2021 (A.F. Newton, pers. comm.; Appendix A).” with “Among one extinct and 32 extant subfamilies [1], Tachyporinae is a medium-sized group at the subfamily level and currently comprises 1638 species (28 extinct) of 52 genera (12 extinct) in five tribes as of 9 April 2021 (A.F. Newton, pers. comm.; Appendix A).”.

On page 3, in the figure caption of Figure 1, replace the text “(**E**) *Lordithon* (*Lordithon*) *bicolor* (Gravenhorst) (Mycetoporini) in Kumamoto Pref., Japan.” with “(**E**) *Lordithon* (*Lordithon*) *bicolor* (Gravenhorst) (Mycetoporini, now Mycetoporinae) in Kumamoto Pref., Japan.”.

On page 5, in Materials and Methods “Section 2.2. Taxon Sampling and Deposition of Material”, paragraph 2, replace the text “A total of 71 operational taxonomic units (OTUs) were scored for the cladistic analyses” with “A total of 70 operational taxonomic units (OTUs) were scored for the cladistic analyses”.

On page 6 in Materials and Methods “Section 2.5. Phylogenetic Analyses”, replace the sentence “My final data matrix includes 156 characters scored for 71 terminal taxa (57 ingroup Tachyporinae, 14 non-tachyporine outgroups).” with “My final data matrix included 156 characters scored for 70 terminal taxa (57 ingroup Tachyporinae, 13 non-tachyporine outgroups).”.

On page 16, in “Section 3.2. Systematic Part”, 3.2.1. Subfamily Tachyporinae MacLeay sensu nov., Description, replace the text “Labial palpus (e.g., Figures 9E, 16F, 26B,E,F, 45B, 53B,D,E,G and 59B) 3-segmented, inconspicuous.” with “Labial palpus (e.g., Figures 9E, 16F, 26B,E,F, 45B, 53B,D,E,G and 59B) 3-segmented, inconspicuous, except *Euconosoma*.”.

On page 21, in “Section 3.2. Systematic Part”, 3.2.1. Subfamily Tachyporinae MacLeay sensu nov., Composition, replace the text “Four tribes of 36 genera (7 extinct), with 1191 species (18 extinct). See Table A1 for overview and distributions.” with “Four tribes of 36 genera (7 extinct), with 1194 species (18 extinct). See Table A1 for overview and distributions. See also Appendix A.”.

On page 41, in “Section 3.2. Systematic Part”, 3.2.7. Tribe Vatesini Seevers, 1958 sensu nov., Emended diagnosis, replace the text “abdomen rather strongly to very strongly tapering posteriorly, lacking blackish macrosetae dorsally (except abdominal terminalia)” with “abdomen rather strongly to very strongly tapering posteriorly, lacking blackish macrosetae dorsally (except abdominal terminalia), except *Tachinoporus* and *Vatesus berghoffae* Kistner, 2006, which have scattered erect macrosetae on the abdominal tergites”. See Kistner & Berghoff [2].

On page 51, in “Section 3.2. Systematic Part”, 3.2.7. Tribe Vatesini Seevers, 1958 sensu nov., Description, replace the text “Tergites III–VII (Figure 25A: 109-0) without macrosetae (except *Tachinoporus*; Figure 35C,D)” with “Tergites III–VII (Figure 25A: 109-0) without macrosetae (except *Tachinoporus* and *Vatesus berghoffae*; Figure 35C,D)”.

On page 78, in “Section 3.2. Systematic Part”, 3.2.13. Tribe Tachinusini Fleming, 1821 stat. rev., sensu nov., replace the text “=Megarthropsini Cameron, 1919 [101]: 231 syn. nov.” with “=Megarthropsini Cameron, 1919 [101]: 231 syn. nov. (type genus: *Megarthropsis* Cameron, 1919 [101]: 231).”.

On page 90, in “Section 3.2. Systematic Part”, 3.2.13. Tribe Tachinusini Fleming, 1821 stat. rev., sensu nov., replace the text “Pronotum (Figures 51 and 58) of typical Tachyporinae sensu nov. from except in *Nepaliodes* (Figure 58C: 49-4), usually widest between basal 1/4 to middle (Figures 51, (A) (B) 58B: 50-3)” with “Pronotum (Figures 51 and 58) of typical Tachyporinae sensu nov. form except in *Nepaliodes* (Figure 58C: 49-4), usually widest between basal 1/4 to middle (Figures 51A,B and 58A: 50-3)”.

On pages 109 and 110, in “Section 3.2. Systematic Part”, 3.2.16. Genus *Bobitobus* Tottenham, 1939 stat. rev., the author erroneously used “comb. rev.” instead of “comb. nov.” for the *Bobitobus* species on both pages. All instances of “comb. rev.” should be replaced with “comb. nov.” throughout the text, except *Bobitobus pulchellus* (Mannerheim, 1830) based on the previous taxonomic treatments [3,4].

On page 113, in “Section 3.3. Identification Keys”, 3.3.4. Key to Extant Genera of Vatesini, replace the text “5. Body minute (length: ca. 1.0 mm) (Figure 21G)” with “5. Body minute (length: ca. 1.2 mm) (Figure 21G)”.

On page 116, in “Section 3.3. Identification Keys”, 3.3.6. Key to Extant Genera of Mycetoporinae, replace the text “7. Labial palpomere 3 longer than labial palpomeres 2 and 3 combined (Figure 67F) … 8” and its subsequent text “- Labial palpomere 3 shorter than labial palpomeres 2 and 3 combined (cf. Figure 67E) … 10” with “7. Labial palpomere 3 longer than labial palpomeres 1 and 2 combined (Figure 67F) … 8” and “- Labial palpomere 3 shorter than labial palpomeres 1 and 2 combined (cf. Figure 67E) … 10”, respectively.

On page 139, in “Appendix B. List of Characters”, Character 109, replace the sentence “Meanwhile, they are completely absent in the rest of Tachyporinae, except *Nitidotachinus* and *Leucotachinus* (both Tachinusini).” with “Meanwhile, they are completely absent in the rest of Tachyporinae, except for *Nitidotachinus* (Tachinusini), *Leucotachinus* (Tachinusini), *Tachinoporus* (Vatesini), and *Vatesus berghoffae* (Vatesini).”.

The author apologizes for any inconvenience caused and states that the scientific conclusions are unaffected. The rest of the manuscript does not need to be changed. The original article has been updated.
