# Supplementary material for: Tachyporinae Revisited: Phylogeny, Evolution, and Higher Classification Based on Morphology, with Recognition of a New Rove Beetle Subfamily (Coleoptera: Staphylinidae)
Source: Biology (Basel). 2021 Apr 13;10(4):323. doi: 10.3390/biology10040323 (PMC8069000; doi:10.3390/biology10040323)
Supplement: Supplementary file 1 [file biology-10-00323-s001.zip › Supplementary_materials/FiguresS1-3.pdf]

Figures S1–3. Supplementary figures in PDF format with figure captions (.pdf).

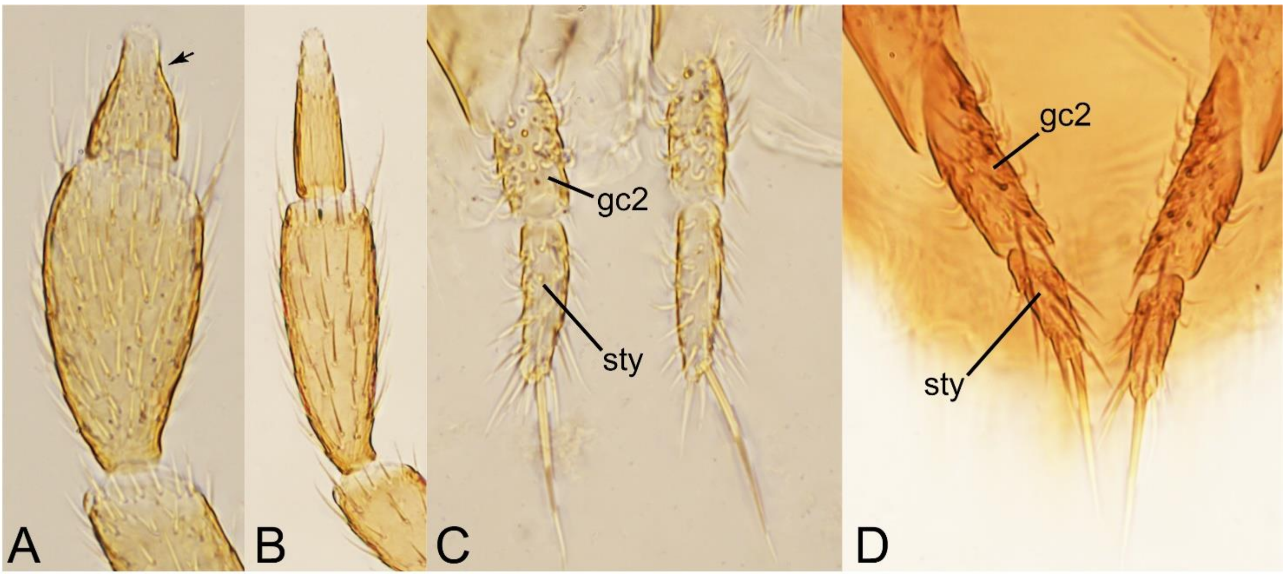

Figure S1. Photographs of body parts of Tachyporini, enlarged: the subtribe Tachyporina. A, left maxillary palpus of *Palporus nitidulus*, dorsal view. B, left maxillary palpus of *Tachyporus obtusus*, dorsal view. C, gonocoxite 2 and gonostylus of *P. nitidulus*, dorsal view. D, gonocoxite 2 and gonostylus of *T. obtusus*, dorsal view. Abbreviations: gc2, gonocoxite 2; sty, gonostylus. Arrow showing the abruptly narrowed maxillary palpomere 4 in apical third.

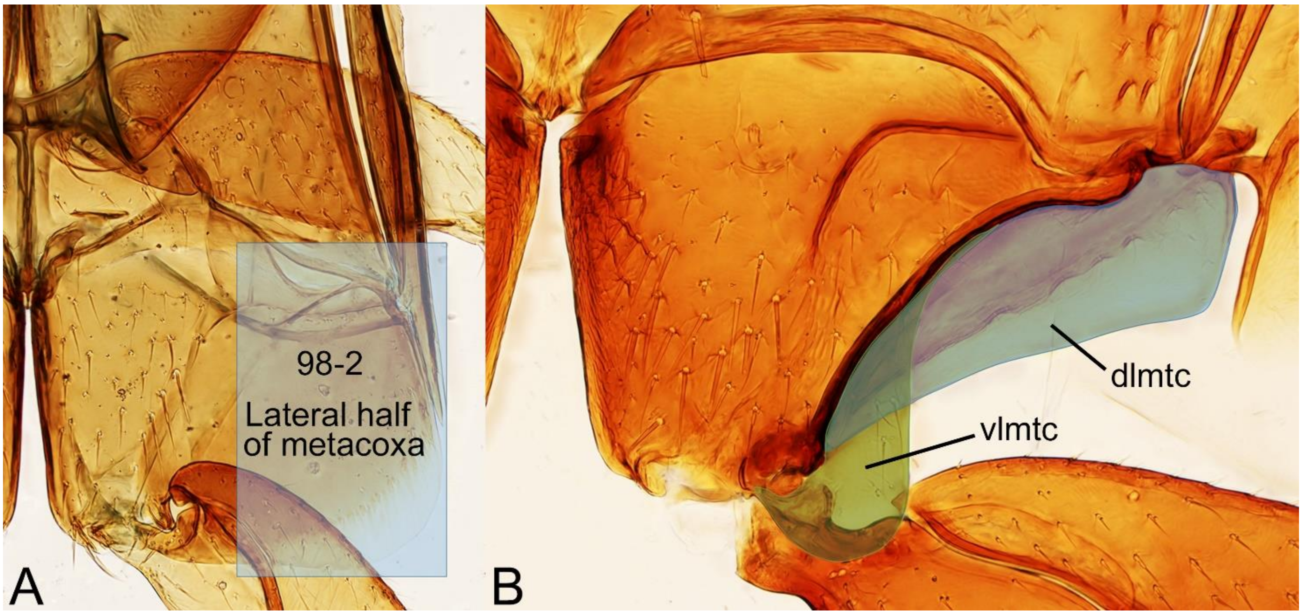

Figure S2. Photographs of metacoxae of Tachyporinae and Mycetoporinae. A, left metacoxa of *Bryophacis smetanai*, ventral view, highlighting large lateral half of metacoxa (used in character 98). B, left metacoxa of *Leucotachinus luteonitens*, ventral view, showing both dorsal and ventral lamellae. Abbreviations: dlmtc, dorsal lamella of metacoxa; vlmtc, ventral lamella of metacoxa. Characters and character states (format X-X) are indicated on each figure.

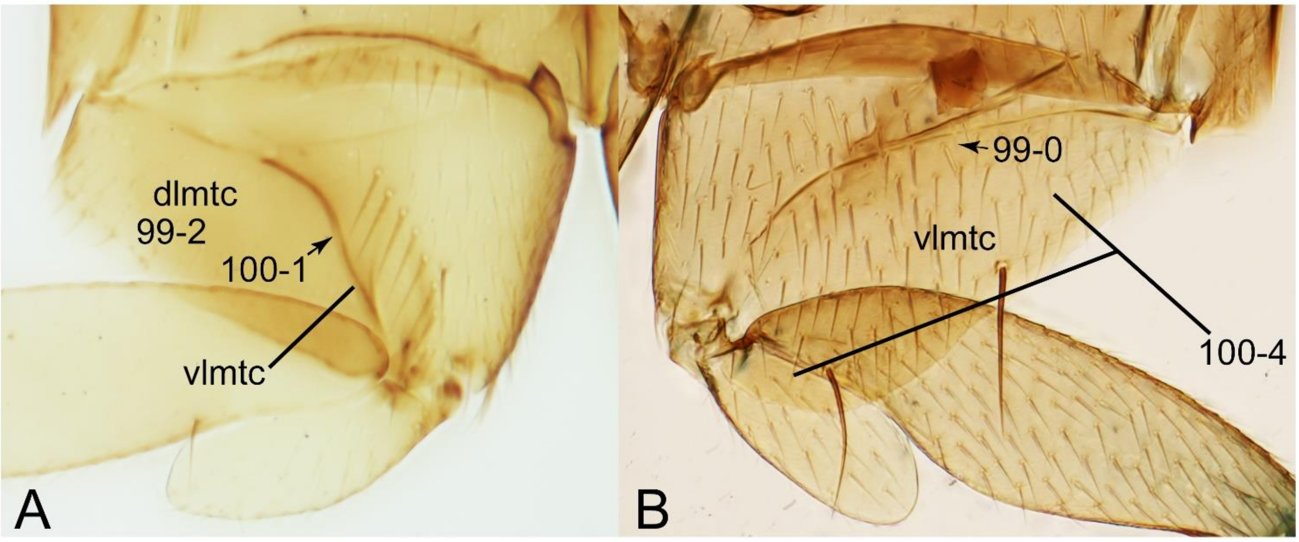

Figure S3. Photographs of metacoxae of Trichophyinae and Habrocerinae (out-groups). A, right metacoxa of *Trichophya tarsalis* (Casey) (Trichophyinae), ventral view, showing weakly developed ventral lamella (*vlmtc*) restricted to along mesial margin of metacoxa (used in character 100). B, left metacoxa of *Habrocerus capillaricornis* (Gravenhorst) (Habrocerinae), ventral view, showing very widely expanded, markedly large ventral lamella (*vlmtc*) (used in character 100) and completely reduced dorsal lamella (*dlmtc*) of metacoxa (used in character 99). Abbreviations: dlmtc, dorsal lamella of metacoxa; vlmtc, ventral lamella of metacoxa. Characters and character states (format X-X) are indicated on each figure.
