# Supplementary material for: Tachyporinae Revisited: Phylogeny, Evolution, and Higher Classification Based on Morphology, with Recognition of a New Rove Beetle Subfamily (Coleoptera: Staphylinidae)
Source: Biology (Basel). 2021 Apr 13;10(4):323. doi: 10.3390/biology10040323 (PMC8069000; doi:10.3390/biology10040323)
Supplement: Supplementary file 1 [file biology-10-00323-s001.zip › Supplementary_materials/FileS1.docx]

**Appendix S1. List of examined material and literature in Word format (.docs).**

The examined taxa were listed under the traditional classification. Type genus of a tribe or subfamily (*); type species of a genus (§). Almost all the taxa were used for character coding, but those of the two genera (i.e., *Tachinoporus*, *Tachinoproporus*) were based on observations only. Their original data are removed here, but alternatively included in the main article. The “glass card” refers to the style mentioned in Maruyama (2004).

A total of four different sources were used for the extraction of the morphological information in this study. They were classified into the four types as follows:

Data (*P*): Dry, pinned specimens.

Data (*S*): Slide specimens, mounted on a glass slide.

Data (*W*): Wet specimens, preserved in 70~80% EtOH (ethyl alcohol) solution.

Data (*R*): References, extracted from the literature.

**Abbreviations:**

A [black]: adult.

A, B, or C [red, top of slide]: larval types of *Sepedophilus* with which these adults were associated (see Newton, 1984).

CB: Canada balsam mounting medium [usually an error for Kleermount®, a synthetic histological mounting medium].

PM: Permount®, a synthetic histological mounting medium.

R: right.

W: wing.

**Institutions:**

BMNH: The Natural History Museum, London (M. F. Geiser).

FMNH: The Field Museum of Natural History, Chicago (J. Snyder).

FMNH, cAN: The Field Museum of Natural History, Chicago (private collection of A. F. Newton).

FMNH, cMT: The Field Museum of Natural History, Chicago (private collection of M. K. Thayer).

MHNG: Muséum d'Histoire Naturelle, Genève (G. Cuccodoro).

NHMW: Naturhistorisches Museum Wien (H. Schillhammer).

NSMT, cSN: National Museum of Nature and Science, Tsukuba, Japan (private collection of S. Nomura).

**Ingroup:**

*Tachyporine Group of subfamilies*

**Subfamily Tachyporinae MacLeay, 1825**

**Tribe Deropini Smetana, 1983**

Genus *Derops* Sharp, 1889*

*Derops divalis* (Sanderson, 1947)

*Present classification*. Tachyporinae; Deropini; *Derops*.

*Data type*. P + S + R.

*Data (P)*. 1 male: “Winslow, Ark. | June 8, 1946 | M.W. Sanderson” < handwritten in rectangular small white label >, “Devils Den ST. | PK. Debris | in crevices” < handwritten in rectangular small white label >, “PARATYPE | Rimulincola [handwritten] | divalis [handwritten] | Sanderson [handwritten] | ♂ [handwritten]” < printed in rectangular small blue label, not dissected > (FMNH); 1 female: “Winslow, Ark. | Mar. 30, 1941 | M.W. Sanderson” < handwritten in rectangular small white label >, “Devil’s Den | State Park | Debris in crevice” < handwritten in rectangular small white label >, “PARATYPE | Rimulincola [handwritten] | divalis [handwritten] | Sanderson [handwritten] | ♀ [handwritten]” < printed in rectangular small blue label, not dissected > (FMNH); 1 male: “III. Union Co. | 1 mi E Wolf | Lake 8.V.76 | A. Smetana” < printed in rectangular small white label >, “A. Newton | Collection” < printed in rectangular small green label >, “Rimulincola [handwritten] | divalis Sand. [handwritten] | A. Newton det. 1978” < printed in rectangular small blue label, not dissected > (FMNH).

*Data (S)*. 1 female: “♀ A | Derops | ~~Rimulincola~~ | divalis Sand. | ARK.: Wash. Co. | Devil’s Den St. | Pk, July 13, | 1949 deep crev- | ices, damp deb. [debris] |Sand. + Stan. | CB” < directly handwritten on the slide > (FMNH, cAN).

*Data (R)*. Sanderson (1947).

*Derops longicornis* Sharp, 1889^§^

*Present classification*. Tachyporinae; Deropini; *Derops*.

*Data type*. P + S + R.

*Data (P)*. 1 male, 1 female: “Kikuchi-valley. | Kumamoto-pref. | 11. iv. 1981 | S. Naomi leg.” < printed in rectangular small white label >, “*Derops longicornis* | Sharp, 1889 | det. S. Yamamoto, 2019” < printed in rectangular small white label, not dissected > (FMNH).

*Data (S)*. 1 male, 1 female: “Kikuchi-valley. | Kumamoto-pref. | 11. iv. 1981 | S. Naomi leg.” < printed in rectangular small white label, directly pasted on the slide >, “*Derops longicornis* | Sharp, 1989 | Det. S. Yamamoto, 2017” < printed in rectangular small white label, directly pasted on the slide > (FMNH).

*Data (R)*. Naomi (1986); Watanabe (1985).

*Derops yaeyamanus* Kishimoto, 2001

*Present classification*. Tachyporinae; Deropini; *Derops*.

*Data type*. P + S + R.

*Data (P)*. 1 male, 2 females: “JAPAN: RYÛKYÛ, | Kampire fall, Taketomi-chô, | Okinawa-ken, 19. IV. 2014, | S. Yamamoto leg.” < printed in rectangular small white label >, “*Derops yaeyamanus* | Kishimoto, 2001 | det. S. Yamamoto, 2014” < printed in rectangular small green label, not dissected > (KUM).

*Data (S)*. 1 male, 1 female: “Kikuchi-valley. | Kumamoto-pref. | 11. Iv. 1981 | S. Naomi leg.” < printed in rectangular small white label, directly pasted on the slide >, “*Derops yaeyamanus* | Kishimoto, 2001 | det. S. Yamamoto, 2014” < printed in rectangular small green label, directly pasted on the slide > (FMNH).

*Data (P)*. Kishimoto (2001).

**Tribe Megarthropsini Cameron, 1919**

Genus *Lacvietina* Herman, 2004

*Lacvietina takashii* (Hayashi, 2003)

*Present classification*. Tachyporinae; Tachinusini; *Lacvietina*.

*Data type*. P + S + R.

*Data (P)*. 1 male, 1 female: “JAPAN: RYÛKYÛ, | Okinawa-ken, Ishigaki-shi, | Mt. Omoto-dake, | 31.iii.2014, S. Yamamoto” < printed in rectangular small white label, not dissected > (KUM).

*Data (S)*. 2 males: “JAPAN: RYÛKYÛ, | Okinawa-ken, Ishigaki-shi, | Mt. Omoto-dake, | 31.iii.2014, S. Yamamoto” < printed in rectangular small white label, directly pasted on the slide >, “*Lacvietina takashii* | (Hayashi, 2003) | det. Yamamoto, 2014” < printed in rectangular small green label, directly pasted on the slide > (FMNH); 2 females: “JAPAN: RYÛKYÛ, | Mt. Omoto-dake, Ishigaki-shi, | Okinawa-ken, 15. IV. 2014, | S. Yamamoto leg., shifting” < printed in rectangular small white label, directly pasted on the slide >, “*Lacvietina takashii* | (Hayashi, 2003) | det. S. Yamamoto, 2017” < printed in rectangular small green label, directly pasted on the slide > (FMNH).

*Data (R)*. Hayashi (2003); Herman (2004).

Genus *Megarthropsis* Cameron, 1919*

*Megarthropsis decorata* Cameron, 1919^§^

*Present classification*. Tachyporinae; Tachinusini; *Megarthropsis*.

*Data type*. P + S + R.

*Data (P)*. 1 male, 1 female: “BORNEO SABAH Mt. | Kinabalu N.P. Sum- | mit Trail 1890m | A. Smetana” < printed in rectangular small white label >, “FIELD MUSEUM | ex collection of | H. G. Nelson” < printed in rectangular small white label >, with additional identification label for the male specimen: “Megarthropsis [handwritten] | decorata [handwritten] | det. A. Newton 20~~15~~ 01 [01, handwritten]” < printed in rectangular small white label, not dissected > (FMNH).

*Data (S)*. 1 male, 1 female: “♂♀ A | Megarthropsis | decorata | Cameron | BORNEO: Sabah: | Mt. Kinabalu | N. P., Summit | Trail 1890m | A. Smetana | PM” < directly handwritten on the slide >, < with additional green round seal on the right side: “3.01 [handwritten; it means that the slide was made by Al. Newton on March 2001.] >” (FMNH, cAN).

*Data (R)*. Herman (2004).

Genus *Nepaliodes* Coiffait, 1977

*Nepaliodes solangelae* Herman, 2004

*Present classification*. Tachyporinae; Tachinusini; *Nepaliodes*.

*Data type*. P + R.

*Data (P)*. 2 females: “Dam B’Ri (by Tullgrenf.) | (ca. 800m), Lam Dong. P. | [S-VIETNAM] | 30. v. 2002, S.Nomura leg.” < printed in rectangular small white label, not dissected > (NSMT, cSN); 1 female: “Dam B’Ri (ca. 800m) | LamDong. Prov. | [S-VIETNAM] | 1. vi. 2002, S.Nomura leg.” < printed in rectangular small white label, not dissected > (NSMT, cSN); 1 female: “Dam B’Ri (ca. 800m) | Lam Dong Prov. | [S-VIETNAM] | 2. v., 2003, S.Nomura leg.” < printed in rectangular small label >, < with an additional glass card mounted on the abdominal terminalia > (NSMT, cSN).

*Data (R)*. Herman (2004).

Genus *Peitawopsis* Smetana, 1992

*Peitawopsis monticola* Smetana, 1992^§^

*Present classification*. Tachyporinae; Tachinusini; *Peitawopsis*.

*Data type*. P + R.

*Data (P)*. 1 male: “TAIWAN Pingtung Hsien | Pietawushan, Kuai-Ku | Hut 2325m 22.V.1991 | A. Smetana [T90]” < printed in rectangular small white label >, “PARATYPE | Peitawopsis [handwritten] | monticola [handwritten] | A. Smetana 1991 [1991, handwritten]” < printed in rectangular small yellow label, not dissected > (FMNH); 1 male: “TAIWAN, Pingtung | Hsien, Peitawushen | Kuai-Ku Hut 2135m | 30.IV.1992 | A. Smetana [T108]” < printed in rectangular small white label >, “Peitawopsis [handwritten] | monticola Smet. [handwritten] | det. A. Smetana 1993 [93, handwritten]” < printed in rectangular small white label, not dissected > (FMNH).

*Data (R)*. Herman (2004).

**Tribe Mycetoporini Thomson, 1859**

Genus *Bolitobius* Leach, 1819

Subgenus *Bolitobius* Leach, 1819

*Bolitobius castaneus castaneus* (Stephens, 1832)^§^

*Present classification*. Mycetoporinae; *Bolitobius*.

*Data type*. P + S + R.

*Data (P)*. 1 female: “Topla | Paganetti” < printed in rectangular small white label >, “analis Payk [handwritten] | det. Bernh.” < printed in rectangular small white label >, “Bolitobius [handwritten] | castaneus (STEPH) [handwritten] | det. M.Schülke 19 92 [92, handwritten]” < printed in rectangular small white label >, “Chicago NHMus | M.Bernhauer | Collection” < printed in rectangular small white label, < with an additional glass card mounted on the abdominal terminalia > (FMNH); 1 male: “Kameno | Paganetti” < printed in rectangular small white label >, “analis Ndm [handwritten] | det. Bernh.” < printed in rectangular small white label >, “Chicago NHMus | M. Bernhauer | Collection”, “Bolitobius [handwritten] | castaneus (STEPH) [handwritten] | det. M.Schülke 19 92 [92, handwritten]” < printed in rectangular small white label, not dissected > (FMNH).

*Data (S)*. 1 male: “Lausitz | Finsterwld.” < printed in rectangular small white label, directly pasted on the slide >, “Bryocharis | analis Payk” < handwritten in rectangular small white label, directly pasted on the slide >, “♂”< handwritten in rectangular small white label, directly pasted on the slide >, “CNHM 1955 | Eduard Knirsch | Palaearctic Colln.” < printed in rectangular small white label, directly pasted on the slide >, “Bolitobius [handwritten] | castaneus (STEPH) [handwritten] | det. M.Schülke 19 92 [92, handwritten]” < printed in rectangular small white label, directly pasted on the slide >, “*Bolitobius* | (*Bolitobius*) | castaneus castaneus | (Stephens, 1832) | Tachyporinae: | Mycetoporini” < printed in rectangular small white label, directly pasted on the slide > (FMNH).

*Data (R)*. Schülke (2010).

Genus *Bolitopunctus* Campbell, 1993

*Bolitopunctus* sp.

*Present classification*. Mycetoporinae; *Bolitopunctus*.

*Data type*. P.

*Data (P)*. 1 male: “USA: CA: Riverside Co., | San Jacinto Mts., Lake | Fulmor, ca. 15 km NW | Idyllwild, 33°48.56'N, | 116°46.3'W, 1530 m;” < printed in rectangular small white label >, “oak grove in pine forest; | 26.III.1999, litter in swale | at base of boulders | S. & J. Peck 99-112 | FIELD MUS. NAT. HIST.” < printed in rectangular small white label >, “Bolitopunctus [handwritten] | n. sp. [handwritten] | det. Newton 2001” < printed in rectangular small white label, not dissected > (FMNH).

Genus *Bryophacis* Reitter, 1909

*Bryophacis smetanai* Campbell, 1993

*Present classification*. Mycetoporinae; *Bryophacis*.

*Data type*. P + S + R.

*Data (P)*. 1 male: “Arizona. | Graham Mts. | May 18 1969 | K. Stephan leg.” < printed in rectangular small white label >, “Field Mus. Nat. Hist. | Karl H. Stephan | Collection, 1977 | Acc. No. Z-15, 639” < printed in rectangular small white label >, “PARATYPE | Bryophacis | smetanai | J.M.Campbell 1991” < printed in rectangular small yellow label, not dissected > (FMNH).

*Data (S)*. 1 male, 1 female: “♂♀ A | Bryophacis | smetanai Champ. | (PM ←)” < directly handwritten on the slide >, “N.H.: Coos Co., 0.7mi | s Jefferson Notch | at Jefferson Brook, | 880m, 17.ix.1983 | mixed conif.-hdwd. | A. Newton, M. Thayer” < printed in rectangular small white label, directly pasted on the slide >, “berl. forest | leaf & log | litter” < printed in rectangular small white label, directly pasted on the slide >, < with additional comment: “PM” [directly handwritten on the slide] > (FMNH, cAN).

*Data (R)*. Campbell (1993a).

Genus *Bryoporus* Kraatz, 1857

*Bryoporus* cf. *orientalis* Cameron, 1937

*Present classification*. Mycetoporinae; *Bryoporus*.

*Data type*. P + S.

*Data (P)*. 2 males, 1 female: “INDONESIA: Bali Is., | Gerokgak, | Bendungan Gerokgak, | 24-27 III 2005, | SUZUKI W. (FIT)” < printed in rectangular small white label >, “*Bryoporus* cf. *orientalis* | Cameron, 1937 | det. S. Yamamoto, 2019” < printed in rectangular small white label, not dissected > (KUM); 1 male, “INDONESIA: Bali Is., | Gerokgak, | Bendungan Gerokgak, | 28-29 III 2005, | SUZUKI W. (FIT)” < printed in rectangular small white label >, “*Bryoporus* cf. *orientalis* | Cameron, 1937 | det. S. Yamamoto, 2019” < printed in rectangular small white label, not dissected > (FMNH).

*Data (S)*. 1 male: “INDONESIA: Bali Is., | Gerokgak, | Bendungan Gerokgak, | 24-27 III 2005, | SUZUKI W. (FIT)” < printed in rectangular small white label, directly pasted on the slide >, “*Bryoporus* cf. *orientalis* | Cameron | det. M. Schülke, 2007” < printed in rectangular small green label, directly pasted on the slide > (FMNH); 1 female: “INDONESIA: Bali Is., | Gerokgak, | Bendungan Gerokgak, | 28-29 III 2005, | SUZUKI W. (FIT)” < printed in rectangular small white label, directly pasted on the slide >, “*Bryoporus* cf. *orientalis* | Cameron, 1937 | Det. M. Schülke, 2007” < printed in rectangular small white label, directly pasted on the slide > (FMNH).

*Bryoporus* *rufescens* LeConte, 1863

*Present classification*. Mycetoporinae; *Bryoporus*.

*Data type*. P + S + R.

*Data (P)*. 1 male: “NEW JERSEY: Morris | Co.: Newfoundland | leg. W. Suter. | 16:VIII:1979.” < printed in rectangular small white label >, “WS#79-29B. | Sphagnum | under tamarack.” < printed in rectangular small white label >, “Bryoporus | rufescens | LeConte | Det. J.M.Campbell” < printed in rectangular small white label, not dissected > (FMNH); 1 male: “TENN.: Sevier Co. | Gatlinburg | v.17-22.1972 3200’ [3200’, handwritten]” < printed in rectangular small white label >, “Grotto Falls | Trail” < handwritten in rectangular small white label >, “under bark | elm? [handwritten] | A. Newton” < printed in rectangular small white label >, “Bryoporus [handwritten] | rufescens LeC. [handwritten] | Det. J.M.Campbell” < printed in rectangular small white label, not dissected > (FMNH); 1 female: “KENTUCKY: Edmonson | Co.; Mammoth Cave Nat’l. | Pk. Cabin Woods. Leg: | W. Suter. 111:29:1974.” < printed in rectangular small white label >, “FM(HD)#74-57. | WS #74-24g. Ber.: | Subcortical & basal | stump” < printed in rectangular small white label >, “Bryoporus | rufescens | LeConte | Det. J.M.Campbell” < printed in rectangular small white label, not dissected > (FMNH).

*Data (S)*. 1 male, 1 female: “♂♀ A | Bryoporus | rufescens | TEX.: Bastrop Co. | Buescher St. Pk. | VI.15-16.1973 | berl., litter, oak- | pine forest | A. Newton | PM” < directly handwritten on the slide> (FMNH, cAN).

*Data (R)*. Campbell (1993a).

Genus *Canariobolitobius* Schülke, 2004

*Canariobolitobius* *filicornis* (Wollaston, 1864)

*Present classification*. Mycetoporinae; *Canariobolitobius*.

*Data type*. P + R.

*Data (P)*. 1 male: “Tenerife | Santa Ursula | 3. 2. 49 Lindberg” < printed in rectangular small white label >, “Mycetoporus | monilicornis | Woll.” < handwritten in rectangular small white label >, “ex coll. | Scheerpeltz” < handwritten in rectangular small blue label >, “Mycetoporus sp. [sp., handwritten] | nec monilicornis [handwritten] | Woll. [handwritten] | Det. J. M. Campbell” < printed in rectangular small white label >, “BRYOPHACIS | sp. 5 [handwritten] | M. Kocian det. 94 [94, handwritten]” < printed in rectangular small white label >, “ “Bryophacis” | filicornis (Wollaston) | det. M. Schülke 2002” < printed in rectangular small white label >, <with an additional plastic card mounted on the abdominal terminalia > (NHMW); 1 male: “Tenerife, Pico | del Teide | 2600-3000m” < printed in rectangular small white label >, “ “Bryophacis” | filicornis (Wollaston) | det. M. Schülke 2002” < printed in rectangular small white label, not dissected > (NHMW); 1 unsexed: “~~Mocan al~~ bei Valverde | Isla del Hierro | lg.H. Franz” < printed in rectangular small white label >, “ “Bryophacis” | filicornis (Wollaston) | det. M. Schülke 2002” < printed in rectangular small white label, not dissected > (NHMW); 1 unsexed: “El Hierro | La Dehesa” < printed in rectangular small white label >, “ “Bryophacis” | filicornis (Wollaston) | det. M. Schülke 2002” < printed in rectangular small white label, not dissected > (NHMW).

*Data (R)*. Schülke (2004).

Genus *Carphacis* Gozis, 1886

*Carphacis* *effrenatus* Herman, 2001

*Present classification*. Mycetoporinae; *Carphacis*.

*Data type*. P + S + R.

*Data (P)*. 1 male: “Ledyard, Ct. | 19. V. 1977 [handwritten] | Larry Watrous” < printed in rectangular small white label, not dissected > (FMNH); 1 female: “Smith Sta.,La | Porte Co.,IND. | 23:V:53 [handwritten]” < printed in rectangular small white label >, “under piece | of wood on freshly cut | maple stump” < handwritten in rectangular small white label >, “Carphacis | intrusus (Horn) | Det. J.M. | Campbell 1978” < handwritten in rectangular small white label, not dissected > (FMNH).

*Data (S)*. 1 male, 1 female: “♂♀ A | Carphacis | intrusus | (PM ←)” < directly handwritten on the slide >, “MASS.:Middlesex Co. | Lincoln |15. vi. 1982 [handwritten] | A. Newton, M. Thayer” < printed in rectangular small white label, directly pasted on the slide >, “on mushrooms: | Tricholomopsis | platyphylla” < printed in rectangular small white label, directly pasted on the slide >, < with additional comment: “PM” [directly handwritten on the slide] > (FMNH, cAN).

*Data (R)*. Campbell (1980, as *C. intrusus*); Herman (2001).

Genus *Ischnosoma* Stephens, 1829

*Ischnosoma* *splendidum* (Gravenhorst, 1806)^§^

*Present classification*. Mycetoporinae; *Ischnosoma*.

*Data type*. P + S + R.

*Data (P)*. 1 male: “Cro. Bernh. | Fuzine 97” < printed in rectangular small white label >, “splendidum G. [handwritten] | det. Bernh.” < printed in rectangular small white label >, “Chicago NHMus | M.Bernhauer | Collection” < printed in rectangular small white label >, <with an additional plastic card mounted on the abdominal terminalia > (FMNH); 1 female: “USA: S. Dak., Pen- | nington Co., N. Roch- | ford, Irish Gulch, | 26-V-1980,” < printed in rectangular small white label >, “FMHD #80-3149, wil- | low scrub litter at | creek, W. S. Suter” < printed in rectangular small white label >, “Ischnosoma | splendidum | (Gravenhorst) | Det. J.M.Campbell 1990” < printed in rectangular small white label, not dissected > (FMNH).

*Data (S)*. 1 female: “USA: S. Dak., Custer | Co., 16mi W Mud | Springs Rd. facili- | ty, 24-V-1980,” < printed in rectangular small white label, directly pasted on the slide >, “FMHD #80-3134, under | cedar & pine, W. S. | Suter” < printed in rectangular small white label, directly pasted on the slide >, “Ischnosoma | splendidum | (Gravenhorst) | Det. J.M.Campbell 1990” < printed in rectangular small white label, directly pasted on the slide > (FMNH); 1 female: “USA: S. Dak., Pen- | nington Co., N Roch- | ford, Irish Gulch, | 26-V-1980,” < printed in rectangular small white label, directly pasted on the slide >, “FMHD #80-3152, | litter on aspen log | on slope, W. S. | Suter” < printed in rectangular small white label, directly pasted on the slide > (FMNH).

*Data (R)*. Campbell (1991).

Genus *Lordithon* Thomson, 1859

*Lordithon* *lunulatus* (Linnaeus, 1760)

*Present classification*. Mycetoporinae; *Bobitobus*.

*Data type*. P + S + R.

*Data (P)*. 1 female, 1 unsexed: “SPAIN, Puerto de la | Bonaigua (hotel de los | Abletos). El Pallars,” < printed in rectangular small white label >, “Pireneos Orientales | 1400 m.20-28.VII 1973 | Borys Molkin” < printed in rectangular small white label >, “carcass bait | traps” < printed in rectangular small white label >, < with an additional glass card mounted on the abdominal terminalia, only for the female > (FMNH); 1 male: “Bernh.Bosn. | Trebovic.” < printed in rectangular small white label >, “Lunulatus L. [handwritten] | det. Bernh.” < printed in rectangular small white label >, “Chicago NHMus | M.Bernhauer | Collection” < printed in rectangular small white label > (FMNH).

*Data (S)*. 1 male: “SPAIN, Puerto de la | Bonaigua (hotel de los | Abletos). El Pallars,” < printed in rectangular small white label, directly pasted on the slide >, “Pireneos Orientales | 1400 m.20-28.VII 1973 | Borys Molkin” < printed in rectangular small white label, directly pasted on the slide >, “carcass bait | traps” < printed in rectangular small white label, directly pasted on the slide >, “*Lordithon* | *lunulatus* | (Linnaeus, 1761)” < printed in rectangular small white label, directly pasted on the slide > (FMNH).

*Data (R)*. Campbell (1982).

*Lordithon* *thoracicus thoracicus* (Fabricius, 1777)^§^

*Present classification*. Mycetoporinae; *Lordithon*.

*Data type*. P + S + R.

*Data (P)*. 3 males: “California: | Butte Co., | M & T Ranch | 5.v. 60” < handwritten in rectangular small white label >, “ex. shelf fungus” < handwritten in rectangular small white label >, “Coll. (leg.) [leg., handwritten] | D. H. Kistner | Field No.” < printed in rectangular small white label >, “Lordithon | thoracicus (Fab.) [handwritten] | det. J.M. Campbell | 1978” < printed in rectangular small white label >, < with an additional glass card mounted on the abdominal terminalia, only for the single male > (FMNH); 1 unsexed: “California: | Butte Co., | Chico, | 12-III-60 [handwritten]” < printed in rectangular small white label >, “gill fungus” < printed in rectangular small white label >, “Coll. | D. H. Kistner” < printed in rectangular small white label >, “Lordithon | thoracicus (Fab.) [handwritten] | det. J.M. Campbell | 1978” < printed in rectangular small white label, not dissected > (FMNH).

*Data (S)*. 2 females: “California: | Butte Co., | M & T Ranch | 5.v. 60” < handwritten in rectangular small white label, directly pasted on the slide >, “ex. shelf fungus” < handwritten in rectangular small white label, directly pasted on the slide >, “Coll. (leg.) [leg., handwritten] | D. H. Kistner | Field No.” < printed in rectangular small white label, directly pasted on the slide >, “Lordithon | thoracicus (Fab.) [handwritten] | det. J.M. Campbell | 1978” < printed in rectangular small white label, directly pasted on the slide > (FMNH).

*Data (R)*. Campbell (1982).

Genus *Mycetoporus* Mannerheim, 1830*

*Mycetoporus* *punctus* (Gravenhorst, 1806)^§^

*Present classification*. Mycetoporinae; *Mycetoporus*.

*Data type*. P + S.

*Data (P)*. 1 male: “BADGASTEIN | GRAVENEGG” < printed in rectangular small white label >, “Chicago NHMus | M. Bernhauer | Collection” < printed in rectangular small white label >, “Mycetoporus | punctus (Gravenhorst) | det. M. Schülke 2004” < printed in rectangular small white label >, < with an additional glass card mounted on the abdominal terminalia > (FMNH); 1 female: “HOHE TAUERN | KOETSCHACHTAL | BERNHAUER” < printed in rectangular small white label >, “Chicago NHMus | M. Bernhauer | Collection” < printed in rectangular small white label >, “Mycetoporus | punctus (Gravenhorst) | det. M. Schülke 2004” < printed in rectangular small white label, not dissected > (FMNH); 2 unsexed: “TI. OETZTAL | OB.-GURGL | VII. 36. BERNH” < printed in rectangular small white label >, “Chicago NHMus | M. Bernhauer | Collection” < printed in rectangular small white label >, “Mycetoporus | punctus (Gravenhorst) | det. M. Schülke 2004” < printed in rectangular small white label, not dissected > (FMNH).

*Data (S)*. 1 male: “HOHE TAUERN | ANLAUFTAL | BERNHAUER” < printed in rectangular small white label >, “Chicago NHMus | M. Bernhauer | Collection” < printed in rectangular small white label >, “Mycetoporus | punctus (Gravenhorst) | det. M. Schülke 2004” < printed in rectangular small white label > (FMNH).

Genus *Neobolitobius* Campbell, 1993

*Neobolitobius* *varians* (Hatch, 1957)^§^

*Present classification*. Mycetoporinae; *Neobolitobius*.

*Data type*. P + S + R.

*Data (P)*. 1 male [both of the antennae originally lost]: “Corvallis, Ore. | I-24-35 | Coll. J. Schuh” < printed in rectangular small white label >, “C.N.H.M. 1960 | Borys Malkin | Coleoptera Colln.” < printed in rectangular small white label >, “Neobolitobius | varians | (Hatch) | Det. J.M.Campbell” < printed in rectangular small white label, not dissected > (FMNH); 1 female: “Eugene Ore | 7. III. 42” < handwritten in rectangular small white label >, “C. N. H. M. 1960 | Borys Malkin | Coleoptera Colln.” < printed in rectangular small white label >, “Neobolitobius | varians | (Hatch) | Det. J.M.Campbell” < printed in rectangular small white label >, < with an additional glass card mounted on the abdominal terminalia > (FMNH).

*Data (S)*. 1 female: “McMinnvilleOre | 2 15 1941 [each separated by ‘|’; handwritten, except “19”] | KM & DM Fender” < printed in rectangular small white label, directly pasted on the slide >, “C.N.H.M. 1960 | Borys Malkin | Coleoptera Colln.” < printed in rectangular small white label, directly pasted on the slide >, “Neobolitobius | varians | (Hatch) | Det. J.M.Campbell” < printed in rectangular small white label, directly pasted on the slide > (FMNH).

*Data (R)*. Campbell (1993a).

Genus *Parabolitobius* L.-Z. Li, M.-J. Zhao & Sakai, 2000

*Parabolitobius* *formosus* (Gravenhorst, 1806)

*Present classification*. Mycetoporinae; *Parabolitobius*.

*Data type*. P + S.

*Data (P)*. 1 male: “Cro. Bernh. | Capella 97” < printed in rectangular small white label >, “Formosa Gr. [handwritten] | det. Bernh.” < printed in rectangular small white label >, “Chicago NHMus | M.Bernhauer | Collection” < printed in rectangular small white label >, “Bolitobius [handwritten] | formosus (Gr) [handwritten] | det. M. Schülke 19 92 [92, handwritten]” < printed in rectangular small white label, not dissected > (FMNH); 1 female: “1 | 97” < printed in rectangular small white label >, “Olomouc | Tschechoslow. [Czechoslovakia]” < handwritten in rectangular small white label >, “*Br*. | *formosa* | *Grav*.” < handwritten in rectangular small white label, folded in three times >, “Field Mus. Nat. Hist. | 1966 | A. Bierig Colln. | Acc. Z-13812” < printed in rectangular small white label >, “Bolitobius [handwritten] | formosus (Gr) [handwritten] | det. M. Schülke 19 92 [92, handwritten]” < printed in rectangular small white label >, < with an additional glass card mounted on the abdominal terminalia > (FMNH).

*Data (S)*. 1 male: “Karakau | Natterer” < printed in rectangular small white label, directly pasted on the slide >, “formosa Gr [handwritten] | det. Bernh.” < printed in rectangular small white label, directly pasted on the slide >, “Chicago NHMus | M.Bernhauer | Collection” < printed in rectangular small white label, directly pasted on the slide >, “Bolitobius [handwritten] | formosus (Gr.) [handwritten] | det. M. Schülke 19 92 [92, handwritten]” < printed in rectangular small white label, directly pasted on the slide >, “*Parabolitobius* | *formosus* | (Gravenhorst, 1806) | Tachyporinae: | Mycetoporini” < printed in rectangular small white label, directly pasted on the slide > (FMNH).

**Tribe Tachyporini MacLeay, 1825**

Genus *Austrotachinus* Steel, 1956

*Austrotachinus* *fuscipes* Steel, 1956^§^

*Present classification*. Tachyporinae; Tachinusini; *Austrotachinus*.

*Data type*. P + R + W.

*Data (P)*. 1 female: “AUSTRALIA: Queensland: | Windsor Tableland. 42 km | from highway, 16°15'13"S, | 145°02'16"E, 10.xi-26.xii.” < printed in rectangular small white label >, “1983, MDPI FIT site 14a, | Storey & Watford-Higgins | FIELD MUS. NAT. HIST.” < printed in rectangular small white label >, “Austrotachinus [handwritten] | n. sp. 3 [handwritten] | det. A. Newton 2015” < printed in rectangular small white label, not dissected > (FMNH); 1 female: “AUSTRALIA: New South | Wales: South East Forest | N.P., Wog Way, 2.0 km | from Coolangubra Forest | Way, 806m, 37°00.038'S,” < printed in rectangular small white label >,“149°3.772'E, 23.xi-1.xii. | 2013, moist *Eucalyptus* | forest *wl* tree ferns & dense | ground ferns; FMHD# | 2013-70 (ANIC Bulk#” < printed in rectangular small white label >, “4090), flight intercept trap, | A. Newton, M. Thayer, A. | Seago; ANMT site 1252 | FIELD MUS. NAT. HIST.” < printed in rectangular small white label >, “Austrotachinus [handwritten] | M.K.Thayer det.2014” < printed in rectangular small white label, not dissected > (FMNH).

*Data (W)*. 1 female: “Mts. above (SW) | Atherton, Q. | Feb.7,’58 3-4000’ [Feb.7,’58, handwritten] | Darlingtons” < printed in rectangular small white label >, “Austrotachinus [handwritten] | fuscipes Steel [handwritten] | A.Newton det. 1977” < printed in rectangular small white label >, with additional comments on the back of this label: “ex series [i.e., the specimen is from a series of specimens at MCZ] | MCZ (♂, ♀)” < handwritten >, “wing folding staph. | “A”, LL [“A” here is Al. Newton’s informal term for typical staphylinid wing folding pattern (highly asymmetrical, compact, left + right wings broadly overlapping), and “LL” likely means “left long”, referring to which wing has the longest second fold. (the other type is “B”, where the wings are folded symmetrically and with only partial overlap, as in Scaphidiinae)]” < handwritten in rectangular small white label, fully dissected and disarticulated > (FMNH, cAN).

*Data (R)*. Steel (1956).

Genus *Cilea* Jacquelin du Val, 1856

*Cilea* *limbifera* (Motschulsky, 1858)

*Present classification*. Tachyporinae; Vatesini; *Cilea*.

*Data type*. P + S.

*Data (P)*. 2 males, 2 females: “JAPAN: RYÛKYÛ, | Akaishi, Ishigaki-shi, | Okinawa-ken, 15. IV. 2014, | S. Yamamoto (cattle dung)” < printed in rectangular small white label >, “*Cilea limbifera* | (Motschulsky, 1858) | det. S. Yamamoto, 2014” < printed in rectangular small green label, not dissected > (FMNH).

*Data (S)*. 1 male, 2 females: “JAPAN: RYÛKYÛ, | Akaishi, Ishigaki-shi, | Okinawa-ken, 15. IV. 2014, | S. Yamamoto (cattle dung)” < printed in rectangular small white label, directly pasted on the slide >, “*Cilea limbifera* | (Motschulsky, 1858) | det. S. Yamamoto, 2014” < printed in rectangular small green label, directly pasted on the slide > (FMNH).

*Cilea* *silphoides* (Linnaeus, 1767)^§^

*Present classification*. Tachyporinae; Vatesini; *Cilea*.

*Data type*. P + S + R.

*Data (P)*. 1 male, 1 female: “N.-OE. HORN | BERNHAUER | KOMPOST” < printed in rectangular small white label >, “Chicago NHMus | M.Bernhauer | Collection” < printed in rectangular small white label >, with additional identification label for the male specimen: “Cilea [handwritten] | silphoides [handwritten] | J. M. Campbell [handwritten] | det. ~~A. Newton 2015~~” < printed in rectangular small white label, not dissected > (FMNH).

*Data (S)*. 1 male: “N.-OE. HORN | BERNHAUER | KOMPOST” < printed in rectangular small white label, directly pasted on the slide >, “Chicago NHMus | M.Bernhauer | Collection” < printed in rectangular small white label, directly pasted on the slide >, “*Cilea silphoides* | (Linnaeus, 1767) | Tachyporinae: | Tachyporini” < printed in rectangular small white label, directly pasted on the slide > (FMNH); 1 female: “♀ A | Cilea | silphoides | (L.) | Silesia | PM” < directly handwritten on the slide >, < with additional green round seal on the right side: “3.88 [handwritten; it means that the slide was made by Al. Newton on March 1988.] >” (FMNH, cAN).

*Data (R)*. Campbell (1975).

Genus *Cileoporus* Campbell, 1994

*Cileoporus* *fenestratus* (Sharp, 1883)

*Present classification*. Tachyporinae; Vatesini; *Cileoporus*.

*Data type*. P + R.

*Data (P)*. 1 male: “PANAMA: Canal Zone | Barro Colorado Is. | February 16,22 1976 | A. Newton” < printed in rectangular small white label >, “under bark, | fermenting” < printed in rectangular small white label >, “Cileoporus [handwritten] | fenestratus [handwritten] | det. A.Newton 1993 ~~2015~~ [1993, handwritten]” < printed in rectangular small white label, not dissected > (FMNH); 2 males: “PANAMA: Canal Zone | Barro Colorado Is. | February 8,25 1976 | A. Newton” < printed in rectangular small white label >, “under bark | 6 mos. dead” < printed in rectangular small white label >, “Cileoporus [handwritten] | fenestratus [handwritten] | (Sharp) [handwritten] | det. Newton 1993” < printed in rectangular small white label, not dissected > (FMNH); 1 male [ventral view fully visible, dorsally glued to the paper card]: “COSTA RICA | F NEVERMANN | 31 X 25 [handwritten]” < printed in rectangular small green label >, with additional comments on the back of this label: “HAMBURGFARM | REVENTAZON | EBENE LIMON” < printed >, “unter loser | Rinde” < printed in rectangular small white label >, “Tachinus | princeps Brnh | n. sp.” < handwritten in rectangular small white label >, “Chicago NHMus | M.Bernhauer | Collection” < printed in rectangular small white label >, “FMNH” < printed in rectangular small white label >, “Coproporus [handwritten] | Det. sp. [sp., handwritten] | J.M.Campbell 19 80 [80, handwritten]” < printed in rectangular small white label >, “Cileoporus [handwritten] | fenestratus [handwritten] | (Sharp) [handwritten] | Det. J.M. Campbell” < printed in rectangular small white label >, <with an additional plastic card mounted on the abdominal terminalia > (FMNH).

*Data (R)*. Campbell (1994a).

Genus *Coproporus* Kraatz, 1857

*Coproporus* *colchicus* Kraatz, 1858^§^

*Present classification*. Tachyporinae; Vatesini; *Coproporus*.

*Data type*. P + S.

*Data (P)*. 1 male, 1 female: “Krim | Baidar Thor ["Baidar Thor", = Baidarisches Tor] | DR. KNIRSOH” < printed in rectangular small white label >, “Coproporus | colchicus | Kr.” < handwritten in rectangular small white label >, “CNHM 1955 | Eduard Knirsch | Palaearctic Colln.” < printed in rectangular small white label, not dissected > (FMNH).

*Data (S)*. 1 male, 1 female: “Krim | Baidar Thor ["Baidar Thor", = Baidarisches Tor] | DR. KNIRSOH” < printed in rectangular small white label, directly pasted on the slide >, “Coproporus | colchicus | Kr.” < handwritten in rectangular small white label, directly pasted on the slide >, “CNHM 1955 | Eduard Knirsch | Palaearctic Colln.” < printed in rectangular small white label, directly pasted on the slide >, “Coproporus [handwritten] | colchicus [handwritten] | E. Knirsch [handwritten] | det. ~~A.Newton 2015~~” < printed in rectangular small white label, directly pasted on the slide >, “*Coproporus* | *colchicus* | Kraatz, 1858” < printed in rectangular small white label, directly pasted on the slide > (FMNH).

*Coproporus* *laevis* LeConte, 1863

*Present classification*. Tachyporinae; Vatesini; *Coproporus*.

*Data type*. P + S + R.

*Data (P)*. 1 male: “FLA.: Highlands Co., | Archbold Biol. Sta. | 12-19:VI:1955 at light. | leg. H. S. Dybas.” < printed in rectangular small white label >, “*Coproporus* | *laevis* | Leconte, 1863” < printed in rectangular small white label, not dissected > (FMNH); 1 female: “Fla.: Sarasota Co | Myakka Riv.St.Pk. | 28-VII-1976 uv lt | trap C.W. O’Brien” < printed in rectangular small white label >, “*Coproporus* | *laevis* | Leconte, 1863” < printed in rectangular small white label, not dissected > (FMNH).

*Data (S)*. 1 male, 1 female: “Fla.: Sarasota Co | Myakka Riv.St.Pk. | 28-VII-1976 uv lt | trap C.W. O’Brien” < printed in rectangular small white label, directly pasted on the slide >, “*Coproporus* | *laevis* | Leconte, 1863 | Det. J.M. Campbell, 1981” < printed in rectangular small white label, directly pasted on the slide > (FMNH).

*Data (R)*. Campbell (1975).

*Coproporus* *rutilus* (Erichson, 1839)

*Present classification*. Tachyporinae; Vatesini; *Coproporus*.

*Data type*. P + S + R.

*Data (P)*. 1 male: “Harrison Co.MS | 3.5 mi NE Saucier | 14 - V [handwritten] 198 4 [4, handwritten] | Paul K. Lago” < printed in rectangular small white label, not dissected > (FMNH); 1 female: “Texas: S. Patricio | Co., Welder Wild- | life refuge 23.v. | 74 CW LB O’Brien | GBMarshall uv lt” < printed in rectangular small white label, not dissected > (FMNH).

*Data (S)*. 1 male: “LOUISIANA: Plaque- | mines Parish; Tide H.O. | 10:XI:1976. Leg: W. Suter. | WS#76-79: a, b.” < printed in rectangular small white label, directly pasted on the slide >, “*Coproporus* | *rutilus* | Erichson, 1839” < printed in rectangular small white label, directly pasted on the slide > (FMNH); 1 female: “Jackson Co. MS | Ocean Springs | 13-V [handwritten] 1984 [84, handwritten] | Paul K. Lago” < printed in rectangular small white label, directly pasted on the slide >, “*Coproporus* | *rutilus* | Erichson, 1839” < printed in rectangular small white label, directly pasted on the slide > (FMNH).

*Data (R)*. Campbell (1975).

Genus *Coprotachinus* Cameron, 1933

*Coprotachinus* *habrocerinus* (Eppelsheim, 1895)

*Present classification*. Tachyporinae; Vatesini; *Coprotachinus*.

*Data type*. P + S + R.

*Data (P)*. 1 male [ventral view fully visible, dorsally glued to the paper card; abdominal terminalia removed and pasted near the body]: “Kamaiembi (Luebo)” < printed in strongly transverse, rectangular small white label >, “ - IX - 1921 | Schouteden” < printed in rectangular small white label >, “CONGO” < printed in rectangular small white label >, “diversicornis Epp [handwritten] | det.Bernhauer” < printed in rectangular small white label >, “Chicago NHMus | M.Bernhauer | Collection” < printed in rectangular small white label >, “Coprotachinus | habrocerinus | det. (Eppelsheim) | 1994, J. M. Campbell” < printed in rectangular small white label > (FMNH); 1 female: “20/30-V-1930 | Dr P. Gérard” < printed in rectangular small white label >, “MUSÉE DU CONGO | Mulongo (Niunzu)” < printed in strongly transverse, rectangular small white label, folded in twice >, “Chicago NHMus | M.Bernhauer | Collection” < printed in rectangular small white label >, “Coprotachinus | habrocerinus | det. (Eppelsheim) | 1994, J. M. Campbell” < printed in rectangular small white label, not dissected > (FMNH); 1 female: “Ituri: | Moto : Madyu | L. Burgeon” < printed in rectangular small white label >, “CONGO” < printed in rectangular small white label >, “diversicornis Epp [handwritten] | det.Bernhauer” < printed in rectangular small white label >, “Chicago NHMus | M.Bernhauer | Collection” < printed in rectangular small white label >, “Coprotachinus | habrocerinus | det. (Eppelsheim) | 1994, J. M. Campbell” < printed in rectangular small white label, not dissected > (FMNH).

*Data (S)*. 1 male: “MUSÉE DU CONGO | Mulongo (Mafinge)” < printed in strongly transverse, rectangular small white label, originally folded in twice, directly pasted on the slide >, “10/17-VII-1930 | Dr P. Gérard” < printed in rectangular small white label, directly pasted on the slide >, “Chicago NHMus | M.Bernhauer | Collection” < printed in rectangular small white label, directly pasted on the slide >, “Coprotachinus | habrocerinus | det. (Eppelsheim) | 1994, J. M. Campbell” < printed in rectangular small white label, directly pasted on the slide >, “*Coprotachinus* | *habrocerinus* | (Eppelsheim, 1895) | Tachyporinae: | Tachyporini” < printed in rectangular small white label, directly pasted on the slide > (FMNH); 1 female: “CONGO” < printed in rectangular small white label, directly pasted on the slide >, “HAUT UELE | Watsa, XI-1919 | *L*. B*urgeon*.” < printed in rectangular small white label, directly pasted on the slide >, “diversicornis Epp [handwritten] | det.Bernhauer” < printed in rectangular small white label, directly pasted on the slide >, “Chicago NHMus | M.Bernhauer | Collection” < printed in rectangular small white label, directly pasted on the slide >, “Coprotachinus | habrocerinus | det. (Eppelsheim) | 1994, J. M. Campbell” < printed in rectangular small white label, directly pasted on the slide >, “*Coprotachinus* | *habrocerinus* | (Eppelsheim, 1895) | Tachyporinae: | Tachyporini” < printed in rectangular small white label, directly pasted on the slide > (FMNH).

*Data (R)*. Campbell (1994b).

Genus *Euconosoma* Cameron, 1918

*Euconosoma* *picta* (Bernhauer, 1903)

*Present classification*. Tachyporinae; Tachyporini; Euconosomatina; *Euconosoma*.

*Data type*. P + R.

*Data (P)*. 1 male [right hindleg detached, separately glued on another paper card]: “Dohrn | Sumatra | Soekaranda” < printed in rectangular small white label >, “pictum Brh [handwritten] | det. Bernh.” < printed in rectangular small white label >, “Chicago NHMus | M.Bernhauer | Collection” < printed in rectangular small white label >, “Euconosoma [handwritten] | pictum [handwritten] | Det. (Bernh.) [(Bernh.), handwritten] | J.M.Campbell 1975 [75, handwritten]” < printed in rectangular small white label, not dissected > (FMNH); 1 female: “Quop, W. Sarawak. | G. E. Bryant. | 4. 4. 16 [handwritten]” < printed in rectangular small white label >, “G.Bryant Coll. | 1919-147” < printed in rectangular small white label >, “Chicago NHMus | M.Bernhauer | Collection” < printed in rectangular small white label >, “Conosoma | pictum Bernh.” < handwritten in rectangular small white label >,“Euconosoma [handwritten] | pictum [handwritten] | Det. (Bernh.) [(Bernh.), handwritten] | J.M.Campbell 1975 [75, handwritten]” < printed in rectangular small white label, not dissected >, <with an additional plastic card mounted on the abdominal terminalia > (FMNH).

*Data (R)*. Campbell (1976).

Genus *Lamprinodes* Luze, 1901

*Lamprinodes* *saginatus* (Gravenhorst, 1806)^§^

*Present classification*. Tachyporinae; Tachyporini; Tachyporina; *Lamprinodes*.

*Data type*. P + S.

*Data (P)*. 1 male [attached with two possible host ants of the same species, each mounted on a separated paper card] [two possible host ants also attached, each mounted on a separate paper card]: “Bruck N.Ö. | Bernhauer” < printed in rectangular small white label >, “saginatus Gr. [handwritten] | det. Bernh.” < printed in rectangular small white label >, “Chicago NHMus | M.Bernhauer | Collection” < printed in rectangular small white label >, “Lamprinodes [handwritten] | saginatus [handwritten] | M. Bernhauer [handwritten] | det. ~~A.Newton 2015~~” < printed in rectangular small white label, not dissected > (FMNH); 1 female: “L. Enzersdf. [Enzersdorf] | Luze” < printed in rectangular small white label >, “saginatus Gvh | Niederösterrch [Niederösterreich, = Lower Austria] | ded. Luze.” < printed in rectangular small white label >, “Chicago NHMus | M.Bernhauer | Collection” < printed in rectangular small white label, not dissected > (FMNH); 1 female: “Reichenberg | Boh. Bang Hs [= Bang Haas, a collector or dealer]” < handwritten in rectangular small white label >, “saginatus Grh. [handwritten] | det. Bernh.” < printed in rectangular small white label >, “Chicago NHMus | M.Bernhauer | Collection” < printed in rectangular small white label, not dissected > (FMNH).

*Data (S)*. 1 female: “Thüringen” < handwritten in rectangular small white label, directly pasted on the slide >, “CNHM 1955 | Karl Brancsik Colln. | ex Eduard Knirsch” < printed in rectangular small white label, directly pasted on the slide >, “*Lamprinodes* | *saginatus* | (Gravenhorst, 1806) | Tachyporinae: | Tachyporini” < printed in rectangular small white label, directly pasted on the slide > (FMNH).

Genus *Lamprinus* Heer, 1839

*Lamprinus* *erythropterus* (Panzer, 1796)^§^

*Present classification*. Tachyporinae; Tachyporini; Tachyporina; *Lamprinus*.

*Data type*. P + S.

*Data (P)*. 1 male: “Trag” < printed in rectangular small white label >, “CNHM 1955 | Karl Brancsik Colln. | ex Eduard Knirsch” < printed in rectangular small white label >, <with an additional plastic card mounted on the abdominal terminalia > (FMNH); 1 female: “Kalávryta | Morea, Holtz” < printed in rectangular small white label >, “erythropterus [handwritten] | Holtz [handwritten] | det. Bernhau” < printed in rectangular small white label >, “Chicago NHMus | M.Bernhauer | Collection” < printed in rectangular small white label, not dissected > (FMNH); 1 female: “Lg.-Enzersd. | Luze” < printed in rectangular small white label >, “erythropterus | Pz. A. v?f. [?, overlapping with a hole of the insect pin] | ded. Luze” < handwritten in rectangular small white label >, “Chicago NHMus | M.Bernhauer | Collection” < printed in rectangular small white label, not dissected > (FMNH); 1 female: “Lg.-Enzersd. | Luze” < printed in rectangular small white label >, “erythropter. | Luze don.” < handwritten in rectangular small white label >, “CNHM 1955 | Eduard Knirsch | Palaearctic Colln.” < printed in rectangular small white label, not dissected > (FMNH).

*Data (S)*. 1 female [pinned together with the above listed male specimen]: “Trag” < printed in rectangular small white label, directly pasted on the slide >, “CNHM 1955 | Karl Brancsik Colln. | ex Eduard Knirsch” < printed in rectangular small white label, directly pasted on the slide >, “*Lamprinus* | *erythropterus* | (Panzer, 1796) | Tachyporinae: | Tachyporini”, < printed in rectangular small white label, directly pasted on the slide > (FMNH).

Genus *Leucotachinus* Coiffait & Saiz, 1968

*Leucotachinus* *luteonitens* (Fairmaire & Germain, 1861)^§^

*Present classification*. Tachyporinae; Tachinusini; *Leucotachinus*.

*Data type*. P + S.

*Data (P)*. 1 male: “CHILE: Osorno Pr. | Puyehue, Aguas | Calientes, 500m | 2-6.i.1982 L.Peña” < printed in rectangular small white label >, “Leucotachinus | luteonitens | (Fairm. & Germain) | Det. J.M.Campbell 1993” < printed in rectangular small white label, not dissected > (FMNH); 1 female: “CHILE: Valdivia | Valdivia, XII.1982 | L.E.Peña” < printed in rectangular small white label >, “Leucotachinus | luteonitens | (Fairm. & Germain) | Det. J.M.Campbell 1993” < printed in rectangular small white label, not dissected > (FMNH); 1 female: “Corral. CHILE | Dec. 1905 | R. Thaxter” < printed in rectangular small white label >, “Leucotachinus | luteonitens | (Fairm. & Germain) | Det. J.M.Campbell 1993” < printed in rectangular small white label, not dissected > (FMNH).

*Data (S)*. 1 male: “CHILE: Osofno | Anticura | 19-29. X. 1985 | L. E. Pexa” < handwritten in rectangular small white label, directly pasted on the slide >, “Leucotachinus | luteonitens | (Fairm. & Germain) | Det. J.M.Campbell 1993” < printed in rectangular small white label, directly pasted on the slide >, “*Leucotachinus* | *luteonitens* | (Fairmaire & Germain, | 1861)” < printed in rectangular small white label, directly pasted on the slide >, “Tachyporinae: | Tachyporini” < printed in rectangular small white label, directly pasted on the slide > (FMNH); 1 female: “Alunalhue | Villarrica” < handwritten in rectangular small white label, directly pasted on the slide >, “16.II.1979 | P. Vidal” < handwritten in rectangular small white label, directly pasted on the slide >, “FMNH 1986 | L.E. Peña Colln. | [Santiago, Chile] | Acc. # 17-422.” < printed in rectangular small white label, directly pasted on the slide >, “Leucotachinus | luteonitens | (Fairm. & Germain) | Det. J.M.Campbell 1993” < printed in rectangular small white label, directly pasted on the slide >, “Leucotachinus [handwritten] | det. Newton, 1991” < printed in rectangular small white label, directly pasted on the slide > (FMNH).

*Leucotachinus* *novitius* (Blackburn, 1891)

*Present classification*. Tachyporinae; Tachinusini; *Leucotachinus*.

*Data type*. P + S + W.

*Data (P)*. 1 female: “AUSTL.: VIC.: Errinun- | dra N.P., Coast Range | Rd., 8.8km E jct. | Gunmark Rd., 1020m, | 37°17'S, 148°57'E,” < printed in rectangular small white label >, “11-24.II.1993, cool | temperate rainforest; | FMHD #93-82, window | trap, A.Newton & | M. Thayer 925 | FIELD MUS. NAT. HIST.” < printed in rectangular small white label >, “Leucotachinus [handwritten] | novitius [handwritten] | (Blackburn) [handwritten] | det. A.Newton 2005” < printed in rectangular small white label, not dissected > (FMNH); 1 female: “Mt. Kosciusko | 4-5,000 ft. | Dec. 1931” < printed in rectangular small white label >, “Australia | Harvard Exp., | Darlington” < printed in rectangular small white label >, “MCZ” < handwritten in rectangular small white label >, <with an additional small plastic tube, preserving the separated abdominal terminalia > (FMNH).

*Data (S)*. 1 female: “♀ A | Leuco- | ‘Tachinus’ | novitius | Blkbn. | →Leucotachinus | Aust: N.S.W. | Mt. Kosciusko | 5-7000; Dec. | 1931, Darlington | PM” < directly handwritten on the slide > (FMNH, cAN).

*Data (W)*. 1 male: “Mt Kosciusko | 5-7,000 ft. N. S.W. | Dec. 1931” < printed in rectangular small white label >, “Australia | Harvard Exp., | Darlington” < printed in rectangular small white label >, “Collection | A. Newton” < printed in rectangular small blue label >, “ “Tachinus” [handwritten] | novitius [handwritten] | Bikbn. [handwritten] | ♂♀ [handwritten] | A. Newton det 1979 [9, handwritten]” < printed in rectangular small white label > (FMNH, cAN).

Genus *Mimocyptus* Cameron, 1919

*Mimocyptus* sp.

*Present classification*. Tachyporinae; Vatesini; *Mimocyptus*.

*Data type*. P + S.

*Data (P)*. 2 males, 1 female: “BORNEO Sabah Mt. | Kinabalu Nat.PK. | Poring Hot Spgs. | 485m 29.VIII.88. | A. Smetana[B160]” < printed in rectangular small white label >, “FIELD MUSEUM | ex collection of | H. G. Nelson” < printed in rectangular small white label, not dissected > (FMNH).

*Data (S)*. 1 male: “BORNEO Sabah Mt. | Kinabalu Nat.PK. | Poring Hot Spgs. | 490m 16.VIII.88 | A. Smetana [B118]” < printed in rectangular small white label, directly pasted on the slide >, “FIELD MUSEUM | ex collection of | H. G. Nelson” < printed in rectangular small white label, directly pasted on the slide >, “*Mimocyptus* sp. | Tachyporinae: | Tachyporini” < printed in rectangular small white label, directly pasted on the slide > (FMNH); 1 female: “BORNEO Sabah Mt | Kinabalu Nat.PK. | Poring Hot Spgs. | 485m 29.VIII.88. | A. Smetana[B160]” < printed in rectangular small white label, directly pasted on the slide >, “FIELD MUSEUM | ex collection of | H. G. Nelson” < printed in rectangular small white label, directly pasted on the slide > (FMNH).

Genus *Nitidotachinus* Campbell, 1993

*Nitidotachinus* *scrutator* (Gemminger & Harold, 1868)

*Present classification*. Tachyporinae; Tachinusini; *Nitidotachinus*.

*Data type*. P + S + R.

*Data (P)*. 1 male: “MO:CarterCo. | FourHoleCave | 11mi SWVanBuren | 17. IX. 80; JEGardner” < printed in rectangular small white label >, “Tachinus Nitido- [handwritten] | scrutator G. & H. [handwritten] | det.J.M.Campbell” < printed in rectangular small white label >, < with an additional glass card mounted on the abdominal terminalia > (FMNH); 1 female: “MASS: Middlesex | Co., Estabrook | Woods, Concord | VII. 10-18. 1976 [handwritten]” < printed in rectangular small white label >, “wet moss | stream edge” < printed in rectangular small white label >, “A. Newton | M. Thayer | Collectors” < printed in rectangular small white label >, “J. F. Lawrence | Collector” < printed in rectangular small white label >, < with an additional glass card mounted on the abdominal terminalia > (FMNH).

*Data (S)*. 1 male: “♂ A | Nitidotachinus | ~~Tachinus~~ | scrutator | G & H | MASS.: Mdsx [Middlesex] Co. | Estabrook Woods | nr. Concord | VIII. 10. 1974 | Sphagnum moss | A. N. CB” < directly handwritten on the slide> (FMNH, cAN); 1 female: “♀ A | Nitidotachinus | ~~Tachinus~~ | scrutator | G + H | MASS.: Mdsx. Co. | Concord | VIII. 10. 1974 | Sphagnum moss | A. Newton CB” < directly handwritten on the slide> (FMNH, cAN).

*Data (R)*. Campbell (1993b).

*Nitidotachinus* *tachyporoides* (Horn, 1877)^§^

*Present classification*. Tachyporinae; Tachinusini; *Nitidotachinus*.

*Data type*. P + S + R.

*Data (P)*. 1 male: “N.H.: Grafton Co., | Zealand R. 2200’ | 6miSE Twin Mtn. | IX. 5. 1975” < printed in rectangular small white label >, “A. Newton | & M. Thayer | collectors” < printed in rectangular small white label >, “wet debris, | small stream” < printed in rectangular small white label, not dissected > (FMNH); 1 female: “N. H.: Coos Co., Zmi | N Crawford House | 2000’ IX.6.1975 | A. Newton, M. Thayer” < printed in rectangular small white label >, “wet moss | stream edge” < printed in rectangular small white label, not dissected > (FMNH).

*Data (S)*. 1 male, 1 female: “N.H.: Grafton Co. | Sawyer River nr. | Bartlett, 1600’ | VIII.25.1974” < printed in rectangular small white label, directly pasted on the slide >, “A. Newton | & M. Thayer | collectors” < printed in rectangular small white label, directly pasted on the slide >, “wet moss | stream edge” < printed in rectangular small white label, directly pasted on the slide >, “*Nitidotachinus* | *tachyporoides* | (Horn, 1877)” < printed in rectangular small white label, directly pasted on the slide > (FMNH).

*Data (R)*. Campbell (1993b).

Genus *Olophrinus* Fauvel, 1895

*Olophrinus* cf. *philippinus* Campbell, 1993

*Present classification*. Tachyporinae; Tachinusini; *Olophrinus*.

*Data type*. P + S.

*Data (P)*. 1 male, 1 female [hindwings for the male detached, but mounted on another paper card]: “Philippines: Mindanao, | Bukidnon, Panamokan, XII” < printed in rectangular small white label >, “*Olophrinus* cf. *philippinus* | Campbell, 1993 | Det. S. Yamamoto, 2017” < printed in rectangular small white label, not dissected > (FMNH).

*Data (S)*. 1 male, 1 female: “Philippines: Mindanao, | Bukidnon, Panamokan, XII” < printed in rectangular small white label, directly pasted on the slide >, “*Olophrinus* cf. *philippinus* | Campbell, 1993 | Det. S. Yamamoto, 2017” < printed in rectangular small white label, directly pasted on the slide > (FMNH).

Genus *Pseudotachinus* Cameron, 1932

*Pseudotachinus* *besucheti* Schülke, 2005

*Present classification*. Tachyporinae; Tachinusini; *Pseudotachinus*.

*Data type*. P + R.

*Data (P)*. 1 male: “PAKISTAN: Swat | Manglaur | 1150m, 9.V.83 | Besuchet-Löbl” < printed in rectangular small white label >, “PARATYPUS | Pseudotachinus | besucheti spec. nov. | det. M. Schülke, 2004” < printed in rectangular small yellow label >, “MHNG | ENTO | 00012103” < printed in rectangular small white label, not dissected > (MHNG); 1 female: “PAKISTAN: Swat | Saidu Sharif | 1000 m; 11.V.1983 | Besuchet - Löbl” < printed in rectangular small white label >, “PARATYPUS | Pseudotachinus | besucheti spec. nov. | det. M. Schülke, 2004” < printed in rectangular small yellow label >, “MHNG | ENTO | 00012100” < printed in rectangular small white label, not dissected > (MHNG).

*Data (R)*. Schülke (2005).

Genus *Sepedophilus* Gistel, 1856

*Sepedophilus* *bisignatus* (Horn, 1877)

*Present classification*. Tachyporinae; Tachyporini; Euconosomatina; *Sepedophilus*.

*Data type*. P + S.

*Data (P)*. 1 male, 1 female: “CALF: Amador Co., | PeddlerHill 7000’ | VI.27-28.1975 | A. Newton, M. Thayer” < printed in rectangular small white label >, “under bark | conifer” < printed in rectangular small white label, not dissected >, <with an additional identification label for the male: “Sepedophilus [handwritten] | bisignatus [handwritten] | det. A.Newton ~~2015~~ 1983 [1983, handwritten]” [printed in rectangular small white label] > (FMNH).

*Data (S)*. 1 male, 1 female: “♂♀ C [red letter] A | Sepedophilus | bisignatus | (Horn) | CAL: Plumas | Co., Butt Val. Dam | 4000’ VI.30.75 | JFL 3981 [= JF. Lawrence fungus lot number 3981] | Fomitopsis | pinicola | ANMT [= A. Newton & M. Thayer] PM” < directly handwritten on the slide>, <with additional white rectangular seal on the right side: “PM D - RL” [handwritten] >, “VOUCHER | Associated | with larva” < printed in rectangular small green label, directly pasted on the slide > (FMNH, cAN).

*Sepedophilus* *cinctulus* (Erichson, 1839)

*Present classification*. Tachyporinae; Tachyporini; Euconosomatina; *Sepedophilus*.

*Data type*. P + S.

*Data (P)*. 1 male: “Ohio; Franklin | Co., Columbus | 13-IX-1975” < printed in rectangular small white label >, “Berlese, forest | floor litter | L. E. Watrous” < printed in rectangular small white label, not dissected > (FMNH); 1 female: “Ohio; Franklin | Co., Columbus | 13-IX-1975” < printed in rectangular small white label >, “Berlese rotten | wood debris | LEWatrous” < printed in rectangular small white label >, “Sepedophilus [handwritten] | cinctulus [handwritten] | J. M. Campbell [handwritten] | det. ~~A. Newton 2015~~” < printed in rectangular small white label, not dissected > (FMNH).

*Data (S)*. 1 male, 1 female: “♂♀ A [red letter] A | Sepedophilus | cinctulus | (Er.) | N.H.: Hillsb. Co., | Greenfield | Aug, 5, 1973 | JFL 3465 | Cor. [=Coriolus] versi- | color | PM” < directly handwritten on the slide>, <with additional white rectangular seal on the right side: “PM dorsal” [handwritten] >, “VOUCHER | Associated | with larva” < printed in rectangular small green label, directly pasted on the slide > (FMNH, cAN).

*Sepedophilus* *crassus* (Gravenhorst, 1802)

*Present classification*. Tachyporinae; Tachyporini; Euconosomatina; *Sepedophilus*.

*Data type*. P + S.

*Data (P)*. 1 male, 1 female: “Ohio: Franklin | Co., Columbus | 13-IX-1975” < printed in rectangular small white label >, “LEWatrous | colr.” < printed in rectangular small white label, not dissected >, <with an additional identification label for the female: “Sepedophilus [handwritten] | crassus [handwritten] | J.M. Campbell [handwritten] | det. ~~A.Newton 2015~~” [printed in rectangular small white label] > (FMNH).

*Data (S)*. 1 male [without elytra and hindwings]: “♂ C [red letter] A | Sepedophilus | basalis (Er.) | crassus (Grav.) | LA.: Rapides P. | Magnolia Rec. | Area X. 4.73 | on Pleurotis [= Pleurotus] | A. Newton CB” < directly handwritten on the slide>, “VOUCHER | Associated | with larva” < printed in rectangular small green label, directly pasted on the slide > (FMNH, cAN); 1 female [without elytra and hindwings, mounted together with the dissected specimen of *Sepedophilus versicolor*]: “♂♀ C [red letter] A | Sepedophilus | crassus ♀ | versicolor ♂ | LA.: Rapides | Par., Magnolia | Rec. Area, 15 | mi SW Alex- | andria X.4.73 | Pleurotus AFN | PM” < directly handwritten on the slide>, “VOUCHER | Associated | with larva” < printed in rectangular small green label, directly pasted on the slide > (FMNH, cAN); 1 female [elytra and hindwings only, mounted together with those of *Sepedophilus versicolor*]: “C [red letter] W | Sepedophilus | ~~basalis (Er.)~~ | crassus (Grav.) (2) | + vers. (1) | LA.: Rapides P. | Magnolia Rec. | Area, X. 4. 1973 | on Pleurotis | A. Newton | CB” < directly handwritten on the slide>, “VOUCHER | Associated | with larva” < printed in rectangular small green label, directly pasted on the slide > (FMNH, cAN).

*Sepedophilus* *littoreus* (Linnaeus, 1758)^§^

*Present classification*. Tachyporinae; Tachyporini; Euconosomatina; *Sepedophilus*.

*Data type*. P + S.

*Data (P)*. 1 male: “U.S.A.: ILLINOIS | Kane Co.; Forest Pre- | serve on AH.30. 3 mi. S. | Lily Lake. 22:XI:1960. | Leg: W. Suter.” < printed in rectangular small white label >, “Berlese: Stage | IV oak log.” < printed in rectangular small white label, not dissected > (FMNH); 1 female: “III.” < printed in square small white label >, “F M N H Coll. | No. | (E Chope Coll)” < printed in rectangular small white label >, “Sepedophilus | littoreus (L.) | det. 1974 | J.M.Campbell” < printed in rectangular small white label, not dissected > (FMNH).

*Data (S)*. 1 male: “MO: Phelps Co. | Yancy Mills#1Cave | 16.I.79;JEGardner” < printed in rectangular small white label, directly pasted on the slide >, “Sepedophilus [handwritten] | littoreus (L.) [handwritten] | Det.J.M.Campbell” < printed in rectangular small white label, directly pasted on the slide >, “*Sepedophilus* | *littoreus* | (Linnaeus, 1758)” < printed in rectangular small white label, directly pasted on the slide > (FMNH); 1 female: “Ontario; Essex | Co., Wheatley | July 1967 | K.Stephan leg.” < printed in rectangular small white label, directly pasted on the slide >, “Field Mus. Nat. Hist. | Karl H. Stephen | Collection, 1977 | Acc. No. Z-15, 639” < printed in rectangular small white label, directly pasted on the slide >, “Sepedophilus | littoreus (L.) | det. 1974 | J.M.Campbell” < printed in rectangular small white label, directly pasted on the slide >, “*Sepedophilus* | *littoreus* | (Linnaeus, 1758)” < printed in rectangular small white label, directly pasted on the slide > (FMNH).

*Sepedophilus* *scriptus* (Horn, 1877)

*Present classification*. Tachyporinae; Tachyporini; Euconosomatina; *Sepedophilus*.

*Data type*. P + S.

*Data (P)*. 1 male, 1 female: “FLA.:Jackson Co.: | Fla. Caverns St. Pk. | IV:6:1969 | leg. S. Peck” < printed in rectangular small white label >, “FM(HD)#69-3 | Berlese:#159 | log leaf litter | 131 lbs., 130 liters” < printed in rectangular small white label >, <with an additional identification label for the female: “Sepedophilus [handwritten] | scriptus [handwritten] | J.M. Campbell [handwritten] | det. ~~A.Newton 2015~~” [printed in rectangular small white label] >, < with an additional glass card mounted on the abdominal terminalia only for female; male not dissected > (FMNH).

*Data (S)*. 1 male, 1 female: “♂♀ B [red letter] A | Sepedophilus | scriptus | (Hn.) | TEX: Bastrop | Co., Bastrop St. | Pk., VI.17.1973 | on fungusy | oak log | AFN PM” < directly handwritten on the slide>, “VOUCHER | Associated | with larva” < printed in rectangular small green label, directly pasted on the slide > (FMNH, cAN).

Genus *Symmixus* Bernhauer, 1915

*Symmixus* *sikkimensis* Bernhauer, 1915^§^

*Present classification*. Tachyporinae; Tachyporini; Tachyporina; *Symmixus*.

*Data type*. P + R.

*Data (P)*. 1 male: “Sikkim” < printed in rectangular small white label >, “nov. gen et spec | BangHaas” < handwritten in rectangular small white label >, “Ost-Himalaya, | ex Coll Waagen | ded. Bang-Haas” < printed in rectangular small white label >, “Symmixus | sikkimensis | Bernh. Typus un.” < handwritten in rectangular small yellow label >, “Chicago NHMus | M.Bernhauer | Collection” < printed in rectangular small white label >, “Tachyporus [handwritten] | (Symmixus)- [handwritten] | sikkimensis Bernh. [handwritten] | Det. J.M. Campbell” < printed in rectangular small white label, not dissected > (FMNH).

*Data (R)*. Schülke (2003).

Genus *Tachinomorphus* Kraatz, 1859

*Tachinomorphus* *grossulus* (LeConte, 1863)

*Present classification*. Tachyporinae; Tachinusini; *Tachinomorphus*.

*Data type*. P + S + R.

*Data (P)*. 2 males, 1 female: “ARIZ.: Pinal Co.; | Florence (20 mi S). | dying-fallen saguaro. | leg: W. Suter. 20:VIII:1978.” < printed in rectangular small white label, not dissected > (FMNH).

*Data (S)*. 1 male, 1 female: “ARIZ.: Pinal Co.; | Florence (20 mi S). | dying-fallen saguaro. | leg: W. Suter. 20:VIII:1978.” < printed in rectangular small white label, directly pasted on the slide >, “*Tachinomorphus* | *grossulus* | (LeConte, 1863) | det. S. Yamamoto, 2018” < printed in rectangular small white label, directly pasted on the slide > (FMNH).

*Data (R)*. Campbell (1973a).

Genus *Tachinoplesius* Bernhauer, 1936

*Tachinoplesius* *latipennis* Schülke, 2006

*Present classification*. Tachyporinae; Tachinusini; *Tachinoplesius*.

*Data type*. P + R.

*Data (P)*. 1 male [ventral view fully visible, dorsally glued to the paper card; fore- and hindlegs removed and glued on the paper card near the body]: “UGANDA | Kibale Forest | 1250m, 23.V.1993 | Cuccodoro&Erne # 12A” < printed in rectangular small white label >, “PARATYPUS | *Tachinoplesius* | *latipennis* spec. nov. | det. M. Schülke 2005” < printed in rectangular small yellow label >, “MHNG | ENTO | 00012115” < printed in rectangular small white label >, <with an additional plastic card mounted on the mouthparts > (MHNG); 1 female: “UGANDA | Kibale Forest | 1250m, 25.V.1993 | Cuccodoro&Erne # 14B” < printed in rectangular small white label >, “PARATYPUS | *Tachinoplesius* | *latipennis* spec. nov. | det. M. Schülke 2005” < printed in rectangular small yellow label >, “MHNG | ENTO | 00012118” < printed in rectangular small white label >, <with an additional plastic card mounted on the abdominal terminalia > (MHNG).

*Data (R)*. Schülke (2006).

Genus *Tachinus* Gravenhorst, 1802

Subenus *Tachinus* Gravenhorst, 1802

*Tachinus* (*Tachinus*) *frigidus* Erichson, 1839

*Present classification*. Tachyporinae; Tachinusini; *Tachinus*.

*Data type*. P + S + R.

*Data (P)*. 1 male, 1 female: “Wrangell, ALASKA | 5-15.VIII.1951 | B.Malkin” < printed in rectangular small white label >, “C.N.H.M. 1960 | Borys Malkin | Coleoptera Colln.” < printed in rectangular small white label >, <with an additional identification label for the male: “Tachinus [handwritten] | frigidus [handwritten] | J.M. Campbell [handwritten] | det. ~~A.Newton 2015~~” [printed in rectangular small white label] > (FMNH).

*Data (S)*. 1 male, 1 female: “♂♀ A | Tachinus | frigidus Er.” < directly handwritten on the slide>, “N.H.: Coos Co. | 0.3mi S Jefferson | Notch, 895m, | Picea-Abies for. | 23-27.VIII.1980 | A. Newton, M. Thayer” < printed in rectangular small white label, directly pasted on the slide >, “Lot 80-G | Myxomycete [sic.; Myxomycetes] | PM” < directly handwritten on the slide>, < with additional comment on the right side of the slide: “PM RL” [directly handwritten on the slide] > (FMNH, cAN).

*Data (R)*. Campbell (1973b).

*Tachinus* (*Tachinus*) *fumipennis* Say, 1832

*Present classification*. Tachyporinae; Tachinusini; *Tachinus*.

*Data type*. P + S + R.

*Data (P)*. 1 male, 1 female: “USA:IN:Porter Co.:Indiana | Dunes Nat. Lakeshore Park | VI-24-2007 | ex. rotting *Laetiporus* sp. Fungi | Taro Eldredge” < printed in rectangular small yellowish-white label >, “Tachinus [handwritten] | fumipennis [handwritten] | det. Taro Eldredge ’08 [’08, handwritten]” < printed in rectangular small yellowish-white label, not dissected > (KUM).

*Data (S)*. 1 male, 1 female: “USA:IN:Porter Co.:Indiana | Dunes Nat. Lakeshore Park | VI-24-2007 | ex. rotting *Laetiporus* sp. Fungi | Taro Eldredge” < printed in rectangular small yellowish-white label, directly pasted on the slide >, “Tachinus [handwritten] | fumipennis [handwritten] | det. Taro Eldredge ’08 [’08, handwritten]” < printed in rectangular small yellowish-white label, directly pasted on the slide > (FMNH).

*Data (R)*. Campbell (1973b).

*Tachinus* (*Tachinus*) *rufipes* (Linnaeus, 1758)^§^

*Present classification*. Tachyporinae; Tachinusini; *Tachinus*.

*Data type*. P + S.

*Data (P)*. 1 male [male genitalia fully exposed]: “SWITZERLAND, | Les Diablarets | (Vaud). 1100 m.” < printed in rectangular small white label >, “3-31.VIII. [handwritten] 1976 [6, handwritten] | B. ~~and H~~. Malkin” < printed in rectangular small white label >, “Staphylinidae | Tachinus rufipes (L.) | det. Marcin Smoleński” < printed in rectangular small white label, not dissected > (FMNH); 1 male: “POLAND: Choinów | woi. warszawskie | 16. IX. [handwritten] 1973 [3, handwritten] | B. and H. Malkin” < printed in rectangular small white label >, “Staphylinidae | Tachinus rufipes (L.) | det. Marcin Smoleński” < printed in rectangular small white label, not dissected > (FMNH); 1 female: “POLAND: Gorzyczki | nr. Wodzisławice, | woj. katowickie” < printed in rectangular small white label >, “19.III.1973 | A. Kuśka” < printed in rectangular small white label >, “Staphylinidae | Tachinus rufipes (L.) | det. Marcin Smoleński” < printed in rectangular small white label, not dissected > (FMNH); 1 female: “West Germany | Karlsruhe | May 1950 | K. Stephan leg.” < printed in rectangular small white label >, “19.III.1973 | A. Kuśka” < printed in rectangular small white label >, “Field Mus. Nat. Hist. | Karl H. Stephen | Collection, 1977 | Acc. No. Z-15, 639” < printed in rectangular small white label >, “Tachinus | rufipes (Deg.) [handwritten] | det. | J. M. Campbell” < printed in rectangular small white label, not dissected > (FMNH).

*Data (S)*. 1 male, 1 female: “SWITZERLAND: | Langendorf | /Solothurn/” < printed in rectangular small white label, directly pasted on the slide >, “12.VII. [handwritten] 1979 [79, handwritten] | B. and H. Malkin” < printed in rectangular small white label, directly pasted on the slide >, “Staphylinidae | Tachinus rufipes (L.) | det. Marcin Smoleński” < printed in rectangular small white label, directly pasted on the slide > (FMNH).

Genus *Tachyporus* Gravenhorst, 1802*

Subgenus *Palporus* Campbell, 1979

*Tachyporus* (*Palporus*) *nitidulus* (Fabricius, 1781)

*Present classification*. Tachyporinae; Tachyporini; Tachyporina; *Palporus*.

*Data type*. P + S.

*Data (P)*. 2 males, 1 female: “U.S.A.: OHIO. Marion | Co.; Waldo (N) | 2:VI:1978. | Leg: W. Suter.” < printed in rectangular small white label >, “WS# 78-34B | Ash-bush | pseudo fork.” < printed in rectangular small white label, not dissected > (FMNH); 2 females: “U.S.A.: OHIO. Marion | Co.; Waldo (N) | 2:VI:1978. | Leg: W. Suter.” < printed in rectangular small white label >, “WS# 78-34C | Moss | on log.” < printed in rectangular small white label, not dissected >, <with an additional identification label for the single female: “Tachyporus [handwritten] | nitidulus Fab. [handwritten in blue ink] | Det. | J.M.Campbell 1981 [81, handwritten in blue ink]” [printed in rectangular small white label] > (FMNH).

*Data (S)*. 1 male, 1 female: “U.S.A.: Maine; Han- | cock Co.; W. Goulds- | boro. 21:X:1967 | Leg: S. Peck.” < printed in rectangular small white label, directly pasted on the slide >, “FM(HD)# 67-116 | Ber. #105. | Spruce duff.” < printed in rectangular small white label, directly pasted on the slide >, <with an additional identification label for the male: “*Tachyporus* | *nitidulus* | Fabricius, 1781” [printed in rectangular small white label, directly pasted on the slide] >, <with an additional identification label for the female: “*Tachyporus* | (*Palporus*) | *nitidulus* | (Fabricius, 1781) | Tachyporinae: | Tachyporini” [printed in rectangular small white label, directly pasted on the slide] > (FMNH); 1 male: “U.S.A.: OHIO. Marion | Co.; Waldo (N) | 2:VI:1978. | Leg: W. Suter.” < printed in rectangular small white label, directly pasted on the slide >, “WS# 78-34B | Ash-bush | pseudo fork.” < printed in rectangular small white label, directly pasted on the slide >, “*Tachyporus* | (*Palporus*) | *nitidulus* | (Fabricius, 1781) | Tachyporinae: | Tachyporini” < printed in rectangular small white label, directly pasted on the slide > (FMNH); 1 female: “Ohio:Franklin Co. | O’ Shaughnessy Res. | 20-III-1977” < printed in rectangular small white label, directly pasted on the slide >, “berlese hardwood | litter LEWatrous” < printed in rectangular small white label, directly pasted on the slide >, “FM(HD)#76-192. | WS#76-79b. | nest u. can on | water hyacinth” < printed in rectangular small white label, directly pasted on the slide >, “*Tachyporus* *nitidulus* | (Fabricius, 1781) | Det. J. M., Campbell” < printed in rectangular small white label, directly pasted on the slide > (FMNH).

Subgenus *Tachyporus* Gravenhorst, 1802

*Tachyporus* (*Tachyporus*) *chrysomelinus* (Linnaeus, 1758)^§^

*Present classification*. Tachyporinae; Tachyporini; Tachyporina; *Tachyporus*.

*Data type*. P + S.

*Data (P)*. 1 male: “SWITZERLAND: | Oberdorf, | /Solothurn/” < printed in rectangular small white label >, “29.V. [handwritten] 1982 [82, handwritten] | B. ~~and H.~~ Malkin” < printed in rectangular small white label >, “Staphylinidae | Tachyporus chrysomelinus (L.) | det. Marcin Smoleński” < printed in strongly transverse, rectangular white label >, < with an additional glass card mounted on the abdominal terminalia > (FMNH); 1 female: “SWITZERLAND: | (Solothurn). | Borys Malkin” < printed in rectangular small white label >, “Rüttinen, | I.VI. [handwritten] 1978 [8, handwritten]” < printed in rectangular small white label >, “Staphylinidae | Tachyporus chrysomelinus (L.) | det. Marcin Smoleński” < printed in strongly transverse, rectangular white label, not dissected > (FMNH).

*Data (S)*. 1 female: “SWITZERLAND: | Oberdorf | (Solothurn)” < printed in rectangular small white label, directly pasted on the slide >, “2.VI. [handwritten] 1978 [8, handwritten] | B. ~~and H.~~ Malkin” < printed in rectangular small white label, directly pasted on the slide >, “Staphylinidae | Tachyporus chrysomelinus (L.) | det. Marcin Smoleński” < printed in rectangular small white label, directly pasted on the slide > (FMNH).

*Tachyporus* (*Tachyporus*) *jocosus* Say, 1832

*Present classification*. Tachyporinae; Tachyporini; Tachyporina; *Tachyporus*.

*Data type*. P + S.

*Data (P)*. 1 male: “Fla.: Tallahassee | Leon Co.21-X-1976 | Berlese hardwood | litter LDJustice” < printed in rectangular small white label >, “Tachyporus [handwritten] | jocosus [handwritten] | J. M. Campbell [handwritten] | det. ~~A. Newton 2015~~” < printed in rectangular small white label, not dissected > (FMNH); 1 female: “Fla.: Tallahassee | LeonCo. 28-VI-1976 | Marshall & Justice” < printed in rectangular small white label >, “Berlese mixed | hardwood litter” < printed in rectangular small white label, not dissected > (FMNH).

*Data (S)*. 1 male: “♂ A | Tachyporus | jocosus Say | TEX.: Bastrop Co. | Buescher St. Pk. | VI. 15-16. 1973 | berl., litter, oak- | pine forest | A. Newton CB” < directly handwritten on the slide> (FMNH, cAN); 1 female: “♀ A | Tachyporus | jocosus Say | TEX.: Bastrop | Co. Buescher | St. Pk., VI. 15-16. | 1973, ber., | forest litter | A. Newton CB” < directly handwritten on the slide> (FMNH, cAN).

*Tachyporus* (*Tachyporus*) *obtusus* (Linnaeus, 1767)

*Present classification*. Tachyporinae; Tachyporini; Tachyporina; *Tachyporus*.

*Data type*. P + S.

*Data (P)*. 1 male: “SWITZERLAND: | Bellach | (Solothurn)” < printed in rectangular small white label >, “23.X. [handwritten] 1976 [6, handwritten] | B. ~~and H.~~ Malkin” < printed in rectangular small white label >, “*Tachyporus* | *obtusus* | (Linnaeus, 1767)” < printed in rectangular small white label >, < with an additional glass card mounted on the abdominal terminalia > (FMNH); 1 female: “SWITZERLAND: | Bellach | (Solothurn)” < printed in rectangular small white label >, “5.VIII. [handwritten] 1977 [7, handwritten] | B. ~~and H.~~ Malkin” < printed in rectangular small white label >, “Tachyporus [handwritten] | obtusus (L.) [handwritten] | det.E.Ulbrich 1980 [80, handwritten]” < printed in rectangular small white label >, “*Tachyporus* | *obtusus* | (Linnaeus, 1767)” < printed in rectangular small white label, not dissected > (FMNH).

*Data (S)*. 1 female: “SWITZERLAND: | La Neuveville, | (Bern). 9.VI. [9.VI., handwritten] 1976. [6, handwritten] | B. and H. Malkin” < printed in rectangular small white label, directly pasted on the slide >, “*Tachyporus* | *obtusus* | (Linnaeus, 1767)” < printed in rectangular small white label, directly pasted on the slide > (FMNH).

Genus *Termitoplus* Silvestri, 1946

*Termitoplus* *grandis* Silvestri, 1946^§^

*Present classification*. Tachyporinae; Vatesini; *Termitoplus*.

*Data type*. P + S + R.

*Data (P)*. 1 male, 1 female: “Sao Paulo [handwritten] | S.P., BRAZIL | VII-3- [handwritten] 1951 [51, handwritten]” < printed in rectangular small white label >, “R.L.Aráujo leg. | No. 3. 372. [3. 372, handwritten]” < printed in rectangular small white label >, with additional identification label for the male specimen: “Termitoplus [handwritten] | grandis [handwritten] | C. Seevers [handwritten] | det. ~~A. Newton 2015~~” < printed in rectangular small white label >, < with an additional glass card mounted on the abdominal terminalia, only for the male > (FMNH).

*Data (S)*. 1 female: “♀ A | Termitoplus | grandis | Silvestri” < directly handwritten on the slide>, “Sao Paulo [handwritten] | S.P., BRAZIL | VII-3- [handwritten] 1951 [51, handwritten]” < printed in rectangular small white label, directly pasted on the slide >, “R.L.Aráujo leg. | No. 3. 372. [3. 372, handwritten]” < printed in rectangular small white label, directly pasted on the slide >, < with additional green round seal on the right side: “3.88 [handwritten; it means that the slide was made by Al. Newton on March 1988.] >” (FMNH, cAN).

*Data (R)*. Silvestri (1946).

Genus *Urolitus* Silvestri, 1947

*Urolitus nigeriensis* Silvestri 1947^§^

*Present classification*. Tachyporinae; Tachyporini; Euconosomatina; *Sepedophilus*.

*Data type*. R.

*Data (R)*. Silvestri (1947).

**Tribe Vatesini Seevers, 1958**

Genus *Vatesus* Sharp, 1876*

*Vatesus* *gigas* (Wasmann, 1909)

*Present classification*. Tachyporinae; Vatesini; *Vatesus*.

*Data type*. P + S.

*Data (P)*. 1 female: “ECUADOR: Napo Prov., | Zancudo (400 m), | 23 [handwritten] II 2009, | Maruyama & Komatsu leg.” < printed in rectangular small white label >, “*Vatesus* | *gigas* | (Wasmann 1909) | Host: *Eciton rapax* | Smith, 1855 | Det. M. Maruyama, 2017” < printed in rectangular small white label >, < with an additional glass card mounted on the abdominal terminalia > (KUM).

*Data (S)*. 1 male: “ECUADOR: Napo Prov., | Zancudo (400 m), | 23 [handwritten (copied)] II 2009, | Maruyama & Komatsu leg.” < printed in rectangular small white label, directly pasted on the slide >, “*Vatesus* | *gigas* | (Wasmann 1909) | Host: *Eciton rapax* | Smith, 1855 | Det. M. Maruyama, 2017” < printed in rectangular small white label, directly pasted on the slide > (FMNH).

*Vatesus* *praedatorius* Seevers, 1958

*Present classification*. Tachyporinae; Vatesini; *Vatesus*.

*Data type*. P + S.

*Data (P)*. 1 male, 1 female [each attached with a possible host ant, mounted on a separated paper card]: “PANAMA: Canal Zone | Madden Forest | Pres., VI. 15. [15, handwritten] 1976 | A. Newton” < printed in rectangular small white label >, “in emigration | column” < printed in rectangular small white label, not dissected > (FMNH).

*Data (S)*. 1 male, 1 female: “♂♀ A | Vatesus | praedatorius | Seevers | PAN.: C. Z. | Madden Pres. | VI. 15. 1976 in | emigration col. | A. Newton | PM” < directly handwritten on the slide>, < with additional comment: “aed. on side | (pm → R?) [This means the aedeagus on its side in the abdomen, with the parameres facing the right side of the beetle]” [directly handwritten on the slide] > (FMNH, cAN).

**Outgroup:**

**Family Silphidae Latreille, 1806**

Subfamily Silphinae Latreille, 1806

Genus *Silpha* Linnaeus, 1758*

*Silpha perforata* Gebler, 1832

*Data type*. P + S + W.

*Data (P)*. 1 male, 2 females: “[JAPAN: HOKKAIDÔ] | Kamishihoro-chô, Lake | Nukabira-ko [near Gonosawa Riv.] | (北海道上士幌町糠平湖) | 13 July 2014. | Shûhei YAMAMOTO leg.” < printed in rectangular small white label >, “*Silpha* | *perforata* | Gebler, 1832 | Det. S. Yamamoto, 2014” < printed in rectangular small white label, not dissected > (FMNH).

*Data (S)*. 1 male [divided in three slides]: “JAPAN: Hokkaido, | Kamishihoro-cho, | Nukabira-ko Lake, [near Gonosawa Riv.] | 13 VII 2014, S. Yamamoto | leg. (PT)” < printed in rectangular small white label, directly pasted on the slide >, “*Silpha* | *perforata* | Gebler, 1832 | det. S. Yamamoto, 2014” < printed in rectangular small white label, directly pasted on the slide > (FMNH).

*Data (W)*. 1 male: “[JAPAN: HOKKAIDÔ] | Kamishihoro-chô, Lake | Nukabira-ko < near Gonosawa Riv. > | (北海道上士幌町糠平湖) | 13 July 2014. | Shûhei YAMAMOTO leg.” < printed in rectangular small white label >, “*Silpha* | *perforata* | Gebler, 1832 | Det. S. Yamamoto, 2014” < printed in rectangular small white label, not dissected >, “Nukabira, Hokkaido, Japan | 13. vii. 2014, S. Yamamoto lg.” < handwritten in strongly transverse, rectangular white label >, “Silpha perforata | det. S. Yamamoto, 2017” < handwritten in strongly transverse, rectangular white label, not dissected > (FMNH).

**Family Staphylinidae Latreille, 1802**

*Omaliine Group of subfamilies*

**Subfamily Neophoninae Fauvel, 1905**

Genus *Neophonus* Fauvel, 1905*

*Neophonus bruchi* Fauvel, 1905^§^

*Data type*. P + S.

*Data (P)*. 1 male [male genitalia fully exposed]: “CHILE: Malleco Prov., | Puren Natur. Mon., | Contulmo, 350m, | 11.XII.1984 - | 13.II.1985” < printed in rectangular small white label >, “FMHD#85-902, mixed | evergreen forest, S. | Peck, P#85-16, FIT | FIELD MUSEUM NAT HIST” < printed in rectangular small white label, not dissected > (FMNH); 1 female: “CHILE: Chiloé Pr.: Chiroé | I., Ahoni Alto, 70m, | 22.ii.1988, primary forest, | L. Masner leg. | FIELD MUS. NAT. HIST.” < printed in rectangular small white label >, “Neophonus [handwritten] | bruchi [handwritten] | det. Newton 2003” < printed in rectangular small white label, not dissected > (FMNH).

*Data (S)*. 1 male, 1 female: “♂♀ | Neophonus | bruchi Fauv. | det. M. Thayer 1983 [3, handwritten]” < printed in rectangular small white label, directly pasted on the slide >, “CHILE: Osorno Prov. | 7.7km NE Termas de | Puyehue, 200m, site | 664, 19-25.xii.1982 | Valdivian rainfor. | A.Newton & M.Thayer” < printed in rectangular small white label, directly pasted on the slide >, “window | trap 664 [664, handwritten]”, < with additional comment: “more gut → PVLP | ♂ - several clumps | gut contents | L of legs III & abd. | app no Laboulb. on ♂ | or ♀ g.s. or A8 [This means there are more gut contents on a separate slide in PVLP (polyvinyl lactophenol); male with several clumps of gut contents to the left of the hindlegs and abdomen on this slide; apparently no Laboulbeniales on male or female genital segments or abdominal segment 8]” [directly handwritten on the slide] > (FMNH, cAN).

**Subfamily Omaliinae MacLeay, 1825**

Tribe Omaliini McLeay, 1825

Genus *Omalium* Gravenhorst, 1802*

*Omalium rivulare* (Paykull, 1789)^§^

*Data type*. P + S.

*Data (P)*. 1 male: “ILLINOIS | Clark County | Rocky Branch | 27 Apr 1998 | M. A. Goodrich” < printed in rectangular small white label >, “under bark of | dead tree” < printed in rectangular small white label >, “FIELD MUSEUM | ex EIUC [EIUC, handwritten] | (retained duplic.)” < printed in rectangular small white label >, “Omalium | rivulare | (Paykull) | Thayer det. 1998” < printed in rectangular small white label >, “♂” < printed in rectangular small white label, not dissected > (FMNH); 1 female: “MICH.:Berrien Co.; | 1/4mi.S. Mud Lake Bog | nr. Three Oaks, VI:8: | 1966 leg. S. Peck” < printed in rectangular small white label >, “FMNH(HD)#66-160 | carrion trap | in forest” < printed in rectangular small white label, not dissected > (FMNH); 1 female: “OH: Wayne Co | Wooster | 26-V-1978” < handwritten in rectangular small white label, not dissected > (FMNH).

*Data (S)*. 1 male, 1 female: “FIELD MUSEUM NAT. HIST. | Omalium ♂♀ [handwritten] | rivulare (Payk.) [handwritten] | USA: INDIANA: LaPorte [handwritten] | Co., Michiana 8.VI.1966 [handwritten] | wet forest carrion [handwritten] | trap S. Peck [handwritten]” < printed seal directly pasted on the slide>, < with additional green round seal on the right side: “22 | VII.89 [handwritten; it means that the slide was made by Al. Newton on July 22, 1989.] >” (FMNH, cAN).

*Oxyteline Group of subfamilies*

**Subfamily Osoriinae Erichson, 1839**

Tribe Thoracophorini Reitter, 1909

Subtribe Lispinina Bernhauer & Schubert, 1910

Genus *Lispinus* Erichson, 1839

*Lispinus quadripunctulus* Fauvel, 1864

*Data type*. P + S.

*Data (P)*. 1 male, 1 female, 2 unsexed: “PANAMA: Canal Zone | Barro Colorado Is. | February 8,25 1976 | A. Newton” < printed in rectangular small white label >, “under bark | 6 mos. dead [This means 6 months dead (tree)]” < printed in rectangular small white label >, “Lispinus | quadripunctulus | det. U. Irmler” < printed in rectangular small white label >, < with an additional glass card mounted on the abdominal terminalia for the single male specimen > (FMNH).

*Data (S)*. 1 male, 2 females: “♂♀ A | Lispinus | PANAMA: C. Z. | Barro Col. Isl. | Feb. 4, 1976 | und. bark, | rotting logs | AFN PM” < directly handwritten on the slide>, < with additional comment: “aed. pm. rent.” [directly handwritten on the slide] > (FMNH, cAN); 3 females [divided in three slides]: “PANAMA: Canal Zone | Barro Colorado Is. | February 8,25 1976 | A. Newton” < printed in rectangular small white label, directly pasted on the slide >, “under bark | 6 mos. dead” < printed in rectangular small white label, directly pasted on the slide >, “Lispinus | quadripunctulus | det. U. Irmler” < printed in rectangular small white label, directly pasted on the slide > (FMNH).

*Staphylinine Group of subfamilies*

**Subfamily Pseudopsinae Ganglbauer, 1895**

Genus *Pseudopsis* Newman, 1834*

*Pseudopsis montoraria* Herman, 1975

*Data type*. P + S + R.

*Data (P)*. 2 males, 2 females: “USA: CA: Marin Co., Lily | Gulch, W side Alpine | Lake, 220m, 37°57'N, | 122°38'W, 6.xi.1993, | Sequoia-Acer forest near | pond” < printed in rectangular small white label >, “FMHD #93-126, Winkler | extract, forest leaf and | log litter, A. Newton & | M. Thayer 937 | FIELD MUS. NAT. HIST.” < printed in rectangular small white label, not dissected > (FMNH).

*Data (S)*. 1 male, 1 female: “♂♀ A | Pseudopsis | montoraria | Herman | CAL.: Amador Co | Peddler Hill, | 7000’, VI.27.1975 | berl., litter, mixed | conifer forest | AFN PM” < directly handwritten on the slide> (FMNH, cAN).

*Data (R)*. Herman (1975).

**Subfamily Staphylininae Latreille, 1802**

Tribe Staphylinini Latreille, 1802

Subtribe Quediina Kraatz, 1857

Genus *Quedius* Stephens, 1829

Subgenus *Distichalius* Casey, 1915

*Quedius* (*Distichalius*) *paradisi* Hatch, 1957

*Data type*. P + S.

*Data (P)*. 1 male, 1 female: “WASH:Pierce Co., | Mt. Rainier N.P. | Paradise 5420’ | VII.18.1975 | A. Newton, M. Thayer” < printed in rectangular small white label >, “under rocks | and logs, | timberline” < printed in rectangular small white label, not dissected > (FMNH); 1 male, 1 female: “WASH:Pierce Co., | Mt. Rainier N.P. | 1.6mi SW Paradise | 5000’ VII.18.1975 | A. Newton, M. Thayer” < printed in rectangular small white label >, “under bark | conifer” < printed in rectangular small white label, not dissected > (FMNH).

*Data (S)*. 1 male, 1 female: “♂♀ A | Quedius (Dist.) | paradisi Hatch | (pm ←) | ORE.: Hood R. Co. | Mt. Hood Mdws. [Meadows] | 5200’, VII.10.1975 | under conifer bark | AN / MT | PM” < directly handwritten on the slide> (FMNH, cAN).

*Tachyporine Group of subfamilies*

**Subfamily Aleocharinae Fleming, 1821**

Tribe Gymnusini Heer, 1839

Genus *Gymnusa* Gravenhorst, 1806*

*Gymnusa atra* Casey, 1911

*Data type*. P + S + R.

*Data (P)*. 1 female: “Duparquet, Que. | 3. VI. [handwritten] 1941 [1, handwritten] | G. Stace Smith” < printed in rectangular small white label >, “Gymnusa [handwritten] | ?= atra [handwritten] | det. Newton 1991” < printed in rectangular small white label, not dissected > (FMNH).

*Data (S)*. 1 male, 1 female: “♂♀ A | Gymnusa | ?atra Csy. | det. MKT ‘81 | no data- | Liebeck | colln. ? | PM” < directly handwritten on the slide>, < with additional comment: “aed. pm. dorsal” [directly handwritten on the slide] > (FMNH, cAN).

*Data (R)*. Klimaszewski (1979); Klimaszewski *et al*. (2018).

Tribe Oxypodini Thomson, 1859

Subtribe Oxypodina Thomson, 1859

Genus *Oxypoda* Mannerheim, 1830*

Subgenus *Oxypoda* Mannerheim, 1830

*Oxypoda* (*Oxypoda*) sp.

*Data type*. P + S.

*Data (P)*. 1 male, 1 female, 1 unsexed: “USA:Vt. Chitten- | den Co. Essex Jct. | (10miS) mixed for- | est 19-viii-1974” < printed in rectangular small white label >, “FM(HD) #74-228 ex | beech buttress | W. Suter” < printed in rectangular small white label, not dissected > (FMNH).

*Data (S)*. 1 male, 1 female [divided in two slides]: “USA:Vt. Chitten- | den Co. Essex Jct. | (10miS) mixed for- | est 19-viii-1974” < printed in rectangular small white label, directly pasted on the slide >, “FM(HD) #74-228 ex | beech buttress | W. Suter” < printed in rectangular small white label, directly pasted on the slide >, “*Oxypoda* sp. | Det. J. Klimaszewski, | 1993.” (FMNH).

**Subfamily Habrocerinae Mulsant & Rey, 1877**

Genus *Habrocerus* Erichson, 1839*

*Habrocerus capillaricornis* (Gravenhorst, 1806)^§^

*Data type*. P + S + R.

*Data (P)*. 1 male, 1 female: “Penn.: Somerset Co. | 1 mi S Kantner | 10-VI-1976” < printed in rectangular small white label >, “berlese forest | litter LEWatrous” < printed in rectangular small white label >, “Habrocerus | capillaricornis | Gravenhorst | det. Assing, 1992” < printed in rectangular small white label, not dissected > (FMNH).

*Data (S)*. 2 males, 1 female: “♂♀ FM [handwritten in blue ink] A | Habrocerus | capillaricornis | (Gr.) | MASS.: Mdsx. Co. | Cambridge | VIII.20.1977 | berl., wood chip | pile, AFN, MKT | PM” < directly handwritten on the slide> (FMNH, cAN).

*Data (R)*. Assing & Wunderle (1995).

Genus *Nomimocerus* Coiffait & Saiz, 1965

*Nomimocerus marginicollis* (Solier, 1849)^§^

*Data type*. P + S + R.

*Data (P)*. 1 male, 1 female: “CHILE: Aysen Prov., 34 | km W Pto. Aysen, San | Sebastian, 150m, | 24.I.1985” < printed in rectangular small white label >, “FMHD#85-986, cliffbase | mixed forest bamboo | litter, S.&J. Peck, | P#85-103, berlese | FIELD MUSEUM NAT HIST” < printed in rectangular small white label, not dissected > (FMNH).

*Data (S)*. 1 male, 1 female: “♂♀ FM [handwritten in red ink] A | Nomimocerus | marginicollis | (Solier)” < directly handwritten on the slide>, “CHILE: Osorno Prov. | Parque Nac. Puyehue, | 4.1 km E Anticura, | 430m, trap site 662 | 19-26.xii.1982 | valdivian rainfor. | A. Newton & M. Thayer” < printed in rectangular small white label, directly pasted on the slide >, “berl. forest | leaf & log | litter” < printed in rectangular small white label, directly pasted on the slide >, < with additional comment: “PM” [directly handwritten on the slide] > (FMNH, cAN).

*Data (R)*. Assing & Wunderle (1995).

**Subfamily Olisthaerinae Thomson, 1858**

Genus *Olisthaerus* Dejean, 1833*

*Olisthaerus substriatus* (Paykull, 1790)^§^

*Data type*. P + S.

*Data (P)*. 2 males [single male with its abdominal terminalia removed, and glued on the paper card near the body], 1 female, 1 unsexed: “REPUBLIC of ALTAI | Ongudaysky distr., 10 km SE of | Semlinsky pass | 15.VI.1999 leg. A.G. Kirejchuk” < printed in rectangular small white label >, “Olisthaerus [handwritten] | substriatus (Gyll.) [handwritten] | A.Solodovnikov det. 1999” < printed in rectangular small white label >, < with additional glass cards mounted on the abdominal terminalia for the male and female specimens > (FMNH).

*Data (S)*. 1 male [body without its elytra and hindwings; aedeagus not visible]: “♂ A | Olisthaerus | substriatus | (Gyll.) | “N.H.” | FM [handwritten in red ink] | CB” < directly handwritten on the slide> (FMNH, cAN); 1 male [elytra and hindwings only]: “♂ W | Olisthaerus | substriatus | (Gyll.) | “N.H.” | CB” < directly handwritten on the slide> (FMNH, cAN); 1 female: “♀ A | Olisthaerus | substriatus | (Gyll.) | N.H.: Coos Co. | Jefferson Notch | 3000’ | IX.19-20.1974 | under conifer | bark JFL | CB” < directly handwritten on the slide> (FMNH, cAN).

**Subfamily Phloeocharinae Erichson, 1839**

Genus *Phloeocharis* Mannerheim, 1830*

Subgenus *Phloeocharis* Mannerheim, 1830

*Phloeocharis* (*Phloeocharis*) *californica* Smetana & Campbell, 1980

*Data type*. P + S.

*Data (P)*. 1 male [abdominal terminalia glued on the paper card near the body]: “CAL: Tulare Co., 1.8mi. S | Kaveah Camp 5800’ | 13.V.1976, A.Newton + | M. Thayer, berlese litter | Sequoia + conif. forest” < handwritten in rectangular small white label >, “VOUCHER | Associated | with larva” < printed in rectangular small blue label >, “PARATYPE | Phloeocharis [handwritten] | californica [handwritten] | Smetana & Campbell [handwritten]” < printed in rectangular small yellow label > (FMNH); 1 female: “Julia Pfeiffer- | Burns St. Pk., Calif. | Mont. Co., VI-13-1972 | DSChandler&DPLevin | colrs.” < printed in rectangular small white label >, “Berlese | Redwood duff” < printed in rectangular small white label >, “ALLOTYPE | Phloeocharis [handwritten] | californica Smetana | and Campbell [handwritten] | CNC No. 16048 [16048, handwritten]” < printed in rectangular small red label, not dissected > (FMNH).

*Data (S)*. 1 male, 1 female: “♂♀ A | Phloeocharis | californica | Smet. + Campb. 1980 | 19.III.1995 | FMHD 95-52, | 954 litter | ANMT PM <The complete data is as follows: “USA: CA: Monterey Co., Los Padres N.F., Skinner Ridge Trail at Mill Creek, 725 m, Quercus-Arbutus menziesii woodland, berl., leaf & log litter, ANMT site 954” >” < directly handwritten on the slide >, < with additional green round seal on the right side: “1.98 [handwritten; it means that the slide was made by Al. Newton on January 1998.] >” (FMNH, cAN); 1 male: “♂ FM [handwritten in red ink] A | Phloeocharis | californica | Smet. + Camp. | CAL.: Calaveras | Co., 3 mi NE | Glencoe, 2000’ | VI.25.1975 | berl., litter, oak- | conifer forest | A. Newton CB” < directly handwritten on the slide> (FMNH, cAN).

**Subfamily Trichophyinae Thomson, 1858**

Genus *Trichophya* Mannerheim, 1830*

*Trichophya pilicornis* (Gyllenhal, 1810)^§^

*Data type*. P + S.

*Data (P)*. 1 male, 1 female: “U.S.A.: OREGON. | Baker Co.; Pine Ck. nr. | Baker. Blue Mountains. | 2:VI:1957. Leg: H Dybas.” < printed in rectangular small white label >, “*Trichophya* | *pilicornis* | (Gyllenhal) | det. Newton 1992” < printed in rectangular small white label, not dissected > (FMNH).

*Data (S)*. 2 males, 2 females: “♂♀ A | *Trichophya* | *pilicornis* (Gyll.) | WASH.: King Co. | c. 10-12 mi N of No. | Bend, c. 1200 ft. 17. IV. 1983 2° | mixed conifer for. | P.J. + C. C. Johnson | aerial dredging | (afternoon) | PM” < directly handwritten on the slide > (FMNH, cAN).

**References**

Assing, V., Wunderle, P. (1995) A revision of the species of the subfamily Habrocerinae (Coleoptera: Staphylinidae) of the world. *Revue Suisse de Zoologie* 102: 307–359.

Cameron, M. 1928. New species of Staphylinidae from Borneo. *Sarawak Museum Journal* **3:** 423–451.

Campbell, J.M. (1973a) A revision of the genus *Tachinomorphus* (Coleoptera: Staphylinidae) of North and Central America. *Canadian Entomologist* 105: 1015–1034.

Campbell, J.M. (1973b) A revision of the genus *Tachinus* (Coleoptera: Staphylinidae) of North and Central America. *Memoirs of the Entomological Society of Canada* 90: 1–137.

Campbell, J.M. (1975) A revision of the genera *Coproporus* and *Cilea* (Coleoptera: Staphylinidae) of America north of Mexico. *Canadian Entomologist* 107: 175–216.

Campbell, J.M. (1976) A review of the Tachyporine genus *Euconosoma* Cameron (Coleoptera: Staphylinidae) with a description of a new species from Nepal. *Coleopterists Bulletin* 30: 139–145.

Campbell, J.M. (1980) A revision of the genus *Carphacis* des Gozis (Coleoptera: Staphylinidae) of North America. *Canadian Entomologist* 112: 935–953.

Campbell, J.M. (1982) A revision of the genus *Lordithon* Thomson of North and Central America (Coleoptera: Staphylinidae). *Memoirs of the Entomological Society of Canada* 119: 1–116.

Campbell, J.M. (1991) A revision of the genera *Mycetoporus* Mannerheim and *Ischnosoma* Stephens (Coleoptera: Staphylinidae: Tachyporinae) of North and Central America. *Memoirs of the Entomological Society of Canada* 156: 1–169.

Campbell, J.M. (1993a) A revision of the genera *Bryoporus* Kraatz and *Bryophacis* Reitter and two new related genera from America North of Mexico (Coleoptera: Staphylinidae: Tachyporinae). *Memoirs of the Entomological Society of Canada* 166: 1–85.

Campbell, J.M. (1993b) A review of the species of *Nitidotachinus* new genus (Coleoptera: Staphylinidae: Tachyporinae). *Canadian Entomologist* 125: 521–548.

Campbell, J.M. (1994a) *Cileoporus*, a new genus of rove beetles from Central and South America (Coleoptera, Staphylinidae, Tachyporinae). *Studies on Neotropical Fauna and Environment* 29: 125–144.

Campbell, J.M. (1994b) A revision of the genus *Coprotachinus* Cameron (Coleoptera: Staphylinidae: Tachyporinae). *Bulletin de l'Institut Royal des Sciences Naturelles de Belgique. Entomologie* 64: 25–47.

Hayashi, Y. (2003) Notes on *Peitawopsis* (Coleoptera: Staphylinidae: Tachyporinae), with a description of a new species from Japan. *Entomological Review of Japan* 58: 113–119.

Herman, L.H. (1975) Revision and phylogeny of the monogeneric subfamily Pseudopsinae for the world (Staphylinidae: Coleoptera). *Bulletin of the American Museum of Natural History* 155: 241–318.

Herman, L.H. (2001) Catalog of the Staphylinidae (Insecta: Coleoptera). 1758 to the end of the second millennium. Parts I–VII. *Bulletin of the American Museum of Natural History* 265: 1–4218.

Herman, L.H. (2004) Revision of the Asian tribe Megarthropsini (Coleoptera: Staphylinidae: Tachyporinae). *American Museum Novitates* 3430: 1–72.

Kishimoto, T. (2001) A new species of the genus *Derops* (Coleoptera, Staphylinidae) on the island of Iriomote-jima, Southwest Japan. *Elytra, Tokyo* 29: 175–179.

Klimaszewski, J. (1979) A revision of the Gymnusini and Deinopsini of the world. Coleoptera: Staphylinidae, Aleocharinae. *Agriculture Canada Monograph* 25: 1–169.

Klimaszewski, J., Webster, R.P., Langor, D.W., Brunke, A., Davies, A., Bourdon, C., Labrecque, M., Newton, A.F., Dorval, J-A., Frank, J.H. (2018) *Aleocharine rove beetles of eastern Canada (Coleoptera, Staphylinidae, Aleocharinae): a glimpse of megadivetsity*. Cham: Springer.

Maruyama, M. (2004) A permanent slide pinned under a specimen. *Elytra, Tokyo* 32: 276.

Naomi, S.-I. (1986) On the genus *Derops* Sharp (Staphylinidae) and its systematic position. *Nature and Insect, Tokyo* 21: 17–22. (in Japanese, with English title)

Newton, A.F. Jr. (1984) Mycophagy in Staphylinoidea (Coleoptera) In: Wheeler Q, Blackwell M, eds. *Fungus-insect relationships: perspectives in ecology and evolution*. New York: Columbia University Press, pp. 302–353.

Sanderson, M.W. (1947) A new genus of Nearctic Staphylinidae (Coleoptera). *Journal of the Kansas Entomological Society* 19: 130–133. [Note: nominal year of publication 1946]

Schülke, M. (2003) Eine neue Art der Gattung *Symmixus* Bernhauer, mit Bemerkungen zur Tribus Symmixini Bernhauer (Coleoptera, Staphylinidae, Tachyporinae). *Linzer biologische Beiträge* 35: 443–452.

Schülke, M. (2003) Revision der paläarktischen Arten der Gattung *Bryophacis* Reitter, mit Bemerkungen zu *Bolitobius biseriatus* Mannerheim und *Bolitobius filicornis* Wollaston (Coleoptera, Staphylinidae, Tachyporinae). *Linzer biologische Beiträge* 36: 1001–1054.

Schülke, M. (2005) Zwei neue Arten der Gattung *Pseudotachinus* Cameron aus dem Himalaja-Gebiet (Coleoptera, Staphylinidae, Tachyporinae). *Linzer biologische Beitrage* 37: 1609–1624.

Schülke, M. (2005) Zur Kenntnis Gattung *Tachinoplesius* Bernhauer (Coleoptera, Staphylinidae, Tachyporinae). *Linzer biologische Beitrage* 38: 889–901.

Schülke, M. (2010) Zur Verbreitung und subspezifischen Gliederung von *Bolitobius* *castaneus* (Stephens 1832) (Coleoptera, Staphylinidae, Tachyporinae). *Linzer biologische Beitrage* 42: 1461–1480.

Steel, W.O. (1956) A new genus and species of Tachyporinae from Australia (Coleoptera: Staphylinidae). *Proceedings of the Royal Entomological Society of London (B)* 25: 13–16.

Silvestri, F. (1946) Primo contributo alla conoscenza dei termitofili viventi con specie de *Syntermes*. *Commentationes Pontificia Academia Scientiarum* 9: 515–559. [Note: nominal year of publication 1945]

Silvestri, F. (1947) Contributo alla conoscenza dei Termitodiscinae e Cephaloplectinae (Staphylinidae, Coleoptera) termitofili. *Archivio Zoologico Italiano* 31: 123–149. [Note: nominal year of publication 1943–1946]

Watanabe, Y. (1985) A revision of the Japanese species of *Derops* (Coleoptera, Staphylinidae). *Kontyu* 53: 436–451.
